# Novel Insights into Chromosome Evolution in Birds, Archosaurs, and Reptiles

**DOI:** 10.1093/gbe/evw166

**Published:** 2016-07-10

**Authors:** Marta Farré, Jitendra Narayan, Gancho T. Slavov, Joana Damas, Loretta Auvil, Cai Li, Erich D. Jarvis, David W. Burt, Darren K. Griffin, Denis M. Larkin

**Affiliations:** ^1^Department of Comparative Biomedical Sciences, Royal Veterinary College, Royal College Street, University of London, NW1 0TU, UK; ^2^Institute of Biological, Environmental and Rural Sciences, Aberystwyth University, SY23 3DA, UK; ^3^Illinois Informatics Institute, University of Illinois, Urbana, IL 61801, USA; ^4^China National GeneBank, BGI-Shenzhen, Shenzhen 518083, China; ^5^Centre for GeoGenetics, Natural History Museum of Denmark, University of Copenhagen, Copenhagen, 1350, Denmark; ^6^Department of Neurobiology, Duke University Medical Center, Durham, NC, 27710, USA; ^7^Howard Hughes Medical Institute, Chevy Chase, MD, 20815, USA; ^8^Department of Genomics and Genetics, the Roslin Institute and Royal (Dick) School of Veterinary Studies, University of Edinburgh, Midlothian EH25 9RG, UK; ^9^School of Biosciences, University of Kent, Canterbury CT2 7NJ, UK

**Keywords:** chromosome rearrangements, birds, reptiles, genome evolution, comparative genomics

## Abstract

Homologous synteny blocks (HSBs) and evolutionary breakpoint regions (EBRs) in mammalian chromosomes are enriched for distinct DNA features, contributing to distinct phenotypes. To reveal HSB and EBR roles in avian evolution, we performed a sequence-based comparison of 21 avian and 5 outgroup species using recently sequenced genomes across the avian family tree and a newly-developed algorithm. We identified EBRs and HSBs in ancestral bird, archosaurian (bird, crocodile, and dinosaur), and reptile chromosomes. Genes involved in the regulation of gene expression and biosynthetic processes were preferably located in HSBs, including for example, avian-specific HSBs enriched for genes involved in limb development. Within birds, some lineage-specific EBRs rearranged genes were related to distinct phenotypes, such as forebrain development in parrots. Our findings provide novel evolutionary insights into genome evolution in birds, particularly on how chromosome rearrangements likely contributed to the formation of novel phenotypes.

## Introduction

**Fig. 1.—:**
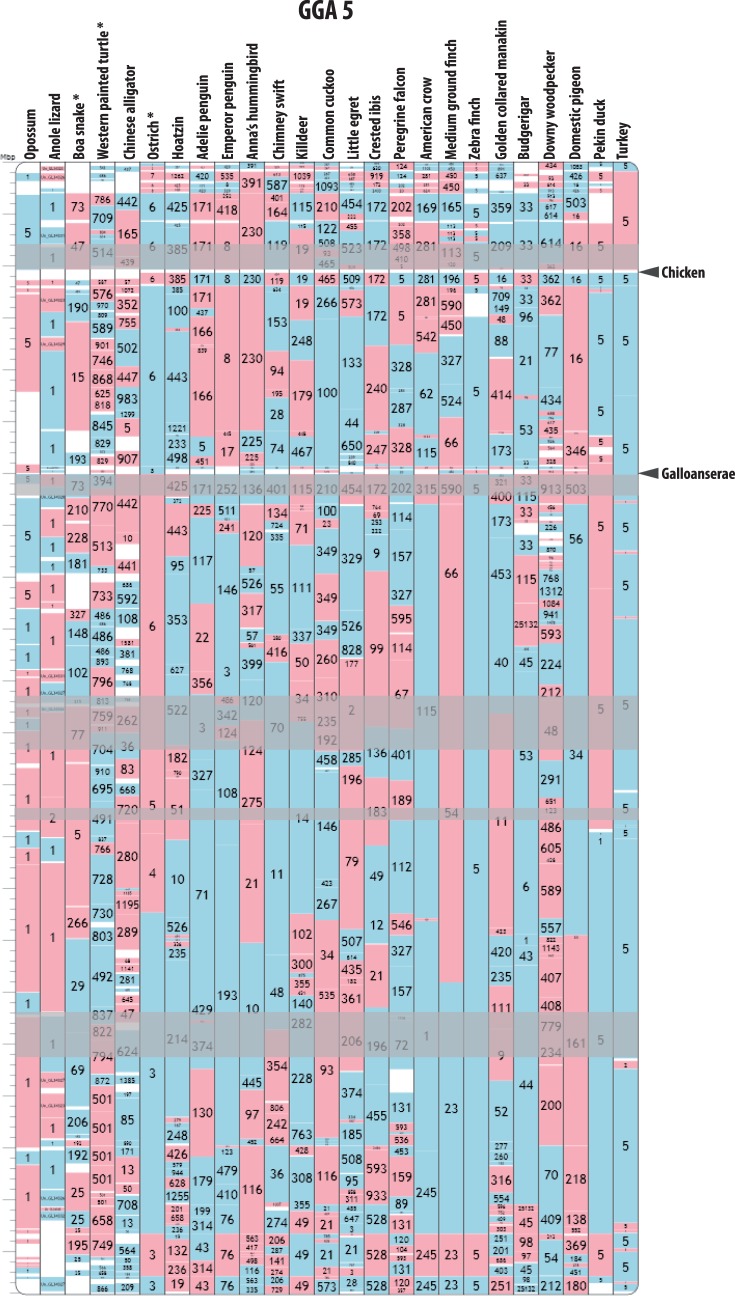
EBRs, SFs, and HSBs. Blue and red blocks define SFs in target genomes in “+” and “−” orientation, respectively compared to the chicken chromosome 5 defined at 100 kb resolution, with target species scaffold or chromosome numbers indicated inside the blocks. Only the columns with genomes assembled to chromosomes (turkey, duck, zebra finch, Anole lizard, and opossum) contain complete HSBs while blocks in the remaining columns represent either HSBs or SFs. EBRs are defined as white intervals in between either two adjacent SFs originating from the same scaffold in a target genome or two adjacent HSBs. Reference-specific EBRs are represented by the white intervals that overlap in all species. The arrowheads point to a chicken-specific and a Galloanserae-specific EBRs. Pale grey boxes demarcate avian msHSBs that are >1.5 Mbp in the chicken genome. Asterisks demark genomes with modified scaffold IDs for better visibility. All reference chromosome and target genome alignments are available from the avian Evolution Highway website: http://eh-demo.ncsa.uiuc.edu/birds.

A prominent feature of animal genome evolution is the nonrandom rearrangement of chromosomes (Pevzner and Tesler 2003). For millions of years genomes of multiple species have maintained homologous synteny blocks (HSBs), demarcated by dynamic “evolutionary breakpoint regions” (EBRs) (fig. 1). Evidence suggests that each of them evolves by distinctly different mechanisms (Larkin et al. 2009): HSBs maintain the order of genes related to organismal development whereas EBRs often affect chromosomal regions related to lineage-specific biology (Groenen et al. 2012; Ullastres et al. 2014). These data are somewhat mammal-centric and conclusions thus may not hold for other amniotes. While the availability of genetic maps and chromosome assemblies of the chicken, turkey, and zebra finch genomes provided an important insight into avian chromosome evolution (Burt et al. 1999; Völker et al. 2010; Warren et al. 2010), a comprehensive study at the sequence level is lacking, making unclear if bird chromosomes follow similar patterns of evolution as their mammalian counterparts.

Birds have more compact genomes with shorter intronic and intergenic regions than mammals (ICGSC 2004; Zhang et al. 2014). The proportion of repetitive DNA in bird genomes is ∼15% (ICGSC 2004; Zhang et al. 2014), whereas in mammals it is ∼50% (Lander et al. 2001). Birds have more gene families that lost paralogs than other amniotes (Huang et al. 2013; Lovell et al. 2014). Avian karyotypes have been maintained without interchromosomal changes for millions of years (Romanov et al. 2014) and are less variable than those of mammals (Ellegren 2010; Ruiz-Herrera et al. 2012) with a characteristic 2*n* = ∼80 in most species (Griffin et al. 2007).

Using a new EBR-detection approach applied to 21 bird genomes assembled to whole chromosomes or large scaffolds (Zhang et al. 2014), and four nonavian reptile genomes of similar quality, we examined the association of EBRs and multispecies HSBs (msHSBs) with gene networks, transposable elements (TEs), and conserved noncoding sequences. We identified gene networks that: 1) were preferentially reshuffled during avian chromosome evolution, or 2) have been maintained in msHSBs for millions of years of evolution. Our results represent the first comprehensive sequence analysis of chromosome evolution in birds and reptiles, demonstrating how chromosome evolution may have acted upon the formation of various phenotypes.

## Results and Discussion

### Lineage-Specific EBRs in Birds

We developed an interactive resource for genome synteny comparison in 26 species (Evolution Highway; http://eh-demo.ncsa.uiuc.edu/birds; supplementary table S1, Supplementary Material online). We aligned 20 avian and five outgroup genomes to the chicken genome to define syntenic fragments (SFs) at three resolutions of rearrangement detection: 100, 300, and 500 kb (fig. 1). We developed and evaluated (supplementary tables S2–S4, Supplementary Material online) a method of detecting EBRs within scaffolds of scaffold-based assemblies that combines an algorithmic approach to identify putative EBRs (supplementary table S5, Supplementary Material online) with independent PCR verification of these regions in several assemblies to find paired read spanning levels in scaffolds associated with confirmed EBRs in order to estimate and minimise the number of chimeric joints in the final EBR list (supplementary tables S5 and S8, Supplementary Material online). This resulted in 0–22% false positives and 33–45% false negatives in our EBR set, depending on the sequencing coverage of each assembly (supplementary table S7, Supplementary Material online). At 100 kb resolution 1,796 avian EBRs were assigned to phylogenetic nodes and 1,021 (56.85%) passed our chimeric scaffold detection quality controls. Out of 1,021 EBRs, 42 were specific to all Galliformes, and 16 were specific to the chicken lineage (fig. 1 and supplementary table S5, Supplementary Material online). We detected a total of 874 lineage-specific EBRs, that is, assigned to lineages leading to each species in our set after the divergence from the most recent common ancestor with other included species (supplementary table S5, Supplementary Material online).

### Lineage-Specific EBRs are Enriched in TEs in Birds

**Fig. 2.—:**
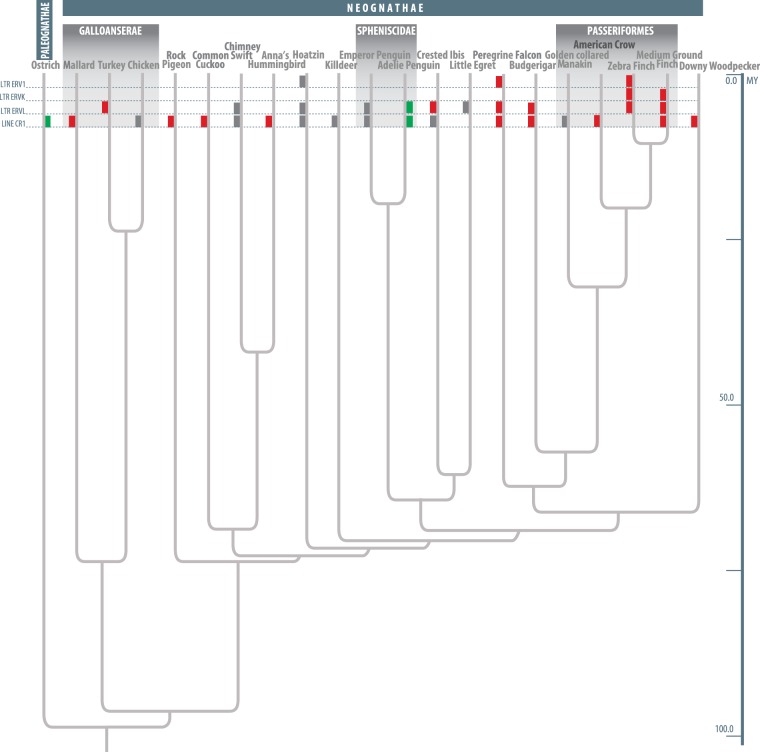
Relationship between lineage-specific EBRs and TEs in avian species. The phylogenetic tree is based on (Jarvis et al. 2014). Red bars indicate a significant enrichment of TEs from one or more abundant avian TE families (LINE-CR1, LTR-ERVL, LTR-ERVK, and LTR-ERV1) in lineage-specific EBRs (*P* value <0.05; FDR < 10%); green bars show significant negative associations of TEs with lineage-specific EBRs (*P* value <0.05; FDR < 10%); and grey bars indicate elevated numbers of the TE families in lineage EBRs (higher number of TEs in EBRs compared to the rest of the genome but not reaching a significance level of *P* value <0.05 and FDR < 10% likely due to a low number of lineage-specific EBRs resulting in low power of the statistical test).

In mammals, lineage- and order-specific EBRs are enriched for TEs that were active at the time of lineage/order formation (Larkin et al. 2009; Schibler et al. 2006; Groenen et al. 2012), and TEs can promote chromosome rearrangements by nonallelic homologous recombination (Bailey et al. 2004). In birds, we found that one or more of four families of TEs (LINE-CR1, LTR-ERVL, LTR-ERVK, and LTR-ERV1) were significantly enriched in lineage-specific EBRs among 19 bird species (>100 bp on average in the EBR- or nonEBR-containing nonoverlapping 10 kb genome intervals; false discovery rate (FDR) < 10%; fig. 2). The only exceptions were ostrich and Adelie penguin lineage-specific EBRs, which had a significant negative association with the LINE-CR1 elements and LINE-CR1 and LTR-ERVL elements, respectively, implying the presence of still unidentified lineage-specific TEs associated with EBRs in these two species. Our findings suggest that lineage-specific EBRs are associated with the presence of TE elements in birds, following the trend previously reported for mammals (Groenen et al. 2012).

### msHSBs in Avian and Reptile Genomes

To evaluate if msHSBs were maintained during bird evolution, five sets of msHSBs (the regions of genomes that were not interrupted by EBRs; supplementary tables S10 and S11, Supplementary Material online) were defined: avian, archosaurian, archosaurian/testudines, sauropsid, and amniote. We detected 1,746 avian msHSBs, covering 76.29% of the chicken genome. Using the Kolmogorov–Smirnov test, the distribution of msHSB sizes was tested for goodness-of-fit to an exponential distribution, following previous publications (Pevzner and Tesler 2003; Larkin et al. 2009). We detected 21 msHSBs longer than the maximum lengths expected from a random distribution of EBRs (supplementary tables S10 and S11, Supplementary Material online), indicating that large msHSBs could be maintained in evolution of bird and other reptile genomes (supplementary table S10, Supplementary Material online). Six amniote-, four sauropsid-, three archosaurian/testudines-, three archosaurian-, and five avian-msHSBs were significantly longer than would be expected from a random distribution of EBRs (supplementary table S10, Supplementary Material online).

To unravel the potential functional role of msHSBs in reptilian genomes we asked whether msHSBs were enriched in avian conserved noncoding elements (CNEs), many of which are gene regulatory sequences or miRNA (Zhang et al. 2014), and chicken genes. All five msHSB sets were highly enriched (*P* value <3e−12) in avian CNEs, with a ratio between CNE base pairs in msHSBs and other genome intervals ranging from 1.45 for avian to 1.62 for archosaurian/testudines msHSBs (table 1). The density of chicken genes in all msHSBs followed the opposite trend, with msHSBs having significantly fewer genes than other genome intervals (ranging from 0.58 for avian msHSBs to 0.74 for sauropsid and amniote ones; *P* value <3e−12; table 1). To test if CNEs enrichment in msHSBs is not due to the reduction in the number of genes in msHSBs, we renamed all coding bases as additional CNE bases within the 91,947 windows in the chicken genome used to analyze the CNE density. We compared the original and obtained CNE densities in each window and found that the increment was very low with an average genome-wide ratio of the obtained to the real CNE bases of 1.02. We repeated this experiment for msHSB windows and nonmsHSB windows separately and observed very similar values (1.02 for both). These values are much lower than the ratio of CNE bases in msHSBs compared to other genome intervals (table 1), suggesting that the enrichment of CNEs in msHSBs detected is not due to the lack of genes in msHSBs. Overall, msHSBs in birds and other reptiles are gene-sparse but enriched for bird-specific nonrandomly conserved DNA sequences (table 1). Avian and reptile msHSBs lack coding genes but are enriched in CNEs, and at least the largest msHSBs are nonrandomly maintained in evolution. This likely reflects the existence of selection against chromosome rearrangements in some avian genome intervals.

**Table 1:** Density per 10 kb Window of CNEs and Genes in msHSBs and Other Genome Intervals

msHSB set	Genes*						CNEs*					
	All msHSBs			msHSBs > 1.5 Mbp			All msHSBs			msHSBs > 1.5 Mbp		
	msHSBs	Other	Ratio	msHSBs	Other	Ratio	msHSBs	Other	Ratio	msHSBs	Other	Ratio
Avian	0.14	0.24	0.58	0.10	0.17	0.59	2.20	1.52	1.45	2.44	1.96	1.25
Archosaurian	0.14	0.20	0.70	0.10	0.17	0.59	2.33	1.47	1.58	2.58	1.96	1.32
Archosaurian/Testudines	0.14	0.20	0.70	0.11	0.17	0.65	2.35	1.45	1.62	2.49	1.98	1.26
Sauropsid	0.14	0.19	0.74	0.12	0.17	0.71	2.45	1.55	1.58	2.60	1.99	1.31
Amniote	0.14	0.19	0.74	0.12	0.17	0.71	2.44	1.58	1.54	2.36	2.01	1.17

* All differences are statistically significant (raw *P* values <0.0000000001).

### Signatures of Gene-Functional Enrichment in msHSBs

**Fig. 3.—:**
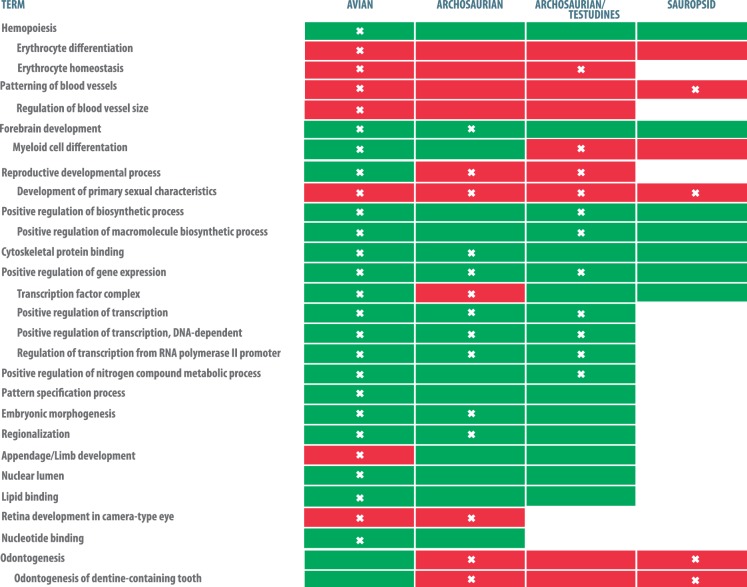
GO terms enriched in four sets of msHSBs. Green boxes show a fold enrichment >1.3 while red boxes depict a fold enrichment >2. White crosses inside boxes show categories with FDR < 10%. Underlying data could be found in supplementary table S12, Supplementary Material online.

To identify if there are gene pathways associated with bird and/or reptile msHSBs we measured gene ontology (GO) enrichment in msHSBs. We analyzed msHSBs >1.5 Mbp in the chicken genome, covering from 8.03% to 18.12% of the genome in amniote and avian msHSBs, respectively and 10,830 genes with a single orthologue in human and chicken. We identified functional enrichment in all five sets of msHSBs (fig. 3 and supplementary table S12, Supplementary Material online; FDR < 10%).

The *development of primary sexual characteristics* term-related genes were significantly enriched in avian, archosaurian and archosaurian/testudines msHSB sets. Out of these 17 genes distributed across 12 chicken chromosomes, only one (*BMPR1B*) was found in an avian-specific msHSBs but absent from the remaining msHSB sets. *BMPR1B* plays a role in ovulation (Onagbesan et al. 2003), and in the formation of the bird three-digit limb (Welten et al. 2005). A bird-specific CNE found 100 bp upstream from *BMPR1B* contains two transcription factor binding sites (TFBSs) for *AP-1* (known as *cJun*) and *NF-E4*. The *AP-1* transcription factor superfamily plays a role in the regulation of apoptosis during limb development in chickens (Suda et al. 2014), and could account for the reported differences in expression of *BMPR1B* in birds compared to other vertebrates (Brawand et al. 2011). Therefore, the presence of this CNE containing a relevant TFBS could have contributed to the formation and stability of this msHSB in avian evolution.

*Appendage* and *limb development* genes (19 genes in 12 avian msHSBs on 8 chicken chromosomes) were significantly enriched in the avian msHSB set only. Five genes were in avian-specific msHSBs (*SHOX*, *DLX5*, *DLX6*, *HOXA11*, and *BMPR1B*). *DLX5* is under positive selection in birds (Zhang et al. 2014) and mis-expression in chicken embryos leads to feather fusions and loss (Rouzankina et al. 2004). In line with a previous study (Lowe et al. 2015) reporting CNEs near feather-related genes controlling the expression of these genes, we found a bird-specific CNE, containing a TFBS for TGGCA-binding proteins, 1.9 kb upstream of *DLX5*. The *HOXA11* gene is expressed during the proximodistal limb bud development leading to the formation of ulna and radius bones (Zeller et al. 2009), and is under positive selection in birds (Zhang et al. 2014). Overall, msHSBs are enriched for genes related to clade-specific phenotypes, suggesting a link between the formation of these genomic regions and clade-specific traits.

### Functional Categories of Genes in Lineage-Specific EBRs

To evaluate potential associations between gene functional groups and lineage-specific EBRs, we performed GO enrichment analysis in EBRs from the 21 bird genomes. Only EBRs from genomes assembled with the aid of maps and those that passed our chimeric scaffold quality control were included in this analysis (supplementary table S5, Supplementary Material online). We considered enriched GO terms those with genes in at least four EBRs per species to detect the terms affected by multiple chromosome rearrangements. Twenty-three categories were significantly enriched in EBRs in lineages leading to eight bird species (table 2 and supplementary table S13, Supplementary Material online).

**Table 2:** Gene Ontology Terms Enriched in Lineage-Specific EBRs

EBR classification	GO term	No. genes	No. EBRs	Fold-enrichment	FDR (%)
Budgerigar	Forebrain development	12	11	2.74	5.47
	Neuron differentiation	15	13	2.33	6.83
	Neuron development	12	11	2.62	8.19
	Response to wounding	11	11	2.77	8.35
Common cuckoo	Mitotic cell cycle	11	11	3.57	1.14
	Condensed chromosome	7	5	4.88	2.67
	M phase	10	9	3.25	4.50
Little egret	Passive transmembrane transport	10	5	4.15	0.59
	Cation channel activity	7	4	4.32	5.61
Anna’s hummingbird	Hexose metabolic process	10	8	2.90	9.70
Peregrine falcon	RNA degradation	6	6	6.13	2.29
	Soluble fraction	5	4	6.23	8.35
Downy woodpecker	Histidine metabolism	6	5	10.30	0.16

Note.—An extended version of this table, including the gene names in each GO term is the supplementary table S13, Supplementary Material online.

**Fig. 4.—:**
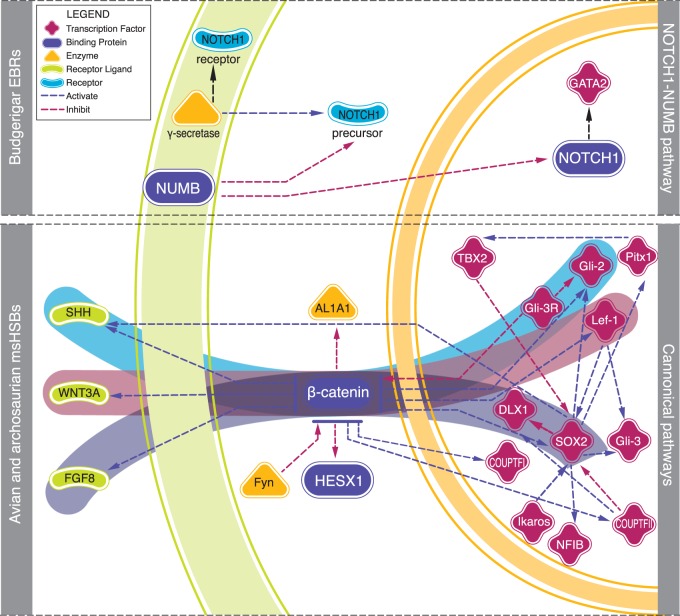
Gene pathways related to forebrain development in budgerigar lineage-specific EBRs and avian and archosaurian msHSBs. Budgerigar lineage-specific EBRs (top box) are enriched for genes related to the *NOTCH1-NUMB* pathway, while avian and archosaurian msHSBs (bottom box) for genes related to three conserved canonical pathways (*SHH* pathway in blue, *WNT3* pathway in pink and *FGF8* pathway in purple). The function of each protein is indicated in the legend by different shapes and colours. Red lines connecting two proteins indicate inhibition, while blue lines show activation. The green circular shade represents the cell membrane, while the orange circular shade demarcates the nuclear envelope. The image was modified from Metacore version 6.22 build 67265 and Bertrand and Dahmane (2006).

The EBRs leading to budgerigar after the divergence from the ancestor of Passeriformes/parrots tended to reshuffle genes involved in *forebrain development*. Remarkably, the same term was also enriched in avian and archousaurian msHSBs, however, the gene pathways affected by EBRs and msHSBs were different (figs. 3 and 4). The msHSBs contained genes related to three of the five conserved canonical signaling pathways involved in forebrain development in vertebrates (Bertrand and Dahmane 2006; Rhinn et al. 2006): the Hedgehog pathway (*SHH*, *Gli2*, and *Gli3*), the WNT pathway (*WNT3A*, *beta-catenin*, and *Lef-1*) and the FGF pathway (*FGF8* and *SOX2*) (Quinlan et al. 2009; Harrison-Uy and Pleasure 2012) (fig. 4). Several studies demonstrated that *WNT3A* is expressed in mouse dorsal telencephalon, but not in chicken (Hollyday et al. 1995), possibly explaining the anatomical differences between the forebrain in these species (Shimogori et al. 2004; Robertshaw and Kiecker 2012). In contrast, the budgerigar lineage-specific EBRs contained genes related to the *NOTCH1*-*NUMB* pathway (fig. 4) as well as *DRAXIN*. All three genes are involved in differentiation of neurones (Wakamatsu et al. 1999; Islam et al. 2009). Although all vocal-learner bird species (songbirds, parrots, and hummingbirds) have “vocal brain nuclei” in the forebrain, parrots, in addition, have an extra shell song-system compared to other vocal-learners (Jarvis 2004; Chakraborty et al. 2015). To the best of our knowledge, this is the first report of distinct components of the same developmental network being found in the evolutionary stable and dynamic parts of animal genomes.

In summary, we demonstrated that genome synteny comparison represents a powerful tool to detect ancestral and lineage-specific genome rearrangements, as well as evolutionary stable chromosomal intervals. Consistent with previous studies in mammals (Murphy et al. 2005; Larkin et al. 2009), chromosome breakage in reptiles and birds is not random but associated with genomic features including TEs and CNEs. We identified functional categories of genes enriched in conserved regions maintained from ancestral chromosomes or in some lineage-specific EBRs with genes related to ancestral- or lineage-specific biology. The most interesting result of EBR contribution to avian evolution (budgerigar) in our set was associated with the highest quality genome supported by additional mapping information. Therefore, the availability of more genomes supported by maps or assembled to a chromosome level will allow us to identify further genomic changes that contributed to the formation of existing species and clades.

## Methods

### Identification of SFs

Alignments of 20 bird genomes and 5 outgroup genomes were performed against chicken genome using SatsumaSynteny (Grabherr et al. 2010) (supplementary table S1, Supplementary Material online). SFs were defined using three sets of parameters to detect genome rearrangements that are ≥500, ≥300, and ≥100 kb in the chicken genome with SyntenyTracker (Donthu et al. 2009).

### Identification and Classification of EBRs

Breakpoint regions (BRs) were defined as the intervals delimited by two adjacent SF boundaries on the same reference chromosome. We developed a new multi-step approach to detect and classify EBRs from chromosome-level and fragmented assemblies. Briefly, we identified all potential BRs for every target genome pairwise comparison with the reference at each resolution in the reference genome coordinates. Then BRs from all pair-wise genome comparisons were cross-compared for reference genome coordinate overlaps. If a target genome was not assembled to chromosomal level, only BRs found within the scaffolds of the target assembly were classified as EBRs. We performed a phylogenetic classification of BRs using an ad hoc likelihood ratio approach, by calculating likelihoods for all possible classifications for each BR. The ratios of likelihoods were calculated for the first and second most likely classifications and were used as a quantitative basis for assigning BRs to phylogenetic branches, thereby qualifying them as EBRs, and distinguishing EBRs from so called *uncertain* BRs that could not be unambiguously assigned to a specific phylogenetic branch (see supplementary data, Supplementary Material online for more details).

To test the accuracy of our EBR classification approach we: 1) compared the EBRs detected by our algorithm in the cattle genome to the previously published manually-defined cattle EBRs (supplementary table S2, Supplementary Material online) and 2) simulated a set of rearranged genomes with predefined phylogeny of EBRs (supplementary fig. S2, Supplementary Material online). We compared these EBRs and their classification to the EBRs detected and classified by our algorithm from the same set of genomes (supplementary tables S3 and S4, Supplementary Material online). Since many of the assemblies used in this study were sequenced and assembled at scaffold level using NGS technologies, we developed a methodology to distinguish between putative assembly errors and lineage-specific EBR in NGS assemblies. First, we tested the EBR intervals by PCR using primers from the EBR-flanking DNA regions for three genomes with different sequencing coverage (63×, 85×, and 105×). We calculated a minimum paired-read spanning coverage from the read libraries in all potential EBR intervals in the same genomes and correlated the levels of coverage to the rates of positive and negative PCR results to estimate the paired-read spanning level for each sequencing coverage that resulted in the minimum number of false positive and false negative EBRs (supplementary tables S7 and S8, Supplementary Material online). We applied these thresholds to other genomes with similar sequencing coverage (supplementary table S8, Supplementary Material online).

To avoid possible underestimation of EBR numbers that would lead to detection of false regions of multispecies synteny we chose the highest (100 kb) resolution to define msHSBs. The 500 kb set was selected for gene enrichment analysis in EBRs to further minimize the effects of potential assembly errors in EBRs.

### Identification of msHSBs

msHSBs were defined as the regions of reference chromosomes with no EBRs or *uncertain* BRs detected in our set of species. Five sets of msHSBs were defined: 1) avian msHSBs, including all birds, 2) archosaurian msHSBs, including birds and crocodiles, 3) archosaurian/testudines msHSBs, in birds, crocodiles, and turtles, 4) sauropsida msHSBs, including all reptiles, and 5) amniote msHSBs, identified in all species studied. The distribution of msHSB sizes was tested for goodness-of-fit to an exponential distribution using the Kolmogorov–Smirnov test following previous publications (Pevzner and Tesler 2003; Larkin et al. 2009) (supplementary tables S9 and S10, Supplementary Material online).

### Functional Analysis of Genes in EBRs and msHSBs

Coordinates of all genes with a single known orthologue in the chicken and human genomes were downloaded from Ensembl (v.74). We focused on this set of genes because the follow-up analyses used functional annotation of genes generated mostly for mammalian genomes. To avoid genes that could be located in mis-assembled parts of both genomes or have erroneous definitions of orthology in Ensembl, we used the gene list to build chicken–human pairwise HSBs with SyntenyTracker using the gene coordinates. This allowed the detection of “singleton” and “out-of-place” genes located in unexpected positions within or between HSBs. These genes were removed from further analyses. We assigned the genes to EBRs or msHSBs following the previously published procedures (Larkin et al. 2009). For the identification of GO terms overrepresented in msHSBs, we considered msHSBs >1.5 Mbp in the chicken genome to avoid genes that could be located in proximity to EBRs. To evaluate gene functional enrichment in EBRs, we considered genes that were located within or ±300 kb from EBR boundaries. We used the Database for Annotation, Visualization and Integrated Discovery (DAVID) (Huang et al. 2008) to detect overrepresented GO terms in our datasets. We considered as significantly enriched terms with >2-fold-enrichment and FDR <10% in EBRs or msHSBs relative to all other regions on chicken chromosomes.

### Comparing Densities of TEs in EBRs and Other Parts of Bird Genomes

Lineage-specific EBRs identified in chicken genome coordinates were translated into the coordinates of target bird genomes using the correspondence between SF boundary coordinates in the chicken and target genomes. In the resulting EBR sets and chicken-specific EBRs we calculated the densities of TEs from major families and compared to those in other intervals of each target genome (RepeatMasker, RepBase v.18), as previously described (Elsik et al. 2009; Larkin et al. 2009; Groenen et al. 2012).

### Density of Bird-Specific CNEs and Genes in msHSBs

Bird-specific conserved elements (Zhang et al. 2014) defined in galGal3 coordinates were filtered to remove elements present in coding parts of chicken genes and all mRNA sequences mapped to the chicken genome, leaving only putative CNEs. Then, we used LiftOver (Kent et al. 2003) to translate the CNE coordinates to galGal4 assembly to make the data compatible with our HSBs sets. We repeated filtering steps for the new genome coordinates obtained. The set of elements that was not overlapping with coding sequences after two filtering steps represented the bird CNEs in the chicken genome. Densities of CNEs and chicken genes (UCSC; all known gene set) were calculated in all msHSBs sets, and were compared to the rest of the reference genome using the previously published pipeline (Larkin et al. 2009). After the GO enrichment analysis was performed, we screened the avian-specific CNEs nearby genes in the enriched categories for TFBSs using PROMO (Messeguer et al. 2002) with a dissimilarity margin ≤10% with TFBSs found in chicken.

## Supplementary Material

Supplementary figures S1–S7 and tables S1–S13 are available at *Genome Biology and Evolution* online (http://www.gbe.oxfordjournals.org/).

Supplementary Data
