# Supplementary material for: Novel Insights into Chromosome Evolution in Birds, Archosaurs, and Reptiles
Source: Genome Biol Evol. 2016 Jul 10;8(8):2442–51. doi: 10.1093/gbe/evw166 (PMC5010900; doi:10.1093/gbe/evw166)
Supplement: Supplementary Data [file supp_8_8_2442__index.html]

Novel Insights into Chromosome Evolution in Birds, Archosaurs, and Reptiles — Supplementary Data 

# Novel Insights into Chromosome Evolution in Birds, Archosaurs, and Reptiles

## Supplementary Data

files

- Supplementary Data - pdf file
- Supplementary Data - xls file
- Supplementary Data - xls file
- Supplementary Data - pdf file
- Supplementary Data - pdf file
