# Supplementary material for: Novel Insights into Chromosome Evolution in Birds, Archosaurs, and Reptiles
Source: Genome Biol Evol. 2016 Jul 10;8(8):2442–51. doi: 10.1093/gbe/evw166 (PMC5010900; doi:10.1093/gbe/evw166)
Supplement: Supplementary Data [file supp_evw166_SupplementaryTable11_msHSBs.pdf]

Supplementary table 11a. Multispecies homologous synteny blocks (msHSBs) detected in birds

Chr	msHSB start (bp)	msHSB end (bp)	msHSB length (bp)	Probability of being found under the Poisson process	Gene ID	Chr	Gene start (bp)	Gene end (bp)
4	57,866,704	59,398,610	1,531,906	1				
					BMPR1B	4	57,960,584	58,196,338
					UNC5C	4	58,208,189	58,447,378
					RAP1GDS1	4	59,298,214	59,387,366
2	56,148,511	57,688,217	1,539,706	1				
					ADNP2	2	56,151,314	56,169,460
					C18ORF22	2	56,186,903	56,202,681
					TXNL4A	2	56,260,575	56,271,815
					HSBP1L1	2	56,276,632	56,279,702
					PQLC1	2	56,286,727	56,359,738
					KCNG2	2	56,365,653	56,391,148
					CTDP1	2	56,473,381	56,565,099
					NFATC1	2	56,697,424	56,780,391
					ATP9B	2	56,802,202	56,969,787
					SALL3	2	57,012,984	57,032,376
1	53,301,957	54,851,094	1,549,137	1				
					PWP1	1	53,306,966	53,321,328
					BTBD11	1	53,339,867	53,411,487
					CRY1	1	53,554,188	53,588,375
					C1H12ORF23	1	53,597,859	53,610,656
					RIC8B	1	53,634,893	53,669,600
					RFX4	1	53,674,650	53,748,422
					POLR3B	1	53,773,941	53,842,276
					TCP11L2	1	53,843,800	53,857,446
					CKAP4	1	53,861,783	53,873,170
					NUAK1	1	53,895,864	53,940,812
					OCC-1	1	54,142,067	54,158,684
					C12orf45	1	54,283,953	54,287,411
					CHST11	1	54,543,813	54,741,556
					TXNRD1	1	54,742,740	54,768,918
					NFYB	1	54,792,307	54,806,820
					HCFC2	1	54,813,504	54,834,785
					MTERFD3	1	53,593,807	53,595,000
2	8,014,363	9,565,314	1,550,951	1				
					SHH	2	8,080,091	8,089,855
					RNF32	2	8,414,446	8,427,357
					LMBR1	2	8,434,744	8,494,298
					NOM1	2	8,513,775	8,529,642
					MNX1	2	8,538,868	8,541,508
					UBE3C	2	8,582,314	8,644,842
					PTPRN2	2	8,785,742	9,406,733
					NCAPG2	2	9,417,857	9,456,238
					ESYT2	2	9,467,167	9,532,244
1	145,045,056	146,620,275	1,575,219	1				
					UGGT2	1	145,103,089	145,180,354
					DNAJC3	1	145,184,466	145,216,297
					DZIP1	1	145,229,679	145,262,325
					CLDN10	1	145,266,284	145,279,564
					ABCC4	1	145,358,360	145,506,681
					GPR180	1	145,671,022	145,692,774
					TGDS	1	145,692,790	145,705,488
					DCT	1	145,716,342	145,737,439
1	97,365,488	98,957,585	1,592,097	1				
					HSPA13	1	97,411,085	97,418,694
					SAMSN1	1	97,445,928	97,480,511
					USP25	1	97,936,529	98,030,131
					CXADR	1	98,638,187	98,668,892
					C1H21ORF91	1	98,700,854	98,720,026
					CHODL	1	98,864,912	98,893,667

					TMPRSS15	1	98,898,052	98,906,375
					NRIP1	1	97,651,374	97,654,841
1	112,922,900	114,521,910	1,599,010	1				
					TMEM47	1	113,256,480	113,283,351
4	71,953,595	73,555,681	1,602,086	1				
					STIM2	4	72,683,440	72,741,445
					TBC1D19	4	72,753,287	72,801,383
					CCK1R	4	72,818,063	72,824,919
					RBPJ	4	72,840,334	72,980,021
					C4ORF52	4	72,987,542	72,991,389
					SEL1L3	4	72,995,781	73,024,332
					SLC34A2	4	73,038,472	73,055,616
					ANAPC4	4	73,093,795	73,110,519
					ZCCHC4	4	73,110,600	73,121,105
					PI4K2B	4	73,122,273	73,139,009
					SEPSECS	4	73,142,031	73,165,468
					LGI2	4	73,182,369	73,204,135
					CCDC149	4	73,225,282	73,274,932
					SOD3	4	73,280,251	73,281,120
					DHX15	4	73,318,070	73,361,220
3	5,925,176	7,559,819	1,634,643	1				
					NRXN1	3	6,450,555	7,094,776
					FSHR	3	7,340,937	7,418,777
					LHCGR	3	7,505,304	7,524,818
Z	11,130,428	12,770,793	1,640,365	1				
3	24,475,771	26,122,614	1,646,843	1				
					PLEKHH2	3	24,491,110	24,545,482
					DYNC2LI1	3	24,548,141	24,570,676
					ABCG5	3	24,567,546	24,588,926
					ABCG8	3	24,585,792	24,598,091
					LRPPRC	3	24,608,804	24,695,385
					PPM1B	3	24,761,763	24,820,317
					SLC3A1	3	24,827,681	24,841,984
					PREPL	3	24,831,731	24,859,714
					SIX2	3	25,240,527	25,243,491
					SRBD1	3	25,432,172	25,549,520
					PRKCE	3	25,576,043	25,856,010
					EPAS1	3	25,951,104	25,982,708
					RHOQ	3	26,075,656	26,091,343
					PIGF	3	26,084,410	26,110,103
					CRIPT	3	26,110,349	26,115,765
7	19,720,582	21,369,588	1,649,006	1				
					KCNH7	7	20,330,959	20,538,685
					IFIH1	7	20,543,411	20,570,333
					FAP	7	20,575,083	20,613,175
					GCG	7	20,616,148	20,633,934
					DPP4	7	20,637,422	20,676,092
					SLC4A10	7	20,678,066	20,774,184
					TBR1	7	20,868,507	20,872,724
					PSMD14	7	20,876,177	20,921,590
					TANK	7	20,950,028	20,975,797
					RBMS1	7	21,176,600	21,260,777
					ITGB6	7	21,293,508	21,321,944
					PLA2R1	7	21,327,981	21,363,749
					FIGN	7	20,078,215	20,080,458
2	114,752,755	116,406,395	1,653,640	1				
					ADHFE1	2	114,787,387	114,799,160
					C8orf46	2	114,810,823	114,828,586
					MYBL1	2	114,842,426	114,865,431
					VCPIP1	2	114,881,199	114,894,526
					MCMDC2	2	114,970,567	114,979,273
					PPP1R42	2	114,999,640	115,011,937
					COPS5	2	115,019,073	115,030,460
					CSPP1	2	115,030,393	115,080,545

						CPA6	2	115,185,937	115,273,389
						PREX2	2	115,324,553	115,492,355
						C8orf34	2	115,526,242	115,690,070
						SULF1	2	115,993,905	116,060,835
						SLCO5A1	2	116,069,828	116,132,391
						PRDM14	2	116,194,099	116,199,530
						NCOA2	2	116,210,250	116,389,448
	2	105,905,923	107,559,591	1,653,668	1				
						TRAPPC8	2	105,967,982	106,017,814
						RNF138	2	106,040,321	106,047,320
						GAREM	2	106,104,445	106,201,400
						KLHL14	2	106,272,905	106,330,763
						CCDC178	2	106,537,940	106,579,883
						ASXL3	2	106,579,834	106,707,763
							2	106,756,439	106,849,498
						DTNA	2	107,131,493	107,228,834
						MAPRE2	2	107,310,933	107,345,428
	4	30,977,991	32,641,548	1,663,557	1				
						TTC29	4	31,030,502	31,218,350
						EDNRA	4	31,349,185	31,378,701
						TMEM184C	4	31,400,287	31,410,845
						PRMT10	4	31,412,158	31,427,252
						ARHGAP10	4	31,431,945	31,559,864
						NR3C2	4	31,566,745	31,763,750
						DCLK2	4	32,207,746	32,286,859
						MAB21L2	4	32,453,927	32,455,006
	2	23,180,896	24,845,823	1,664,927	1				
						CALCR	2	23,188,382	23,325,400
						TFPI2	2	23,431,045	23,434,937
						BET1	2	23,524,126	23,530,040
						COL1A2	2	23,702,024	23,740,814
						GGA.31495	2	23,751,418	23,775,319
						SGCE	2	23,779,732	23,806,287
						PPP1R9A	2	23,846,203	23,968,530
						ASB4	2	24,010,593	24,020,518
						PDK4	2	24,033,200	24,043,554
						DYNC1I1	2	24,085,822	24,251,013
						SLC25A13	2	24,256,193	24,345,252
						SHFM1	2	24,450,646	24,457,181
						DLX6	2	24,572,105	24,577,130
						DLX5	2	24,580,154	24,582,822
						ACN9	2	24,607,642	24,634,662
						TAC1	2	24,773,218	24,779,911
						ASNS	2	24,791,550	24,805,274
						C1GALT1	2	24,823,339	24,831,591
	5	33,186,512	34,867,480	1,680,968	1				
						KIAA1333	5	33,413,582	33,433,671
						SCFD1	5	33,438,355	33,483,021
						COCH	5	33,517,885	33,534,657
						AP4S1	5	33,603,694	33,613,992
						HECTD1	5	33,616,118	33,666,611
						HEATR5A	5	33,685,490	33,736,833
						NUBPL	5	33,774,090	33,856,331
						ARHGAP5	5	33,958,351	33,993,431
						AKAP6	5	34,050,021	34,304,671
z		47,444,682	49,154,115	1,709,433	1				
	1	80,304,616	82,024,763	1,720,147	1				
						LSAMP	1	81,373,015	81,656,000
						GAP43	1	81,680,485	81,734,254
	17	2,877,535	4,606,475	1,728,940	1				
						PAPPA	17	3,040,970	3,209,266
						ASTN2	17	3,219,825	3,482,002
						TLR4	17	3,566,454	3,571,907
						DBC1	17	3,985,882	4,064,290

					CDK5RAP2	17	4,495,102	4,566,562
					MEGF9	17	4,575,673	4,590,722
2	123,315,136	125,044,596	1,729,460	1				
					MMP16	2	123,445,894	123,621,303
					RIPK2	2	124,047,743	124,066,705
					OSGIN2	2	124,129,057	124,139,546
					NBN	2	124,149,190	124,170,636
					DECR1	2	124,172,883	124,182,569
					CALB1	2	124,187,670	124,207,058
					TMEM64	2	124,388,961	124,400,813
					NECAB1	2	124,431,977	124,487,257
						2	124,491,416	124,499,565
					TMEM55A	2	124,512,882	124,538,359
					SLC26A7	2	124,587,622	124,658,017
					RUNX1T1	2	124,823,184	124,932,746
10	17,205,849	18,945,200	1,739,351	1				
					SPG21	10	17,214,064	17,223,515
					CLPX	10	17,224,372	17,244,650
					PDCD7	10	17,245,077	17,249,216
					UBAP1L	10	17,252,331	17,258,965
					RASL12	10	17,265,410	17,269,553
					MTFMT	10	17,280,250	17,285,028
					CILP	10	17,290,315	17,300,075
					PARP16	10	17,314,082	17,318,997
					IGDCC3	10	17,337,460	17,420,513
					IGDCC4	10	17,424,605	17,505,535
					DPP8	10	17,518,747	17,539,501
					PTPLAD1	10	17,541,428	17,549,930
					VWA9	10	17,550,456	17,561,266
					SLC24A1	10	17,564,942	17,579,906
					DENND4A	10	17,584,576	17,628,407
					RAB11A	10	17,646,169	17,662,291
					MEGF11	10	17,666,849	17,858,077
					DIS3L	10	17,937,780	17,953,572
					TIPIN	10	17,954,714	17,962,115
					MAP2K1	10	17,965,094	17,995,641
					SNAPC5	10	17,997,064	17,999,465
					RPL4	10	18,000,215	18,005,535
					ZWILCH	10	18,005,980	18,015,614
					SMAD6	10	18,171,488	18,201,886
					SMAD3	10	18,282,943	18,345,447
					AAGAB	10	18,351,819	18,367,839
					IQCH	10	18,368,140	18,426,597
					MAP2K5	10	18,430,852	18,432,733
					MAP2K5	10	18,440,729	18,553,284
					PIAS1	10	18,595,984	18,624,673
					CALML4	10	18,629,343	18,632,005
					CLN6	10	18,632,517	18,638,897
					FEM1B	10	18,641,302	18,648,029
					ITGA11	10	18,646,162	18,665,906
					CORO2B	10	18,699,925	18,729,841
					NOX5	10	18,770,495	18,779,187
					GLCE	10	18,807,760	18,817,356
					PAQR5	10	18,823,404	18,826,891
					KIF23	10	18,828,578	18,846,365
					KBTBD13	10	17,262,311	17,263,693
5	10,087,655	11,834,285	1,746,630	1				
					SOX6	5	10,549,851	10,784,968
					C5H11ORF58	5	10,948,695	10,953,363
					PLEKHA7	5	10,959,448	11,057,508
					RPS13	5	11,136,190	11,139,937
					PIK3C2A	5	11,142,129	11,205,960
					NUCB2	5	11,205,875	11,231,516
					KCNJ11	5	11,247,848	11,250,065

					ABCC8	5	11,253,945	11,319,342
					USH1C	5	11,334,156	11,377,576
					OTOG	5	11,381,876	11,476,414
					MYOD1	5	11,578,793	11,582,278
					KCNC1	5	11,597,391	11,714,071
4	41,889,738	43,640,988	1,751,250	1				
					GALNTL6	4	42,203,702	42,635,333
					GALNT7	4	42,697,173	42,736,160
					HMGB2	4	42,740,630	42,743,052
					SAP30	4	42,754,695	42,760,373
					SCRG1	4	42,763,397	42,769,576
					FBXO8	4	43,131,548	43,148,365
					CEP44	4	43,149,107	43,165,568
					HPGD	4	43,211,798	43,236,772
					GLRA3	4	43,301,385	43,363,404
3	65,651,343	67,404,826	1,753,483	1				
					RPF2	3	65,709,193	65,721,929
					GTF3C6	3	65,721,376	65,726,791
					AMD1	3	65,744,041	65,761,269
					CDK19	3	65,801,524	65,899,585
					SLC22A16	3	65,931,799	65,962,885
					DDO	3	65,969,395	65,975,774
					C6ORF186	3	65,985,390	66,029,579
					CDC40	3	66,033,229	66,074,352
					WASF1	3	66,123,600	66,154,746
					GPR6	3	66,173,387	66,197,130
					FIG4	3	66,201,896	66,268,774
					AK9	3	66,269,578	66,325,155
					ZBTB24	3	66,327,020	66,334,932
					PPIL6	3	66,340,530	66,350,895
					CD164	3	66,357,144	66,365,037
					C6ORF183	3	66,413,334	66,432,788
					CEP57L1	3	66,460,394	66,473,222
					SESN1	3	66,547,545	66,556,746
					ARMC2	3	66,560,205	66,617,959
					FOXO3	3	66,702,483	66,716,393
					LACE1	3	66,809,734	66,870,599
					SNX3	3	66,876,089	66,895,012
					NR2E1	3	66,898,978	66,913,949
					OSTM1	3	66,950,856	66,961,817
					SEC63	3	66,978,001	67,033,488
					SCML4	3	67,042,180	67,098,929
					SOBP	3	67,134,086	67,243,457
					PDSS2	3	67,259,779	67,378,577
					BEND3	3	67,393,979	67,403,976
3	99,384,303	101,145,816	1,761,513	1				
					VSNL1	3	99,739,837	99,814,889
					SMC6	3	99,815,077	99,852,120
					GEN1	3	99,853,931	99,870,493
					KCNS3	3	99,922,984	99,942,855
					RDH14	3	100,213,340	100,217,996
					OSR1	3	100,695,798	100,697,672
					TTC32	3	101,036,992	101,038,388
					WDR35	3	101,039,084	101,075,670
					MATN3	3	101,078,563	101,093,823
					LAPTM4A	3	101,097,118	101,110,445
					MSGN1	3	99,887,826	99,889,127
8	23,780,361	25,569,631	1,789,270	1				
					GLIS1	8	23,804,849	23,900,621
					NDC1	8	23,986,245	24,001,606
					YIPF1	8	24,001,645	24,006,175
					DIO1	8	24,006,797	24,011,727
					LRRC42	8	24,015,720	24,020,780
					LDLRAD1	8	24,023,108	24,025,375

					TMEM59	8	24,030,293	24,038,522
					TCEANC2	8	24,038,683	24,041,647
					CDCP2	8	24,045,805	24,049,407
					MRPL37	8	24,064,757	24,068,753
					SSBP3	8	24,074,037	24,124,115
					ACOT11	8	24,131,387	24,140,486
					TTC4	8	24,142,957	24,148,277
					C1ORF177	8	24,152,396	24,156,229
					DHCR24	8	24,157,606	24,165,747
					TMEM61	8	24,173,996	24,176,346
					BSND	8	24,177,461	24,179,735
					PCSK9	8	24,180,817	24,184,784
					USP24	8	24,190,573	24,237,937
					PPAP2B	8	24,600,649	24,643,425
					PRKAA2	8	24,663,005	24,679,845
					C1ORF168	8	24,687,128	24,702,955
					C8A	8	24,711,328	24,727,316
					C8B	8	24,731,782	24,747,335
					DAB1	8	24,753,891	24,842,483
					OMA1	8	25,201,610	25,219,335
					MYSM1	8	25,232,688	25,245,087
					FGGY	8	25,416,352	25,524,129
					HOOK1	8	25,544,038	25,566,897
					PARS2	8	24,148,653	24,149,966
					TACSTD2	8	25,225,850	25,226,746
					JUN	8	25,267,213	25,268,157
1	109,809,272	111,600,312	1,791,040	1				
					CXorf36	1	110,128,634	110,143,679
					FUNDC1	1	110,347,436	110,362,439
					EFHC2	1	110,382,985	110,442,596
					NDP	1	110,522,886	110,531,383
					MAOB	1	110,555,374	110,609,289
					MAOA	1	110,613,430	110,658,911
					GPR34	1	111,232,706	111,235,607
					NYX	1	111,334,091	111,336,368
					GPR82	1	111,219,959	111,220,993
3	35,427,107	37,224,095	1,796,988	1				
					GREM2	3	35,493,725	35,533,009
					FMN2	3	35,540,378	35,680,173
					CHRM3	3	35,727,538	35,877,494
					RYR2	3	36,498,791	36,661,068
					MTR	3	36,890,573	36,933,337
					ACTN2	3	36,948,211	37,014,188
					HEATR1	3	37,025,129	37,060,227
					LGALS8	3	37,061,029	37,073,707
					EDARADD	3	37,078,799	37,097,401
					ERO1LB	3	37,097,636	37,134,158
					GPR137B	3	37,135,755	37,159,463
					NID1	3	37,178,196	37,213,970
4	36,719,773	38,524,904	1,805,131	1				
					COL25A1	4	36,799,079	37,092,841
					ETNPPL	4	37,104,651	37,118,540
					LEF-1	4	37,323,508	37,377,729
					HADH	4	37,389,440	37,406,348
					CYP2U1	4	37,409,174	37,420,402
					SGMS2	4	37,429,511	37,455,185
					PAPSS1	4	37,490,651	37,526,579
					DKK2	4	37,660,752	37,705,773
					AIMP1	4	37,847,835	37,861,897
					TBCK	4	37,878,922	37,971,616
					NPNT	4	37,998,529	38,044,094
					GSTCD	4	38,057,434	38,119,998
					INTS12	4	38,124,523	38,140,681
					ARHGEF38	4	38,146,410	38,172,500

					PPA2	4	38,190,034	38,225,681
					TET2	4	38,256,307	38,277,565
1	10,916,110	12,726,034	1,809,924	1				
					CD36	1	11,005,425	11,038,504
					GNAT3	1	11,086,584	11,113,407
					GNAI3	1	11,190,979	11,223,596
					MAGI2	1	11,930,694	12,230,824
					PHTF2	1	12,251,576	12,314,104
					TMEM60	1	12,314,478	12,318,704
					RSBN1L	1	12,327,050	12,358,827
					PTPN18	1	12,381,899	12,422,097
					GSAP	1	12,486,935	12,533,438
					FGL2	1	12,586,975	12,594,183
					FAM185A	1	12,607,530	12,647,356
					P37NB	1	12,679,724	12,694,908
4	20,777,938	22,596,657	1,818,719	1				
					GLRB	4	20,823,798	20,857,554
					GRIA2	4	20,873,025	20,960,836
					FAM198B	4	21,221,107	21,234,597
					TMEM144	4	21,242,338	21,257,193
					RXFP1	4	21,328,064	21,376,386
					ETFDH	4	21,379,598	21,396,998
					PPID	4	21,395,223	21,405,790
					FNIP2	4	21,425,883	21,457,823
					C4ORF45	4	21,455,357	21,496,063
					RAPGEF2	4	21,509,902	21,683,609
					FSTL5	4	22,250,827	22,516,765
2	1,476,281	3,303,402	1,827,121	1				
					VIPR1	2	1,722,123	1,826,646
					SEC22C	2	1,886,499	1,900,914
					NKTR	2	1,927,422	1,965,795
					ZBTB47	2	1,978,851	1,993,362
					KBTBD5	2	1,999,758	2,009,597
					HHATL	2	2,014,408	2,026,554
					CCDC13	2	2,032,322	2,059,811
					HIGD1C	2	2,061,655	2,066,620
					CCBP2	2	2,072,000	2,073,926
					OBSCN	2	2,080,439	2,253,956
					C2H1ORF69	2	2,295,676	2,300,126
					GJC2	2	2,303,738	2,310,415
					GUK1	2	2,334,607	2,343,799
					MRPL55	2	2,348,731	2,351,041
					C1ORF35	2	2,351,366	2,358,790
					ARF1	2	2,357,324	2,369,250
					WNT3A	2	2,459,534	2,542,486
					WNT9A	2	2,573,612	2,624,503
					SNAP47	2	3,185,315	3,205,912
					JMJD4	2	3,211,699	3,220,712
					ALS2CL	2	3,216,850	3,246,773
					TMIE	2	3,268,001	3,297,394
4	23,185,693	25,022,794	1,837,101	1				
					TMEM192	4	23,236,487	23,257,108
					KLHL2	4	23,265,508	23,325,234
					SC4MOL	4	23,326,894	23,333,143
					CPE	4	23,336,496	23,382,106
					TLL1	4	23,520,463	23,656,666
					SPOCK3	4	23,947,374	24,051,813
					ANXA10	4	24,400,386	24,416,023
					PALLD	4	24,486,948	24,675,736
					AADAT	4	24,691,532	24,707,952
					MFAP3L	4	24,743,867	24,747,796
					C4ORF27	4	24,796,835	24,803,472
					CLCN3	4	24,804,714	24,864,749
					NEK1	4	24,866,404	24,909,512

						CBR4	4	25,012,882	25,021,145
	1	189,931,149	191,772,697	1,841,548	1				
						TENM4	1	191,230,962	191,733,590
	4	86,080,977	87,947,885	1,866,908	1				
						ADRA1D	4	87,863,056	87,903,531
						LRRTM1	4	87,094,015	87,096,026
	1	26,819,307	28,694,711	1,875,404	1				
						C7ORF53	1	26,846,392	26,853,187
						IFRD1	1	26,854,961	26,865,547
						ZNF277	1	26,905,761	26,960,018
						DNAJB9	1	28,382,860	28,392,521
						THAP5	1	28,392,695	28,399,112
						PNPLA8	1	28,473,454	28,508,702
						NRCAM	1	28,525,499	28,668,231
						LRRN3	1	27,413,542	27,415,668
	2	18,319,715	20,197,363	1,877,648	1				
						PLXDC2	2	18,410,966	18,641,995
						C10orf112	2	18,793,459	18,829,474
						ARL5B	2	18,960,948	18,977,002
						CACNB2	2	19,003,860	19,074,365
						SLC39A12	2	19,249,639	19,287,303
						STAM	2	19,520,144	19,541,550
						PTPLA	2	19,567,820	19,575,886
						ST8SIA6	2	19,614,633	19,653,024
						VIM	2	19,677,910	19,684,966
						CUBN	2	19,737,380	19,876,287
						RSU1	2	19,881,822	19,979,217
						PTER	2	20,004,930	20,020,105
	8	25,607,217	27,516,548	1,909,331	1				
						CNFI-A4	8	25,948,005	26,179,911
						TM2D1	8	26,200,325	26,219,046
						INADL	8	26,226,028	26,357,392
						KANK4	8	26,365,577	26,378,312
						USP1	8	26,391,541	26,401,494
						ANGPTL3	8	26,448,477	26,457,422
						ATG4C	8	26,501,092	26,520,896
						ALG6	8	26,691,601	26,712,046
						ITGB3BP	8	26,713,799	26,739,349
						EFCAB7	8	26,748,611	26,762,766
						PGM1	8	26,764,668	26,783,622
						ROR1	8	26,884,366	26,926,589
						CACHD1	8	26,977,492	27,061,474
						RAVER2	8	27,069,633	27,101,820
						AK3L2	8	27,167,597	27,176,227
						DNAJC6	8	27,186,950	27,217,638
						LEPROT	8	27,220,963	27,226,804
						LEPR	8	27,238,562	27,268,762
						PDE4B	8	27,415,316	27,426,443
						SGIP1	8	27,431,896	27,462,447
						TCTEX1D1	8	27,465,606	27,467,851
						WDR78	8	27,471,548	27,487,186
						MIER1	8	27,487,798	27,516,244
	3	81,161,816	83,102,150	1,940,334	0.999999999				
						RIMS1	3	81,337,238	81,638,081
						OGFRL1	3	81,808,877	81,818,348
						B3GAT2	3	81,940,693	81,962,704
						FAM135A	3	82,085,759	82,150,209
						COL9A1	3	82,230,623	82,296,804
						COL19A1	3	82,304,939	82,484,565
						LMBRD1	3	82,508,371	82,577,230
z		27,522,420	29,468,950	1,946,530	0.999999999				
	1	141,339,838	143,294,319	1,954,481	0.999999998				
						SLC10A2	1	141,914,133	141,925,460
						BIVM	1	142,024,553	142,041,190

					KDELC1	1	142,045,388	142,054,410
					C13ORF27	1	142,057,711	142,064,431
					METTL21C	1	142,092,496	142,099,273
					TPP2	1	142,097,596	142,147,887
					ITGBL1	1	142,614,261	142,752,083
					NALCN	1	142,765,240	142,969,863
					TMTC4	1	143,142,401	143,197,059
					A2LD1	1	143,230,157	143,230,654
4	73,572,127	75,554,297	1,982,170	0.999999995				
					PPARGC1A	4	73,626,292	73,691,855
					GPR125	4	74,015,279	74,072,320
					KCNIP4	4	74,436,584	74,527,405
					PACRGL	4	74,526,045	74,539,587
					SLIT2	4	74,557,149	74,802,699
					LCORL	4	75,401,346	75,452,145
					NCAPG	4	75,480,300	75,503,309
					FAM184B	4	75,524,898	75,546,434
					MED28	4	75,551,241	75,553,275
10	13,555,367	15,569,180	2,013,813	0.99999998				
					SV2B	10	13,701,912	13,728,770
					SLCO3A1	10	13,838,315	13,953,221
					ST8SIA2	10	13,994,580	14,019,205
					FAM174B	10	14,039,364	14,055,885
					CHD2	10	14,112,774	14,149,142
					RGMA	10	14,166,477	14,183,190
					MCTP2	10	14,461,079	14,573,856
					NR2F2	10	15,328,163	15,333,861
3	58,723,180	60,739,930	2,016,750	0.99999998				
					RSPO3	3	58,726,123	58,785,427
					CENPW	3	59,060,255	59,067,195
					TRMT11	3	59,178,746	59,205,181
					HINT3	3	59,212,941	59,219,743
					NCOA7	3	59,223,034	59,291,522
					HDDC2	3	59,516,400	59,525,907
					TPD52L1	3	59,527,859	59,580,356
					RNF217	3	59,606,826	59,662,599
					NKAIN2	3	59,710,415	59,999,592
					TRDN	3	60,315,841	60,432,093
					CLVS2	3	60,548,727	60,598,139
					SMPD1	3	60,658,437	60,671,812
					FABP7	3	60,674,294	60,677,648
2	141,251,316	143,287,606	2,036,290	0.999999955				
					TMEM71	2	141,260,434	141,266,499
					PHF20L1	2	141,275,093	141,322,936
					TG	2	141,333,054	141,469,306
					SLA	2	141,410,203	141,432,079
					WISP1	2	141,532,750	141,540,007
					NDRG1	2	141,549,217	141,589,409
					ST3GAL1	2	141,665,331	141,689,879
					ZFAT	2	142,122,786	142,207,755
					KHDRBS3	2	142,700,486	142,776,708
1	12,729,400	14,775,388	2,045,988	0.999999924				
					NAPEPLD	1	12,737,151	12,756,983
					PMPCB	1	12,778,148	12,786,817
					DNAJC2	1	12,786,999	12,804,232
					PSMC2	1	12,804,486	12,811,505
					SLC26A5	1	12,815,215	12,848,571
					RELN	1	12,865,261	13,129,087
					ORC5L	1	13,157,747	13,226,609
					KMT2E	1	13,575,018	13,627,041
					PUS7	1	13,788,331	13,809,208
					RINT1	1	13,810,273	13,822,128
					EFCAB10	1	13,821,811	13,823,862
					ATXN7L1	1	13,831,430	13,898,855

					CDHR3	1	13,960,601	13,995,845
					SYPL1	1	14,001,061	14,009,388
					PIK3CG	1	14,213,411	14,241,547
					PRKAR2B	1	14,328,390	14,346,800
					HBP1	1	14,356,759	14,367,631
					COG5	1	14,369,255	14,553,213
					DUS4L	1	14,553,188	14,563,663
					BCAP29	1	14,567,866	14,591,310
					SLC26A4	1	14,597,269	14,618,545
					SLC26A3	1	14,632,208	14,642,808
					DLD	1	14,666,134	14,679,885
					LAMB1	1	14,681,577	14,715,087
					LAMB4	1	14,725,779	14,766,576
					CCDC71L	1	14,180,471	14,180,770
					GPR22	1	14,518,793	14,520,725
2	112,226,473	114,277,051	2,050,578	0.999999922				
					CA8	2	112,413,504	112,453,419
					RAB2A	2	112,517,303	112,558,232
					CHD7	2	112,612,964	112,699,976
					CLVS1	2	112,882,281	112,978,767
					NKAIN3	2	113,137,211	113,469,039
					GGH	2	113,490,054	113,501,161
					TTPA	2	113,502,155	113,517,713
					YTHDF3	2	113,543,309	113,551,391
					CYP7B1	2	114,093,263	114,114,645
7	17,647,530	19,715,876	2,068,346	0.999999866				
					GORASP2	7	17,668,960	17,675,340
					GAD67	7	17,698,291	17,724,925
					SP5	7	17,752,450	17,753,992
					MYO3B	7	17,770,145	17,966,292
					ZNF650	7	17,977,176	18,060,443
					METTL5	7	18,071,082	18,075,904
					SSB	7	18,074,706	18,082,768
					KLHL23	7	18,084,797	18,088,611
					C2ORF77	7	18,093,729	18,100,030
					PPIG	7	18,115,845	18,140,425
					FASTKD1	7	18,144,867	18,157,494
					LRP2	7	18,254,209	18,342,504
					RDH5	7	18,356,767	18,360,929
					ABCB11	7	18,366,761	18,411,191
					G6PC2	7	18,415,594	18,420,438
					SPC25	7	18,426,550	18,430,278
					LASS6	7	18,442,947	18,552,421
					SCN9A	7	19,008,759	19,043,596
					TTC21B	7	19,154,460	19,185,811
					GALNT3	7	19,189,885	19,211,629
					CSRNP3	7	19,226,361	19,245,814
					SCN2A	7	19,318,427	19,366,026
					SCN3A	7	19,411,316	19,468,431
					SLC38A11	7	19,501,055	19,525,264
					COBLL1	7	19,544,324	19,617,293
					GRB14	7	19,657,871	19,705,480
					PHOSPHO2	7	18,092,617	18,093,339
					B3GALT1	7	18,689,016	18,689,996
1	63,243,561	65,315,080	2,071,519	0.999999811				
					STRAP	1	63,250,603	63,259,735
					DERA	1	63,261,392	63,316,947
					SLC15A5	1	63,366,498	63,395,422
					MGST1	1	63,429,485	63,435,977
					LMO3	1	63,504,395	63,561,066
					RERGL	1	64,044,462	64,052,106
					PIK3C2G	1	64,133,608	64,331,932
					PLCZ1	1	64,333,507	64,376,477
					PLEKHA5	1	64,579,551	64,672,674

					AEBP2	1	64,687,656	64,736,649
					PDE3A	1	65,036,282	65,270,875
					SLCO1C1	1	65,289,165	65,310,400
					CAPZA3	1	64,376,712	64,377,673
5	29,659,107	31,749,250	2,090,143	0.999999706				
					FMN1	5	29,701,247	29,817,292
					GREM1	5	29,842,277	29,849,273
					SCG5	5	29,864,000	29,890,748
					RASGRP1	5	29,940,334	29,974,856
					FAM98B	5	29,981,575	29,990,845
					SPRED1	5	30,010,890	30,068,434
					MEIS2	5	30,506,655	30,675,196
					C5H15orf41	5	30,717,442	30,832,671
					ATPBD4	5	31,253,451	31,438,936
					ZNF770	5	31,468,292	31,472,971
					AQR	5	31,481,161	31,528,089
					ACTC1	5	31,546,121	31,552,902
					GJD2	5	31,568,439	31,572,498
					STXBP6	5	31,693,846	31,748,417
2	125,160,507	127,250,892	2,090,385	0.999999678				
					TRIQK	2	125,273,982	125,333,683
					FAM92A1	2	125,604,624	125,621,196
					RBM12B	2	125,623,603	125,630,097
					TMEM67	2	125,632,568	125,659,384
					PDP1	2	125,683,595	125,690,722
					CDH17	2	125,754,667	125,783,019
					GEM	2	125,802,915	125,811,572
					RAD54B	2	125,844,098	125,903,394
					FSBP	2	125,877,035	125,887,013
					KIAA1429	2	125,925,066	125,952,171
					ESRP1	2	125,956,620	125,988,739
					DPY19L4	2	126,052,840	126,076,741
					INTS8	2	126,126,452	126,151,324
					CCNE2	2	126,151,226	126,161,848
					TP53INP1	2	126,170,346	126,183,360
					NDUFAF6	2	126,201,059	126,216,326
					PLEKHF2	2	126,225,096	126,240,839
					C8orf37	2	126,258,109	126,269,454
					MTERFD1	2	126,597,212	126,615,684
					PTDSS1	2	126,616,011	126,645,632
					SDC2	2	126,740,060	126,929,143
					MTDH	2	127,057,053	127,088,247
					LAPTM4B	2	127,102,614	127,165,676
					MATN2	2	127,183,885	127,242,408
1	129,013,014	131,109,299	2,096,285	0.999999568				
					SHOX	1	129,086,063	129,094,361
					PPP2R3B	1	129,302,922	129,356,735
					GTPBP6	1	129,412,881	129,423,230
					PLCXD1	1	129,423,877	129,442,045
					RGN	1	129,470,051	129,482,680
					PHF16	1	129,491,121	129,535,706
					RP2	1	129,557,913	129,575,998
					SLC9A7	1	129,620,479	129,659,911
					TUBGCP5	1	129,707,209	129,728,936
					CYFIP1	1	129,747,745	129,798,637
					NIPA2	1	129,800,366	129,811,364
					OCA2	1	130,005,140	130,159,850
					GABRG3	1	130,314,510	130,614,689
					GABRA5	1	130,620,080	130,675,538
					GABRB3	1	130,705,242	130,819,496
10	7,538,412	9,634,830	2,096,418	0.999999574				
					UNC13C	10	7,641,038	7,759,221
					WDR72	10	7,807,793	7,874,460
					FAM214A	10	8,193,075	8,224,905

					ARPP19	10	8,227,077	8,235,366
					MYO5A	10	8,240,552	8,332,115
					MYO5C	10	8,334,755	8,365,618
					MAPK6	10	8,418,112	8,446,458
					LEO1	10	8,499,714	8,506,990
					TMOD3	10	8,510,697	8,533,995
					TMOD2	10	8,537,029	8,567,174
					LYSMD2	10	8,581,242	8,590,880
					SCG3	10	8,596,070	8,621,388
					AP4E1	10	8,625,187	8,643,899
					TNFAIP8L3	10	8,648,404	8,687,755
					CYP19A1	10	8,712,476	8,726,241
					GLDN	10	8,745,469	8,761,869
					DMXL2	10	8,763,459	8,813,670
					SEMA6D	10	9,404,673	9,419,492
					SLC24A5	10	9,545,024	9,553,009
					MYEF2	10	9,551,752	9,570,906
					SLC12A1	10	9,587,065	9,632,792
2	133,158,961	135,277,884	2,118,923	0.999999177				
					TRPS1	2	134,048,833	134,252,747
					EIF3H	2	134,628,301	134,709,262
					UTP23	2	134,720,381	134,723,977
					RAD21	2	134,749,874	134,773,800
					SLC30A8	2	134,870,849	134,891,418
					MED30	2	134,983,354	135,002,167
1	3,376,561	5,510,024	2,133,463	0.999998524				
					RBM17	1	3,413,571	3,428,042
					PFKFB3	1	3,450,218	3,478,178
					PRKCQ	1	3,559,595	3,595,000
					SFMBT2	1	3,825,402	3,921,158
					ITIH5	1	4,052,014	4,096,123
					ITIH2	1	4,053,533	4,127,906
					KIN	1	4,129,715	4,144,409
					ATP5C1	1	4,144,466	4,151,271
					TAF3	1	4,153,923	4,268,028
					GATA3	1	4,288,842	4,318,191
3	56,447,732	58,590,234	2,142,502	0.999998344				
					CTGF	3	56,638,964	56,641,934
					ENPP1	3	56,656,447	56,707,127
					ENPP3	3	56,723,839	56,757,736
					MED23	3	56,762,370	56,797,379
					AKAP7	3	56,878,364	56,949,970
					EPB41L2	3	56,980,129	57,075,289
					SAMD3	3	57,322,882	57,363,225
					L3MBTL3	3	57,360,442	57,425,789
					C6ORF191	3	57,466,325	57,472,268
					ARHGAP18	3	57,506,532	57,565,989
					LAMA2	3	57,586,334	57,923,906
					PTPRK	3	58,029,862	58,422,557
					THEMIS	3	58,437,710	58,506,141
					C6orf58	3	58,527,005	58,539,770
					TMEM200A	3	57,238,296	57,239,765
2	102,945,374	105,091,268	2,145,894	0.999998092				
					OSBPL1A	2	102,948,952	103,020,897
					IMPACT	2	103,033,304	103,051,363
					ZNF521	2	103,383,680	103,410,706
					SS18	2	103,745,620	103,788,130
					TAF4B	2	103,821,689	103,883,809
					KCTD1	2	103,913,222	103,977,131
					AQP4	2	104,100,790	104,109,628
					CHST9	2	104,126,305	104,127,381
					CDH2	2	104,449,184	104,562,821
2	120,101,406	122,248,807	2,147,401	0.99999801				
					STMN2	2	120,505,360	120,515,753

					HEY1	2	120,538,764	120,541,690
					TPD52	2	120,668,873	120,704,236
					ZBTB10	2	120,783,328	120,809,194
					ZNF704	2	120,881,814	120,900,516
					PAG1	2	120,988,402	121,062,954
					FABP5	2	121,148,557	121,153,615
					PMP2	2	121,215,806	121,220,355
					FABP4	2	121,225,723	121,228,944
					IMPA1	2	121,262,379	121,277,486
					ZFAND1	2	121,279,568	121,285,857
					CHMP4C	2	121,287,839	121,304,205
					SNX16	2	121,309,189	121,333,508
3	93,488,681	95,639,533	2,150,852	0.999997886				
					CMPK2	3	94,575,875	94,584,183
					RSAD2	3	94,589,239	94,594,293
					RNF144A	3	94,613,864	94,661,730
					ID2	3	95,424,295	95,426,233
					KIDINS220	3	95,445,100	95,500,211
					MBOAT2	3	95,513,204	95,603,900
					SOX11	3	94,137,728	94,138,918
5	36,000,903	38,160,604	2,159,701	0.999997348				
					SLC25A21	5	36,015,953	36,247,167
					MIPOL1	5	36,277,202	36,335,031
					TTC6	5	36,464,047	36,517,135
					SEC23A	5	36,843,282	36,865,890
					GEMIN2	5	36,871,372	36,880,109
					TRAPPC6B	5	36,880,151	36,883,970
					PNN	5	36,884,690	36,894,010
					FBXO33	5	36,932,268	36,950,848
					ZNF410	5	36,952,636	36,968,699
					FAM161B	5	36,969,905	36,977,054
					COQ6	5	36,978,464	36,985,973
					ENTPD5	5	36,989,049	37,003,244
					CCDC176	5	37,003,760	37,012,621
					ALDH6A1	5	37,016,493	37,025,856
					LIN52	5	37,025,663	37,064,969
					VSX2	5	37,105,886	37,125,352
					ABCD4	5	37,135,753	37,148,405
					SYNDIG1L	5	37,167,461	37,170,933
					ISCA2	5	37,203,490	37,204,559
					NPC2	5	37,207,826	37,210,000
					LTBP2	5	37,210,588	37,268,314
					KIAA0317	5	37,286,003	37,304,516
					FCF1	5	37,308,073	37,313,662
					YLPM1	5	37,315,712	37,349,907
					PROX2	5	37,363,125	37,365,789
					DLST	5	37,370,864	37,384,872
					RPS6KL1	5	37,387,519	37,391,470
					PGF	5	37,393,657	37,400,127
					EIF2B2	5	37,406,611	37,412,371
					MLH3	5	37,413,704	37,431,030
					ACYP1	5	37,431,802	37,432,761
					FAM164C	5	37,434,156	37,436,408
					NEK9	5	37,442,481	37,464,017
					TMED10	5	37,468,792	37,483,006
					FOS	5	37,501,649	37,502,995
					BATF	5	37,562,117	37,567,648
					FLVCR2	5	37,575,337	37,599,366
					C14orf1	5	37,607,278	37,608,782
					TTLL5	5	37,609,205	37,728,665
					TGFB3	5	37,739,714	37,747,666
					IFT43	5	37,756,375	37,800,040
					C14ORF118	5	37,839,126	37,870,137
					ESRRB	5	37,917,633	38,040,716

					VASH1	5	38,144,496	38,157,153
					SS1R	5	36,632,383	36,633,663
					VRTN	5	37,158,585	37,161,565
9	20,542,558	22,709,637	2,167,079	0.999996829				
					SLITRK3	9	20,586,079	20,587,887
					OTOL1	9	21,401,027	21,404,693
					SPTSSB	9	21,417,214	21,425,710
					NMD3	9	21,436,129	21,447,358
					PPM1L	9	21,476,302	21,557,159
					KPNA4	9	21,597,000	21,610,445
					SMC4	9	21,626,305	21,657,730
					IFT80	9	21,661,987	21,703,750
					IL12A	9	21,727,352	21,729,152
					IQCJ	9	21,810,250	21,844,816
					MFSD1	9	21,872,165	21,885,523
					RARRES1	9	21,904,934	21,912,426
					GFM1	9	21,913,507	21,933,383
					LXN	9	21,919,321	21,923,817
					MLF1	9	21,936,183	21,948,704
					RSRC1	9	21,958,768	22,066,377
					VEPH1	9	22,173,837	22,221,855
					PTX3	9	22,180,190	22,186,891
					CCNL1	9	22,236,549	22,248,075
					LEKR1	9	22,281,614	22,298,405
					TIPARP	9	22,313,993	22,346,827
					SSR3	9	22,347,715	22,354,679
					GMPS	9	22,393,885	22,417,726
					SLC33A1	9	22,418,561	22,429,141
					C3orf33	9	22,430,628	22,434,432
					MME	9	22,527,165	22,563,563
					GPR149	9	22,627,534	22,648,732
					DHX36	9	22,652,324	22,670,920
					B3GALNT1	9	21,566,727	21,567,710
					ARL14	9	21,572,455	21,573,027
3	45,156,314	47,328,829	2,172,515	0.999996002				
					UTRN	3	45,215,648	45,566,922
					EPM2A	3	45,769,623	45,811,385
					FBXO30	3	45,824,455	45,831,133
					SHPRH	3	45,868,769	45,921,436
					GRM1	3	45,937,157	46,118,743
					RAB32	3	46,154,328	46,173,538
					STXBP5	3	46,388,675	46,489,008
					SAMD5	3	46,522,593	46,647,011
					SASH1	3	46,930,247	47,049,931
					UST	3	47,153,562	47,250,156
					STX11	3	45,190,578	45,191,444
1	95,031,571	97,207,250	2,175,679	0.999995066				
					ROBO1	1	95,642,548	95,948,471
3	96,762,582	98,960,307	2,197,725	0.999992075				
					E2F6	3	96,852,537	96,861,622
					GREB1	3	96,906,122	96,965,158
					LPIN1	3	96,971,362	97,044,067
					TRIB2	3	97,427,885	97,448,910
					FAM84A	3	98,116,394	98,118,503
					NBAS	3	98,343,307	98,504,175
					DDX1	3	98,505,120	98,526,381
					MYCN	3	98,778,414	98,780,948
3	49,175,304	51,453,133	2,277,829	0.999943487				
					OPRM1	3	49,319,843	49,341,770
					SCAF8	3	49,604,475	49,635,861
					TFB1M	3	49,868,438	49,895,829
					CLDN20	3	49,875,601	49,880,776
					NOX3	3	49,915,188	49,952,437
					ARID1B	3	50,465,764	50,754,522

					TMEM242	3	50,810,196	50,829,319
					SNX9	3	50,997,690	51,052,016
					SYNJ2	3	51,063,895	51,127,835
					SERAC1	3	51,133,713	51,152,940
					GTF2H5	3	51,154,353	51,158,831
					TULP4	3	51,191,083	51,299,301
					TMEM181	3	51,320,725	51,350,651
					DYNLT1	3	51,354,922	51,358,678
					SYTL3	3	51,364,644	51,389,519
					EZR	3	51,391,093	51,427,199
3	77,406,587	79,690,855	2,284,268	0.999935111				
					TPBG	3	77,636,164	77,637,640
					IBTK	3	77,659,755	77,717,628
					FAM46A	3	77,839,636	77,873,063
					BCKDHB	3	78,457,902	78,570,667
					TTK	3	78,612,134	78,643,792
					ELOVL4	3	78,677,336	78,713,537
					SH3BGRL2	3	78,756,279	78,772,633
					LCA5	3	78,816,727	78,828,429
					HMGN3	3	78,877,354	78,901,407
					PHIP	3	78,926,379	79,016,040
					IRAK1BP1	3	79,040,637	79,051,664
					HTR1B	3	79,492,220	79,493,385
12	9,123,361	11,458,024	2,334,663	0.99980254				
					RPN1	12	9,149,093	9,156,192
					GATA2	12	9,185,919	9,200,490
					EEFSEC	12	9,379,038	9,490,404
					RUVBL1	12	9,495,555	9,513,271
					SEC61A1	12	9,527,460	9,536,843
					KBTBD12	12	9,552,454	9,577,601
					MGLL	12	9,598,736	9,655,915
					ABTB1	12	9,657,435	9,685,045
					PODXL2	12	9,689,545	9,717,481
					MCM2	12	9,725,752	9,737,075
					GPR175	12	9,738,594	9,754,460
					PLXNA1	12	9,887,588	9,971,748
					TXNRD3	12	10,366,876	10,387,808
					CHST13	12	10,449,680	10,473,797
					SLC41A3	12	10,475,439	10,492,889
					KLF15	12	10,614,316	10,626,926
					UROC1	12	10,667,219	10,697,052
					CHCHD4	12	10,756,017	10,764,587
					TMEM43	12	10,764,796	10,775,842
					XPC	12	10,776,979	10,787,331
					SLC6A6	12	10,950,982	10,995,867
					C12H3ORF19	12	11,234,246	11,245,587
					TMA7	12	11,249,996	11,253,626
					CCDC51	12	11,253,813	11,258,331
					PLXNB1	12	11,277,818	11,327,866
					SETD5	12	11,367,736	11,404,678
					LHFPL4	12	11,407,844	11,414,232
					MTMR14	12	11,415,875	11,444,558
					CPNE9	12	11,445,448	11,449,301
					DNAJB8	12	9,245,863	9,246,974
6	22,961,468	25,352,313	2,390,845	0.999498544				
					NT5C2	6	23,043,685	23,098,818
						6	23,104,676	23,106,678
					PCGF6	6	23,110,411	23,132,990
					TAF5	6	23,133,668	23,144,709
					USMG5	6	23,144,647	23,148,454
					PDCD11	6	23,149,107	23,171,490
					CALHM2	6	23,175,699	23,177,288
					CALHM1	6	23,181,808	23,183,651
					CALHM3	6	23,187,673	23,189,859

					NEURL	6	23,201,517	23,337,251
					SH3PXD2A	6	23,349,107	23,406,139
					OBFC1	6	23,585,345	23,623,213
					SLK	6	23,623,564	23,666,255
					COL17A1	6	23,670,969	23,706,526
					SFR1	6	23,730,903	23,734,477
					WDR96	6	23,736,120	23,778,750
					CCDC147	6	23,826,509	23,881,678
					SORCS3	6	23,968,010	24,235,717
					SORCS1	6	24,664,740	24,940,362
					XPNPEP1	6	25,219,899	25,248,494
					ADD3	6	25,264,735	25,352,280
					ITPRIP	6	23,801,103	23,802,752
2	5,328,195	7,833,125	2,504,930	0.996978591				
					SCN5A	2	5,408,261	5,613,834
					EXOG	2	5,692,041	5,711,470
					ACVR2B	2	5,721,529	5,739,855
					XYLB	2	6,140,053	6,166,029
					NUB1	2	6,187,456	6,200,306
					WDR86	2	6,203,237	6,221,340
					CRYGN	2	6,235,658	6,239,901
					PRKAG2	2	6,247,476	6,461,068
					GALNT11	2	6,503,696	6,527,983
					MLL3	2	6,528,611	6,717,633
					XRCC2	2	6,755,237	6,767,527
					DPP6	2	7,411,564	7,635,852
					PAXIP1	2	7,658,648	7,692,640
					HTR5A	2	7,711,450	7,714,285
					INSIG1	2	7,827,461	7,832,977
2	116,407,731	118,932,805	2,525,074	0.996129137				
					LACTB2	2	116,455,967	116,473,097
					XKR9	2	116,481,189	116,488,707
					EYA1	2	116,629,419	116,718,405
					MSC	2	116,915,841	116,918,080
					TRPA1	2	116,997,437	117,055,053
					KCNB2	2	117,126,579	117,312,904
					TERF1	2	117,328,695	117,352,082
					SBSPON	2	117,350,076	117,361,786
					RPL7	2	117,431,264	117,437,199
					RDH10	2	117,438,975	117,467,086
					STAU2	2	117,503,018	117,648,491
					UBE2W	2	117,680,876	117,713,123
					TMEM70	2	117,735,622	117,739,636
					LY96	2	117,745,310	117,753,197
					JPH1	2	117,820,324	117,897,580
					GDAP1	2	117,914,310	117,920,391
					PI15	2	118,088,996	118,111,354
					CRISPLD1	2	118,186,852	118,223,238
					HNF4G	2	118,421,432	118,442,821
11	3,163,524	5,711,059	2,547,535	0.994737636				
					MMP2	11	3,182,494	3,220,410
					IRX5	11	3,356,406	3,358,310
					FTO	11	4,129,194	4,353,272
					RPGRIP1L	11	4,357,663	4,419,746
					AKTIP	11	4,456,387	4,469,147
					RBL2	11	4,474,789	4,490,821
					CHD9	11	4,503,008	4,563,980
					TOX3	11	4,752,569	4,824,785
					SALL1	11	5,418,071	5,433,815
					CYLD	11	5,636,333	5,654,522
					SNX20	11	5,663,396	5,670,064
1	164,506,093	167,067,658	2,561,565	0.993754265				
					OLFM4	1	164,871,751	164,897,395
					PCDH8	1	164,995,573	165,000,858

					LECT1	1	165,018,166	165,029,891
					SUGT1	1	165,034,517	165,057,072
					ELF1	1	165,058,991	165,145,844
					WBP4	1	165,145,975	165,167,892
					MTRF1	1	165,172,543	165,185,717
					NAA16	1	165,191,023	165,255,094
					RGCC	1	165,264,942	165,276,995
					VWA8	1	165,302,186	165,480,795
					DGKH	1	165,513,387	165,674,975
					AKAP11	1	165,702,876	165,738,647
					TNFSF11	1	165,817,349	165,839,120
					EPSTI1	1	165,967,144	166,019,257
					DNAJC15	1	166,031,302	166,055,788
					ENOX1	1	166,088,091	166,385,249
					CCDC122	1	166,476,400	166,486,181
					LACC1	1	166,492,231	166,512,495
					TSC22D1	1	166,717,963	166,796,518
					NUFIP1	1	166,904,655	166,927,469
					KIAA1704	1	166,927,078	166,942,984
					GTF2F2	1	166,965,970	167,057,679
					TPT1	1	167,058,420	167,066,619
					KCTD4	1	166,995,127	166,995,900
7	30,893,512	33,494,400	2,600,888	0.991047478				
					SPOPL	7	30,896,072	30,910,605
					NXPH2	7	30,928,491	30,961,286
					KYNU	7	32,143,918	32,202,554
					ARHGAP15	7	32,211,979	32,530,918
					GTDC1	7	32,617,757	32,782,028
					ZEB2	7	32,812,269	32,916,777
1	20,525,913	23,173,074	2,647,161	0.984389725				
					ZNF800	1	20,644,895	20,659,015
					GRM8	1	20,700,732	21,020,127
					POT1	1	21,513,823	21,575,759
					GPR37	1	21,614,383	21,629,956
					SPAM1	1	21,866,991	21,875,891
					WASL	1	21,980,330	22,030,621
					LMOD2	1	22,047,781	22,055,493
					ASB15	1	22,059,754	22,075,013
					NDUFA5	1	22,078,542	22,084,456
					IQUB	1	22,086,126	22,106,344
					SLC13A1	1	22,194,831	22,218,981
					CADPS2	1	22,359,460	22,601,087
					FEZF1	1	22,608,523	22,610,729
					AASS	1	22,638,791	22,669,004
					PTPRZ1	1	22,669,170	22,806,015
					FAM3C	1	22,979,821	23,107,885
					WNT16	1	23,012,648	23,023,872
4	63,927,174	66,618,821	2,691,647	0.978156613				
					DLC1	4	63,979,467	64,199,133
					C8ORF79	4	64,216,397	64,317,827
					LONRF1	4	64,323,174	64,337,738
					AIRC	4	64,341,542	64,377,673
					PPAT	4	64,350,166	64,393,109
					AASDH	4	64,396,365	64,418,972
					KIAA1211	4	64,418,839	64,453,487
					CEP135	4	64,533,647	64,563,061
					EXOC1	4	64,565,276	64,587,028
					NMU	4	64,646,052	64,657,877
					PDCL2	4	64,659,119	64,664,541
					CLOCK	4	64,696,007	64,717,393
					SRD5A3	4	64,724,586	64,735,325
					SRD5A3	4	64,738,412	64,744,643
					KDR	4	64,794,087	64,824,189
					KIT	4	64,878,556	64,930,842

					PDGFRA	4	65,003,747	65,031,783
					CHIC2	4	65,084,086	65,104,823
					LNX1	4	65,267,149	65,328,349
					FIP1L1	4	65,345,818	65,366,325
					SCFD2	4	65,537,526	65,550,782
					RASL11B	4	65,555,016	65,557,517
					USP46	4	65,597,260	65,618,172
					SPATA18	4	65,750,624	65,771,219
					SGCB	4	65,771,639	65,777,574
					DCUN1D4	4	65,794,330	65,821,389
					CWH43	4	65,834,288	65,859,538
					OCIAD1	4	65,878,670	65,892,164
					FRYL	4	65,919,831	66,048,744
					SLC10A4	4	66,054,693	66,056,492
					SLAIN2	4	66,066,500	66,094,716
					TEC	4	66,112,142	66,153,920
					TXK	4	66,159,821	66,172,212
					NIPAL1	4	66,175,121	66,186,188
					CNGA1	4	66,194,847	66,198,930
					NFXL1	4	66,200,755	66,242,146
					CORIN	4	66,262,035	66,367,775
					ATP10D	4	66,374,739	66,404,130
					COMMD8	4	66,422,820	66,429,658
					GABRB1	4	66,433,150	66,478,683
					GABRA4	4	66,540,864	66,576,459
9	15,666,220	18,477,568	2,811,348	0.94641408				
					EIF4A2	9	15,671,724	15,678,579
					RFC4	9	15,678,911	15,690,512
					MCF2L2	9	15,742,979	15,839,708
					LAMP3	9	15,845,910	15,857,555
					MCCC1	9	15,864,983	15,880,897
					DCUN1D1	9	15,894,430	15,904,156
					ATP11B	9	15,908,775	16,005,533
					DNAJC19	9	16,559,670	16,562,902
					FXR1	9	16,565,596	16,587,667
					CCDC39	9	16,646,100	16,662,762
					TTC14	9	16,663,283	16,672,397
					PEX5L	9	16,760,382	16,850,020
					USP13	9	16,861,502	16,902,461
					NDUFB5	9	16,903,953	16,907,474
					MRPL47	9	16,907,572	16,911,041
					ACTL6A	9	16,911,951	16,921,482
					GNB4	9	16,972,459	16,979,703
					MFN1	9	16,984,938	17,003,984
					ZNF639	9	17,007,571	17,011,621
					PIK3CA	9	17,020,759	17,042,366
					ZMAT3	9	17,052,618	17,062,023
					NAALADL2	9	17,795,977	17,991,569
					NLGN1	9	18,196,758	18,471,834
					B3GNT5	9	15,777,289	15,778,413
					SOX2	9	16,342,563	16,343,510
2	15,286,481	18,178,069	2,891,588	0.909706722				
					MPP7	2	15,410,320	15,499,606
					ARMC4	2	15,517,575	15,589,648
					MKX	2	15,606,690	15,649,798
					RAB18	2	15,704,613	15,717,690
					YME1L1	2	15,807,104	15,827,193
					MASTL	2	15,830,069	15,845,780
					ACBD5	2	15,848,492	15,875,313
					ABI1	2	15,897,343	15,947,115
					PDSS1	2	15,977,931	15,999,697
					APBB1IP	2	16,026,711	16,084,527
					GAD2	2	16,147,743	16,180,423
					MYO3A	2	16,182,205	16,276,588

						GPR179	2	16,372,423	16,557,109
						ENKUR	2	16,613,396	16,622,552
						PRTFDC1	2	16,631,918	16,676,613
						ARHGAP21	2	16,677,325	16,817,876
						KIAA1217	2	16,817,533	17,151,976
						OTUD1	2	17,237,193	17,240,043
						C10orf67	2	17,250,380	17,288,284
						PTF1A	2	17,298,877	17,299,386
						ARMC3	2	17,331,978	17,388,781
						PIP4K2A	2	17,424,993	17,525,165
						SPAG6	2	17,577,501	17,600,696
						COMMD3	2	17,625,279	17,653,946
						DNAJC1	2	17,662,537	17,798,689
						MLLT10	2	17,799,470	17,915,028
							2	17,930,605	17,933,285
						THNSL1	2	16,606,746	16,608,950
	2	136,771,293	139,781,764	3,010,471	0.841132774				
						HAS2	2	136,937,865	136,952,782
						DERL1	2	137,483,314	137,498,444
						WDR67	2	137,498,743	137,523,642
						FAM83A	2	137,525,083	137,540,453
						ATAD2	2	137,586,976	137,621,986
						WDYHV1	2	137,622,325	137,632,958
						FBXO32	2	137,644,051	137,668,702
						KLHL38	2	137,696,848	137,701,773
						ANXA13	2	137,709,300	137,734,052
						FAM91A1	2	137,737,298	137,762,174
						FER1L6	2	137,800,984	137,872,216
						TMEM65	2	137,986,349	138,016,847
						TATDN1	2	138,058,219	138,071,047
						NDUFB9	2	138,071,064	138,074,938
						MTSS1	2	138,077,064	138,193,553
						SQLE	2	138,265,073	138,279,203
						KIAA0196	2	138,283,684	138,306,583
						NSMCE2	2	138,307,230	138,433,695
						TRIB1	2	138,436,990	138,444,511
						FAM84B	2	138,873,014	138,876,023
						MYC	2	139,316,928	139,321,894
						ZHX2	2	137,459,621	137,462,113
						ZHX1	2	137,565,618	137,568,239
						RNF139	2	138,055,501	138,057,312
z		57,240,399	60,504,813	3,264,414	0.648941885				
	7	14,087,975	17,587,110	3,499,135	0.457488833				
						CWC22	7	14,281,313	14,312,291
						ZNF385B	7	14,343,682	14,477,554
						SESTD1	7	14,533,688	14,570,925
						CCDC141	7	14,582,883	14,641,810
						PLEKHA3	7	14,887,747	14,903,030
						FKBP7	7	14,902,995	14,907,636
						DFNB59	7	14,909,332	14,917,988
						OSBPL6	7	14,935,828	14,977,668
						RBM45	7	15,031,727	15,042,794
						PDE11A	7	15,051,870	15,174,859
						AGPS	7	15,219,971	15,271,809
						NFE2L2	7	15,304,546	15,320,356
						HNRNPA3	7	15,327,805	15,341,998
						MTX2	7	15,709,764	15,742,887
						HOXD3	7	15,763,092	15,765,488
						HOXD4	7	15,777,579	15,779,976
						HOXD8	7	15,792,590	15,820,406
						HOXD9	7	15,798,954	15,800,275
						HOXD10	7	15,804,061	15,806,437
						HOXD11	7	15,814,096	15,815,924
						HOXD12	7	15,820,669	15,822,513

					HOXD13	7	15,827,616	15,828,537
					EVX2	7	15,833,129	15,835,917
					KIAA1715	7	15,868,345	15,908,061
					ATP5G3	7	16,150,149	16,153,215
					ATF2	7	16,159,461	16,206,145
					CHN1	7	16,219,611	16,299,658
					CHRNA1	7	16,305,300	16,310,101
					WIPF1	7	16,313,906	16,361,739
					GPR155	7	16,380,664	16,402,658
					SCRN3	7	16,404,276	16,412,287
					CIR1	7	16,412,437	16,433,377
					OLA1	7	16,463,674	16,548,896
					SP3	7	16,580,306	16,610,757
					CDCA7	7	16,830,040	16,838,026
					ZAK	7	16,842,857	16,940,702
					PDK1	7	17,199,467	17,208,239
					ITGA6	7	17,214,612	17,252,865
					DLX1	7	17,337,963	17,338,619
					METAP1D	7	17,340,599	17,361,534
					HAT1	7	17,393,716	17,411,970
					SLC25A12	7	17,412,087	17,457,269
					DYNC1I2	7	17,460,829	17,485,000
					CYBRD1	7	17,504,783	17,516,252
					DCAF17	7	17,519,610	17,538,308
					METTL8	7	17,539,023	17,564,353
2	84,215,295	87,814,090	3,598,795	0.38116927				
					TMEM245	2	84,221,677	84,295,762
					C9ORF4	2	84,305,512	84,313,728
					EPB41L4B	2	84,337,586	84,458,094
					PTPN3	2	84,632,425	84,785,709
					BAG1	2	85,647,213	85,656,438
					CHMP5	2	85,656,826	85,665,787
					SDHA	2	85,681,939	85,697,842
					CCDC127	2	85,697,949	85,702,285
					SLC6A19	2	85,710,095	85,731,320
					SLC6A18	2	85,732,747	85,758,741
					TERT	2	85,762,002	85,791,468
					CLPTM1L	2	85,803,794	85,834,463
					LPCAT1	2	85,846,135	85,899,078
					NDUFS6	2	86,002,617	86,007,620
					IRX4	2	86,065,381	86,068,294
					IRX2	2	86,624,614	86,631,727
					IRX1	2	87,131,471	87,135,061
1	172,838,865	176,586,808	3,747,943	0.289411101				
					RFC3	1	173,144,179	173,161,079
					STARD13	1	173,241,712	173,493,625
					KL	1	173,504,524	173,551,941
					PDS5B	1	173,655,059	173,730,298
					N4BP2L1	1	173,808,326	173,820,489
					BRCA2	1	173,820,724	173,857,456
					ZAR1	1	173,859,596	173,861,411
					FRY	1	173,863,589	174,002,319
					B3GALTL	1	174,203,173	174,247,847
					HSPH1	1	174,262,982	174,286,406
					ALOX5AP	1	174,335,143	174,344,760
					USPL1	1	174,350,051	174,363,188
					KATNAL1	1	174,496,738	174,524,653
					UBL3	1	174,588,070	174,645,255
					SLC7A1	1	174,687,111	174,726,955
					MTUS2	1	174,734,826	174,993,334
					SLC46A3	1	175,029,983	175,043,799
					POMP	1	175,045,977	175,053,620
					FLT1	1	175,084,526	175,185,951
					PAN3	1	175,190,314	175,268,212

					FLT3	1	175,281,894	175,309,466
					CDX2	1	175,315,018	175,317,966
					PDX1	1	175,329,114	175,332,493
					LNX2	1	175,450,252	175,475,674
					MTIF3	1	175,511,067	175,514,091
					GTF3A	1	175,514,019	175,518,424
					RASL11A	1	175,565,741	175,567,390
					WASF3	1	175,770,579	175,792,411
					CDK8	1	175,874,647	175,945,957
					RNF6	1	175,952,258	175,958,160
					ATP8A2	1	175,972,298	176,281,155
					SHISA2	1	176,294,304	176,296,214
					NUPL1	1	176,300,071	176,338,858
					MTMR6	1	176,338,945	176,363,997
					AMER2	1	176,394,869	176,399,685
					GPR12	1	175,735,854	175,736,852
6	26,136,406	29,922,314	3,785,908	0.269006532				
					GPAM	6	26,158,125	26,183,815
					TECTB	6	26,215,254	26,223,330
					ACSL5	6	26,247,834	26,264,411
					ZDHHC6	6	26,264,533	26,277,485
					VTI1A	6	26,277,903	26,526,700
					TCF7L2	6	26,590,486	26,762,220
					HABP2	6	26,971,704	26,991,533
					NRAP	6	26,991,279	27,037,248
					CASP7	6	27,042,298	27,062,965
					PLEKHS1	6	27,064,759	27,081,909
					DCLRE1A	6	27,087,946	27,102,495
					NHLRC2	6	27,103,400	27,134,851
					C10ORF118	6	27,217,490	27,246,216
					TDRD1	6	27,245,682	27,267,251
					COL9A3	6	27,267,818	27,285,089
					AFAP1L2	6	27,287,975	27,335,710
					ABLIM1	6	27,359,430	27,532,008
					FAM160B1	6	27,536,767	27,571,562
					TRUB1	6	27,594,383	27,622,142
					ATRNL1	6	27,680,834	28,014,799
					GFRA1	6	28,152,303	28,288,081
					CCDC172	6	28,309,882	28,328,718
					HSPA12A	6	28,413,790	28,450,155
					ENO4	6	28,500,759	28,518,682
					KIAA1598	6	28,516,161	28,575,056
					KCNK18	6	28,649,189	28,653,448
					PDZD8	6	28,696,248	28,747,446
					EMX2	6	28,814,903	28,820,582
					RAB11FIP2	6	29,003,777	29,047,647
					FAM204A	6	29,173,024	29,190,310
					C6H10ORF46	6	29,348,791	29,388,352
					EIF3A	6	29,508,820	29,537,182
					FAM45A	6	29,538,376	29,547,379
					SFXN4	6	29,550,386	29,559,570
					PRDX3	6	29,560,387	29,565,670
					GRK5	6	29,572,855	29,771,556
					RGS10	6	29,757,675	29,767,309
					TIAL1	6	29,782,065	29,799,900
					BAG3	6	29,813,247	29,832,893
					INPP5F	6	29,851,879	29,889,013
					MCMBP	6	29,884,934	29,905,383
					SEC23IP	6	29,905,638	29,921,730
					ADRB1	6	27,190,009	27,191,226
					NANOS1	6	29,504,247	29,505,285
2	9,608,529	13,749,905	4,141,376	0.130132771				
					VPAC2	2	9,612,723	9,663,652
					ZMYND11	2	9,791,128	9,894,465

					DIP2C	2	9,899,531	9,999,771
					LARP4B	2	10,249,042	10,281,209
					GTPBP4	2	10,356,114	10,367,015
					WDR37	2	10,386,560	10,413,702
					ADARB2	2	10,438,685	10,736,438
					PFKP	2	11,354,185	11,395,733
					PITRM1	2	11,396,672	11,421,465
					GJD4	2	12,778,766	12,787,894
					CCNY	2	12,793,547	12,910,204
					CREM	2	12,930,882	12,960,732
					CUL2	2	12,972,707	13,022,987
					PARD3	2	13,068,736	13,498,548
					FZD8	2	12,761,773	12,762,399
1	132,504,106	137,310,640	4,806,534	0.030166525				
					PDCL3	1	132,518,630	132,524,289
					NPAS2	1	132,655,000	132,723,188
					RPL31	1	132,725,776	132,731,147
					TBC1D8	1	132,731,877	132,781,402
					CNOT11	1	132,803,062	132,814,520
					RNF149	1	132,819,684	132,837,930
					CREG2	1	132,840,094	132,860,137
					RFX8	1	132,861,202	132,895,021
					MAP4K4	1	132,936,573	133,084,109
					IL1R2	1	133,109,396	133,119,021
					IL1RL1	1	133,227,061	133,252,726
					IL18R1	1	133,259,701	133,278,851
					IL18RAP	1	133,283,645	133,298,556
					SLC9A4	1	133,300,715	133,329,117
					SLC9A2	1	133,354,849	133,381,741
					MFSD9	1	133,391,984	133,402,160
					TMEM182	1	133,408,227	133,435,369
					MRPS9	1	134,393,340	134,425,489
					TGFBRAP1	1	134,450,449	134,476,912
					C1H2ORF49	1	134,481,595	134,496,274
					FHL2	1	134,496,864	134,536,397
					NCK2	1	134,633,927	134,714,716
					C2ORF40	1	134,771,683	134,776,149
					ST6GAL2	1	135,007,544	135,053,400
					SLC5A7	1	135,397,400	135,423,764
					SULT1C	1	135,521,670	135,527,319
					RANBP2	1	135,763,887	135,793,418
					EDAR	1	135,819,209	135,882,851
					UPF3A	1	136,254,098	136,279,867
					CDC16	1	136,281,362	136,301,395
					RASA3	1	136,345,769	136,476,626
					GAS6	1	136,509,160	136,547,639
					FAM70B	1	136,551,868	136,614,965
					ATP4B	1	136,636,254	136,641,973
					TMCO3	1	136,694,300	136,726,217
					DCUN1D2	1	136,728,468	136,751,599
					ADPRHL1	1	136,753,731	136,773,443
					GRTP1	1	136,801,888	136,830,896
					LAMP1	1	136,831,179	136,848,516
					CUL4A	1	136,854,216	136,888,547
					PCID2	1	136,889,042	136,902,069
					PROZ	1	136,903,332	136,910,194
					F10	1	136,922,630	136,935,187
					F7	1	136,938,052	136,946,321
					MCF2L	1	136,949,339	137,097,962
					ATP11A	1	137,110,630	137,228,603
					SOWAHC	1	136,196,812	136,198,516
					CHAMP1	1	136,241,415	136,244,261

Supplementary table 11b. Multispecies homologous synteny blocks (msHSBs) detected in archosaurians

Chr	msHSB start (bp)	msHSB end (bp)	msHSB length (bp)	Probability of being found under the Poisson process	Gene ID	Chr	Gene start (bp)	Gene end (bp)
3	75,256,244	76,762,298	1,506,054	1				
					CNR1	3	75,580,393	75,593,814
					SPACA1	3	75,621,239	75,640,693
					AKIRIN2	3	75,777,524	75,782,279
					ORC3L	3	75,787,755	75,824,704
					RARS2	3	75,824,858	75,856,866
					SLC35A1	3	75,858,402	75,874,162
					C6ORF163	3	75,901,121	75,909,870
					C6ORF162	3	75,912,085	75,914,718
					ZNF292	3	75,929,305	75,982,691
					CGA	3	76,000,168	76,009,682
					SYNCRIP	3	76,289,510	76,312,354
					SNX14	3	76,326,433	76,373,661
					NT5E	3	76,375,727	76,397,579
					TBX18	3	76,646,414	76,672,046
					HTR1E	3	76,038,226	76,039,341
1	10,916,110	12,431,817	1,515,707	1				
					CD36	1	11,005,425	11,038,504
					GNAT3	1	11,086,584	11,113,407
					GNAI3	1	11,190,979	11,223,596
					MAGI2	1	11,930,694	12,230,824
					PHTF2	1	12,251,576	12,314,104
					TMEM60	1	12,314,478	12,318,704
					RSBN1L	1	12,327,050	12,358,827
					PTPN18	1	12,381,899	12,422,097
1	145,101,546	146,620,275	1,518,729	1				
					UGGT2	1	145,103,089	145,180,354
					DNAJC3	1	145,184,466	145,216,297
					DZIP1	1	145,229,679	145,262,325
					CLDN10	1	145,266,284	145,279,564
					ABCC4	1	145,358,360	145,506,681
					GPR180	1	145,671,022	145,692,774
					TGDS	1	145,692,790	145,705,488
					DCT	1	145,716,342	145,737,439
2	8,014,363	9,563,687	1,549,324	1				
					SHH	2	8,080,091	8,089,855
					RNF32	2	8,414,446	8,427,357
					LMBR1	2	8,434,744	8,494,298
					NOM1	2	8,513,775	8,529,642
					MNX1	2	8,538,868	8,541,508
					UBE3C	2	8,582,314	8,644,842
					PTPRN2	2	8,785,742	9,406,733
					NCAPG2	2	9,417,857	9,456,238
					ESYT2	2	9,467,167	9,532,244
1	97,365,488	98,957,585	1,592,097	1				
					HSPA13	1	97,411,085	97,418,694
					SAMSN1	1	97,445,928	97,480,511
					USP25	1	97,936,529	98,030,131
					CXADR	1	98,638,187	98,668,892
					C1H21ORF91	1	98,700,854	98,720,026
					CHODL	1	98,864,912	98,893,667
					TMPRSS15	1	98,898,052	98,906,375
					NRIP1	1	97,651,374	97,654,841
4	71,953,595	73,555,681	1,602,086	1				
					STIM2	4	72,683,440	72,741,445
					TBC1D19	4	72,753,287	72,801,383
					CCK1R	4	72,818,063	72,824,919
					RBPJ	4	72,840,334	72,980,021
					C4ORF52	4	72,987,542	72,991,389

					SEL1L3	4	72,995,781	73,024,332
					SLC34A2	4	73,038,472	73,055,616
					ANAPC4	4	73,093,795	73,110,519
					ZCCHC4	4	73,110,600	73,121,105
					PI4K2B	4	73,122,273	73,139,009
					SEPSECS	4	73,142,031	73,165,468
					LGI2	4	73,182,369	73,204,135
					CCDC149	4	73,225,282	73,274,932
					SOD3	4	73,280,251	73,281,120
					DHX15	4	73,318,070	73,361,220
1	190,170,291	191,772,697	1,602,406	1				
					TENM4	1	191,230,962	191,733,590
4	86,080,977	87,690,044	1,609,067	1				
					LRRTM1	4	87,094,015	87,096,026
4	73,572,127	75,214,999	1,642,872	1				
					PPARGC1A	4	73,626,292	73,691,855
					GPR125	4	74,015,279	74,072,320
					KCNIP4	4	74,436,584	74,527,405
					PACRGL	4	74,526,045	74,539,587
					SLIT2	4	74,557,149	74,802,699
3	24,475,771	26,122,614	1,646,843	1				
					PLEKHH2	3	24,491,110	24,545,482
					DYNC2LI1	3	24,548,141	24,570,676
					ABCG5	3	24,567,546	24,588,926
					ABCG8	3	24,585,792	24,598,091
					LRPPRC	3	24,608,804	24,695,385
					PPM1B	3	24,761,763	24,820,317
					SLC3A1	3	24,827,681	24,841,984
					PREPL	3	24,831,731	24,859,714
					SIX2	3	25,240,527	25,243,491
					SRBD1	3	25,432,172	25,549,520
					PRKCE	3	25,576,043	25,856,010
					EPAS1	3	25,951,104	25,982,708
					RHOQ	3	26,075,656	26,091,343
					PIGF	3	26,084,410	26,110,103
					CRIPT	3	26,110,349	26,115,765
1	109,932,747	111,600,312	1,667,565	1				
					CXorf36	1	110,128,634	110,143,679
					FUNDC1	1	110,347,436	110,362,439
					EFHC2	1	110,382,985	110,442,596
					NDP	1	110,522,886	110,531,383
					MAOB	1	110,555,374	110,609,289
					MAOA	1	110,613,430	110,658,911
					GPR34	1	111,232,706	111,235,607
					NYX	1	111,334,091	111,336,368
					GPR82	1	111,219,959	111,220,993
5	33,186,512	34,867,480	1,680,968	1				
					KIAA1333	5	33,413,582	33,433,671
					SCFD1	5	33,438,355	33,483,021
					COCH	5	33,517,885	33,534,657
					AP4S1	5	33,603,694	33,613,992
					HECTD1	5	33,616,118	33,666,611
					HEATR5A	5	33,685,490	33,736,833
					NUBPL	5	33,774,090	33,856,331
					ARHGAP5	5	33,958,351	33,993,431
					AKAP6	5	34,050,021	34,304,671
3	99,384,303	101,081,244	1,696,941	1				
					VSNL1	3	99,739,837	99,814,889
					SMC6	3	99,815,077	99,852,120
					GEN1	3	99,853,931	99,870,493
					KCNS3	3	99,922,984	99,942,855
					RDH14	3	100,213,340	100,217,996
					OSR1	3	100,695,798	100,697,672
					TTC32	3	101,036,992	101,038,388

					WDR35	3	101,039,084	101,075,670
					MSGN1	3	99,887,826	99,889,127
1	80,304,616	82,024,763	1,720,147	1				
					LSAMP	1	81,373,015	81,656,000
					GAP43	1	81,680,485	81,734,254
17	2,877,535	4,606,475	1,728,940	1				
					PAPPA	17	3,040,970	3,209,266
					ASTN2	17	3,219,825	3,482,002
					TLR4	17	3,566,454	3,571,907
					DBC1	17	3,985,882	4,064,290
					CDK5RAP2	17	4,495,102	4,566,562
					MEGF9	17	4,575,673	4,590,722
5	10,087,655	11,834,285	1,746,630	1				
					SOX6	5	10,549,851	10,784,968
					C5H11ORF58	5	10,948,695	10,953,363
					PLEKHA7	5	10,959,448	11,057,508
					RPS13	5	11,136,190	11,139,937
					PIK3C2A	5	11,142,129	11,205,960
					NUCB2	5	11,205,875	11,231,516
					KCNJ11	5	11,247,848	11,250,065
					ABCC8	5	11,253,945	11,319,342
					USH1C	5	11,334,156	11,377,576
					OTOG	5	11,381,876	11,476,414
					MYOD1	5	11,578,793	11,582,278
					KCNC1	5	11,597,391	11,714,071
3	65,651,343	67,404,826	1,753,483	1				
					RPF2	3	65,709,193	65,721,929
					GTF3C6	3	65,721,376	65,726,791
					AMD1	3	65,744,041	65,761,269
					CDK19	3	65,801,524	65,899,585
					SLC22A16	3	65,931,799	65,962,885
					DDO	3	65,969,395	65,975,774
					C6ORF186	3	65,985,390	66,029,579
					CDC40	3	66,033,229	66,074,352
					WASF1	3	66,123,600	66,154,746
					GPR6	3	66,173,387	66,197,130
					FIG4	3	66,201,896	66,268,774
					AK9	3	66,269,578	66,325,155
					ZBTB24	3	66,327,020	66,334,932
					PPIL6	3	66,340,530	66,350,895
					CD164	3	66,357,144	66,365,037
					C6ORF183	3	66,413,334	66,432,788
					CEP57L1	3	66,460,394	66,473,222
					SESN1	3	66,547,545	66,556,746
					ARMC2	3	66,560,205	66,617,959
					FOXO3	3	66,702,483	66,716,393
					LACE1	3	66,809,734	66,870,599
					SNX3	3	66,876,089	66,895,012
					NR2E1	3	66,898,978	66,913,949
					OSTM1	3	66,950,856	66,961,817
					SEC63	3	66,978,001	67,033,488
					SCML4	3	67,042,180	67,098,929
					SOBP	3	67,134,086	67,243,457
					PDSS2	3	67,259,779	67,378,577
					BEND3	3	67,393,979	67,403,976
4	20,823,966	22,596,657	1,772,691	0.999999999				
					GRIA2	4	20,873,025	20,960,836
					FAM198B	4	21,221,107	21,234,597
					TMEM144	4	21,242,338	21,257,193
					RXFP1	4	21,328,064	21,376,386
					ETFDH	4	21,379,598	21,396,998
					PPID	4	21,395,223	21,405,790
					FNIP2	4	21,425,883	21,457,823
					C4ORF45	4	21,455,357	21,496,063

					RAPGEF2	4	21,509,902	21,683,609
					FSTL5	4	22,250,827	22,516,765
8	23,780,361	25,569,631	1,789,270	0.999999999				
					GLIS1	8	23,804,849	23,900,621
					NDC1	8	23,986,245	24,001,606
					YIPF1	8	24,001,645	24,006,175
					DIO1	8	24,006,797	24,011,727
					LRRC42	8	24,015,720	24,020,780
					LDLRAD1	8	24,023,108	24,025,375
					TMEM59	8	24,030,293	24,038,522
					TCEANC2	8	24,038,683	24,041,647
					CDCP2	8	24,045,805	24,049,407
					MRPL37	8	24,064,757	24,068,753
					SSBP3	8	24,074,037	24,124,115
					ACOT11	8	24,131,387	24,140,486
					TTC4	8	24,142,957	24,148,277
					C1ORF177	8	24,152,396	24,156,229
					DHCR24	8	24,157,606	24,165,747
					TMEM61	8	24,173,996	24,176,346
					BSND	8	24,177,461	24,179,735
					PCSK9	8	24,180,817	24,184,784
					USP24	8	24,190,573	24,237,937
					PPAP2B	8	24,600,649	24,643,425
					PRKAA2	8	24,663,005	24,679,845
					C1ORF168	8	24,687,128	24,702,955
					C8A	8	24,711,328	24,727,316
					C8B	8	24,731,782	24,747,335
					DAB1	8	24,753,891	24,842,483
					OMA1	8	25,201,610	25,219,335
					MYSM1	8	25,232,688	25,245,087
					FGGY	8	25,416,352	25,524,129
					HOOK1	8	25,544,038	25,566,897
					PARS2	8	24,148,653	24,149,966
					TACSTD2	8	25,225,850	25,226,746
					JUN	8	25,267,213	25,268,157
2	120,455,577	122,248,807	1,793,230	0.999999998				
					STMN2	2	120,505,360	120,515,753
					HEY1	2	120,538,764	120,541,690
					TPD52	2	120,668,873	120,704,236
					ZBTB10	2	120,783,328	120,809,194
					ZNF704	2	120,881,814	120,900,516
					PAG1	2	120,988,402	121,062,954
					FABP5	2	121,148,557	121,153,615
					PMP2	2	121,215,806	121,220,355
					FABP4	2	121,225,723	121,228,944
					IMPA1	2	121,262,379	121,277,486
					ZFAND1	2	121,279,568	121,285,857
					CHMP4C	2	121,287,839	121,304,205
					SNX16	2	121,309,189	121,333,508
3	35,427,107	37,224,095	1,796,988	0.999999998				
					GREM2	3	35,493,725	35,533,009
					FMN2	3	35,540,378	35,680,173
					CHRM3	3	35,727,538	35,877,494
					RYR2	3	36,498,791	36,661,068
					MTR	3	36,890,573	36,933,337
					ACTN2	3	36,948,211	37,014,188
					HEATR1	3	37,025,129	37,060,227
					LGALS8	3	37,061,029	37,073,707
					EDARADD	3	37,078,799	37,097,401
					ERO1LB	3	37,097,636	37,134,158
					GPR137B	3	37,135,755	37,159,463
					NID1	3	37,178,196	37,213,970
2	133,158,961	134,972,233	1,813,272	0.999999995				
					TRPS1	2	134,048,833	134,252,747

					EIF3H	2	134,628,301	134,709,262
					UTP23	2	134,720,381	134,723,977
					RAD21	2	134,749,874	134,773,800
					SLC30A8	2	134,870,849	134,891,418
2	1,476,281	3,303,402	1,827,121	0.999999991				
					VIPR1	2	1,722,123	1,826,646
					SEC22C	2	1,886,499	1,900,914
					NKTR	2	1,927,422	1,965,795
					ZBTB47	2	1,978,851	1,993,362
					KBTBD5	2	1,999,758	2,009,597
					HHATL	2	2,014,408	2,026,554
					CCDC13	2	2,032,322	2,059,811
					HIGD1C	2	2,061,655	2,066,620
					CCBP2	2	2,072,000	2,073,926
					OBSCN	2	2,080,439	2,253,956
					C2H1ORF69	2	2,295,676	2,300,126
					GJC2	2	2,303,738	2,310,415
					GUK1	2	2,334,607	2,343,799
					MRPL55	2	2,348,731	2,351,041
					C1ORF35	2	2,351,366	2,358,790
					ARF1	2	2,357,324	2,369,250
					WNT3A	2	2,459,534	2,542,486
					WNT9A	2	2,573,612	2,624,503
					SNAP47	2	3,185,315	3,205,912
					JMJD4	2	3,211,699	3,220,712
					ALS2CL	2	3,216,850	3,246,773
					TMIE	2	3,268,001	3,297,394
4	23,185,693	25,022,794	1,837,101	0.999999987				
					TMEM192	4	23,236,487	23,257,108
					KLHL2	4	23,265,508	23,325,234
					SC4MOL	4	23,326,894	23,333,143
					CPE	4	23,336,496	23,382,106
					TLL1	4	23,520,463	23,656,666
					SPOCK3	4	23,947,374	24,051,813
					ANXA10	4	24,400,386	24,416,023
					PALLD	4	24,486,948	24,675,736
					AADAT	4	24,691,532	24,707,952
					MFAP3L	4	24,743,867	24,747,796
					C4ORF27	4	24,796,835	24,803,472
					CLCN3	4	24,804,714	24,864,749
					NEK1	4	24,866,404	24,909,512
					CBR4	4	25,012,882	25,021,145
1	26,819,307	28,677,205	1,857,898	0.999999959				
					C7ORF53	1	26,846,392	26,853,187
					IFRD1	1	26,854,961	26,865,547
					ZNF277	1	26,905,761	26,960,018
					DNAJB9	1	28,382,860	28,392,521
					THAP5	1	28,392,695	28,399,112
					PNPLA8	1	28,473,454	28,508,702
					NRCAM	1	28,525,499	28,668,231
					LRRN3	1	27,413,542	27,415,668
8	25,607,217	27,516,548	1,909,331	0.999999761				
					CNFI-A4	8	25,948,005	26,179,911
					TM2D1	8	26,200,325	26,219,046
					INADL	8	26,226,028	26,357,392
					KANK4	8	26,365,577	26,378,312
					USP1	8	26,391,541	26,401,494
					ANGPTL3	8	26,448,477	26,457,422
					ATG4C	8	26,501,092	26,520,896
					ALG6	8	26,691,601	26,712,046
					ITGB3BP	8	26,713,799	26,739,349
					EFCAB7	8	26,748,611	26,762,766
					PGM1	8	26,764,668	26,783,622
					ROR1	8	26,884,366	26,926,589

						CACHD1	8	26,977,492	27,061,474
						RAVER2	8	27,069,633	27,101,820
						AK3L2	8	27,167,597	27,176,227
						DNAJC6	8	27,186,950	27,217,638
						LEPROT	8	27,220,963	27,226,804
						LEPR	8	27,238,562	27,268,762
						PDE4B	8	27,415,316	27,426,443
						SGIP1	8	27,431,896	27,462,447
						TCTEX1D1	8	27,465,606	27,467,851
						WDR78	8	27,471,548	27,487,186
						MIER1	8	27,487,798	27,516,244
	3	58,723,180	60,639,979	1,916,799	0.999999659				
						RSPO3	3	58,726,123	58,785,427
						CENPW	3	59,060,255	59,067,195
						TRMT11	3	59,178,746	59,205,181
						HINT3	3	59,212,941	59,219,743
						NCOA7	3	59,223,034	59,291,522
						HDDC2	3	59,516,400	59,525,907
						TPD52L1	3	59,527,859	59,580,356
						RNF217	3	59,606,826	59,662,599
						NKAIN2	3	59,710,415	59,999,592
						TRDN	3	60,315,841	60,432,093
						CLVS2	3	60,548,727	60,598,139
z		27,533,307	29,468,950	1,935,643	0.99999939				
	3	81,161,816	83,102,150	1,940,334	0.999999219				
						RIMS1	3	81,337,238	81,638,081
						OGFRL1	3	81,808,877	81,818,348
						B3GAT2	3	81,940,693	81,962,704
						FAM135A	3	82,085,759	82,150,209
						COL9A1	3	82,230,623	82,296,804
						COL19A1	3	82,304,939	82,484,565
						LMBRD1	3	82,508,371	82,577,230
	1	12,815,274	14,761,167	1,945,893	0.999998885				
						RELN	1	12,865,261	13,129,087
						ORC5L	1	13,157,747	13,226,609
						KMT2E	1	13,575,018	13,627,041
						PUS7	1	13,788,331	13,809,208
						RINT1	1	13,810,273	13,822,128
						EFCAB10	1	13,821,811	13,823,862
						ATXN7L1	1	13,831,430	13,898,855
						CDHR3	1	13,960,601	13,995,845
						SYPL1	1	14,001,061	14,009,388
						PIK3CG	1	14,213,411	14,241,547
						PRKAR2B	1	14,328,390	14,346,800
						HBP1	1	14,356,759	14,367,631
						COG5	1	14,369,255	14,553,213
						DUS4L	1	14,553,188	14,563,663
						BCAP29	1	14,567,866	14,591,310
						SLC26A4	1	14,597,269	14,618,545
						SLC26A3	1	14,632,208	14,642,808
						DLD	1	14,666,134	14,679,885
						LAMB1	1	14,681,577	14,715,087
						CCDC71L	1	14,180,471	14,180,770
						GPR22	1	14,518,793	14,520,725
	1	141,339,838	143,294,319	1,954,481	0.999998569				
						SLC10A2	1	141,914,133	141,925,460
						BIVM	1	142,024,553	142,041,190
						KDELC1	1	142,045,388	142,054,410
						C13ORF27	1	142,057,711	142,064,431
						METTL21C	1	142,092,496	142,099,273
						TPP2	1	142,097,596	142,147,887
						ITGBL1	1	142,614,261	142,752,083
						NALCN	1	142,765,240	142,969,863
						TMTC4	1	143,142,401	143,197,059

					A2LD1	1	143,230,157	143,230,654
3	45,173,549	47,136,044	1,962,495	0.999998346				
					UTRN	3	45,215,648	45,566,922
					EPM2A	3	45,769,623	45,811,385
					FBXO30	3	45,824,455	45,831,133
					SHPRH	3	45,868,769	45,921,436
					GRM1	3	45,937,157	46,118,743
					RAB32	3	46,154,328	46,173,538
					STXBP5	3	46,388,675	46,489,008
					SAMD5	3	46,522,593	46,647,011
					SASH1	3	46,930,247	47,049,931
					STX11	3	45,190,578	45,191,444
4	64,648,446	66,618,821	1,970,375	0.999997926				
					PDCL2	4	64,659,119	64,664,541
					CLOCK	4	64,696,007	64,717,393
					SRD5A3	4	64,724,586	64,735,325
					SRD5A3	4	64,738,412	64,744,643
					KDR	4	64,794,087	64,824,189
					KIT	4	64,878,556	64,930,842
					PDGFRA	4	65,003,747	65,031,783
					CHIC2	4	65,084,086	65,104,823
					LNX1	4	65,267,149	65,328,349
					FIP1L1	4	65,345,818	65,366,325
					SCFD2	4	65,537,526	65,550,782
					RASL11B	4	65,555,016	65,557,517
					USP46	4	65,597,260	65,618,172
					SPATA18	4	65,750,624	65,771,219
					SGCB	4	65,771,639	65,777,574
					DCUN1D4	4	65,794,330	65,821,389
					CWH43	4	65,834,288	65,859,538
					OCIAD1	4	65,878,670	65,892,164
					FRYL	4	65,919,831	66,048,744
					SLC10A4	4	66,054,693	66,056,492
					SLAIN2	4	66,066,500	66,094,716
					TEC	4	66,112,142	66,153,920
					TXK	4	66,159,821	66,172,212
					NIPAL1	4	66,175,121	66,186,188
					CNGA1	4	66,194,847	66,198,930
					NFXL1	4	66,200,755	66,242,146
					CORIN	4	66,262,035	66,367,775
					ATP10D	4	66,374,739	66,404,130
					COMMD8	4	66,422,820	66,429,658
					GABRB1	4	66,433,150	66,478,683
					GABRA4	4	66,540,864	66,576,459
3	56,602,089	58,590,234	1,988,145	0.999996285				
					CTGF	3	56,638,964	56,641,934
					ENPP1	3	56,656,447	56,707,127
					ENPP3	3	56,723,839	56,757,736
					MED23	3	56,762,370	56,797,379
					AKAP7	3	56,878,364	56,949,970
					EPB41L2	3	56,980,129	57,075,289
					SAMD3	3	57,322,882	57,363,225
					L3MBTL3	3	57,360,442	57,425,789
					C6ORF191	3	57,466,325	57,472,268
					ARHGAP18	3	57,506,532	57,565,989
					LAMA2	3	57,586,334	57,923,906
					PTPRK	3	58,029,862	58,422,557
					THEMIS	3	58,437,710	58,506,141
					C6orf58	3	58,527,005	58,539,770
					TMEM200A	3	57,238,296	57,239,765
10	13,555,367	15,569,180	2,013,813	0.999991246				
					SV2B	10	13,701,912	13,728,770
					SLCO3A1	10	13,838,315	13,953,221
					ST8SIA2	10	13,994,580	14,019,205

					FAM174B	10	14,039,364	14,055,885
					CHD2	10	14,112,774	14,149,142
					RGMA	10	14,166,477	14,183,190
					MCTP2	10	14,461,079	14,573,856
					NR2F2	10	15,328,163	15,333,861
2	141,251,316	143,287,606	2,036,290	0.999984648				
					TMEM71	2	141,260,434	141,266,499
					PHF20L1	2	141,275,093	141,322,936
					TG	2	141,333,054	141,469,306
					SLA	2	141,410,203	141,432,079
					WISP1	2	141,532,750	141,540,007
					NDRG1	2	141,549,217	141,589,409
					ST3GAL1	2	141,665,331	141,689,879
					ZFAT	2	142,122,786	142,207,755
					KHDRBS3	2	142,700,486	142,776,708
2	112,226,473	114,277,051	2,050,578	0.999977393				
					CA8	2	112,413,504	112,453,419
					RAB2A	2	112,517,303	112,558,232
					CHD7	2	112,612,964	112,699,976
					CLVS1	2	112,882,281	112,978,767
					NKAIN3	2	113,137,211	113,469,039
					GGH	2	113,490,054	113,501,161
					TTPA	2	113,502,155	113,517,713
					YTHDF3	2	113,543,309	113,551,391
					CYP7B1	2	114,093,263	114,114,645
3	49,175,304	51,254,835	2,079,531	0.999954291				
					OPRM1	3	49,319,843	49,341,770
					SCAF8	3	49,604,475	49,635,861
					TFB1M	3	49,868,438	49,895,829
					CLDN20	3	49,875,601	49,880,776
					NOX3	3	49,915,188	49,952,437
					ARID1B	3	50,465,764	50,754,522
					TMEM242	3	50,810,196	50,829,319
					SNX9	3	50,997,690	51,052,016
					SYNJ2	3	51,063,895	51,127,835
					SERAC1	3	51,133,713	51,152,940
					GTF2H5	3	51,154,353	51,158,831
5	29,659,107	31,749,250	2,090,143	0.999942814				
					FMN1	5	29,701,247	29,817,292
					GREM1	5	29,842,277	29,849,273
					SCG5	5	29,864,000	29,890,748
					RASGRP1	5	29,940,334	29,974,856
					FAM98B	5	29,981,575	29,990,845
					SPRED1	5	30,010,890	30,068,434
					MEIS2	5	30,506,655	30,675,196
					C5H15orf41	5	30,717,442	30,832,671
					ATPBD4	5	31,253,451	31,438,936
					ZNF770	5	31,468,292	31,472,971
					AQR	5	31,481,161	31,528,089
					ACTC1	5	31,546,121	31,552,902
					GJD2	5	31,568,439	31,572,498
					STXBP6	5	31,693,846	31,748,417
2	125,160,507	127,250,892	2,090,385	0.999938962				
					TRIQK	2	125,273,982	125,333,683
					FAM92A1	2	125,604,624	125,621,196
					RBM12B	2	125,623,603	125,630,097
					TMEM67	2	125,632,568	125,659,384
					PDP1	2	125,683,595	125,690,722
					CDH17	2	125,754,667	125,783,019
					GEM	2	125,802,915	125,811,572
					RAD54B	2	125,844,098	125,903,394
					FSBP	2	125,877,035	125,887,013
					KIAA1429	2	125,925,066	125,952,171
					ESRP1	2	125,956,620	125,988,739

					DPY19L4	2	126,052,840	126,076,741
					INTS8	2	126,126,452	126,151,324
					CCNE2	2	126,151,226	126,161,848
					TP53INP1	2	126,170,346	126,183,360
					NDUFAF6	2	126,201,059	126,216,326
					PLEKHF2	2	126,225,096	126,240,839
					C8orf37	2	126,258,109	126,269,454
					MTERFD1	2	126,597,212	126,615,684
					PTDSS1	2	126,616,011	126,645,632
					SDC2	2	126,740,060	126,929,143
					MTDH	2	127,057,053	127,088,247
					LAPTM4B	2	127,102,614	127,165,676
					MATN2	2	127,183,885	127,242,408
10	7,538,412	9,634,830	2,096,418	0.999925706				
					UNC13C	10	7,641,038	7,759,221
					WDR72	10	7,807,793	7,874,460
					FAM214A	10	8,193,075	8,224,905
					ARPP19	10	8,227,077	8,235,366
					MYO5A	10	8,240,552	8,332,115
					MYO5C	10	8,334,755	8,365,618
					MAPK6	10	8,418,112	8,446,458
					LEO1	10	8,499,714	8,506,990
					TMOD3	10	8,510,697	8,533,995
					TMOD2	10	8,537,029	8,567,174
					LYSMD2	10	8,581,242	8,590,880
					SCG3	10	8,596,070	8,621,388
					AP4E1	10	8,625,187	8,643,899
					TNFAIP8L3	10	8,648,404	8,687,755
					CYP19A1	10	8,712,476	8,726,241
					GLDN	10	8,745,469	8,761,869
					DMXL2	10	8,763,459	8,813,670
					SEMA6D	10	9,404,673	9,419,492
					SLC24A5	10	9,545,024	9,553,009
					MYEF2	10	9,551,752	9,570,906
					SLC12A1	10	9,587,065	9,632,792
2	85,709,755	87,814,090	2,104,335	0.999914874				
					SLC6A19	2	85,710,095	85,731,320
					SLC6A18	2	85,732,747	85,758,741
					TERT	2	85,762,002	85,791,468
					CLPTM1L	2	85,803,794	85,834,463
					LPCAT1	2	85,846,135	85,899,078
					NDUFS6	2	86,002,617	86,007,620
					IRX4	2	86,065,381	86,068,294
					IRX2	2	86,624,614	86,631,727
					IRX1	2	87,131,471	87,135,061
1	172,850,666	174,964,633	2,113,967	0.999888485				
					RFC3	1	173,144,179	173,161,079
					STARD13	1	173,241,712	173,493,625
					KL	1	173,504,524	173,551,941
					PDS5B	1	173,655,059	173,730,298
					N4BP2L1	1	173,808,326	173,820,489
					BRCA2	1	173,820,724	173,857,456
					ZAR1	1	173,859,596	173,861,411
					FRY	1	173,863,589	174,002,319
					B3GALTL	1	174,203,173	174,247,847
					HSPH1	1	174,262,982	174,286,406
					ALOX5AP	1	174,335,143	174,344,760
					USPL1	1	174,350,051	174,363,188
					KATNAL1	1	174,496,738	174,524,653
					UBL3	1	174,588,070	174,645,255
					SLC7A1	1	174,687,111	174,726,955
12	9,123,361	11,244,728	2,121,367	0.999870507				
					RPN1	12	9,149,093	9,156,192
					GATA2	12	9,185,919	9,200,490

					EEFSEC	12	9,379,038	9,490,404
					RUVBL1	12	9,495,555	9,513,271
					SEC61A1	12	9,527,460	9,536,843
					KBTBD12	12	9,552,454	9,577,601
					MGLL	12	9,598,736	9,655,915
					ABTB1	12	9,657,435	9,685,045
					PODXL2	12	9,689,545	9,717,481
					MCM2	12	9,725,752	9,737,075
					GPR175	12	9,738,594	9,754,460
					PLXNA1	12	9,887,588	9,971,748
					TXNRD3	12	10,366,876	10,387,808
					CHST13	12	10,449,680	10,473,797
					SLC41A3	12	10,475,439	10,492,889
					KLF15	12	10,614,316	10,626,926
					UROC1	12	10,667,219	10,697,052
					CHCHD4	12	10,756,017	10,764,587
					TMEM43	12	10,764,796	10,775,842
					XPC	12	10,776,979	10,787,331
					SLC6A6	12	10,950,982	10,995,867
					DNAJB8	12	9,245,863	9,246,974
3	93,512,293	95,639,533	2,127,240	0.999863093				
					CMPK2	3	94,575,875	94,584,183
					RSAD2	3	94,589,239	94,594,293
					RNF144A	3	94,613,864	94,661,730
					ID2	3	95,424,295	95,426,233
					KIDINS220	3	95,445,100	95,500,211
					MBOAT2	3	95,513,204	95,603,900
					SOX11	3	94,137,728	94,138,918
1	3,376,561	5,510,024	2,133,463	0.999823734				
					RBM17	1	3,413,571	3,428,042
					PFKFB3	1	3,450,218	3,478,178
					PRKCQ	1	3,559,595	3,595,000
					SFMBT2	1	3,825,402	3,921,158
					ITIH5	1	4,052,014	4,096,123
					ITIH2	1	4,053,533	4,127,906
					KIN	1	4,129,715	4,144,409
					ATP5C1	1	4,144,466	4,151,271
					TAF3	1	4,153,923	4,268,028
					GATA3	1	4,288,842	4,318,191
2	102,945,374	105,091,268	2,145,894	0.999789534				
					OSBPL1A	2	102,948,952	103,020,897
					IMPACT	2	103,033,304	103,051,363
					ZNF521	2	103,383,680	103,410,706
					SS18	2	103,745,620	103,788,130
					TAF4B	2	103,821,689	103,883,809
					KCTD1	2	103,913,222	103,977,131
					AQP4	2	104,100,790	104,109,628
					CHST9	2	104,126,305	104,127,381
					CDH2	2	104,449,184	104,562,821
5	36,000,903	38,160,604	2,159,701	0.999736096				
					SLC25A21	5	36,015,953	36,247,167
					MIPOL1	5	36,277,202	36,335,031
					TTC6	5	36,464,047	36,517,135
					SEC23A	5	36,843,282	36,865,890
					GEMIN2	5	36,871,372	36,880,109
					TRAPPC6B	5	36,880,151	36,883,970
					PNN	5	36,884,690	36,894,010
					FBXO33	5	36,932,268	36,950,848
					ZNF410	5	36,952,636	36,968,699
					FAM161B	5	36,969,905	36,977,054
					COQ6	5	36,978,464	36,985,973
					ENTPD5	5	36,989,049	37,003,244
					CCDC176	5	37,003,760	37,012,621
					ALDH6A1	5	37,016,493	37,025,856

					LIN52	5	37,025,663	37,064,969
					VSX2	5	37,105,886	37,125,352
					ABCD4	5	37,135,753	37,148,405
					SYNDIG1L	5	37,167,461	37,170,933
					ISCA2	5	37,203,490	37,204,559
					NPC2	5	37,207,826	37,210,000
					LTBP2	5	37,210,588	37,268,314
					KIAA0317	5	37,286,003	37,304,516
					FCF1	5	37,308,073	37,313,662
					YLPM1	5	37,315,712	37,349,907
					PROX2	5	37,363,125	37,365,789
					DLST	5	37,370,864	37,384,872
					RPS6KL1	5	37,387,519	37,391,470
					PGF	5	37,393,657	37,400,127
					EIF2B2	5	37,406,611	37,412,371
					MLH3	5	37,413,704	37,431,030
					ACYP1	5	37,431,802	37,432,761
					FAM164C	5	37,434,156	37,436,408
					NEK9	5	37,442,481	37,464,017
					TMED10	5	37,468,792	37,483,006
					FOS	5	37,501,649	37,502,995
					BATF	5	37,562,117	37,567,648
					FLVCR2	5	37,575,337	37,599,366
					C14orf1	5	37,607,278	37,608,782
					TTLL5	5	37,609,205	37,728,665
					TGFB3	5	37,739,714	37,747,666
					IFT43	5	37,756,375	37,800,040
					C14ORF118	5	37,839,126	37,870,137
					ESRRB	5	37,917,633	38,040,716
					VASH1	5	38,144,496	38,157,153
					SS1R	5	36,632,383	36,633,663
					VRTN	5	37,158,585	37,161,565
3	96,762,582	98,960,307	2,197,725	0.999438844				
					E2F6	3	96,852,537	96,861,622
					GREB1	3	96,906,122	96,965,158
					LPIN1	3	96,971,362	97,044,067
					TRIB2	3	97,427,885	97,448,910
					FAM84A	3	98,116,394	98,118,503
					NBAS	3	98,343,307	98,504,175
					DDX1	3	98,505,120	98,526,381
					MYCN	3	98,778,414	98,780,948
9	20,542,558	22,750,594	2,208,036	0.999353302				
					SLITRK3	9	20,586,079	20,587,887
					OTOL1	9	21,401,027	21,404,693
					SPTSSB	9	21,417,214	21,425,710
					NMD3	9	21,436,129	21,447,358
					PPM1L	9	21,476,302	21,557,159
					KPNA4	9	21,597,000	21,610,445
					SMC4	9	21,626,305	21,657,730
					IFT80	9	21,661,987	21,703,750
					IL12A	9	21,727,352	21,729,152
					IQCJ	9	21,810,250	21,844,816
					MFSD1	9	21,872,165	21,885,523
					RARRES1	9	21,904,934	21,912,426
					GFM1	9	21,913,507	21,933,383
					LXN	9	21,919,321	21,923,817
					MLF1	9	21,936,183	21,948,704
					RSRC1	9	21,958,768	22,066,377
					VEPH1	9	22,173,837	22,221,855
					PTX3	9	22,180,190	22,186,891
					CCNL1	9	22,236,549	22,248,075
					LEKR1	9	22,281,614	22,298,405
					TIPARP	9	22,313,993	22,346,827
					SSR3	9	22,347,715	22,354,679

					GMPS	9	22,393,885	22,417,726
					SLC33A1	9	22,418,561	22,429,141
					C3orf33	9	22,430,628	22,434,432
					MME	9	22,527,165	22,563,563
					GPR149	9	22,627,534	22,648,732
					DHX36	9	22,652,324	22,670,920
					ARHGEF26	9	22,675,159	22,714,986
					B3GALNT1	9	21,566,727	21,567,710
					ARL14	9	21,572,455	21,573,027
3	77,406,587	79,690,855	2,284,268	0.997649019				
					TPBG	3	77,636,164	77,637,640
					IBTK	3	77,659,755	77,717,628
					FAM46A	3	77,839,636	77,873,063
					BCKDHB	3	78,457,902	78,570,667
					TTK	3	78,612,134	78,643,792
					ELOVL4	3	78,677,336	78,713,537
					SH3BGRL2	3	78,756,279	78,772,633
					LCA5	3	78,816,727	78,828,429
					HMGN3	3	78,877,354	78,901,407
					PHIP	3	78,926,379	79,016,040
					IRAK1BP1	3	79,040,637	79,051,664
					HTR1B	3	79,492,220	79,493,385
6	23,049,412	25,352,313	2,302,901	0.997000785				
						6	23,104,676	23,106,678
					PCGF6	6	23,110,411	23,132,990
					TAF5	6	23,133,668	23,144,709
					USMG5	6	23,144,647	23,148,454
					PDCD11	6	23,149,107	23,171,490
					CALHM2	6	23,175,699	23,177,288
					CALHM1	6	23,181,808	23,183,651
					CALHM3	6	23,187,673	23,189,859
					NEURL	6	23,201,517	23,337,251
					SH3PXD2A	6	23,349,107	23,406,139
					OBFC1	6	23,585,345	23,623,213
					SLK	6	23,623,564	23,666,255
					COL17A1	6	23,670,969	23,706,526
					SFR1	6	23,730,903	23,734,477
					WDR96	6	23,736,120	23,778,750
					CCDC147	6	23,826,509	23,881,678
					SORCS3	6	23,968,010	24,235,717
					SORCS1	6	24,664,740	24,940,362
					XPNPEP1	6	25,219,899	25,248,494
					ADD3	6	25,264,735	25,352,280
					ITPRIP	6	23,801,103	23,802,752
1	20,525,913	22,853,843	2,327,930	0.995309161				
					ZNF800	1	20,644,895	20,659,015
					GRM8	1	20,700,732	21,020,127
					POT1	1	21,513,823	21,575,759
					GPR37	1	21,614,383	21,629,956
					SPAM1	1	21,866,991	21,875,891
					WASL	1	21,980,330	22,030,621
					LMOD2	1	22,047,781	22,055,493
					ASB15	1	22,059,754	22,075,013
					NDUFA5	1	22,078,542	22,084,456
					IQUB	1	22,086,126	22,106,344
					SLC13A1	1	22,194,831	22,218,981
					CADPS2	1	22,359,460	22,601,087
					FEZF1	1	22,608,523	22,610,729
					AASS	1	22,638,791	22,669,004
					PTPRZ1	1	22,669,170	22,806,015
2	137,387,679	139,781,764	2,394,085	0.990042057				
					DERL1	2	137,483,314	137,498,444
					WDR67	2	137,498,743	137,523,642
					FAM83A	2	137,525,083	137,540,453

						ATAD2	2	137,586,976	137,621,986
						WDYHV1	2	137,622,325	137,632,958
						FBXO32	2	137,644,051	137,668,702
						KLHL38	2	137,696,848	137,701,773
						ANXA13	2	137,709,300	137,734,052
						FAM91A1	2	137,737,298	137,762,174
						FER1L6	2	137,800,984	137,872,216
						TMEM65	2	137,986,349	138,016,847
						TATDN1	2	138,058,219	138,071,047
						NDUFB9	2	138,071,064	138,074,938
						MTSS1	2	138,077,064	138,193,553
						SQLE	2	138,265,073	138,279,203
						KIAA0196	2	138,283,684	138,306,583
						NSMCE2	2	138,307,230	138,433,695
						TRIB1	2	138,436,990	138,444,511
						FAM84B	2	138,873,014	138,876,023
						MYC	2	139,316,928	139,321,894
						ZHX2	2	137,459,621	137,462,113
						ZHX1	2	137,565,618	137,568,239
						RNF139	2	138,055,501	138,057,312
	6	4,073,221	6,480,385	2,407,164	0.988871328				
						RASGEF1A	6	4,196,805	4,240,235
						CSGALNACT2	6	4,248,637	4,262,880
						RET	6	4,283,164	4,363,396
						BMS1	6	4,549,290	4,570,790
						C6H10orf57	6	4,583,363	4,588,681
						ANXA11	6	4,642,948	4,662,218
						ECD	6	4,669,027	4,677,636
						FAM149B1	6	4,677,768	4,689,108
						DNAJC9	6	4,691,408	4,694,526
						TFAM	6	4,694,683	4,699,942
						UBE2D1	6	4,700,548	4,716,284
						CISD1	6	4,716,456	4,723,609
						IPMK	6	4,731,815	4,761,989
						PCDH15	6	5,764,830	6,063,170
						SIRT1	6	6,071,359	6,096,385
						DNAJC12	6	6,100,818	6,109,848
						LRRTM3	6	6,249,644	6,333,333
z		58,075,466	60,504,813	2,429,347	0.986119719				
	2	5,328,195	7,833,125	2,504,930	0.969881646				
						SCN5A	2	5,408,261	5,613,834
						EXOG	2	5,692,041	5,711,470
						ACVR2B	2	5,721,529	5,739,855
						XYLB	2	6,140,053	6,166,029
						NUB1	2	6,187,456	6,200,306
						WDR86	2	6,203,237	6,221,340
						CRYGN	2	6,235,658	6,239,901
						PRKAG2	2	6,247,476	6,461,068
						GALNT11	2	6,503,696	6,527,983
						MLL3	2	6,528,611	6,717,633
						XRCC2	2	6,755,237	6,767,527
						DPP6	2	7,411,564	7,635,852
						PAXIP1	2	7,658,648	7,692,640
						HTR5A	2	7,711,450	7,714,285
						INSIG1	2	7,827,461	7,832,977
	2	116,407,731	118,932,805	2,525,074	0.964614031				
						LACTB2	2	116,455,967	116,473,097
						XKR9	2	116,481,189	116,488,707
						EYA1	2	116,629,419	116,718,405
						MSC	2	116,915,841	116,918,080
						TRPA1	2	116,997,437	117,055,053
						KCNB2	2	117,126,579	117,312,904
						TERF1	2	117,328,695	117,352,082
						SBSPON	2	117,350,076	117,361,786

					RPL7	2	117,431,264	117,437,199
					RDH10	2	117,438,975	117,467,086
					STAU2	2	117,503,018	117,648,491
					UBE2W	2	117,680,876	117,713,123
					TMEM70	2	117,735,622	117,739,636
					LY96	2	117,745,310	117,753,197
					JPH1	2	117,820,324	117,897,580
					GDAP1	2	117,914,310	117,920,391
					PI15	2	118,088,996	118,111,354
					CRISPLD1	2	118,186,852	118,223,238
					HNF4G	2	118,421,432	118,442,821
11	3,163,524	5,711,059	2,547,535	0.956831177				
					MMP2	11	3,182,494	3,220,410
					IRX5	11	3,356,406	3,358,310
					FTO	11	4,129,194	4,353,272
					RPGRIP1L	11	4,357,663	4,419,746
					AKTIP	11	4,456,387	4,469,147
					RBL2	11	4,474,789	4,490,821
					CHD9	11	4,503,008	4,563,980
					TOX3	11	4,752,569	4,824,785
					SALL1	11	5,418,071	5,433,815
					CYLD	11	5,636,333	5,654,522
					SNX20	11	5,663,396	5,670,064
1	164,506,093	167,067,658	2,561,565	0.951786742				
					OLFM4	1	164,871,751	164,897,395
					PCDH8	1	164,995,573	165,000,858
					LECT1	1	165,018,166	165,029,891
					SUGT1	1	165,034,517	165,057,072
					ELF1	1	165,058,991	165,145,844
					WBP4	1	165,145,975	165,167,892
					MTRF1	1	165,172,543	165,185,717
					NAA16	1	165,191,023	165,255,094
					RGCC	1	165,264,942	165,276,995
					VWA8	1	165,302,186	165,480,795
					DGKH	1	165,513,387	165,674,975
					AKAP11	1	165,702,876	165,738,647
					TNFSF11	1	165,817,349	165,839,120
					EPSTI1	1	165,967,144	166,019,257
					DNAJC15	1	166,031,302	166,055,788
					ENOX1	1	166,088,091	166,385,249
					CCDC122	1	166,476,400	166,486,181
					LACC1	1	166,492,231	166,512,495
					TSC22D1	1	166,717,963	166,796,518
					NUFIP1	1	166,904,655	166,927,469
					KIAA1704	1	166,927,078	166,942,984
					GTF2F2	1	166,965,970	167,057,679
					TPT1	1	167,058,420	167,066,619
					KCTD4	1	166,995,127	166,995,900
7	14,087,975	16,675,492	2,587,517	0.944734966				
					CWC22	7	14,281,313	14,312,291
					ZNF385B	7	14,343,682	14,477,554
					SESTD1	7	14,533,688	14,570,925
					CCDC141	7	14,582,883	14,641,810
					PLEKHA3	7	14,887,747	14,903,030
					FKBP7	7	14,902,995	14,907,636
					DFNB59	7	14,909,332	14,917,988
					OSBPL6	7	14,935,828	14,977,668
					RBM45	7	15,031,727	15,042,794
					PDE11A	7	15,051,870	15,174,859
					AGPS	7	15,219,971	15,271,809
					NFE2L2	7	15,304,546	15,320,356
					HNRNPA3	7	15,327,805	15,341,998
					MTX2	7	15,709,764	15,742,887
					HOXD3	7	15,763,092	15,765,488

					HOXD4	7	15,777,579	15,779,976
					HOXD8	7	15,792,590	15,820,406
					HOXD9	7	15,798,954	15,800,275
					HOXD10	7	15,804,061	15,806,437
					HOXD11	7	15,814,096	15,815,924
					HOXD12	7	15,820,669	15,822,513
					HOXD13	7	15,827,616	15,828,537
					EVX2	7	15,833,129	15,835,917
					KIAA1715	7	15,868,345	15,908,061
					ATP5G3	7	16,150,149	16,153,215
					ATF2	7	16,159,461	16,206,145
					CHN1	7	16,219,611	16,299,658
					CHRNA1	7	16,305,300	16,310,101
					WIPF1	7	16,313,906	16,361,739
					GPR155	7	16,380,664	16,402,658
					SCRN3	7	16,404,276	16,412,287
					CIR1	7	16,412,437	16,433,377
					OLA1	7	16,463,674	16,548,896
					SP3	7	16,580,306	16,610,757
7	30,893,512	33,494,400	2,600,888	0.939369771				
					SPOPL	7	30,896,072	30,910,605
					NXPH2	7	30,928,491	30,961,286
					KYNU	7	32,143,918	32,202,554
					ARHGAP15	7	32,211,979	32,530,918
					GTDC1	7	32,617,757	32,782,028
					ZEB2	7	32,812,269	32,916,777
9	15,666,220	18,477,568	2,811,348	0.813025862				
					EIF4A2	9	15,671,724	15,678,579
					RFC4	9	15,678,911	15,690,512
					MCF2L2	9	15,742,979	15,839,708
					LAMP3	9	15,845,910	15,857,555
					MCCC1	9	15,864,983	15,880,897
					DCUN1D1	9	15,894,430	15,904,156
					ATP11B	9	15,908,775	16,005,533
					DNAJC19	9	16,559,670	16,562,902
					FXR1	9	16,565,596	16,587,667
					CCDC39	9	16,646,100	16,662,762
					TTC14	9	16,663,283	16,672,397
					PEX5L	9	16,760,382	16,850,020
					USP13	9	16,861,502	16,902,461
					NDUFB5	9	16,903,953	16,907,474
					MRPL47	9	16,907,572	16,911,041
					ACTL6A	9	16,911,951	16,921,482
					GNB4	9	16,972,459	16,979,703
					MFN1	9	16,984,938	17,003,984
					ZNF639	9	17,007,571	17,011,621
					PIK3CA	9	17,020,759	17,042,366
					ZMAT3	9	17,052,618	17,062,023
					NAALADL2	9	17,795,977	17,991,569
					NLGN1	9	18,196,758	18,471,834
					B3GNT5	9	15,777,289	15,778,413
					SOX2	9	16,342,563	16,343,510
2	15,286,481	18,178,069	2,891,588	0.742484995				
					MPP7	2	15,410,320	15,499,606
					ARMC4	2	15,517,575	15,589,648
					MKX	2	15,606,690	15,649,798
					RAB18	2	15,704,613	15,717,690
					YME1L1	2	15,807,104	15,827,193
					MASTL	2	15,830,069	15,845,780
					ACBD5	2	15,848,492	15,875,313
					ABI1	2	15,897,343	15,947,115
					PDSS1	2	15,977,931	15,999,697
					APBB1IP	2	16,026,711	16,084,527
					GAD2	2	16,147,743	16,180,423

					MYO3A	2	16,182,205	16,276,588
					GPR179	2	16,372,423	16,557,109
					ENKUR	2	16,613,396	16,622,552
					PRTFDC1	2	16,631,918	16,676,613
					ARHGAP21	2	16,677,325	16,817,876
					KIAA1217	2	16,817,533	17,151,976
					OTUD1	2	17,237,193	17,240,043
					C10orf67	2	17,250,380	17,288,284
					PTF1A	2	17,298,877	17,299,386
					ARMC3	2	17,331,978	17,388,781
					PIP4K2A	2	17,424,993	17,525,165
					SPAG6	2	17,577,501	17,600,696
					COMMD3	2	17,625,279	17,653,946
					DNAJC1	2	17,662,537	17,798,689
					MLLT10	2	17,799,470	17,915,028
						2	17,930,605	17,933,285
					THNSL1	2	16,606,746	16,608,950
6	26,136,406	29,922,314	3,785,908	0.142564578				
					GPAM	6	26,158,125	26,183,815
					TECTB	6	26,215,254	26,223,330
					ACSL5	6	26,247,834	26,264,411
					ZDHHC6	6	26,264,533	26,277,485
					VTI1A	6	26,277,903	26,526,700
					TCF7L2	6	26,590,486	26,762,220
					HABP2	6	26,971,704	26,991,533
					NRAP	6	26,991,279	27,037,248
					CASP7	6	27,042,298	27,062,965
					PLEKHS1	6	27,064,759	27,081,909
					DCLRE1A	6	27,087,946	27,102,495
					NHLRC2	6	27,103,400	27,134,851
					C10ORF118	6	27,217,490	27,246,216
					TDRD1	6	27,245,682	27,267,251
					COL9A3	6	27,267,818	27,285,089
					AFAP1L2	6	27,287,975	27,335,710
					ABLIM1	6	27,359,430	27,532,008
					FAM160B1	6	27,536,767	27,571,562
					TRUB1	6	27,594,383	27,622,142
					ATRNL1	6	27,680,834	28,014,799
					GFRA1	6	28,152,303	28,288,081
					CCDC172	6	28,309,882	28,328,718
					HSPA12A	6	28,413,790	28,450,155
					ENO4	6	28,500,759	28,518,682
					KIAA1598	6	28,516,161	28,575,056
					KCNK18	6	28,649,189	28,653,448
					PDZD8	6	28,696,248	28,747,446
					EMX2	6	28,814,903	28,820,582
					RAB11FIP2	6	29,003,777	29,047,647
					FAM204A	6	29,173,024	29,190,310
					C6H10ORF46	6	29,348,791	29,388,352
					EIF3A	6	29,508,820	29,537,182
					FAM45A	6	29,538,376	29,547,379
					SFXN4	6	29,550,386	29,559,570
					PRDX3	6	29,560,387	29,565,670
					GRK5	6	29,572,855	29,771,556
					RGS10	6	29,757,675	29,767,309
					TIAL1	6	29,782,065	29,799,900
					BAG3	6	29,813,247	29,832,893
					INPP5F	6	29,851,879	29,889,013
					MCMBP	6	29,884,934	29,905,383
					SEC23IP	6	29,905,638	29,921,730
					ADRB1	6	27,190,009	27,191,226
					NANOS1	6	29,504,247	29,505,285
2	9,608,529	13,747,682	4,139,153	0.061591934				
					VPAC2	2	9,612,723	9,663,652

					ZMYND11	2	9,791,128	9,894,465
					DIP2C	2	9,899,531	9,999,771
					LARP4B	2	10,249,042	10,281,209
					GTPBP4	2	10,356,114	10,367,015
					WDR37	2	10,386,560	10,413,702
					ADARB2	2	10,438,685	10,736,438
					PFKP	2	11,354,185	11,395,733
					PITRM1	2	11,396,672	11,421,465
					GJD4	2	12,778,766	12,787,894
					CCNY	2	12,793,547	12,910,204
					CREM	2	12,930,882	12,960,732
					CUL2	2	12,972,707	13,022,987
					PARD3	2	13,068,736	13,498,548
					FZD8	2	12,761,773	12,762,399
1	132,848,000	137,310,640	4,462,640	0.028047615				
					RFX8	1	132,861,202	132,895,021
					MAP4K4	1	132,936,573	133,084,109
					IL1R2	1	133,109,396	133,119,021
					IL1RL1	1	133,227,061	133,252,726
					IL18R1	1	133,259,701	133,278,851
					IL18RAP	1	133,283,645	133,298,556
					SLC9A4	1	133,300,715	133,329,117
					SLC9A2	1	133,354,849	133,381,741
					MFSD9	1	133,391,984	133,402,160
					TMEM182	1	133,408,227	133,435,369
					MRPS9	1	134,393,340	134,425,489
					TGFBRAP1	1	134,450,449	134,476,912
					C1H2ORF49	1	134,481,595	134,496,274
					FHL2	1	134,496,864	134,536,397
					NCK2	1	134,633,927	134,714,716
					C2ORF40	1	134,771,683	134,776,149
					ST6GAL2	1	135,007,544	135,053,400
					SLC5A7	1	135,397,400	135,423,764
					SULT1C	1	135,521,670	135,527,319
					RANBP2	1	135,763,887	135,793,418
					EDAR	1	135,819,209	135,882,851
					UPF3A	1	136,254,098	136,279,867
					CDC16	1	136,281,362	136,301,395
					RASA3	1	136,345,769	136,476,626
					GAS6	1	136,509,160	136,547,639
					FAM70B	1	136,551,868	136,614,965
					ATP4B	1	136,636,254	136,641,973
					TMCO3	1	136,694,300	136,726,217
					DCUN1D2	1	136,728,468	136,751,599
					ADPRHL1	1	136,753,731	136,773,443
					GRTP1	1	136,801,888	136,830,896
					LAMP1	1	136,831,179	136,848,516
					CUL4A	1	136,854,216	136,888,547
					PCID2	1	136,889,042	136,902,069
					PROZ	1	136,903,332	136,910,194
					F10	1	136,922,630	136,935,187
					F7	1	136,938,052	136,946,321
					MCF2L	1	136,949,339	137,097,962
					ATP11A	1	137,110,630	137,228,603
					SOWAHC	1	136,196,812	136,198,516
					CHAMP1	1	136,241,415	136,244,261

Supplementary table 11c. Multispecies homologous synteny blocks (msHSBs) detected in archosaurians and testudines

Chr	msHSB start (bp)	msHSB end (bp)	msHSB length (bp)	Probability of being found under the Poisson process	Gene ID	Chr	Gene start (bp)	Gene end (bp)
1	10,916,110	12,431,817	1,515,707	1				
					CD36	1	11,005,425	11,038,504
					GNAT3	1	11,086,584	11,113,407
					GNAI3	1	11,190,979	11,223,596
					MAGI2	1	11,930,694	12,230,824
					PHTF2	1	12,251,576	12,314,104
					TMEM60	1	12,314,478	12,318,704
					RSBN1L	1	12,327,050	12,358,827
					PTPN18	1	12,381,899	12,422,097
2	8,014,363	9,563,687	1,549,324	1				
					SHH	2	8,080,091	8,089,855
					RNF32	2	8,414,446	8,427,357
					LMBR1	2	8,434,744	8,494,298
					NOM1	2	8,513,775	8,529,642
					MNX1	2	8,538,868	8,541,508
					UBE3C	2	8,582,314	8,644,842
					PTPRN2	2	8,785,742	9,406,733
					NCAPG2	2	9,417,857	9,456,238
					ESYT2	2	9,467,167	9,532,244
3	99,384,303	100,950,258	1,565,955	1				
					VSNL1	3	99,739,837	99,814,889
					SMC6	3	99,815,077	99,852,120
					GEN1	3	99,853,931	99,870,493
					KCNS3	3	99,922,984	99,942,855
					RDH14	3	100,213,340	100,217,996
					OSR1	3	100,695,798	100,697,672
					MSGN1	3	99,887,826	99,889,127
1	97,365,488	98,957,585	1,592,097	1				
					HSPA13	1	97,411,085	97,418,694
					SAMSN1	1	97,445,928	97,480,511
					USP25	1	97,936,529	98,030,131
					CXADR	1	98,638,187	98,668,892
					C1H21ORF91	1	98,700,854	98,720,026
					CHODL	1	98,864,912	98,893,667
					TMPRSS15	1	98,898,052	98,906,375
					NRIP1	1	97,651,374	97,654,841
1	190,170,291	191,772,697	1,602,406	1				
					TENM4	1	191,230,962	191,733,590
4	86,080,977	87,690,044	1,609,067	1				
					LRRTM1	4	87,094,015	87,096,026
Z	51,655,572	53,283,058	1,627,486	1				
4	73,572,127	75,214,999	1,642,872	1				
					PPARGC1A	4	73,626,292	73,691,855
					GPR125	4	74,015,279	74,072,320
					KCNIP4	4	74,436,584	74,527,405
					PACRGL	4	74,526,045	74,539,587
					SLIT2	4	74,557,149	74,802,699
2	1,656,608	3,303,402	1,646,794	1				
					VIPR1	2	1,722,123	1,826,646
					SEC22C	2	1,886,499	1,900,914
					NKTR	2	1,927,422	1,965,795
					ZBTB47	2	1,978,851	1,993,362
					KBTBD5	2	1,999,758	2,009,597
					HHATL	2	2,014,408	2,026,554
					CCDC13	2	2,032,322	2,059,811
					HIGD1C	2	2,061,655	2,066,620
					CCBP2	2	2,072,000	2,073,926
					OBSCN	2	2,080,439	2,253,956
					C2H1ORF69	2	2,295,676	2,300,126
					GJC2	2	2,303,738	2,310,415
					GUK1	2	2,334,607	2,343,799
					MRPL55	2	2,348,731	2,351,041

					C1ORF35	2	2,351,366	2,358,790
					ARF1	2	2,357,324	2,369,250
					WNT3A	2	2,459,534	2,542,486
					WNT9A	2	2,573,612	2,624,503
					SNAP47	2	3,185,315	3,205,912
					JMJD4	2	3,211,699	3,220,712
					ALS2CL	2	3,216,850	3,246,773
					TMIE	2	3,268,001	3,297,394
3	24,475,771	26,122,614	1,646,843	1				
					PLEKHH2	3	24,491,110	24,545,482
					DYNC2LI1	3	24,548,141	24,570,676
					ABCG5	3	24,567,546	24,588,926
					ABCG8	3	24,585,792	24,598,091
					LRPPRC	3	24,608,804	24,695,385
					PPM1B	3	24,761,763	24,820,317
					SLC3A1	3	24,827,681	24,841,984
					PREPL	3	24,831,731	24,859,714
					SIX2	3	25,240,527	25,243,491
					SRBD1	3	25,432,172	25,549,520
					PRKCE	3	25,576,043	25,856,010
					EPAS1	3	25,951,104	25,982,708
					RHOQ	3	26,075,656	26,091,343
					PIGF	3	26,084,410	26,110,103
					CRIPT	3	26,110,349	26,115,765
1	109,932,747	111,600,312	1,667,565	1				
					CXorf36	1	110,128,634	110,143,679
					FUNDC1	1	110,347,436	110,362,439
					EFHC2	1	110,382,985	110,442,596
					NDP	1	110,522,886	110,531,383
					MAOB	1	110,555,374	110,609,289
					MAOA	1	110,613,430	110,658,911
					GPR34	1	111,232,706	111,235,607
					NYX	1	111,334,091	111,336,368
					GPR82	1	111,219,959	111,220,993
5	33,186,512	34,867,480	1,680,968	0.999999999				
					KIAA1333	5	33,413,582	33,433,671
					SCFD1	5	33,438,355	33,483,021
					COCH	5	33,517,885	33,534,657
					AP4S1	5	33,603,694	33,613,992
					HECTD1	5	33,616,118	33,666,611
					HEATR5A	5	33,685,490	33,736,833
					NUBPL	5	33,774,090	33,856,331
					ARHGAP5	5	33,958,351	33,993,431
					AKAP6	5	34,050,021	34,304,671
5	30,029,578	31,712,070	1,682,492	0.999999999				
					MEIS2	5	30,506,655	30,675,196
					C5H15orf41	5	30,717,442	30,832,671
					ATPBD4	5	31,253,451	31,438,936
					ZNF770	5	31,468,292	31,472,971
					AQR	5	31,481,161	31,528,089
					ACTC1	5	31,546,121	31,552,902
					GJD2	5	31,568,439	31,572,498
1	80,304,616	82,024,763	1,720,147	0.999999994				
					LSAMP	1	81,373,015	81,656,000
					GAP43	1	81,680,485	81,734,254
5	36,433,461	38,160,604	1,727,143	0.999999993				
					TTC6	5	36,464,047	36,517,135
					SEC23A	5	36,843,282	36,865,890
					GEMIN2	5	36,871,372	36,880,109
					TRAPPC6B	5	36,880,151	36,883,970
					PNN	5	36,884,690	36,894,010
					FBXO33	5	36,932,268	36,950,848
					ZNF410	5	36,952,636	36,968,699
					FAM161B	5	36,969,905	36,977,054
					COQ6	5	36,978,464	36,985,973
					ENTPD5	5	36,989,049	37,003,244
					CCDC176	5	37,003,760	37,012,621

					ALDH6A1	5	37,016,493	37,025,856
					LIN52	5	37,025,663	37,064,969
					VSX2	5	37,105,886	37,125,352
					ABCD4	5	37,135,753	37,148,405
					SYNDIG1L	5	37,167,461	37,170,933
					ISCA2	5	37,203,490	37,204,559
					NPC2	5	37,207,826	37,210,000
					LTBP2	5	37,210,588	37,268,314
					KIAA0317	5	37,286,003	37,304,516
					FCF1	5	37,308,073	37,313,662
					YLPM1	5	37,315,712	37,349,907
					PROX2	5	37,363,125	37,365,789
					DLST	5	37,370,864	37,384,872
					RPS6KL1	5	37,387,519	37,391,470
					PGF	5	37,393,657	37,400,127
					EIF2B2	5	37,406,611	37,412,371
					MLH3	5	37,413,704	37,431,030
					ACYP1	5	37,431,802	37,432,761
					FAM164C	5	37,434,156	37,436,408
					NEK9	5	37,442,481	37,464,017
					TMED10	5	37,468,792	37,483,006
					FOS	5	37,501,649	37,502,995
					BATF	5	37,562,117	37,567,648
					FLVCR2	5	37,575,337	37,599,366
					C14orf1	5	37,607,278	37,608,782
					TTLL5	5	37,609,205	37,728,665
					TGFB3	5	37,739,714	37,747,666
					IFT43	5	37,756,375	37,800,040
					C14ORF118	5	37,839,126	37,870,137
					ESRRB	5	37,917,633	38,040,716
					VASH1	5	38,144,496	38,157,153
					SS1R	5	36,632,383	36,633,663
					VRTN	5	37,158,585	37,161,565
2	133,158,961	134,886,969	1,728,008	0.999999992				
					TRPS1	2	134,048,833	134,252,747
					EIF3H	2	134,628,301	134,709,262
					UTP23	2	134,720,381	134,723,977
					RAD21	2	134,749,874	134,773,800
17	2,877,535	4,606,475	1,728,940	0.999999991				
					PAPPA	17	3,040,970	3,209,266
					ASTN2	17	3,219,825	3,482,002
					TLR4	17	3,566,454	3,571,907
					DBC1	17	3,985,882	4,064,290
					CDK5RAP2	17	4,495,102	4,566,562
					MEGF9	17	4,575,673	4,590,722
5	10,087,655	11,834,285	1,746,630	0.999999983				
					SOX6	5	10,549,851	10,784,968
					C5H11ORF58	5	10,948,695	10,953,363
					PLEKHA7	5	10,959,448	11,057,508
					RPS13	5	11,136,190	11,139,937
					PIK3C2A	5	11,142,129	11,205,960
					NUCB2	5	11,205,875	11,231,516
					KCNJ11	5	11,247,848	11,250,065
					ABCC8	5	11,253,945	11,319,342
					USH1C	5	11,334,156	11,377,576
					OTOG	5	11,381,876	11,476,414
					MYOD1	5	11,578,793	11,582,278
					KCNC1	5	11,597,391	11,714,071
3	65,651,343	67,404,826	1,753,483	0.999999975				
					RPF2	3	65,709,193	65,721,929
					GTF3C6	3	65,721,376	65,726,791
					AMD1	3	65,744,041	65,761,269
					CDK19	3	65,801,524	65,899,585
					SLC22A16	3	65,931,799	65,962,885
					DDO	3	65,969,395	65,975,774
					C6ORF186	3	65,985,390	66,029,579
					CDC40	3	66,033,229	66,074,352

						WASF1	3	66,123,600	66,154,746
						GPR6	3	66,173,387	66,197,130
						FIG4	3	66,201,896	66,268,774
						AK9	3	66,269,578	66,325,155
						ZBTB24	3	66,327,020	66,334,932
						PPIL6	3	66,340,530	66,350,895
						CD164	3	66,357,144	66,365,037
						C6ORF183	3	66,413,334	66,432,788
						CEP57L1	3	66,460,394	66,473,222
						SESN1	3	66,547,545	66,556,746
						ARMC2	3	66,560,205	66,617,959
						FOXO3	3	66,702,483	66,716,393
						LACE1	3	66,809,734	66,870,599
						SNX3	3	66,876,089	66,895,012
						NR2E1	3	66,898,978	66,913,949
						OSTM1	3	66,950,856	66,961,817
						SEC63	3	66,978,001	67,033,488
						SCML4	3	67,042,180	67,098,929
						SOBP	3	67,134,086	67,243,457
						PDSS2	3	67,259,779	67,378,577
						BEND3	3	67,393,979	67,403,976
	4	20,823,966	22,596,657	1,772,691	0.999999943				
						GRIA2	4	20,873,025	20,960,836
						FAM198B	4	21,221,107	21,234,597
						TMEM144	4	21,242,338	21,257,193
						RXFP1	4	21,328,064	21,376,386
						ETFDH	4	21,379,598	21,396,998
						PPID	4	21,395,223	21,405,790
						FNIP2	4	21,425,883	21,457,823
						C4ORF45	4	21,455,357	21,496,063
						RAPGEF2	4	21,509,902	21,683,609
						FSTL5	4	22,250,827	22,516,765
z		27,683,328	29,468,950	1,785,622	0.999999909				
	2	120,455,577	122,248,807	1,793,230	0.999999853				
						STMN2	2	120,505,360	120,515,753
						HEY1	2	120,538,764	120,541,690
						TPD52	2	120,668,873	120,704,236
						ZBTB10	2	120,783,328	120,809,194
						ZNF704	2	120,881,814	120,900,516
						PAG1	2	120,988,402	121,062,954
						FABP5	2	121,148,557	121,153,615
						PMP2	2	121,215,806	121,220,355
						FABP4	2	121,225,723	121,228,944
						IMPA1	2	121,262,379	121,277,486
						ZFAND1	2	121,279,568	121,285,857
						CHMP4C	2	121,287,839	121,304,205
						SNX16	2	121,309,189	121,333,508
	3	35,427,107	37,224,095	1,796,988	0.999999835				
						GREM2	3	35,493,725	35,533,009
						FMN2	3	35,540,378	35,680,173
						CHRM3	3	35,727,538	35,877,494
						RYR2	3	36,498,791	36,661,068
						MTR	3	36,890,573	36,933,337
						ACTN2	3	36,948,211	37,014,188
						HEATR1	3	37,025,129	37,060,227
						LGALS8	3	37,061,029	37,073,707
						EDARADD	3	37,078,799	37,097,401
						ERO1LB	3	37,097,636	37,134,158
						GPR137B	3	37,135,755	37,159,463
						NID1	3	37,178,196	37,213,970
	3	93,840,831	95,639,533	1,798,702	0.999999826				
						CMPK2	3	94,575,875	94,584,183
						RSAD2	3	94,589,239	94,594,293
						RNF144A	3	94,613,864	94,661,730
						ID2	3	95,424,295	95,426,233
						KIDINS220	3	95,445,100	95,500,211
						MBOAT2	3	95,513,204	95,603,900

					SOX11	3	94,137,728	94,138,918
4	23,185,693	25,022,794	1,837,101	0.999999241				
					TMEM192	4	23,236,487	23,257,108
					KLHL2	4	23,265,508	23,325,234
					SC4MOL	4	23,326,894	23,333,143
					CPE	4	23,336,496	23,382,106
					TLL1	4	23,520,463	23,656,666
					SPOCK3	4	23,947,374	24,051,813
					ANXA10	4	24,400,386	24,416,023
					PALLD	4	24,486,948	24,675,736
					AADAT	4	24,691,532	24,707,952
					MFAP3L	4	24,743,867	24,747,796
					C4ORF27	4	24,796,835	24,803,472
					CLCN3	4	24,804,714	24,864,749
					NEK1	4	24,866,404	24,909,512
					CBR4	4	25,012,882	25,021,145
3	58,723,180	60,639,979	1,916,799	0.99998909				
					RSPO3	3	58,726,123	58,785,427
					CENPW	3	59,060,255	59,067,195
					TRMT11	3	59,178,746	59,205,181
					HINT3	3	59,212,941	59,219,743
					NCOA7	3	59,223,034	59,291,522
					HDDC2	3	59,516,400	59,525,907
					TPD52L1	3	59,527,859	59,580,356
					RNF217	3	59,606,826	59,662,599
					NKAIN2	3	59,710,415	59,999,592
					TRDN	3	60,315,841	60,432,093
					CLVS2	3	60,548,727	60,598,139
3	81,161,816	83,102,150	1,940,334	0.99997852				
					RIMS1	3	81,337,238	81,638,081
					OGFRL1	3	81,808,877	81,818,348
					B3GAT2	3	81,940,693	81,962,704
					FAM135A	3	82,085,759	82,150,209
					COL9A1	3	82,230,623	82,296,804
					COL19A1	3	82,304,939	82,484,565
					LMBRD1	3	82,508,371	82,577,230
1	141,339,838	143,294,319	1,954,481	0.999964517				
					SLC10A2	1	141,914,133	141,925,460
					BIVM	1	142,024,553	142,041,190
					KDELC1	1	142,045,388	142,054,410
					C13ORF27	1	142,057,711	142,064,431
					METTL21C	1	142,092,496	142,099,273
					TPP2	1	142,097,596	142,147,887
					ITGBL1	1	142,614,261	142,752,083
					NALCN	1	142,765,240	142,969,863
					TMTC4	1	143,142,401	143,197,059
					A2LD1	1	143,230,157	143,230,654
3	45,173,549	47,136,044	1,962,495	0.999960457				
					UTRN	3	45,215,648	45,566,922
					EPM2A	3	45,769,623	45,811,385
					FBXO30	3	45,824,455	45,831,133
					SHPRH	3	45,868,769	45,921,436
					GRM1	3	45,937,157	46,118,743
					RAB32	3	46,154,328	46,173,538
					STXBP5	3	46,388,675	46,489,008
					SAMD5	3	46,522,593	46,647,011
					SASH1	3	46,930,247	47,049,931
					STX11	3	45,190,578	45,191,444
4	64,648,446	66,618,821	1,970,375	0.999952616				
					PDCL2	4	64,659,119	64,664,541
					CLOCK	4	64,696,007	64,717,393
					SRD5A3	4	64,724,586	64,735,325
					SRD5A3	4	64,738,412	64,744,643
					KDR	4	64,794,087	64,824,189
					KIT	4	64,878,556	64,930,842
					PDGFRA	4	65,003,747	65,031,783
					CHIC2	4	65,084,086	65,104,823

					LNX1	4	65,267,149	65,328,349
					FIP1L1	4	65,345,818	65,366,325
					SCFD2	4	65,537,526	65,550,782
					RASL11B	4	65,555,016	65,557,517
					USP46	4	65,597,260	65,618,172
					SPATA18	4	65,750,624	65,771,219
					SGCB	4	65,771,639	65,777,574
					DCUN1D4	4	65,794,330	65,821,389
					CWH43	4	65,834,288	65,859,538
					OCIAD1	4	65,878,670	65,892,164
					FRYL	4	65,919,831	66,048,744
					SLC10A4	4	66,054,693	66,056,492
					SLAIN2	4	66,066,500	66,094,716
					TEC	4	66,112,142	66,153,920
					TXK	4	66,159,821	66,172,212
					NIPAL1	4	66,175,121	66,186,188
					CNGA1	4	66,194,847	66,198,930
					NFXL1	4	66,200,755	66,242,146
					CORIN	4	66,262,035	66,367,775
					ATP10D	4	66,374,739	66,404,130
					COMMD8	4	66,422,820	66,429,658
					GABRB1	4	66,433,150	66,478,683
					GABRA4	4	66,540,864	66,576,459
1	148,706,739	150,681,323	1,974,584	0.999940077				
					SLITRK6	1	149,478,164	149,480,686
					SLITRK1	1	150,421,565	150,423,643
3	49,175,304	51,152,885	1,977,581	0.999941533				
					OPRM1	3	49,319,843	49,341,770
					SCAF8	3	49,604,475	49,635,861
					TFB1M	3	49,868,438	49,895,829
					CLDN20	3	49,875,601	49,880,776
					NOX3	3	49,915,188	49,952,437
					ARID1B	3	50,465,764	50,754,522
					TMEM242	3	50,810,196	50,829,319
					SNX9	3	50,997,690	51,052,016
					SYNJ2	3	51,063,895	51,127,835
3	56,602,089	58,590,234	1,988,145	0.999923859				
					CTGF	3	56,638,964	56,641,934
					ENPP1	3	56,656,447	56,707,127
					ENPP3	3	56,723,839	56,757,736
					MED23	3	56,762,370	56,797,379
					AKAP7	3	56,878,364	56,949,970
					EPB41L2	3	56,980,129	57,075,289
					SAMD3	3	57,322,882	57,363,225
					L3MBTL3	3	57,360,442	57,425,789
					C6ORF191	3	57,466,325	57,472,268
					ARHGAP18	3	57,506,532	57,565,989
					LAMA2	3	57,586,334	57,923,906
					PTPRK	3	58,029,862	58,422,557
					THEMIS	3	58,437,710	58,506,141
					C6orf58	3	58,527,005	58,539,770
					TMEM200A	3	57,238,296	57,239,765
10	13,555,367	15,569,180	2,013,813	0.999846769				
					SV2B	10	13,701,912	13,728,770
					SLCO3A1	10	13,838,315	13,953,221
					ST8SIA2	10	13,994,580	14,019,205
					FAM174B	10	14,039,364	14,055,885
					CHD2	10	14,112,774	14,149,142
					RGMA	10	14,166,477	14,183,190
					MCTP2	10	14,461,079	14,573,856
					NR2F2	10	15,328,163	15,333,861
2	141,261,815	143,287,606	2,025,791	0.999810429				
					PHF20L1	2	141,275,093	141,322,936
					TG	2	141,333,054	141,469,306
					SLA	2	141,410,203	141,432,079
					WISP1	2	141,532,750	141,540,007
					NDRG1	2	141,549,217	141,589,409

					ST3GAL1	2	141,665,331	141,689,879
					ZFAT	2	142,122,786	142,207,755
					KHDRBS3	2	142,700,486	142,776,708
10	7,607,999	9,634,830	2,026,831	0.999794192				
					UNC13C	10	7,641,038	7,759,221
					WDR72	10	7,807,793	7,874,460
					FAM214A	10	8,193,075	8,224,905
					ARPP19	10	8,227,077	8,235,366
					MYO5A	10	8,240,552	8,332,115
					MYO5C	10	8,334,755	8,365,618
					MAPK6	10	8,418,112	8,446,458
					LEO1	10	8,499,714	8,506,990
					TMOD3	10	8,510,697	8,533,995
					TMOD2	10	8,537,029	8,567,174
					LYSMD2	10	8,581,242	8,590,880
					SCG3	10	8,596,070	8,621,388
					AP4E1	10	8,625,187	8,643,899
					TNFAIP8L3	10	8,648,404	8,687,755
					CYP19A1	10	8,712,476	8,726,241
					GLDN	10	8,745,469	8,761,869
					DMXL2	10	8,763,459	8,813,670
					SEMA6D	10	9,404,673	9,419,492
					SLC24A5	10	9,545,024	9,553,009
					MYEF2	10	9,551,752	9,570,906
					SLC12A1	10	9,587,065	9,632,792
2	112,226,473	114,277,051	2,050,578	0.999673866				
					CA8	2	112,413,504	112,453,419
					RAB2A	2	112,517,303	112,558,232
					CHD7	2	112,612,964	112,699,976
					CLVS1	2	112,882,281	112,978,767
					NKAIN3	2	113,137,211	113,469,039
					GGH	2	113,490,054	113,501,161
					TTPA	2	113,502,155	113,517,713
					YTHDF3	2	113,543,309	113,551,391
					CYP7B1	2	114,093,263	114,114,645
2	125,160,507	127,250,892	2,090,385	0.999280073				
					TRIQK	2	125,273,982	125,333,683
					FAM92A1	2	125,604,624	125,621,196
					RBM12B	2	125,623,603	125,630,097
					TMEM67	2	125,632,568	125,659,384
					PDP1	2	125,683,595	125,690,722
					CDH17	2	125,754,667	125,783,019
					GEM	2	125,802,915	125,811,572
					RAD54B	2	125,844,098	125,903,394
					FSBP	2	125,877,035	125,887,013
					KIAA1429	2	125,925,066	125,952,171
					ESRP1	2	125,956,620	125,988,739
					DPY19L4	2	126,052,840	126,076,741
					INTS8	2	126,126,452	126,151,324
					CCNE2	2	126,151,226	126,161,848
					TP53INP1	2	126,170,346	126,183,360
					NDUFAF6	2	126,201,059	126,216,326
					PLEKHF2	2	126,225,096	126,240,839
					C8orf37	2	126,258,109	126,269,454
					MTERFD1	2	126,597,212	126,615,684
					PTDSS1	2	126,616,011	126,645,632
					SDC2	2	126,740,060	126,929,143
					MTDH	2	127,057,053	127,088,247
					LAPTM4B	2	127,102,614	127,165,676
					MATN2	2	127,183,885	127,242,408
2	85,709,755	87,814,090	2,104,335	0.999062003				
					SLC6A19	2	85,710,095	85,731,320
					SLC6A18	2	85,732,747	85,758,741
					TERT	2	85,762,002	85,791,468
					CLPTM1L	2	85,803,794	85,834,463
					LPCAT1	2	85,846,135	85,899,078
					NDUFS6	2	86,002,617	86,007,620

					IRX4	2	86,065,381	86,068,294
					IRX2	2	86,624,614	86,631,727
					IRX1	2	87,131,471	87,135,061
1	172,850,666	174,964,633	2,113,967	0.99883292				
					RFC3	1	173,144,179	173,161,079
					STARD13	1	173,241,712	173,493,625
					KL	1	173,504,524	173,551,941
					PDS5B	1	173,655,059	173,730,298
					N4BP2L1	1	173,808,326	173,820,489
					BRCA2	1	173,820,724	173,857,456
					ZAR1	1	173,859,596	173,861,411
					FRY	1	173,863,589	174,002,319
					B3GALTL	1	174,203,173	174,247,847
					HSPH1	1	174,262,982	174,286,406
					ALOX5AP	1	174,335,143	174,344,760
					USPL1	1	174,350,051	174,363,188
					KATNAL1	1	174,496,738	174,524,653
					UBL3	1	174,588,070	174,645,255
					SLC7A1	1	174,687,111	174,726,955
12	9,123,361	11,244,240	2,120,879	0.998698623				
					RPN1	12	9,149,093	9,156,192
					GATA2	12	9,185,919	9,200,490
					EEFSEC	12	9,379,038	9,490,404
					RUVBL1	12	9,495,555	9,513,271
					SEC61A1	12	9,527,460	9,536,843
					KBTBD12	12	9,552,454	9,577,601
					MGLL	12	9,598,736	9,655,915
					ABTB1	12	9,657,435	9,685,045
					PODXL2	12	9,689,545	9,717,481
					MCM2	12	9,725,752	9,737,075
					GPR175	12	9,738,594	9,754,460
					PLXNA1	12	9,887,588	9,971,748
					TXNRD3	12	10,366,876	10,387,808
					CHST13	12	10,449,680	10,473,797
					SLC41A3	12	10,475,439	10,492,889
					KLF15	12	10,614,316	10,626,926
					UROC1	12	10,667,219	10,697,052
					CHCHD4	12	10,756,017	10,764,587
					TMEM43	12	10,764,796	10,775,842
					XPC	12	10,776,979	10,787,331
					SLC6A6	12	10,950,982	10,995,867
					DNAJB8	12	9,245,863	9,246,974
2	102,945,374	105,091,268	2,145,894	0.99808235				
					OSBPL1A	2	102,948,952	103,020,897
					IMPACT	2	103,033,304	103,051,363
					ZNF521	2	103,383,680	103,410,706
					SS18	2	103,745,620	103,788,130
					TAF4B	2	103,821,689	103,883,809
					KCTD1	2	103,913,222	103,977,131
					AQP4	2	104,100,790	104,109,628
					CHST9	2	104,126,305	104,127,381
					CDH2	2	104,449,184	104,562,821
9	20,542,558	22,750,594	2,208,036	0.995412373				
					SLITRK3	9	20,586,079	20,587,887
					OTOL1	9	21,401,027	21,404,693
					SPTSSB	9	21,417,214	21,425,710
					NMD3	9	21,436,129	21,447,358
					PPM1L	9	21,476,302	21,557,159
					KPNA4	9	21,597,000	21,610,445
					SMC4	9	21,626,305	21,657,730
					IFT80	9	21,661,987	21,703,750
					IL12A	9	21,727,352	21,729,152
					IQCJ	9	21,810,250	21,844,816
					MFSD1	9	21,872,165	21,885,523
					RARRES1	9	21,904,934	21,912,426
					GFM1	9	21,913,507	21,933,383
					LXN	9	21,919,321	21,923,817

					MLF1	9	21,936,183	21,948,704
					RSRC1	9	21,958,768	22,066,377
					VEPH1	9	22,173,837	22,221,855
					PTX3	9	22,180,190	22,186,891
					CCNL1	9	22,236,549	22,248,075
					LEKR1	9	22,281,614	22,298,405
					TIPARP	9	22,313,993	22,346,827
					SSR3	9	22,347,715	22,354,679
					GMPS	9	22,393,885	22,417,726
					SLC33A1	9	22,418,561	22,429,141
					C3orf33	9	22,430,628	22,434,432
					MME	9	22,527,165	22,563,563
					GPR149	9	22,627,534	22,648,732
					DHX36	9	22,652,324	22,670,920
					ARHGEF26	9	22,675,159	22,714,986
					B3GALNT1	9	21,566,727	21,567,710
					ARL14	9	21,572,455	21,573,027
3	77,406,587	79,690,855	2,284,268	0.98744063				
					TPBG	3	77,636,164	77,637,640
					IBTK	3	77,659,755	77,717,628
					FAM46A	3	77,839,636	77,873,063
					BCKDHB	3	78,457,902	78,570,667
					TTK	3	78,612,134	78,643,792
					ELOVL4	3	78,677,336	78,713,537
					SH3BGRL2	3	78,756,279	78,772,633
					LCA5	3	78,816,727	78,828,429
					HMGN3	3	78,877,354	78,901,407
					PHIP	3	78,926,379	79,016,040
					IRAK1BP1	3	79,040,637	79,051,664
					HTR1B	3	79,492,220	79,493,385
6	23,049,412	25,352,313	2,302,901	0.984897917				
						6	23,104,676	23,106,678
					PCGF6	6	23,110,411	23,132,990
					TAF5	6	23,133,668	23,144,709
					USMG5	6	23,144,647	23,148,454
					PDCD11	6	23,149,107	23,171,490
					CALHM2	6	23,175,699	23,177,288
					CALHM1	6	23,181,808	23,183,651
					CALHM3	6	23,187,673	23,189,859
					NEURL	6	23,201,517	23,337,251
					SH3PXD2A	6	23,349,107	23,406,139
					OBFC1	6	23,585,345	23,623,213
					SLK	6	23,623,564	23,666,255
					COL17A1	6	23,670,969	23,706,526
					SFR1	6	23,730,903	23,734,477
					WDR96	6	23,736,120	23,778,750
					CCDC147	6	23,826,509	23,881,678
					SORCS3	6	23,968,010	24,235,717
					SORCS1	6	24,664,740	24,940,362
					XPNPEP1	6	25,219,899	25,248,494
					ADD3	6	25,264,735	25,352,280
					ITPRIP	6	23,801,103	23,802,752
1	20,525,913	22,853,843	2,327,930	0.978534795				
					ZNF800	1	20,644,895	20,659,015
					GRM8	1	20,700,732	21,020,127
					POT1	1	21,513,823	21,575,759
					GPR37	1	21,614,383	21,629,956
					SPAM1	1	21,866,991	21,875,891
					WASL	1	21,980,330	22,030,621
					LMOD2	1	22,047,781	22,055,493
					ASB15	1	22,059,754	22,075,013
					NDUFA5	1	22,078,542	22,084,456
					IQUB	1	22,086,126	22,106,344
					SLC13A1	1	22,194,831	22,218,981
					CADPS2	1	22,359,460	22,601,087
					FEZF1	1	22,608,523	22,610,729
					AASS	1	22,638,791	22,669,004

						PTPRZ1	1	22,669,170	22,806,015
	2	137,387,679	139,781,764	2,394,085	0.962117086				
						DERL1	2	137,483,314	137,498,444
						WDR67	2	137,498,743	137,523,642
						FAM83A	2	137,525,083	137,540,453
						ATAD2	2	137,586,976	137,621,986
						WDYHV1	2	137,622,325	137,632,958
						FBXO32	2	137,644,051	137,668,702
						KLHL38	2	137,696,848	137,701,773
						ANXA13	2	137,709,300	137,734,052
						FAM91A1	2	137,737,298	137,762,174
						FER1L6	2	137,800,984	137,872,216
						TMEM65	2	137,986,349	138,016,847
						TATDN1	2	138,058,219	138,071,047
						NDUFB9	2	138,071,064	138,074,938
						MTSS1	2	138,077,064	138,193,553
						SQLE	2	138,265,073	138,279,203
						KIAA0196	2	138,283,684	138,306,583
						NSMCE2	2	138,307,230	138,433,695
						TRIB1	2	138,436,990	138,444,511
						FAM84B	2	138,873,014	138,876,023
						MYC	2	139,316,928	139,321,894
						ZHX2	2	137,459,621	137,462,113
						ZHX1	2	137,565,618	137,568,239
						RNF139	2	138,055,501	138,057,312
z		58,080,322	60,504,813	2,424,491	0.953389283				
	7	30,893,512	33,391,484	2,497,972	0.919786969				
						SPOPL	7	30,896,072	30,910,605
						NXPH2	7	30,928,491	30,961,286
						KYNU	7	32,143,918	32,202,554
						ARHGAP15	7	32,211,979	32,530,918
						GTDC1	7	32,617,757	32,782,028
						ZEB2	7	32,812,269	32,916,777
	2	5,328,195	7,833,125	2,504,930	0.912983871				
						SCN5A	2	5,408,261	5,613,834
						EXOG	2	5,692,041	5,711,470
						ACVR2B	2	5,721,529	5,739,855
						XYLB	2	6,140,053	6,166,029
						NUB1	2	6,187,456	6,200,306
						WDR86	2	6,203,237	6,221,340
						CRYGN	2	6,235,658	6,239,901
						PRKAG2	2	6,247,476	6,461,068
						GALNT11	2	6,503,696	6,527,983
						MLL3	2	6,528,611	6,717,633
						XRCC2	2	6,755,237	6,767,527
						DPP6	2	7,411,564	7,635,852
						PAXIP1	2	7,658,648	7,692,640
						HTR5A	2	7,711,450	7,714,285
						INSIG1	2	7,827,461	7,832,977
	2	116,407,731	118,932,805	2,525,074	0.902019617				
						LACTB2	2	116,455,967	116,473,097
						XKR9	2	116,481,189	116,488,707
						EYA1	2	116,629,419	116,718,405
						MSC	2	116,915,841	116,918,080
						TRPA1	2	116,997,437	117,055,053
						KCNB2	2	117,126,579	117,312,904
						TERF1	2	117,328,695	117,352,082
						SBSPON	2	117,350,076	117,361,786
						RPL7	2	117,431,264	117,437,199
						RDH10	2	117,438,975	117,467,086
						STAU2	2	117,503,018	117,648,491
						UBE2W	2	117,680,876	117,713,123
						TMEM70	2	117,735,622	117,739,636
						LY96	2	117,745,310	117,753,197
						JPH1	2	117,820,324	117,897,580
						GDAP1	2	117,914,310	117,920,391
						PI15	2	118,088,996	118,111,354

					CRISPLD1	2	118,186,852	118,223,238
					HNF4G	2	118,421,432	118,442,821
11	3,163,524	5,711,059	2,547,535	0.88630018				
					MMP2	11	3,182,494	3,220,410
					IRX5	11	3,356,406	3,358,310
					FTO	11	4,129,194	4,353,272
					RPGRIP1L	11	4,357,663	4,419,746
					AKTIP	11	4,456,387	4,469,147
					RBL2	11	4,474,789	4,490,821
					CHD9	11	4,503,008	4,563,980
					TOX3	11	4,752,569	4,824,785
					SALL1	11	5,418,071	5,433,815
					CYLD	11	5,636,333	5,654,522
					SNX20	11	5,663,396	5,670,064
1	164,506,093	167,067,658	2,561,565	0.876633035				
					OLFM4	1	164,871,751	164,897,395
					PCDH8	1	164,995,573	165,000,858
					LECT1	1	165,018,166	165,029,891
					SUGT1	1	165,034,517	165,057,072
					ELF1	1	165,058,991	165,145,844
					WBP4	1	165,145,975	165,167,892
					MTRF1	1	165,172,543	165,185,717
					NAA16	1	165,191,023	165,255,094
					RGCC	1	165,264,942	165,276,995
					VWA8	1	165,302,186	165,480,795
					DGKH	1	165,513,387	165,674,975
					AKAP11	1	165,702,876	165,738,647
					TNFSF11	1	165,817,349	165,839,120
					EPSTI1	1	165,967,144	166,019,257
					DNAJC15	1	166,031,302	166,055,788
					ENOX1	1	166,088,091	166,385,249
					CCDC122	1	166,476,400	166,486,181
					LACC1	1	166,492,231	166,512,495
					TSC22D1	1	166,717,963	166,796,518
					NUFIP1	1	166,904,655	166,927,469
					KIAA1704	1	166,927,078	166,942,984
					GTF2F2	1	166,965,970	167,057,679
					TPT1	1	167,058,420	167,066,619
					KCTD4	1	166,995,127	166,995,900
7	14,087,975	16,675,492	2,587,517	0.86424929				
					CWC22	7	14,281,313	14,312,291
					ZNF385B	7	14,343,682	14,477,554
					SESTD1	7	14,533,688	14,570,925
					CCDC141	7	14,582,883	14,641,810
					PLEKHA3	7	14,887,747	14,903,030
					FKBP7	7	14,902,995	14,907,636
					DFNB59	7	14,909,332	14,917,988
					OSBPL6	7	14,935,828	14,977,668
					RBM45	7	15,031,727	15,042,794
					PDE11A	7	15,051,870	15,174,859
					AGPS	7	15,219,971	15,271,809
					NFE2L2	7	15,304,546	15,320,356
					HNRNPA3	7	15,327,805	15,341,998
					MTX2	7	15,709,764	15,742,887
					HOXD3	7	15,763,092	15,765,488
					HOXD4	7	15,777,579	15,779,976
					HOXD8	7	15,792,590	15,820,406
					HOXD9	7	15,798,954	15,800,275
					HOXD10	7	15,804,061	15,806,437
					HOXD11	7	15,814,096	15,815,924
					HOXD12	7	15,820,669	15,822,513
					HOXD13	7	15,827,616	15,828,537
					EVX2	7	15,833,129	15,835,917
					KIAA1715	7	15,868,345	15,908,061
					ATP5G3	7	16,150,149	16,153,215
					ATF2	7	16,159,461	16,206,145
					CHN1	7	16,219,611	16,299,658

					CHRNA1	7	16,305,300	16,310,101
					WIPF1	7	16,313,906	16,361,739
					GPR155	7	16,380,664	16,402,658
					SCRN3	7	16,404,276	16,412,287
					CIR1	7	16,412,437	16,433,377
					OLA1	7	16,463,674	16,548,896
					SP3	7	16,580,306	16,610,757
4	42,533,942	45,339,606	2,805,664	0.675018843				
					GALNT7	4	42,697,173	42,736,160
					HMGB2	4	42,740,630	42,743,052
					SAP30	4	42,754,695	42,760,373
					SCRG1	4	42,763,397	42,769,576
					FBXO8	4	43,131,548	43,148,365
					CEP44	4	43,149,107	43,165,568
					HPGD	4	43,211,798	43,236,772
					GLRA3	4	43,301,385	43,363,404
					GPM6A	4	43,565,210	43,666,838
					WDR17	4	43,727,567	43,765,630
					SPATA4	4	43,766,435	43,773,179
					ASB5	4	43,775,824	43,808,347
					SPCS3	4	43,812,168	43,817,012
					VEGFC	4	43,882,837	43,954,986
					MTHFD2L	4	44,089,931	44,115,535
					EPGN	4	44,118,458	44,127,174
					EREG	4	44,141,004	44,149,715
					USO1	4	44,202,175	44,228,430
					G3BP2	4	44,236,819	44,252,964
					BMP2K	4	44,313,412	44,349,074
					PAQR3	4	44,355,867	44,361,796
					ANTXR2	4	44,658,935	44,721,258
					PRDM8	4	44,734,247	44,737,047
					FGF5	4	44,740,274	44,743,143
					BMP3	4	44,875,121	44,892,847
					RASGEF1B	4	45,006,257	45,022,555
					PRKG2	4	45,032,980	45,054,431
					PKD2	4	45,060,159	45,074,721
					SPP1	4	45,074,192	45,077,215
					IBSP	4	45,089,887	45,091,107
					SPARCL1	4	45,106,288	45,113,208
					NUDT9	4	45,114,314	45,120,701
					KLHL8	4	45,144,527	45,157,728
					AFF1	4	45,164,032	45,214,295
					PTPN13	4	45,225,572	45,330,749
9	15,666,220	18,477,568	2,811,348	0.672429045				
					EIF4A2	9	15,671,724	15,678,579
					RFC4	9	15,678,911	15,690,512
					MCF2L2	9	15,742,979	15,839,708
					LAMP3	9	15,845,910	15,857,555
					MCCC1	9	15,864,983	15,880,897
					DCUN1D1	9	15,894,430	15,904,156
					ATP11B	9	15,908,775	16,005,533
					DNAJC19	9	16,559,670	16,562,902
					FXR1	9	16,565,596	16,587,667
					CCDC39	9	16,646,100	16,662,762
					TTC14	9	16,663,283	16,672,397
					PEX5L	9	16,760,382	16,850,020
					USP13	9	16,861,502	16,902,461
					NDUFB5	9	16,903,953	16,907,474
					MRPL47	9	16,907,572	16,911,041
					ACTL6A	9	16,911,951	16,921,482
					GNB4	9	16,972,459	16,979,703
					MFN1	9	16,984,938	17,003,984
					ZNF639	9	17,007,571	17,011,621
					PIK3CA	9	17,020,759	17,042,366
					ZMAT3	9	17,052,618	17,062,023
					NAALADL2	9	17,795,977	17,991,569
					NLGN1	9	18,196,758	18,471,834

					B3GNT5	9	15,777,289	15,778,413
					SOX2	9	16,342,563	16,343,510
2	10,926,468	13,747,682	2,821,214	0.656289587				
					PFKP	2	11,354,185	11,395,733
					PITRM1	2	11,396,672	11,421,465
					GJD4	2	12,778,766	12,787,894
					CCNY	2	12,793,547	12,910,204
					CREM	2	12,930,882	12,960,732
					CUL2	2	12,972,707	13,022,987
					PARD3	2	13,068,736	13,498,548
					FZD8	2	12,761,773	12,762,399
1	145,101,546	147,940,232	2,838,686	0.636827809				
					UGGT2	1	145,103,089	145,180,354
					DNAJC3	1	145,184,466	145,216,297
					DZIP1	1	145,229,679	145,262,325
					CLDN10	1	145,266,284	145,279,564
					ABCC4	1	145,358,360	145,506,681
					GPR180	1	145,671,022	145,692,774
					TGDS	1	145,692,790	145,705,488
					DCT	1	145,716,342	145,737,439
					GPC5	1	146,831,053	147,232,836
2	15,286,481	18,178,069	2,891,588	0.588733812				
					MPP7	2	15,410,320	15,499,606
					ARMC4	2	15,517,575	15,589,648
					MKX	2	15,606,690	15,649,798
					RAB18	2	15,704,613	15,717,690
					YME1L1	2	15,807,104	15,827,193
					MASTL	2	15,830,069	15,845,780
					ACBD5	2	15,848,492	15,875,313
					ABI1	2	15,897,343	15,947,115
					PDSS1	2	15,977,931	15,999,697
					APBB1IP	2	16,026,711	16,084,527
					GAD2	2	16,147,743	16,180,423
					MYO3A	2	16,182,205	16,276,588
					GPR179	2	16,372,423	16,557,109
					ENKUR	2	16,613,396	16,622,552
					PRTFDC1	2	16,631,918	16,676,613
					ARHGAP21	2	16,677,325	16,817,876
					KIAA1217	2	16,817,533	17,151,976
					OTUD1	2	17,237,193	17,240,043
					C10orf67	2	17,250,380	17,288,284
					PTF1A	2	17,298,877	17,299,386
					ARMC3	2	17,331,978	17,388,781
					PIP4K2A	2	17,424,993	17,525,165
					SPAG6	2	17,577,501	17,600,696
					COMMD3	2	17,625,279	17,653,946
					DNAJC1	2	17,662,537	17,798,689
					MLLT10	2	17,799,470	17,915,028
						2	17,930,605	17,933,285
					THNSL1	2	16,606,746	16,608,950
1	132,848,000	136,591,630	3,743,630	0.09042871				
					RFX8	1	132,861,202	132,895,021
					MAP4K4	1	132,936,573	133,084,109
					IL1R2	1	133,109,396	133,119,021
					IL1RL1	1	133,227,061	133,252,726
					IL18R1	1	133,259,701	133,278,851
					IL18RAP	1	133,283,645	133,298,556
					SLC9A4	1	133,300,715	133,329,117
					SLC9A2	1	133,354,849	133,381,741
					MFSD9	1	133,391,984	133,402,160
					TMEM182	1	133,408,227	133,435,369
					MRPS9	1	134,393,340	134,425,489
					TGFBRAP1	1	134,450,449	134,476,912
					C1H2ORF49	1	134,481,595	134,496,274
					FHL2	1	134,496,864	134,536,397
					NCK2	1	134,633,927	134,714,716
					C2ORF40	1	134,771,683	134,776,149

					ST6GAL2	1	135,007,544	135,053,400
					SLC5A7	1	135,397,400	135,423,764
					SULT1C	1	135,521,670	135,527,319
					RANBP2	1	135,763,887	135,793,418
					EDAR	1	135,819,209	135,882,851
					UPF3A	1	136,254,098	136,279,867
					CDC16	1	136,281,362	136,301,395
					RASA3	1	136,345,769	136,476,626
					GAS6	1	136,509,160	136,547,639
					SOWAHC	1	136,196,812	136,198,516
					CHAMP1	1	136,241,415	136,244,261
6	26,136,406	29,922,314	3,785,908	0.083770615				
					GPAM	6	26,158,125	26,183,815
					TECTB	6	26,215,254	26,223,330
					ACSL5	6	26,247,834	26,264,411
					ZDHHC6	6	26,264,533	26,277,485
					VTI1A	6	26,277,903	26,526,700
					TCF7L2	6	26,590,486	26,762,220
					HABP2	6	26,971,704	26,991,533
					NRAP	6	26,991,279	27,037,248
					CASP7	6	27,042,298	27,062,965
					PLEKHS1	6	27,064,759	27,081,909
					DCLRE1A	6	27,087,946	27,102,495
					NHLRC2	6	27,103,400	27,134,851
					C10ORF118	6	27,217,490	27,246,216
					TDRD1	6	27,245,682	27,267,251
					COL9A3	6	27,267,818	27,285,089
					AFAP1L2	6	27,287,975	27,335,710
					ABLIM1	6	27,359,430	27,532,008
					FAM160B1	6	27,536,767	27,571,562
					TRUB1	6	27,594,383	27,622,142
					ATRNL1	6	27,680,834	28,014,799
					GFRA1	6	28,152,303	28,288,081
					CCDC172	6	28,309,882	28,328,718
					HSPA12A	6	28,413,790	28,450,155
					ENO4	6	28,500,759	28,518,682
					KIAA1598	6	28,516,161	28,575,056
					KCNK18	6	28,649,189	28,653,448
					PDZD8	6	28,696,248	28,747,446
					EMX2	6	28,814,903	28,820,582
					RAB11FIP2	6	29,003,777	29,047,647
					FAM204A	6	29,173,024	29,190,310
					C6H10ORF46	6	29,348,791	29,388,352
					EIF3A	6	29,508,820	29,537,182
					FAM45A	6	29,538,376	29,547,379
					SFXN4	6	29,550,386	29,559,570
					PRDX3	6	29,560,387	29,565,670
					GRK5	6	29,572,855	29,771,556
					RGS10	6	29,757,675	29,767,309
					TIAL1	6	29,782,065	29,799,900
					BAG3	6	29,813,247	29,832,893
					INPP5F	6	29,851,879	29,889,013
					MCMBP	6	29,884,934	29,905,383
					SEC23IP	6	29,905,638	29,921,730
					ADRB1	6	27,190,009	27,191,226
					NANOS1	6	29,504,247	29,505,285
3	103,727,370	108,395,737	4,668,367	0.008620192				
					MFSD2B	3	103,746,139	103,779,362
					C2orf44	3	103,783,106	103,791,013
					FKBP1B	3	103,866,156	103,869,947
					SF3B14	3	103,873,567	103,877,409
					TP53I3	3	103,877,869	103,881,489
					PFN4	3	103,884,507	103,887,705
					ITSN2	3	103,895,602	103,938,672
					GPN1	3	103,970,332	103,984,905
					ZNF512	3	103,984,631	104,002,721
					FNDC4	3	104,008,233	104,014,124

IFT172	3	104,023,556	104,057,142
KRTCAP3	3	104,057,388	104,059,799
NRBP1	3	104,059,886	104,085,090
PPM1G	3	104,085,785	104,102,280
ZNF513	3	104,109,217	104,113,430
SNX17	3	104,109,900	104,118,369
EIF2B4	3	104,118,906	104,125,481
AGBL5	3	104,125,582	104,133,092
TMEM214	3	104,135,364	104,141,333
CAD	3	104,164,895	104,174,351
MPV17	3	104,177,374	104,181,451
GTF3C2	3	104,206,333	104,213,436
MAPRE3	3	104,213,713	104,218,289
DNMT3A	3	104,239,248	104,259,227
ASXL2	3	104,272,171	104,504,133
KIF3C	3	104,518,139	104,529,350
RAB10	3	104,556,702	104,571,975
GAREML	3	104,575,048	104,582,511
HADHA	3	104,583,827	104,607,643
HADHB	3	104,607,849	104,619,357
EPT1	3	104,645,877	104,668,714
PTK2B	3	104,676,810	104,700,808
CHRNA2	3	104,700,299	104,704,881
EPHX2	3	104,712,779	104,721,569
SCARA5	3	104,760,091	104,779,321
PBK	3	104,781,669	104,791,026
ESCO2	3	104,790,088	104,800,567
CCDC25	3	104,804,105	104,814,366
SCARA3	3	104,817,333	104,822,180
C2ORF18	3	104,835,719	104,839,201
KCNK3	3	104,844,800	104,855,646
C2orf70	3	104,887,264	104,889,731
CCDC164	3	104,967,445	104,979,487
TRIM35	3	104,980,477	104,985,240
STMN4	3	104,988,422	104,996,116
POMC	3	105,013,957	105,016,899
EFR3B	3	105,019,505	105,046,269
DNAJC27	3	105,062,440	105,072,565
ADCY3	3	105,072,727	105,085,294
CENPO	3	105,085,782	105,092,472
PTRHD1	3	105,092,550	105,100,626
NCOA1	3	105,103,875	105,211,518
ZNF395	3	105,294,830	105,304,932
PNOC	3	105,308,183	105,311,570
ELP3	3	105,336,342	105,420,205
FBXO16	3	105,432,483	105,455,360
FZD3	3	105,466,097	105,513,573
EXTL3	3	105,599,177	105,650,057
INTS9	3	105,654,633	105,723,673
HMBOX1	3	105,781,724	105,832,659
KIF13B	3	105,851,266	105,973,615
MSRA	3	106,054,095	106,314,882
PRSS55	3	106,318,274	106,332,544
RP1L1	3	106,375,249	106,398,770
XKR6	3	106,569,100	106,587,986
MTMR9	3	106,761,333	106,784,678
FAM167A	3	106,838,785	106,848,916
BLK	3	106,862,804	106,898,321
GATA4	3	106,954,748	106,976,693
NEIL2	3	106,981,676	106,984,917
FDFT1	3	106,985,673	106,996,753
CTSB	3	106,998,423	107,007,359
TRAM2	3	107,117,647	107,125,467
EFHC1	3	107,130,488	107,147,698
MCM3	3	107,167,586	107,179,385
PKHD1	3	107,230,220	107,461,810
TFAP2B	3	107,846,265	107,873,218

TFAP2D	3	107,894,065	107,943,864
PAQR8	3	107,155,454	107,156,515

Supplementary table 11d. Multispecies homologous synteny blocks (msHSBs) detected in sauropsids

Chr	msHSB start (bp)	msHSB end (bp)	msHSB length (bp)	Probability of being found under the Poisson process	Gene ID	Chr	Gene start (bp)	Gene end (bp)
3	24,475,771	25,985,031	1,509,260	0.999999993				
					PLEKHH2	3	24,491,110	24,545,482
					DYNC2LI1	3	24,548,141	24,570,676
					ABCG5	3	24,567,546	24,588,926
					ABCG8	3	24,585,792	24,598,091
					LRPPRC	3	24,608,804	24,695,385
					PPM1B	3	24,761,763	24,820,317
					SLC3A1	3	24,827,681	24,841,984
					PREPL	3	24,831,731	24,859,714
					SIX2	3	25,240,527	25,243,491
					SRBD1	3	25,432,172	25,549,520
					PRKCE	3	25,576,043	25,856,010
					EPAS1	3	25,951,104	25,982,708
1	10,916,110	12,431,817	1,515,707	0.999999988				
					CD36	1	11,005,425	11,038,504
					GNAT3	1	11,086,584	11,113,407
					GNAI3	1	11,190,979	11,223,596
					MAGI2	1	11,930,694	12,230,824
					PHTF2	1	12,251,576	12,314,104
					TMEM60	1	12,314,478	12,318,704
					RSBN1L	1	12,327,050	12,358,827
					PTPN18	1	12,381,899	12,422,097
5	30,029,578	31,568,709	1,539,131	0.999999969				
					MEIS2	5	30,506,655	30,675,196
					C5H15orf41	5	30,717,442	30,832,671
					ATPBD4	5	31,253,451	31,438,936
					ZNF770	5	31,468,292	31,472,971
					AQR	5	31,481,161	31,528,089
					ACTC1	5	31,546,121	31,552,902
1	132,881,781	134,421,245	1,539,464	0.999999961				
					MAP4K4	1	132,936,573	133,084,109
					IL1R2	1	133,109,396	133,119,021
					IL1RL1	1	133,227,061	133,252,726
					IL18R1	1	133,259,701	133,278,851
					IL18RAP	1	133,283,645	133,298,556
					SLC9A4	1	133,300,715	133,329,117
					SLC9A2	1	133,354,849	133,381,741
					MFSD9	1	133,391,984	133,402,160
					TMEM182	1	133,408,227	133,435,369
20	9,563,535	11,104,188	1,540,653	0.999999963				
					OPRL1	20	9,598,823	9,610,812
					RGS19	20	9,692,144	9,695,672
					TCEA2	20	9,699,117	9,715,280
					SOX18	20	9,728,462	9,733,689
					PRPF6	20	9,759,349	9,783,514
					SAMD10	20	9,790,153	9,799,694
					UCKL1	20	9,837,210	9,851,741
					DNAJC5	20	9,868,316	9,882,697
					SLC2A4RG	20	9,958,400	9,961,363
						20	9,970,349	9,976,884
					TNFRSF6B	20	9,998,639	10,003,173
					STMN3	20	10,049,567	10,060,098
					PDYN	20	10,129,970	10,133,174
					NSFL1C	20	10,152,354	10,159,223
					SDCBP2	20	10,178,536	10,180,514
					PSMF1	20	10,210,994	10,216,774
					RSPO4	20	10,230,159	10,233,326
					ANGPT4	20	10,237,437	10,245,306
					FAM110A	20	10,246,853	10,249,532

					SLC52A3	20	10,254,506	10,257,465
					TCF15	20	10,271,534	10,273,928
					TBC1D20	20	10,304,367	10,310,001
					NRSN2	20	10,309,574	10,313,704
					C20orf96	20	10,320,202	10,326,956
					REM1	20	10,327,399	10,330,347
					HM13	20	10,334,332	10,346,234
					BCL2L1	20	10,359,785	10,377,592
					TPX2	20	10,382,093	10,392,964
					MYLK2	20	10,395,495	10,403,016
					DUSP15	20	10,412,313	10,419,874
					TTLL9	20	10,420,572	10,431,015
					PDRG1	20	10,431,992	10,433,839
					XKR7	20	10,436,888	10,443,076
					C20ORF160	20	10,446,236	10,449,489
					HCK	20	10,451,055	10,456,874
					TM9SF4	20	10,461,799	10,472,166
					PLAGL2	20	10,480,919	10,483,343
					POFUT1	20	10,487,518	10,491,477
					KIF3B	20	10,492,514	10,502,873
					ASXL1	20	10,505,808	10,517,025
					COMMD7	20	10,585,431	10,590,382
					DNMT3B	20	10,595,952	10,609,143
					MAPRE1	20	10,610,165	10,617,328
					TENP	20	10,643,158	10,647,278
					BPIFB6	20	10,649,024	10,653,537
					BPIFB3	20	10,656,808	10,661,438
					BPIFB4	20	10,663,664	10,668,879
					CDK5RAP1	20	10,679,042	10,686,268
					KIAA1755	20	10,700,368	10,707,694
					TGM2	20	10,714,657	10,723,053
					RPRD1B	20	10,726,664	10,747,756
					TTI1	20	10,747,457	10,760,392
					VSTM2L	20	10,761,591	10,764,145
					CTNNBL1	20	10,772,139	10,813,719
					WFDC2	20	10,829,801	10,840,153
					DNTTIP1	20	10,849,414	10,853,705
					UBE2C	20	10,853,789	10,854,730
					TNNC2	20	10,856,323	10,858,120
					SNX21	20	10,859,488	10,861,629
					ACOT8	20	10,859,880	10,864,460
					ZSWIM3	20	10,864,587	10,870,236
					NEURL2	20	10,871,289	10,873,861
					CTSA	20	10,874,294	10,878,191
					PLTP	20	10,878,671	10,881,926
					PCIF1	20	10,887,208	10,895,373
					ZNF335	20	10,900,409	10,909,059
					MMP9	20	10,917,368	10,921,453
					SLC12A5	20	10,922,030	10,944,646
					NCOA5	20	10,950,277	10,963,160
					CD40	20	10,966,821	10,970,503
					CDH22	20	10,990,502	11,029,175
					SLC35C2	20	11,071,603	11,076,564
					ELMO2	20	11,076,802	11,090,032
					NPBWR2	20	9,589,297	9,590,932
					SCRT2	20	10,263,332	10,264,039
2	8,014,363	9,563,687	1,549,324	0.999999941				
					SHH	2	8,080,091	8,089,855
					RNF32	2	8,414,446	8,427,357
					LMBR1	2	8,434,744	8,494,298
					NOM1	2	8,513,775	8,529,642
					MNX1	2	8,538,868	8,541,508
					UBE3C	2	8,582,314	8,644,842
					PTPRN2	2	8,785,742	9,406,733

					NCAPG2	2	9,417,857	9,456,238
					ESYT2	2	9,467,167	9,532,244
3	49,583,623	51,152,885	1,569,262	0.999999861				
					SCAF8	3	49,604,475	49,635,861
					TFB1M	3	49,868,438	49,895,829
					CLDN20	3	49,875,601	49,880,776
					NOX3	3	49,915,188	49,952,437
					ARID1B	3	50,465,764	50,754,522
					TMEM242	3	50,810,196	50,829,319
					SNX9	3	50,997,690	51,052,016
					SYNJ2	3	51,063,895	51,127,835
3	56,963,360	58,590,234	1,626,874	0.999998413				
					EPB41L2	3	56,980,129	57,075,289
					SAMD3	3	57,322,882	57,363,225
					L3MBTL3	3	57,360,442	57,425,789
					C6ORF191	3	57,466,325	57,472,268
					ARHGAP18	3	57,506,532	57,565,989
					LAMA2	3	57,586,334	57,923,906
					PTPRK	3	58,029,862	58,422,557
					THEMIS	3	58,437,710	58,506,141
					C6orf58	3	58,527,005	58,539,770
					TMEM200A	3	57,238,296	57,239,765
Z	59,236,376	60,875,956	1,639,580	0.99999769				
4	73,572,127	75,214,999	1,642,872	0.999997176				
					PPARGC1A	4	73,626,292	73,691,855
					GPR125	4	74,015,279	74,072,320
					KCNIP4	4	74,436,584	74,527,405
					PACRGL	4	74,526,045	74,539,587
					SLIT2	4	74,557,149	74,802,699
1	109,932,747	111,600,312	1,667,565	0.99999201				
					CXorf36	1	110,128,634	110,143,679
					FUNDC1	1	110,347,436	110,362,439
					EFHC2	1	110,382,985	110,442,596
					NDP	1	110,522,886	110,531,383
					MAOB	1	110,555,374	110,609,289
					MAOA	1	110,613,430	110,658,911
					GPR34	1	111,232,706	111,235,607
					NYX	1	111,334,091	111,336,368
					GPR82	1	111,219,959	111,220,993
2	133,158,961	134,837,147	1,678,186	0.999989585				
					TRPS1	2	134,048,833	134,252,747
					EIF3H	2	134,628,301	134,709,262
					UTP23	2	134,720,381	134,723,977
					RAD21	2	134,749,874	134,773,800
17	2,877,535	4,580,793	1,703,258	0.999975731				
					PAPPA	17	3,040,970	3,209,266
					ASTN2	17	3,219,825	3,482,002
					TLR4	17	3,566,454	3,571,907
					DBC1	17	3,985,882	4,064,290
					CDK5RAP2	17	4,495,102	4,566,562
1	80,304,616	82,024,763	1,720,147	0.999957338				
					LSAMP	1	81,373,015	81,656,000
					GAP43	1	81,680,485	81,734,254
3	58,916,848	60,639,979	1,723,131	0.999958658				
					CENPW	3	59,060,255	59,067,195
					TRMT11	3	59,178,746	59,205,181
					HINT3	3	59,212,941	59,219,743
					NCOA7	3	59,223,034	59,291,522
					HDDC2	3	59,516,400	59,525,907
					TPD52L1	3	59,527,859	59,580,356
					RNF217	3	59,606,826	59,662,599
					NKAIN2	3	59,710,415	59,999,592
					TRDN	3	60,315,841	60,432,093
					CLVS2	3	60,548,727	60,598,139

4	20,823,966	22,548,595	1,724,629	0.999957445				
					GRIA2	4	20,873,025	20,960,836
					FAM198B	4	21,221,107	21,234,597
					TMEM144	4	21,242,338	21,257,193
					RXFP1	4	21,328,064	21,376,386
					ETFDH	4	21,379,598	21,396,998
					PPID	4	21,395,223	21,405,790
					FNIP2	4	21,425,883	21,457,823
					C4ORF45	4	21,455,357	21,496,063
					RAPGEF2	4	21,509,902	21,683,609
					FSTL5	4	22,250,827	22,516,765
5	10,087,655	11,834,285	1,746,630	0.999921853				
					SOX6	5	10,549,851	10,784,968
					C5H11ORF58	5	10,948,695	10,953,363
					PLEKHA7	5	10,959,448	11,057,508
					RPS13	5	11,136,190	11,139,937
					PIK3C2A	5	11,142,129	11,205,960
					NUCB2	5	11,205,875	11,231,516
					KCNJ11	5	11,247,848	11,250,065
					ABCC8	5	11,253,945	11,319,342
					USH1C	5	11,334,156	11,377,576
					OTOG	5	11,381,876	11,476,414
					MYOD1	5	11,578,793	11,582,278
					KCNC1	5	11,597,391	11,714,071
3	65,651,343	67,404,826	1,753,483	0.999903095				
					RPF2	3	65,709,193	65,721,929
					GTF3C6	3	65,721,376	65,726,791
					AMD1	3	65,744,041	65,761,269
					CDK19	3	65,801,524	65,899,585
					SLC22A16	3	65,931,799	65,962,885
					DDO	3	65,969,395	65,975,774
					C6ORF186	3	65,985,390	66,029,579
					CDC40	3	66,033,229	66,074,352
					WASF1	3	66,123,600	66,154,746
					GPR6	3	66,173,387	66,197,130
					FIG4	3	66,201,896	66,268,774
					AK9	3	66,269,578	66,325,155
					ZBTB24	3	66,327,020	66,334,932
					PPIL6	3	66,340,530	66,350,895
					CD164	3	66,357,144	66,365,037
					C6ORF183	3	66,413,334	66,432,788
					CEP57L1	3	66,460,394	66,473,222
					SESN1	3	66,547,545	66,556,746
					ARMC2	3	66,560,205	66,617,959
					FOXO3	3	66,702,483	66,716,393
					LACE1	3	66,809,734	66,870,599
					SNX3	3	66,876,089	66,895,012
					NR2E1	3	66,898,978	66,913,949
					OSTM1	3	66,950,856	66,961,817
					SEC63	3	66,978,001	67,033,488
					SCML4	3	67,042,180	67,098,929
					SOBP	3	67,134,086	67,243,457
					PDSS2	3	67,259,779	67,378,577
					BEND3	3	67,393,979	67,403,976
4	23,247,017	25,022,794	1,775,777	0.999829099				
					KLHL2	4	23,265,508	23,325,234
					SC4MOL	4	23,326,894	23,333,143
					CPE	4	23,336,496	23,382,106
					TLL1	4	23,520,463	23,656,666
					SPOCK3	4	23,947,374	24,051,813
					ANXA10	4	24,400,386	24,416,023
					PALLD	4	24,486,948	24,675,736
					AADAT	4	24,691,532	24,707,952
					MFAP3L	4	24,743,867	24,747,796

					C4ORF27	4	24,796,835	24,803,472
					CLCN3	4	24,804,714	24,864,749
					NEK1	4	24,866,404	24,909,512
					CBR4	4	25,012,882	25,021,145
2	120,455,577	122,248,807	1,793,230	0.999726922				
					STMN2	2	120,505,360	120,515,753
					HEY1	2	120,538,764	120,541,690
					TPD52	2	120,668,873	120,704,236
					ZBTB10	2	120,783,328	120,809,194
					ZNF704	2	120,881,814	120,900,516
					PAG1	2	120,988,402	121,062,954
					FABP5	2	121,148,557	121,153,615
					PMP2	2	121,215,806	121,220,355
					FABP4	2	121,225,723	121,228,944
					IMPA1	2	121,262,379	121,277,486
					ZFAND1	2	121,279,568	121,285,857
					CHMP4C	2	121,287,839	121,304,205
					SNX16	2	121,309,189	121,333,508
3	35,427,107	37,224,095	1,796,988	0.999708001				
					GREM2	3	35,493,725	35,533,009
					FMN2	3	35,540,378	35,680,173
					CHRM3	3	35,727,538	35,877,494
					RYR2	3	36,498,791	36,661,068
					MTR	3	36,890,573	36,933,337
					ACTN2	3	36,948,211	37,014,188
					HEATR1	3	37,025,129	37,060,227
					LGALS8	3	37,061,029	37,073,707
					EDARADD	3	37,078,799	37,097,401
					ERO1LB	3	37,097,636	37,134,158
					GPR137B	3	37,135,755	37,159,463
					NID1	3	37,178,196	37,213,970
3	45,246,962	47,049,757	1,802,795	0.999666018				
					EPM2A	3	45,769,623	45,811,385
					FBXO30	3	45,824,455	45,831,133
					SHPRH	3	45,868,769	45,921,436
					GRM1	3	45,937,157	46,118,743
					RAB32	3	46,154,328	46,173,538
					STXBP5	3	46,388,675	46,489,008
					SAMD5	3	46,522,593	46,647,011
2	137,878,625	139,781,764	1,903,139	0.997427019				
					TMEM65	2	137,986,349	138,016,847
					TATDN1	2	138,058,219	138,071,047
					NDUFB9	2	138,071,064	138,074,938
					MTSS1	2	138,077,064	138,193,553
					SQLE	2	138,265,073	138,279,203
					KIAA0196	2	138,283,684	138,306,583
					NSMCE2	2	138,307,230	138,433,695
					TRIB1	2	138,436,990	138,444,511
					FAM84B	2	138,873,014	138,876,023
					MYC	2	139,316,928	139,321,894
					RNF139	2	138,055,501	138,057,312
1	141,339,838	143,294,319	1,954,481	0.993846292				
					SLC10A2	1	141,914,133	141,925,460
					BIVM	1	142,024,553	142,041,190
					KDELC1	1	142,045,388	142,054,410
					C13ORF27	1	142,057,711	142,064,431
					METTL21C	1	142,092,496	142,099,273
					TPP2	1	142,097,596	142,147,887
					ITGBL1	1	142,614,261	142,752,083
					NALCN	1	142,765,240	142,969,863
					TMTC4	1	143,142,401	143,197,059
					A2LD1	1	143,230,157	143,230,654
2	5,838,445	7,833,125	1,994,680	0.989468196				
					XYLB	2	6,140,053	6,166,029

					NUB1	2	6,187,456	6,200,306
					WDR86	2	6,203,237	6,221,340
					CRYGN	2	6,235,658	6,239,901
					PRKAG2	2	6,247,476	6,461,068
					GALNT11	2	6,503,696	6,527,983
					MLL3	2	6,528,611	6,717,633
					XRCC2	2	6,755,237	6,767,527
					DPP6	2	7,411,564	7,635,852
					PAXIP1	2	7,658,648	7,692,640
					HTR5A	2	7,711,450	7,714,285
					INSIG1	2	7,827,461	7,832,977
1	145,101,546	147,118,880	2,017,334	0.985581828				
					UGGT2	1	145,103,089	145,180,354
					DNAJC3	1	145,184,466	145,216,297
					DZIP1	1	145,229,679	145,262,325
					CLDN10	1	145,266,284	145,279,564
					ABCC4	1	145,358,360	145,506,681
					GPR180	1	145,671,022	145,692,774
					TGDS	1	145,692,790	145,705,488
					DCT	1	145,716,342	145,737,439
2	112,226,473	114,277,051	2,050,578	0.979349077				
					CA8	2	112,413,504	112,453,419
					RAB2A	2	112,517,303	112,558,232
					CHD7	2	112,612,964	112,699,976
					CLVS1	2	112,882,281	112,978,767
					NKAIN3	2	113,137,211	113,469,039
					GGH	2	113,490,054	113,501,161
					TTPA	2	113,502,155	113,517,713
					YTHDF3	2	113,543,309	113,551,391
					CYP7B1	2	114,093,263	114,114,645
13	813,059	2,865,235	2,052,176	0.978518494				
					PFDN1	13	928,207	957,649
					HBEGF	13	961,553	967,753
					SLC4A9	13	972,298	989,098
					SRA1	13	1,095,133	1,095,980
					APBB3	13	1,096,712	1,100,458
					PCDHGC3	13	1,217,194	1,247,116
					HDAC3	13	1,277,095	1,290,277
					RELL2	13	1,292,499	1,299,951
					FCHSD1	13	1,300,987	1,305,466
					ARAP3	13	1,309,826	1,324,899
					PCDH1	13	1,408,177	1,457,586
					PCDH12	13	1,751,168	1,760,560
					ECSCR	13	1,767,729	1,785,069
					DNAJC18	13	1,788,284	1,799,938
					MZB1	13	1,806,725	1,808,111
					SLC23A1	13	1,808,718	1,812,108
					PAIP2	13	1,813,947	1,819,364
					MATR3	13	1,825,981	1,851,149
					SIL1	13	1,869,523	1,957,625
					CTNNA1	13	1,960,516	2,068,702
					LRRTM2	13	2,010,626	2,012,530
					HSPA9	13	2,134,785	2,156,387
					ETF1	13	2,158,826	2,184,873
					FBXW11	13	2,208,007	2,258,082
					FGF18	13	2,567,435	2,611,832
					NPM1	13	2,641,231	2,651,587
					TLX3	13	2,673,915	2,675,387
					RANBP17	13	2,681,554	2,828,621
					PURA	13	860,147	860,890
2	125,160,507	127,244,400	2,083,893	0.970480684				
					TRIQK	2	125,273,982	125,333,683
					FAM92A1	2	125,604,624	125,621,196
					RBM12B	2	125,623,603	125,630,097

					TMEM67	2	125,632,568	125,659,384
					PDP1	2	125,683,595	125,690,722
					CDH17	2	125,754,667	125,783,019
					GEM	2	125,802,915	125,811,572
					RAD54B	2	125,844,098	125,903,394
					FSBP	2	125,877,035	125,887,013
					KIAA1429	2	125,925,066	125,952,171
					ESRP1	2	125,956,620	125,988,739
					DPY19L4	2	126,052,840	126,076,741
					INTS8	2	126,126,452	126,151,324
					CCNE2	2	126,151,226	126,161,848
					TP53INP1	2	126,170,346	126,183,360
					NDUFAF6	2	126,201,059	126,216,326
					PLEKHF2	2	126,225,096	126,240,839
					C8orf37	2	126,258,109	126,269,454
					MTERFD1	2	126,597,212	126,615,684
					PTDSS1	2	126,616,011	126,645,632
					SDC2	2	126,740,060	126,929,143
					MTDH	2	127,057,053	127,088,247
					LAPTM4B	2	127,102,614	127,165,676
					MATN2	2	127,183,885	127,242,408
12	9,123,361	11,227,411	2,104,050	0.963143916				
					RPN1	12	9,149,093	9,156,192
					GATA2	12	9,185,919	9,200,490
					EEFSEC	12	9,379,038	9,490,404
					RUVBL1	12	9,495,555	9,513,271
					SEC61A1	12	9,527,460	9,536,843
					KBTBD12	12	9,552,454	9,577,601
					MGLL	12	9,598,736	9,655,915
					ABTB1	12	9,657,435	9,685,045
					PODXL2	12	9,689,545	9,717,481
					MCM2	12	9,725,752	9,737,075
					GPR175	12	9,738,594	9,754,460
					PLXNA1	12	9,887,588	9,971,748
					TXNRD3	12	10,366,876	10,387,808
					CHST13	12	10,449,680	10,473,797
					SLC41A3	12	10,475,439	10,492,889
					KLF15	12	10,614,316	10,626,926
					UROC1	12	10,667,219	10,697,052
					CHCHD4	12	10,756,017	10,764,587
					TMEM43	12	10,764,796	10,775,842
					XPC	12	10,776,979	10,787,331
					SLC6A6	12	10,950,982	10,995,867
					DNAJB8	12	9,245,863	9,246,974
1	172,850,666	174,964,633	2,113,967	0.959229799				
					RFC3	1	173,144,179	173,161,079
					STARD13	1	173,241,712	173,493,625
					KL	1	173,504,524	173,551,941
					PDS5B	1	173,655,059	173,730,298
					N4BP2L1	1	173,808,326	173,820,489
					BRCA2	1	173,820,724	173,857,456
					ZAR1	1	173,859,596	173,861,411
					FRY	1	173,863,589	174,002,319
					B3GALTL	1	174,203,173	174,247,847
					HSPH1	1	174,262,982	174,286,406
					ALOX5AP	1	174,335,143	174,344,760
					USPL1	1	174,350,051	174,363,188
					KATNAL1	1	174,496,738	174,524,653
					UBL3	1	174,588,070	174,645,255
					SLC7A1	1	174,687,111	174,726,955
9	20,585,078	22,714,790	2,129,712	0.956044778				
					SLITRK3	9	20,586,079	20,587,887
					OTOL1	9	21,401,027	21,404,693
					SPTSSB	9	21,417,214	21,425,710

					NMD3	9	21,436,129	21,447,358
					PPM1L	9	21,476,302	21,557,159
					KPNA4	9	21,597,000	21,610,445
					SMC4	9	21,626,305	21,657,730
					IFT80	9	21,661,987	21,703,750
					IL12A	9	21,727,352	21,729,152
					IQCJ	9	21,810,250	21,844,816
					MFSD1	9	21,872,165	21,885,523
					RARRES1	9	21,904,934	21,912,426
					GFM1	9	21,913,507	21,933,383
					LXN	9	21,919,321	21,923,817
					MLF1	9	21,936,183	21,948,704
					RSRC1	9	21,958,768	22,066,377
					VEPH1	9	22,173,837	22,221,855
					PTX3	9	22,180,190	22,186,891
					CCNL1	9	22,236,549	22,248,075
					LEKR1	9	22,281,614	22,298,405
					TIPARP	9	22,313,993	22,346,827
					SSR3	9	22,347,715	22,354,679
					GMPS	9	22,393,885	22,417,726
					SLC33A1	9	22,418,561	22,429,141
					C3orf33	9	22,430,628	22,434,432
					MME	9	22,527,165	22,563,563
					GPR149	9	22,627,534	22,648,732
					DHX36	9	22,652,324	22,670,920
					B3GALNT1	9	21,566,727	21,567,710
					ARL14	9	21,572,455	21,573,027
1	134,451,427	136,591,630	2,140,203	0.948315253				
					C1H2ORF49	1	134,481,595	134,496,274
					FHL2	1	134,496,864	134,536,397
					NCK2	1	134,633,927	134,714,716
					C2ORF40	1	134,771,683	134,776,149
					ST6GAL2	1	135,007,544	135,053,400
					SLC5A7	1	135,397,400	135,423,764
					SULT1C	1	135,521,670	135,527,319
					RANBP2	1	135,763,887	135,793,418
					EDAR	1	135,819,209	135,882,851
					UPF3A	1	136,254,098	136,279,867
					CDC16	1	136,281,362	136,301,395
					RASA3	1	136,345,769	136,476,626
					GAS6	1	136,509,160	136,547,639
					SOWAHC	1	136,196,812	136,198,516
					CHAMP1	1	136,241,415	136,244,261
2	102,945,374	105,091,268	2,145,894	0.947112549				
					OSBPL1A	2	102,948,952	103,020,897
					IMPACT	2	103,033,304	103,051,363
					ZNF521	2	103,383,680	103,410,706
					SS18	2	103,745,620	103,788,130
					TAF4B	2	103,821,689	103,883,809
					KCTD1	2	103,913,222	103,977,131
					AQP4	2	104,100,790	104,109,628
					CHST9	2	104,126,305	104,127,381
					CDH2	2	104,449,184	104,562,821
4	42,697,452	44,869,083	2,171,631	0.93579205				
					HMGB2	4	42,740,630	42,743,052
					SAP30	4	42,754,695	42,760,373
					SCRG1	4	42,763,397	42,769,576
					FBXO8	4	43,131,548	43,148,365
					CEP44	4	43,149,107	43,165,568
					HPGD	4	43,211,798	43,236,772
					GLRA3	4	43,301,385	43,363,404
					GPM6A	4	43,565,210	43,666,838
					WDR17	4	43,727,567	43,765,630
					SPATA4	4	43,766,435	43,773,179

					ASB5	4	43,775,824	43,808,347
					SPCS3	4	43,812,168	43,817,012
					VEGFC	4	43,882,837	43,954,986
					MTHFD2L	4	44,089,931	44,115,535
					EPGN	4	44,118,458	44,127,174
					EREG	4	44,141,004	44,149,715
					USO1	4	44,202,175	44,228,430
					G3BP2	4	44,236,819	44,252,964
					BMP2K	4	44,313,412	44,349,074
					PAQR3	4	44,355,867	44,361,796
					ANTXR2	4	44,658,935	44,721,258
					PRDM8	4	44,734,247	44,737,047
					FGF5	4	44,740,274	44,743,143
3	77,406,587	79,690,855	2,284,268	0.861275977				
					TPBG	3	77,636,164	77,637,640
					IBTK	3	77,659,755	77,717,628
					FAM46A	3	77,839,636	77,873,063
					BCKDHB	3	78,457,902	78,570,667
					TTK	3	78,612,134	78,643,792
					ELOVL4	3	78,677,336	78,713,537
					SH3BGRL2	3	78,756,279	78,772,633
					LCA5	3	78,816,727	78,828,429
					HMGN3	3	78,877,354	78,901,407
					PHIP	3	78,926,379	79,016,040
					IRAK1BP1	3	79,040,637	79,051,664
					HTR1B	3	79,492,220	79,493,385
6	23,049,412	25,352,313	2,302,901	0.847895207				
						6	23,104,676	23,106,678
					PCGF6	6	23,110,411	23,132,990
					TAF5	6	23,133,668	23,144,709
					USMG5	6	23,144,647	23,148,454
					PDCD11	6	23,149,107	23,171,490
					CALHM2	6	23,175,699	23,177,288
					CALHM1	6	23,181,808	23,183,651
					CALHM3	6	23,187,673	23,189,859
					NEURL	6	23,201,517	23,337,251
					SH3PXD2A	6	23,349,107	23,406,139
					OBFC1	6	23,585,345	23,623,213
					SLK	6	23,623,564	23,666,255
					COL17A1	6	23,670,969	23,706,526
					SFR1	6	23,730,903	23,734,477
					WDR96	6	23,736,120	23,778,750
					CCDC147	6	23,826,509	23,881,678
					SORCS3	6	23,968,010	24,235,717
					SORCS1	6	24,664,740	24,940,362
					XPNPEP1	6	25,219,899	25,248,494
					ADD3	6	25,264,735	25,352,280
					ITPRIP	6	23,801,103	23,802,752
7	30,893,512	33,294,488	2,400,976	0.757830101				
					SPOPL	7	30,896,072	30,910,605
					NXPH2	7	30,928,491	30,961,286
					KYNU	7	32,143,918	32,202,554
					ARHGAP15	7	32,211,979	32,530,918
					GTDC1	7	32,617,757	32,782,028
					ZEB2	7	32,812,269	32,916,777
1	97,856,037	100,318,705	2,462,668	0.685351891				
					USP25	1	97,936,529	98,030,131
					CXADR	1	98,638,187	98,668,892
					C1H21ORF91	1	98,700,854	98,720,026
					CHODL	1	98,864,912	98,893,667
					TMPRSS15	1	98,898,052	98,906,375
					NCAM2	1	100,155,276	100,281,469
11	3,163,524	5,711,059	2,547,535	0.595901276				
					MMP2	11	3,182,494	3,220,410

					IRX5	11	3,356,406	3,358,310
					FTO	11	4,129,194	4,353,272
					RPGRIP1L	11	4,357,663	4,419,746
					AKTIP	11	4,456,387	4,469,147
					RBL2	11	4,474,789	4,490,821
					CHD9	11	4,503,008	4,563,980
					TOX3	11	4,752,569	4,824,785
					SALL1	11	5,418,071	5,433,815
					CYLD	11	5,636,333	5,654,522
					SNX20	11	5,663,396	5,670,064
2	86,455,552	89,072,737	2,617,185	0.525497027				
					IRX2	2	86,624,614	86,631,727
					IRX1	2	87,131,471	87,135,061
					ANKS6	2	87,823,412	87,864,890
					GALNT12	2	87,893,690	87,919,460
					CTNNAL1	2	88,499,507	88,557,002
					FAM206A	2	88,558,023	88,560,120
					EXOC3	2	88,911,152	88,929,189
					PDCD6	2	88,929,444	88,935,921
					AHRR	2	88,979,697	89,045,510
9	15,666,220	18,477,568	2,811,348	0.350634191				
					EIF4A2	9	15,671,724	15,678,579
					RFC4	9	15,678,911	15,690,512
					MCF2L2	9	15,742,979	15,839,708
					LAMP3	9	15,845,910	15,857,555
					MCCC1	9	15,864,983	15,880,897
					DCUN1D1	9	15,894,430	15,904,156
					ATP11B	9	15,908,775	16,005,533
					DNAJC19	9	16,559,670	16,562,902
					FXR1	9	16,565,596	16,587,667
					CCDC39	9	16,646,100	16,662,762
					TTC14	9	16,663,283	16,672,397
					PEX5L	9	16,760,382	16,850,020
					USP13	9	16,861,502	16,902,461
					NDUFB5	9	16,903,953	16,907,474
					MRPL47	9	16,907,572	16,911,041
					ACTL6A	9	16,911,951	16,921,482
					GNB4	9	16,972,459	16,979,703
					MFN1	9	16,984,938	17,003,984
					ZNF639	9	17,007,571	17,011,621
					PIK3CA	9	17,020,759	17,042,366
					ZMAT3	9	17,052,618	17,062,023
					NAALADL2	9	17,795,977	17,991,569
					NLGN1	9	18,196,758	18,471,834
					B3GNT5	9	15,777,289	15,778,413
					SOX2	9	16,342,563	16,343,510
2	10,926,468	13,747,682	2,821,214	0.336754469				
					PFKP	2	11,354,185	11,395,733
					PITRM1	2	11,396,672	11,421,465
					GJD4	2	12,778,766	12,787,894
					CCNY	2	12,793,547	12,910,204
					CREM	2	12,930,882	12,960,732
					CUL2	2	12,972,707	13,022,987
					PARD3	2	13,068,736	13,498,548
					FZD8	2	12,761,773	12,762,399
6	26,158,184	29,729,667	3,571,483	0.046164945				
					TECTB	6	26,215,254	26,223,330
					ACSL5	6	26,247,834	26,264,411
					ZDHHC6	6	26,264,533	26,277,485
					VTI1A	6	26,277,903	26,526,700
					TCF7L2	6	26,590,486	26,762,220
					HABP2	6	26,971,704	26,991,533
					NRAP	6	26,991,279	27,037,248
					CASP7	6	27,042,298	27,062,965

					PLEKHS1	6	27,064,759	27,081,909
					DCLRE1A	6	27,087,946	27,102,495
					NHLRC2	6	27,103,400	27,134,851
					C10ORF118	6	27,217,490	27,246,216
					TDRD1	6	27,245,682	27,267,251
					COL9A3	6	27,267,818	27,285,089
					AFAP1L2	6	27,287,975	27,335,710
					ABLIM1	6	27,359,430	27,532,008
					FAM160B1	6	27,536,767	27,571,562
					TRUB1	6	27,594,383	27,622,142
					ATRNL1	6	27,680,834	28,014,799
					GFRA1	6	28,152,303	28,288,081
					CCDC172	6	28,309,882	28,328,718
					HSPA12A	6	28,413,790	28,450,155
					ENO4	6	28,500,759	28,518,682
					KIAA1598	6	28,516,161	28,575,056
					KCNK18	6	28,649,189	28,653,448
					PDZD8	6	28,696,248	28,747,446
					EMX2	6	28,814,903	28,820,582
					RAB11FIP2	6	29,003,777	29,047,647
					FAM204A	6	29,173,024	29,190,310
					C6H10ORF46	6	29,348,791	29,388,352
					EIF3A	6	29,508,820	29,537,182
					FAM45A	6	29,538,376	29,547,379
					SFXN4	6	29,550,386	29,559,570
					PRDX3	6	29,560,387	29,565,670
					ADRB1	6	27,190,009	27,191,226
					NANOS1	6	29,504,247	29,505,285
3	103,727,370	108,395,737	4,668,367	0.00192876				
					MFSD2B	3	103,746,139	103,779,362
					C2orf44	3	103,783,106	103,791,013
					FKBP1B	3	103,866,156	103,869,947
					SF3B14	3	103,873,567	103,877,409
					TP53I3	3	103,877,869	103,881,489
					PFN4	3	103,884,507	103,887,705
					ITSN2	3	103,895,602	103,938,672
					GPN1	3	103,970,332	103,984,905
					ZNF512	3	103,984,631	104,002,721
					FNDC4	3	104,008,233	104,014,124
					IFT172	3	104,023,556	104,057,142
					KRTCAP3	3	104,057,388	104,059,799
					NRBP1	3	104,059,886	104,085,090
					PPM1G	3	104,085,785	104,102,280
					ZNF513	3	104,109,217	104,113,430
					SNX17	3	104,109,900	104,118,369
					EIF2B4	3	104,118,906	104,125,481
					AGBL5	3	104,125,582	104,133,092
					TMEM214	3	104,135,364	104,141,333
					CAD	3	104,164,895	104,174,351
					MPV17	3	104,177,374	104,181,451
					GTF3C2	3	104,206,333	104,213,436
					MAPRE3	3	104,213,713	104,218,289
					DNMT3A	3	104,239,248	104,259,227
					ASXL2	3	104,272,171	104,504,133
					KIF3C	3	104,518,139	104,529,350
					RAB10	3	104,556,702	104,571,975
					GAREML	3	104,575,048	104,582,511
					HADHA	3	104,583,827	104,607,643
					HADHB	3	104,607,849	104,619,357
					EPT1	3	104,645,877	104,668,714
					PTK2B	3	104,676,810	104,700,808
					CHRNA2	3	104,700,299	104,704,881
					EPHX2	3	104,712,779	104,721,569
					SCARA5	3	104,760,091	104,779,321

PBK	3	104,781,669	104,791,026
ESCO2	3	104,790,088	104,800,567
CCDC25	3	104,804,105	104,814,366
SCARA3	3	104,817,333	104,822,180
C2ORF18	3	104,835,719	104,839,201
KCNK3	3	104,844,800	104,855,646
C2orf70	3	104,887,264	104,889,731
CCDC164	3	104,967,445	104,979,487
TRIM35	3	104,980,477	104,985,240
STMN4	3	104,988,422	104,996,116
POMC	3	105,013,957	105,016,899
EFR3B	3	105,019,505	105,046,269
DNAJC27	3	105,062,440	105,072,565
ADCY3	3	105,072,727	105,085,294
CENPO	3	105,085,782	105,092,472
PTRHD1	3	105,092,550	105,100,626
NCOA1	3	105,103,875	105,211,518
ZNF395	3	105,294,830	105,304,932
PNOC	3	105,308,183	105,311,570
ELP3	3	105,336,342	105,420,205
FBXO16	3	105,432,483	105,455,360
FZD3	3	105,466,097	105,513,573
EXTL3	3	105,599,177	105,650,057
INTS9	3	105,654,633	105,723,673
HMBOX1	3	105,781,724	105,832,659
KIF13B	3	105,851,266	105,973,615
MSRA	3	106,054,095	106,314,882
PRSS55	3	106,318,274	106,332,544
RP1L1	3	106,375,249	106,398,770
XKR6	3	106,569,100	106,587,986
MTMR9	3	106,761,333	106,784,678
FAM167A	3	106,838,785	106,848,916
BLK	3	106,862,804	106,898,321
GATA4	3	106,954,748	106,976,693
NEIL2	3	106,981,676	106,984,917
FDFT1	3	106,985,673	106,996,753
CTSB	3	106,998,423	107,007,359
TRAM2	3	107,117,647	107,125,467
EFHC1	3	107,130,488	107,147,698
MCM3	3	107,167,586	107,179,385
PKHD1	3	107,230,220	107,461,810
TFAP2B	3	107,846,265	107,873,218
TFAP2D	3	107,894,065	107,943,864
PAQR8	3	107,155,454	107,156,515

Supplementary table 11e. Multispecies homologous synteny blocks (msHSBs) detected in amniotes

Chr	msHSB start (bp)	msHSB end (bp)	msHSB length (bp)	Probability of being found under the Poisson process	Gene ID	Chr	Gene start (bp)	Gene end (bp)
3	24,475,771	25,985,031	1,509,260	0.999999999				
					PLEKHH2	3	24,491,110	24,545,482
					DYNC2LI1	3	24,548,141	24,570,676
					ABCG5	3	24,567,546	24,588,926
					ABCG8	3	24,585,792	24,598,091
					LRPPRC	3	24,608,804	24,695,385
					PPM1B	3	24,761,763	24,820,317
					SLC3A1	3	24,827,681	24,841,984
					PREPL	3	24,831,731	24,859,714
					SIX2	3	25,240,527	25,243,491
					SRBD1	3	25,432,172	25,549,520
					PRKCE	3	25,576,043	25,856,010
					EPAS1	3	25,951,104	25,982,708
1	10,916,110	12,431,817	1,515,707	0.999999999				
					CD36	1	11,005,425	11,038,504
					GNAT3	1	11,086,584	11,113,407
					GNAI3	1	11,190,979	11,223,596
					MAGI2	1	11,930,694	12,230,824
					PHTF2	1	12,251,576	12,314,104
					TMEM60	1	12,314,478	12,318,704
					RSBN1L	1	12,327,050	12,358,827
					PTPN18	1	12,381,899	12,422,097
5	30,029,578	31,568,709	1,539,131	0.999999997				
					MEIS2	5	30,506,655	30,675,196
					C5H15orf41	5	30,717,442	30,832,671
					ATPBD4	5	31,253,451	31,438,936
					ZNF770	5	31,468,292	31,472,971
					AQR	5	31,481,161	31,528,089
					ACTC1	5	31,546,121	31,552,902
1	132,881,781	134,421,245	1,539,464	0.999999996				
					MAP4K4	1	132,936,573	133,084,109
					IL1R2	1	133,109,396	133,119,021
					IL1RL1	1	133,227,061	133,252,726
					IL18R1	1	133,259,701	133,278,851
					IL18RAP	1	133,283,645	133,298,556
					SLC9A4	1	133,300,715	133,329,117
					SLC9A2	1	133,354,849	133,381,741
					MFSD9	1	133,391,984	133,402,160
					TMEM182	1	133,408,227	133,435,369
20	9,563,535	11,104,188	1,540,653	0.999999997				
					OPRL1	20	9,598,823	9,610,812
					RGS19	20	9,692,144	9,695,672
					TCEA2	20	9,699,117	9,715,280
					SOX18	20	9,728,462	9,733,689
					PRPF6	20	9,759,349	9,783,514
					SAMD10	20	9,790,153	9,799,694
					UCKL1	20	9,837,210	9,851,741
					DNAJC5	20	9,868,316	9,882,697
					SLC2A4RG	20	9,958,400	9,961,363
						20	9,970,349	9,976,884
					TNFRSF6B	20	9,998,639	10,003,173
					STMN3	20	10,049,567	10,060,098
					PDYN	20	10,129,970	10,133,174
					NSFL1C	20	10,152,354	10,159,223
					SDCBP2	20	10,178,536	10,180,514
					PSMF1	20	10,210,994	10,216,774
					RSPO4	20	10,230,159	10,233,326
					ANGPT4	20	10,237,437	10,245,306
					FAM110A	20	10,246,853	10,249,532

					SLC52A3	20	10,254,506	10,257,465
					TCF15	20	10,271,534	10,273,928
					TBC1D20	20	10,304,367	10,310,001
					NRSN2	20	10,309,574	10,313,704
					C20orf96	20	10,320,202	10,326,956
					REM1	20	10,327,399	10,330,347
					HM13	20	10,334,332	10,346,234
					BCL2L1	20	10,359,785	10,377,592
					TPX2	20	10,382,093	10,392,964
					MYLK2	20	10,395,495	10,403,016
					DUSP15	20	10,412,313	10,419,874
					TTLL9	20	10,420,572	10,431,015
					PDRG1	20	10,431,992	10,433,839
					XKR7	20	10,436,888	10,443,076
					C20ORF160	20	10,446,236	10,449,489
					HCK	20	10,451,055	10,456,874
					TM9SF4	20	10,461,799	10,472,166
					PLAGL2	20	10,480,919	10,483,343
					POFUT1	20	10,487,518	10,491,477
					KIF3B	20	10,492,514	10,502,873
					ASXL1	20	10,505,808	10,517,025
					COMMD7	20	10,585,431	10,590,382
					DNMT3B	20	10,595,952	10,609,143
					MAPRE1	20	10,610,165	10,617,328
					TENP	20	10,643,158	10,647,278
					BPIFB6	20	10,649,024	10,653,537
					BPIFB3	20	10,656,808	10,661,438
					BPIFB4	20	10,663,664	10,668,879
					CDK5RAP1	20	10,679,042	10,686,268
					KIAA1755	20	10,700,368	10,707,694
					TGM2	20	10,714,657	10,723,053
					RPRD1B	20	10,726,664	10,747,756
					TTI1	20	10,747,457	10,760,392
					VSTM2L	20	10,761,591	10,764,145
					CTNNBL1	20	10,772,139	10,813,719
					WFDC2	20	10,829,801	10,840,153
					DNTTIP1	20	10,849,414	10,853,705
					UBE2C	20	10,853,789	10,854,730
					TNNC2	20	10,856,323	10,858,120
					SNX21	20	10,859,488	10,861,629
					ACOT8	20	10,859,880	10,864,460
					ZSWIM3	20	10,864,587	10,870,236
					NEURL2	20	10,871,289	10,873,861
					CTSA	20	10,874,294	10,878,191
					PLTP	20	10,878,671	10,881,926
					PCIF1	20	10,887,208	10,895,373
					ZNF335	20	10,900,409	10,909,059
					MMP9	20	10,917,368	10,921,453
					SLC12A5	20	10,922,030	10,944,646
					NCOA5	20	10,950,277	10,963,160
					CD40	20	10,966,821	10,970,503
					CDH22	20	10,990,502	11,029,175
					SLC35C2	20	11,071,603	11,076,564
					ELMO2	20	11,076,802	11,090,032
					NPBWR2	20	9,589,297	9,590,932
					SCRT2	20	10,263,332	10,264,039
2	8,014,363	9,563,687	1,549,324	0.999999994				
					SHH	2	8,080,091	8,089,855
					RNF32	2	8,414,446	8,427,357
					LMBR1	2	8,434,744	8,494,298
					NOM1	2	8,513,775	8,529,642
					MNX1	2	8,538,868	8,541,508
					UBE3C	2	8,582,314	8,644,842
					PTPRN2	2	8,785,742	9,406,733

					NCAPG2	2	9,417,857	9,456,238
					ESYT2	2	9,467,167	9,532,244
15	6,244,107	7,798,721	1,554,614	0.999999992				
					ERP29	15	6,254,850	6,258,922
					NAA25	15	6,259,423	6,286,521
					TRAFD1	15	6,287,966	6,297,369
					HECTD4	15	6,301,498	6,347,392
					RPL6	15	6,377,025	6,380,366
					RPH3A	15	6,429,258	6,443,243
					WSCD2	15	6,458,296	6,473,510
					FICD	15	6,496,565	6,498,936
					SART3	15	6,500,745	6,514,750
					ISCU	15	6,514,991	6,518,217
					CORO1C	15	6,530,372	6,560,293
					SSH1	15	6,570,787	6,598,063
					SVOP	15	6,610,623	6,628,850
					USP30	15	6,631,170	6,642,322
					ALKBH2	15	6,643,016	6,644,963
					ACACB	15	6,650,609	6,669,926
					FOXN4	15	6,671,647	6,674,817
					MYO1H	15	6,712,191	6,726,855
					KCTD10	15	6,729,328	6,739,015
					CRYBB3	15	6,993,056	6,994,735
					CRYBB2	15	6,996,738	6,998,847
					SEZ6L	15	7,123,775	7,135,058
					ASPHD2	15	7,139,418	7,142,644
					HPS4	15	7,144,828	7,156,452
					SRRD	15	7,156,592	7,158,367
					TFIP11	15	7,157,858	7,165,652
					TPST2	15	7,165,841	7,170,715
					CRYBB1	15	7,176,718	7,179,013
					CRYBA4	15	7,181,150	7,182,285
					MN1	15	7,488,965	7,521,455
					PITPNB	15	7,530,400	7,547,053
					TTC28	15	7,554,369	7,671,872
					CHEK2	15	7,678,294	7,690,938
					HSCB	15	7,691,060	7,694,579
					CCDC117	15	7,704,993	7,709,918
					XBP1	15	7,713,031	7,716,156
					ZNRF3	15	7,788,957	7,795,744
					TMEM119	15	6,520,471	6,521,139
3	49,583,623	51,152,885	1,569,262	0.999999985				
					SCAF8	3	49,604,475	49,635,861
					TFB1M	3	49,868,438	49,895,829
					CLDN20	3	49,875,601	49,880,776
					NOX3	3	49,915,188	49,952,437
					ARID1B	3	50,465,764	50,754,522
					TMEM242	3	50,810,196	50,829,319
					SNX9	3	50,997,690	51,052,016
					SYNJ2	3	51,063,895	51,127,835
3	56,963,360	58,590,234	1,626,874	0.99999978				
					EPB41L2	3	56,980,129	57,075,289
					SAMD3	3	57,322,882	57,363,225
					L3MBTL3	3	57,360,442	57,425,789
					C6ORF191	3	57,466,325	57,472,268
					ARHGAP18	3	57,506,532	57,565,989
					LAMA2	3	57,586,334	57,923,906
					PTPRK	3	58,029,862	58,422,557
					THEMIS	3	58,437,710	58,506,141
					C6orf58	3	58,527,005	58,539,770
					TMEM200A	3	57,238,296	57,239,765
z	59,236,376	60,875,956	1,639,580	0.999999661				
4	73,572,127	75,214,999	1,642,872	0.999999579				
					PPARGC1A	4	73,626,292	73,691,855

					GPR125	4	74,015,279	74,072,320
					KCNIP4	4	74,436,584	74,527,405
					PACRGL	4	74,526,045	74,539,587
					SLIT2	4	74,557,149	74,802,699
2	6,187,613	7,833,125	1,645,512	0.999999483				
					WDR86	2	6,203,237	6,221,340
					CRYGN	2	6,235,658	6,239,901
					PRKAG2	2	6,247,476	6,461,068
					GALNT11	2	6,503,696	6,527,983
					MLL3	2	6,528,611	6,717,633
					XRCC2	2	6,755,237	6,767,527
					DPP6	2	7,411,564	7,635,852
					PAXIP1	2	7,658,648	7,692,640
					HTR5A	2	7,711,450	7,714,285
					INSIG1	2	7,827,461	7,832,977
1	109,932,747	111,600,312	1,667,565	0.999998674				
					CXorf36	1	110,128,634	110,143,679
					FUNDC1	1	110,347,436	110,362,439
					EFHC2	1	110,382,985	110,442,596
					NDP	1	110,522,886	110,531,383
					MAOB	1	110,555,374	110,609,289
					MAOA	1	110,613,430	110,658,911
					GPR34	1	111,232,706	111,235,607
					NYX	1	111,334,091	111,336,368
					GPR82	1	111,219,959	111,220,993
2	133,158,961	134,837,147	1,678,186	0.999998206				
					TRPS1	2	134,048,833	134,252,747
					EIF3H	2	134,628,301	134,709,262
					UTP23	2	134,720,381	134,723,977
					RAD21	2	134,749,874	134,773,800
17	2,877,535	4,580,172	1,702,637	0.999995483				
					PAPPA	17	3,040,970	3,209,266
					ASTN2	17	3,219,825	3,482,002
					TLR4	17	3,566,454	3,571,907
					DBC1	17	3,985,882	4,064,290
					CDK5RAP2	17	4,495,102	4,566,562
1	80,304,616	82,024,763	1,720,147	0.999991326				
					LSAMP	1	81,373,015	81,656,000
					GAP43	1	81,680,485	81,734,254
3	58,916,848	60,639,979	1,723,131	0.999991544				
					CENPW	3	59,060,255	59,067,195
					TRMT11	3	59,178,746	59,205,181
					HINT3	3	59,212,941	59,219,743
					NCOA7	3	59,223,034	59,291,522
					HDDC2	3	59,516,400	59,525,907
					TPD52L1	3	59,527,859	59,580,356
					RNF217	3	59,606,826	59,662,599
					NKAIN2	3	59,710,415	59,999,592
					TRDN	3	60,315,841	60,432,093
					CLVS2	3	60,548,727	60,598,139
4	20,823,966	22,548,595	1,724,629	0.999991238				
					GRIA2	4	20,873,025	20,960,836
					FAM198B	4	21,221,107	21,234,597
					TMEM144	4	21,242,338	21,257,193
					RXFP1	4	21,328,064	21,376,386
					ETFDH	4	21,379,598	21,396,998
					PPID	4	21,395,223	21,405,790
					FNIP2	4	21,425,883	21,457,823
					C4ORF45	4	21,455,357	21,496,063
					RAPGEF2	4	21,509,902	21,683,609
					FSTL5	4	22,250,827	22,516,765
5	10,087,655	11,834,285	1,746,630	0.99998262				
					SOX6	5	10,549,851	10,784,968
					C5H11ORF58	5	10,948,695	10,953,363

					PLEKHA7	5	10,959,448	11,057,508
					RPS13	5	11,136,190	11,139,937
					PIK3C2A	5	11,142,129	11,205,960
					NUCB2	5	11,205,875	11,231,516
					KCNJ11	5	11,247,848	11,250,065
					ABCC8	5	11,253,945	11,319,342
					USH1C	5	11,334,156	11,377,576
					OTOG	5	11,381,876	11,476,414
					MYOD1	5	11,578,793	11,582,278
					KCNC1	5	11,597,391	11,714,071
3	65,651,343	67,404,826	1,753,483	0.999977954				
					RPF2	3	65,709,193	65,721,929
					GTF3C6	3	65,721,376	65,726,791
					AMD1	3	65,744,041	65,761,269
					CDK19	3	65,801,524	65,899,585
					SLC22A16	3	65,931,799	65,962,885
					DDO	3	65,969,395	65,975,774
					C6ORF186	3	65,985,390	66,029,579
					CDC40	3	66,033,229	66,074,352
					WASF1	3	66,123,600	66,154,746
					GPR6	3	66,173,387	66,197,130
					FIG4	3	66,201,896	66,268,774
					AK9	3	66,269,578	66,325,155
					ZBTB24	3	66,327,020	66,334,932
					PPIL6	3	66,340,530	66,350,895
					CD164	3	66,357,144	66,365,037
					C6ORF183	3	66,413,334	66,432,788
					CEP57L1	3	66,460,394	66,473,222
					SESN1	3	66,547,545	66,556,746
					ARMC2	3	66,560,205	66,617,959
					FOXO3	3	66,702,483	66,716,393
					LACE1	3	66,809,734	66,870,599
					SNX3	3	66,876,089	66,895,012
					NR2E1	3	66,898,978	66,913,949
					OSTM1	3	66,950,856	66,961,817
					SEC63	3	66,978,001	67,033,488
					SCML4	3	67,042,180	67,098,929
					SOBP	3	67,134,086	67,243,457
					PDSS2	3	67,259,779	67,378,577
					BEND3	3	67,393,979	67,403,976
4	23,247,017	25,022,794	1,775,777	0.999958092				
					KLHL2	4	23,265,508	23,325,234
					SC4MOL	4	23,326,894	23,333,143
					CPE	4	23,336,496	23,382,106
					TLL1	4	23,520,463	23,656,666
					SPOCK3	4	23,947,374	24,051,813
					ANXA10	4	24,400,386	24,416,023
					PALLD	4	24,486,948	24,675,736
					AADAT	4	24,691,532	24,707,952
					MFAP3L	4	24,743,867	24,747,796
					C4ORF27	4	24,796,835	24,803,472
					CLCN3	4	24,804,714	24,864,749
					NEK1	4	24,866,404	24,909,512
					CBR4	4	25,012,882	25,021,145
2	120,455,577	122,248,807	1,793,230	0.999929309				
					STMN2	2	120,505,360	120,515,753
					HEY1	2	120,538,764	120,541,690
					TPD52	2	120,668,873	120,704,236
					ZBTB10	2	120,783,328	120,809,194
					ZNF704	2	120,881,814	120,900,516
					PAG1	2	120,988,402	121,062,954
					FABP5	2	121,148,557	121,153,615
					PMP2	2	121,215,806	121,220,355
					FABP4	2	121,225,723	121,228,944

					IMPA1	2	121,262,379	121,277,486
					ZFAND1	2	121,279,568	121,285,857
					CHMP4C	2	121,287,839	121,304,205
					SNX16	2	121,309,189	121,333,508
3	35,427,107	37,224,095	1,796,988	0.999923472				
					GREM2	3	35,493,725	35,533,009
					FMN2	3	35,540,378	35,680,173
					CHRM3	3	35,727,538	35,877,494
					RYR2	3	36,498,791	36,661,068
					MTR	3	36,890,573	36,933,337
					ACTN2	3	36,948,211	37,014,188
					HEATR1	3	37,025,129	37,060,227
					LGALS8	3	37,061,029	37,073,707
					EDARADD	3	37,078,799	37,097,401
					ERO1LB	3	37,097,636	37,134,158
					GPR137B	3	37,135,755	37,159,463
					NID1	3	37,178,196	37,213,970
3	45,246,962	47,049,757	1,802,795	0.999910911				
					EPM2A	3	45,769,623	45,811,385
					FBXO30	3	45,824,455	45,831,133
					SHPRH	3	45,868,769	45,921,436
					GRM1	3	45,937,157	46,118,743
					RAB32	3	46,154,328	46,173,538
					STXBP5	3	46,388,675	46,489,008
					SAMD5	3	46,522,593	46,647,011
21	4,300,346	6,125,413	1,825,067	0.999840508				
					ZBTB17	21	4,302,289	4,306,459
					EPHA2	21	4,318,355	4,326,236
					FBXO42	21	4,331,104	4,371,111
					SZRD1	21	4,372,845	4,385,290
					NECAP2	21	4,385,803	4,391,455
						21	4,392,909	4,410,627
					MFAP2	21	4,405,466	4,410,649
					ATP13A2	21	4,414,089	4,422,658
					PAX-7	21	4,442,610	4,530,774
					EMC1	21	4,656,681	4,666,647
					MRTO4	21	4,666,777	4,668,928
					PQLC2	21	4,673,047	4,678,165
					CAPZB	21	4,679,337	4,732,155
					MINOS1	21	4,740,500	4,754,491
					HTR6	21	4,776,632	4,780,155
					RNF186	21	4,822,545	4,823,109
					OTUD3	21	4,825,556	4,833,537
					PLA2G2E	21	4,835,306	4,836,901
					PLA2G2A	21	4,844,039	4,845,148
					UBXN10	21	4,846,209	4,848,286
					MUL1	21	4,885,335	4,889,550
					FAM43B	21	4,890,645	4,891,820
					CDA	21	4,893,462	4,897,243
					PINK1	21	4,897,971	4,906,221
					AGMAT	21	4,906,433	4,908,768
					DNAJC16	21	4,909,535	4,918,231
					CASP9	21	4,918,316	4,924,583
					CTRC	21	4,925,271	4,927,174
					EFHD2	21	4,927,440	4,930,207
					FHAD1	21	4,931,027	4,938,907
					TMEM51	21	4,949,174	4,951,265
					PRDM2	21	5,113,479	5,177,785
					PDPN	21	5,184,890	5,203,054
					LRRC38	21	5,217,683	5,230,938
					C21H1orf158	21	5,253,356	5,257,720
					DHRS3	21	5,316,086	5,340,991
					VPS13D	21	5,362,132	5,448,582
					TNFRSF1B	21	5,451,978	5,465,671

					TNFRSF8	21	5,468,760	5,481,849
					MIIP	21	5,491,938	5,499,769
					MFN2	21	5,503,597	5,513,761
					PLOD1	21	5,514,083	5,531,209
					KIAA2013	21	5,530,908	5,536,991
					NPPA	21	5,577,062	5,579,681
					CLCN6	21	5,588,806	5,603,988
					MTHFR	21	5,606,164	5,615,523
					AGTRAP	21	5,632,718	5,640,376
					DRAXIN	21	5,645,770	5,657,774
					MAD2L2	21	5,659,446	5,662,213
					FBXO2	21	5,667,616	5,670,436
					DISP3	21	5,721,500	5,736,965
					LACTBL1	21	5,868,247	5,876,675
					EPHB2	21	5,941,759	6,038,880
					C1QB	21	6,058,692	6,063,086
					C1QA	21	6,064,485	6,066,427
2	137,878,625	139,781,764	1,903,139	0.999096934				
					TMEM65	2	137,986,349	138,016,847
					TATDN1	2	138,058,219	138,071,047
					NDUFB9	2	138,071,064	138,074,938
					MTSS1	2	138,077,064	138,193,553
					SQLE	2	138,265,073	138,279,203
					KIAA0196	2	138,283,684	138,306,583
					NSMCE2	2	138,307,230	138,433,695
					TRIB1	2	138,436,990	138,444,511
					FAM84B	2	138,873,014	138,876,023
					MYC	2	139,316,928	139,321,894
					RNF139	2	138,055,501	138,057,312
1	141,339,838	143,294,319	1,954,481	0.997558457				
					SLC10A2	1	141,914,133	141,925,460
					BIVM	1	142,024,553	142,041,190
					KDELC1	1	142,045,388	142,054,410
					C13ORF27	1	142,057,711	142,064,431
					METTL21C	1	142,092,496	142,099,273
					TPP2	1	142,097,596	142,147,887
					ITGBL1	1	142,614,261	142,752,083
					NALCN	1	142,765,240	142,969,863
					TMTC4	1	143,142,401	143,197,059
					A2LD1	1	143,230,157	143,230,654
1	145,101,546	147,118,880	2,017,334	0.993500265				
					UGGT2	1	145,103,089	145,180,354
					DNAJC3	1	145,184,466	145,216,297
					DZIP1	1	145,229,679	145,262,325
					CLDN10	1	145,266,284	145,279,564
					ABCC4	1	145,358,360	145,506,681
					GPR180	1	145,671,022	145,692,774
					TGDS	1	145,692,790	145,705,488
					DCT	1	145,716,342	145,737,439
2	112,226,473	114,277,051	2,050,578	0.990137618				
					CA8	2	112,413,504	112,453,419
					RAB2A	2	112,517,303	112,558,232
					CHD7	2	112,612,964	112,699,976
					CLVS1	2	112,882,281	112,978,767
					NKAIN3	2	113,137,211	113,469,039
					GGH	2	113,490,054	113,501,161
					TTPA	2	113,502,155	113,517,713
					YTHDF3	2	113,543,309	113,551,391
					CYP7B1	2	114,093,263	114,114,645
13	813,059	2,865,235	2,052,176	0.989694448				
					PFDN1	13	928,207	957,649
					HBEGF	13	961,553	967,753
					SLC4A9	13	972,298	989,098
					SRA1	13	1,095,133	1,095,980

					APBB3	13	1,096,712	1,100,458
					PCDHGC3	13	1,217,194	1,247,116
					HDAC3	13	1,277,095	1,290,277
					RELL2	13	1,292,499	1,299,951
					FCHSD1	13	1,300,987	1,305,466
					ARAP3	13	1,309,826	1,324,899
					PCDH1	13	1,408,177	1,457,586
					PCDH12	13	1,751,168	1,760,560
					ECSCR	13	1,767,729	1,785,069
					DNAJC18	13	1,788,284	1,799,938
					MZB1	13	1,806,725	1,808,111
					SLC23A1	13	1,808,718	1,812,108
					PAIP2	13	1,813,947	1,819,364
					MATR3	13	1,825,981	1,851,149
					SIL1	13	1,869,523	1,957,625
					CTNNA1	13	1,960,516	2,068,702
					LRRTM2	13	2,010,626	2,012,530
					HSPA9	13	2,134,785	2,156,387
					ETF1	13	2,158,826	2,184,873
					FBXW11	13	2,208,007	2,258,082
					FGF18	13	2,567,435	2,611,832
					NPM1	13	2,641,231	2,651,587
					TLX3	13	2,673,915	2,675,387
					RANBP17	13	2,681,554	2,828,621
					PURA	13	860,147	860,890
2	125,160,507	127,244,400	2,083,893	0.98508674				
					TRIQK	2	125,273,982	125,333,683
					FAM92A1	2	125,604,624	125,621,196
					RBM12B	2	125,623,603	125,630,097
					TMEM67	2	125,632,568	125,659,384
					PDP1	2	125,683,595	125,690,722
					CDH17	2	125,754,667	125,783,019
					GEM	2	125,802,915	125,811,572
					RAD54B	2	125,844,098	125,903,394
					FSBP	2	125,877,035	125,887,013
					KIAA1429	2	125,925,066	125,952,171
					ESRP1	2	125,956,620	125,988,739
					DPY19L4	2	126,052,840	126,076,741
					INTS8	2	126,126,452	126,151,324
					CCNE2	2	126,151,226	126,161,848
					TP53INP1	2	126,170,346	126,183,360
					NDUFAF6	2	126,201,059	126,216,326
					PLEKHF2	2	126,225,096	126,240,839
					C8orf37	2	126,258,109	126,269,454
					MTERFD1	2	126,597,212	126,615,684
					PTDSS1	2	126,616,011	126,645,632
					SDC2	2	126,740,060	126,929,143
					MTDH	2	127,057,053	127,088,247
					LAPTM4B	2	127,102,614	127,165,676
					MATN2	2	127,183,885	127,242,408
2	1,811,213	3,915,269	2,104,056	0.980957711				
					SEC22C	2	1,886,499	1,900,914
					NKTR	2	1,927,422	1,965,795
					ZBTB47	2	1,978,851	1,993,362
					KBTBD5	2	1,999,758	2,009,597
					HHATL	2	2,014,408	2,026,554
					CCDC13	2	2,032,322	2,059,811
					HIGD1C	2	2,061,655	2,066,620
					CCBP2	2	2,072,000	2,073,926
					OBSCN	2	2,080,439	2,253,956
					C2H1ORF69	2	2,295,676	2,300,126
					GJC2	2	2,303,738	2,310,415
					GUK1	2	2,334,607	2,343,799
					MRPL55	2	2,348,731	2,351,041

					C1ORF35	2	2,351,366	2,358,790
					ARF1	2	2,357,324	2,369,250
					WNT3A	2	2,459,534	2,542,486
					WNT9A	2	2,573,612	2,624,503
					SNAP47	2	3,185,315	3,205,912
					JMJD4	2	3,211,699	3,220,712
					ALS2CL	2	3,216,850	3,246,773
					TMIE	2	3,268,001	3,297,394
					MYL3	2	3,420,143	3,455,225
					PTH1R	2	3,560,824	3,688,454
					CCDC12	2	3,804,742	3,838,168
					NBEAL2	2	3,841,475	3,864,815
1	172,850,666	174,964,633	2,113,967	0.978348235				
					RFC3	1	173,144,179	173,161,079
					STARD13	1	173,241,712	173,493,625
					KL	1	173,504,524	173,551,941
					PDS5B	1	173,655,059	173,730,298
					N4BP2L1	1	173,808,326	173,820,489
					BRCA2	1	173,820,724	173,857,456
					ZAR1	1	173,859,596	173,861,411
					FRY	1	173,863,589	174,002,319
					B3GALTL	1	174,203,173	174,247,847
					HSPH1	1	174,262,982	174,286,406
					ALOX5AP	1	174,335,143	174,344,760
					USPL1	1	174,350,051	174,363,188
					KATNAL1	1	174,496,738	174,524,653
					UBL3	1	174,588,070	174,645,255
					SLC7A1	1	174,687,111	174,726,955
2	102,945,374	105,067,889	2,122,515	0.976922154				
					OSBPL1A	2	102,948,952	103,020,897
					IMPACT	2	103,033,304	103,051,363
					ZNF521	2	103,383,680	103,410,706
					SS18	2	103,745,620	103,788,130
					TAF4B	2	103,821,689	103,883,809
					KCTD1	2	103,913,222	103,977,131
					AQP4	2	104,100,790	104,109,628
					CHST9	2	104,126,305	104,127,381
					CDH2	2	104,449,184	104,562,821
9	20,585,078	22,714,790	2,129,712	0.976197459				
					SLITRK3	9	20,586,079	20,587,887
					OTOL1	9	21,401,027	21,404,693
					SPTSSB	9	21,417,214	21,425,710
					NMD3	9	21,436,129	21,447,358
					PPM1L	9	21,476,302	21,557,159
					KPNA4	9	21,597,000	21,610,445
					SMC4	9	21,626,305	21,657,730
					IFT80	9	21,661,987	21,703,750
					IL12A	9	21,727,352	21,729,152
					IQCJ	9	21,810,250	21,844,816
					MFSD1	9	21,872,165	21,885,523
					RARRES1	9	21,904,934	21,912,426
					GFM1	9	21,913,507	21,933,383
					LXN	9	21,919,321	21,923,817
					MLF1	9	21,936,183	21,948,704
					RSRC1	9	21,958,768	22,066,377
					VEPH1	9	22,173,837	22,221,855
					PTX3	9	22,180,190	22,186,891
					CCNL1	9	22,236,549	22,248,075
					LEKR1	9	22,281,614	22,298,405
					TIPARP	9	22,313,993	22,346,827
					SSR3	9	22,347,715	22,354,679
					GMPS	9	22,393,885	22,417,726
					SLC33A1	9	22,418,561	22,429,141
					C3orf33	9	22,430,628	22,434,432

					MME	9	22,527,165	22,563,563
					GPR149	9	22,627,534	22,648,732
					DHX36	9	22,652,324	22,670,920
					B3GALNT1	9	21,566,727	21,567,710
					ARL14	9	21,572,455	21,573,027
1	134,451,427	136,591,630	2,140,203	0.971463071				
					C1H2ORF49	1	134,481,595	134,496,274
					FHL2	1	134,496,864	134,536,397
					NCK2	1	134,633,927	134,714,716
					C2ORF40	1	134,771,683	134,776,149
					ST6GAL2	1	135,007,544	135,053,400
					SLC5A7	1	135,397,400	135,423,764
					SULT1C	1	135,521,670	135,527,319
					RANBP2	1	135,763,887	135,793,418
					EDAR	1	135,819,209	135,882,851
					UPF3A	1	136,254,098	136,279,867
					CDC16	1	136,281,362	136,301,395
					RASA3	1	136,345,769	136,476,626
					GAS6	1	136,509,160	136,547,639
					SOWAHC	1	136,196,812	136,198,516
					CHAMP1	1	136,241,415	136,244,261
4	42,697,452	44,869,083	2,171,631	0.912421874				
					HMGB2	4	42,740,630	42,743,052
					SAP30	4	42,754,695	42,760,373
					SCRG1	4	42,763,397	42,769,576
					FBXO8	4	43,131,548	43,148,365
					CEP44	4	43,149,107	43,165,568
					HPGD	4	43,211,798	43,236,772
					GLRA3	4	43,301,385	43,363,404
					GPM6A	4	43,565,210	43,666,838
					WDR17	4	43,727,567	43,765,630
					SPATA4	4	43,766,435	43,773,179
					ASB5	4	43,775,824	43,808,347
					SPCS3	4	43,812,168	43,817,012
					VEGFC	4	43,882,837	43,954,986
					MTHFD2L	4	44,089,931	44,115,535
					EPGN	4	44,118,458	44,127,174
					EREG	4	44,141,004	44,149,715
					USO1	4	44,202,175	44,228,430
					G3BP2	4	44,236,819	44,252,964
					BMP2K	4	44,313,412	44,349,074
					PAQR3	4	44,355,867	44,361,796
					ANTXR2	4	44,658,935	44,721,258
					PRDM8	4	44,734,247	44,737,047
					FGF5	4	44,740,274	44,743,143
1	164,873,249	167,082,726	2,209,477	0.88663329				
					PCDH8	1	164,995,573	165,000,858
					LECT1	1	165,018,166	165,029,891
					SUGT1	1	165,034,517	165,057,072
					ELF1	1	165,058,991	165,145,844
					WBP4	1	165,145,975	165,167,892
					MTRF1	1	165,172,543	165,185,717
					NAA16	1	165,191,023	165,255,094
					RGCC	1	165,264,942	165,276,995
					VWA8	1	165,302,186	165,480,795
					DGKH	1	165,513,387	165,674,975
					AKAP11	1	165,702,876	165,738,647
					TNFSF11	1	165,817,349	165,839,120
					EPSTI1	1	165,967,144	166,019,257
					DNAJC15	1	166,031,302	166,055,788
					ENOX1	1	166,088,091	166,385,249
					CCDC122	1	166,476,400	166,486,181
					LACC1	1	166,492,231	166,512,495
					TSC22D1	1	166,717,963	166,796,518

					NUFIP1	1	166,904,655	166,927,469
					KIAA1704	1	166,927,078	166,942,984
					GTF2F2	1	166,965,970	167,057,679
					TPT1	1	167,058,420	167,066,619
					KCTD4	1	166,995,127	166,995,900
3	77,406,587	79,690,855	2,284,268	0.825310556				
					TPBG	3	77,636,164	77,637,640
					IBTK	3	77,659,755	77,717,628
					FAM46A	3	77,839,636	77,873,063
					BCKDHB	3	78,457,902	78,570,667
					TTK	3	78,612,134	78,643,792
					ELOVL4	3	78,677,336	78,713,537
					SH3BGRL2	3	78,756,279	78,772,633
					LCA5	3	78,816,727	78,828,429
					HMGN3	3	78,877,354	78,901,407
					PHIP	3	78,926,379	79,016,040
					IRAK1BP1	3	79,040,637	79,051,664
					HTR1B	3	79,492,220	79,493,385
6	23,049,412	25,352,313	2,302,901	0.808164151				
						6	23,104,676	23,106,678
					PCGF6	6	23,110,411	23,132,990
					TAF5	6	23,133,668	23,144,709
					USMG5	6	23,144,647	23,148,454
					PDCD11	6	23,149,107	23,171,490
					CALHM2	6	23,175,699	23,177,288
					CALHM1	6	23,181,808	23,183,651
					CALHM3	6	23,187,673	23,189,859
					NEURL	6	23,201,517	23,337,251
					SH3PXD2A	6	23,349,107	23,406,139
					OBFC1	6	23,585,345	23,623,213
					SLK	6	23,623,564	23,666,255
					COL17A1	6	23,670,969	23,706,526
					SFR1	6	23,730,903	23,734,477
					WDR96	6	23,736,120	23,778,750
					CCDC147	6	23,826,509	23,881,678
					SORCS3	6	23,968,010	24,235,717
					SORCS1	6	24,664,740	24,940,362
					XPNPEP1	6	25,219,899	25,248,494
					ADD3	6	25,264,735	25,352,280
					ITPRIP	6	23,801,103	23,802,752
7	30,893,512	33,294,488	2,400,976	0.709185575				
					SPOPL	7	30,896,072	30,910,605
					NXPH2	7	30,928,491	30,961,286
					KYNU	7	32,143,918	32,202,554
					ARHGAP15	7	32,211,979	32,530,918
					GTDC1	7	32,617,757	32,782,028
					ZEB2	7	32,812,269	32,916,777
z	42,028,444	44,533,600	2,505,156	0.5963927				
11	3,163,524	5,711,059	2,547,535	0.550823662				
					MMP2	11	3,182,494	3,220,410
					IRX5	11	3,356,406	3,358,310
					FTO	11	4,129,194	4,353,272
					RPGRIP1L	11	4,357,663	4,419,746
					AKTIP	11	4,456,387	4,469,147
					RBL2	11	4,474,789	4,490,821
					CHD9	11	4,503,008	4,563,980
					TOX3	11	4,752,569	4,824,785
					SALL1	11	5,418,071	5,433,815
					CYLD	11	5,636,333	5,654,522
					SNX20	11	5,663,396	5,670,064
1	159,057,813	163,199,617	4,141,804	0.007114146				
					PCDH20	1	161,176,976	161,182,699
					TDRD3	1	161,506,641	161,608,657
					DIAPH3	1	161,696,699	161,932,705

PCDH17	1	163,083,292	163,083,448
