# Supplementary material for: Novel Insights into Chromosome Evolution in Birds, Archosaurs, and Reptiles
Source: Genome Biol Evol. 2016 Jul 10;8(8):2442–51. doi: 10.1093/gbe/evw166 (PMC5010900; doi:10.1093/gbe/evw166)
Supplement: Supplementary Data [file supp_evw166_SupplementaryTable6_EBRs_coord.pdf]

Supplementary Table 6. Evolutionary Breakpoint Regions detected at 100Kbp, 300Kbp, and 500Kbp resolutions.

Reference genome: resolution	Ref. chr.	Final classification	EBR start (bp)	EBR end (bp)	Class
chicken:100K	1	meleagris_gallopavo	423,781	426,953	Unique
chicken:100K	1	meleagris_gallopavo	797,271	805,118	Unique
chicken:100K	1	geospiza_fortis	1,125,949	1,145,018	Unique
chicken:100K	1	falco_peregrinus	1,323,380	1,326,161	Unique
chicken:100K	1	chrysemys_picta	1,519,997	1,539,398	Unique
chicken:100K	1	chrysemys_picta	2,837,168	2,862,585	Unique
chicken:100K	1	anolis_carolinensis	4,281,085	4,285,281	Unique
chicken:100K	1	chrysemys_picta	4,507,338	4,516,008	Unique
chicken:100K	1	meleagris_gallopavo	5,510,025	5,513,465	Unique
chicken:100K	1	melopsittacus_undulatus	6,022,379	6,028,148	Unique
chicken:100K	1	falco_peregrinus	6,029,336	6,031,512	Unique
chicken:100K	1	struthio_camelus:anolis_carolinensis	7,212,487	7,232,548	Reuse
chicken:100K	1	anolis_carolinensis:struthio_camelus	7,212,487	7,232,548	Reuse
chicken:100K	1	falco_peregrinus	7,912,880	7,915,408	Unique
chicken:100K	1	corvus_brachyrhynchos	8,088,667	8,091,323	Unique
chicken:100K	1	corvus_brachyrhynchos	8,228,692	8,232,412	Unique
chicken:100K	1	chrysemys_picta	8,347,267	8,351,964	Unique
chicken:100K	1	nipponia_nippon	8,408,427	8,410,084	Unique
chicken:100K	1	meleagris_gallopavo	9,283,764	9,299,189	Unique
chicken:100K	1	meleagris_gallopavo	9,413,432	9,417,296	Unique
chicken:100K	1	falco_peregrinus	9,887,440	9,888,690	Unique
chicken:100K	1	anolis_carolinensis	9,926,144	9,951,090	Unique
chicken:100K	1	struthio_camelus	10,911,607	10,916,109	Unique
chicken:100K	1	chaetura_pelagica	12,726,035	12,729,399	Unique
chicken:100K	1	picoides_pubescens	13,220,907	13,245,570	Unique
chicken:100K	1	chrysemys_picta	13,667,222	13,692,694	Unique
chicken:100K	1	picoides_pubescens	13,958,731	14,018,386	Unique
chicken:100K	1	opossum:chrysemys_picta	14,766,915	14,823,071	Reuse
chicken:100K	1	chrysemys_picta:opossum	14,766,915	14,823,071	Reuse
chicken:100K	1	chrysemys_picta	16,259,496	16,305,460	Unique
chicken:100K	1	meleagris_gallopavo	16,830,812	16,840,330	Unique
chicken:100K	1	meleagris_gallopavo	16,945,822	16,953,389	Unique
chicken:100K	1	meleagris_gallopavo	17,063,980	17,064,422	Unique
chicken:100K	1	meleagris_gallopavo	17,879,617	17,883,307	Unique
chicken:100K	1	meleagris_gallopavo	18,012,978	18,062,524	Unique
chicken:100K	1	melopsittacus_undulatus	18,411,634	18,414,857	Unique
chicken:100K	1	anolis_carolinensis	18,447,833	18,490,318	Unique
chicken:100K	1	struthio_camelus	19,429,651	19,433,176	Unique
chicken:100K	1	melopsittacus_undulatus	19,576,458	19,581,478	Unique
chicken:100K	1	melopsittacus_undulatus	19,744,403	19,747,900	Unique
chicken:100K	1	anolis_carolinensis	19,827,439	19,856,431	Unique
chicken:100K	1	struthio_camelus	20,523,498	20,525,912	Unique
chicken:100K	1	picoides_pubescens	21,930,965	21,937,064	Unique
chicken:100K	1	anolis_carolinensis	22,853,844	22,995,987	Unique
chicken:100K	1	anas_platyrhynchos	23,926,371	23,927,139	Unique
chicken:100K	1	chinese_alligator	24,545,365	24,573,683	Unique
chicken:100K	1	chinese_alligator:boa_constrictor	25,050,656	25,053,325	Reuse
chicken:100K	1	boa_constrictor:chinese_alligator	25,050,656	25,053,325	Reuse
chicken:100K	1	melopsittacus_undulatus	25,804,207	25,806,627	Unique
chicken:100K	1	pygoscelis_adeliae	26,026,802	26,031,490	Unique
chicken:100K	1	chrysemys_picta	26,537,742	26,548,517	Unique
chicken:100K	1	falco_peregrinus	26,809,315	26,817,163	Unique
chicken:100K	1	melopsittacus_undulatus	28,694,712	28,701,534	Unique
chicken:100K	1	melopsittacus_undulatus	28,948,371	28,950,816	Unique
chicken:100K	1	aptenodytes_forsteri	29,059,748	29,062,983	Unique
chicken:100K	1	chrysemys_picta	32,422,225	32,432,105	Unique
chicken:100K	1	cuculus_canorus	33,346,065	33,357,377	Unique
chicken:100K	1	picoides_pubescens:melopsittacus_undulatus	33,803,844	33,809,977	Reuse
chicken:100K	1	melopsittacus_undulatus:picoides_pubescens	33,803,844	33,809,977	Reuse
chicken:100K	1	anolis_carolinensis	34,037,387	34,061,098	Unique

chicken:100K	1	opossum:chinese_alligator	34,367,220	34,471,821	Reuse
chicken:100K	1	chinese_alligator:opossum	34,367,220	34,471,821	Reuse
chicken:100K	1	chrysemys_picta:chinese_alligator	34,668,164	34,677,307	Reuse
chicken:100K	1	chinese_alligator:chrysemys_picta	34,668,164	34,677,307	Reuse
chicken:100K	1	opossum:chinese_alligator	35,010,646	35,011,903	Reuse
chicken:100K	1	chinese_alligator:opossum	35,010,646	35,011,903	Reuse
chicken:100K	1	anolis_carolinensis	35,789,042	35,790,603	Unique
chicken:100K	1	melopsittacus_undulatus	36,330,037	36,335,101	Unique
chicken:100K	1	chrysemys_picta	37,335,309	37,343,883	Unique
chicken:100K	1	melopsittacus_undulatus	37,370,851	37,373,049	Unique
chicken:100K	1	chinese_alligator:calypte_anna	37,684,432	37,950,492	Reuse
chicken:100K	1	calypte_anna:chinese_alligator	37,684,432	37,950,492	Reuse
chicken:100K	1	picoides_pubescens	38,048,829	38,057,719	Unique
chicken:100K	1	charadrius_vociferus	38,075,586	38,080,977	Unique
chicken:100K	1	columba_livia	38,301,854	38,369,907	Unique
chicken:100K	1	picoides_pubescens	38,852,366	38,902,866	Unique
chicken:100K	1	anolis_carolinensis	39,188,146	39,223,463	Unique
chicken:100K	1	chrysemys_picta	39,271,206	39,282,534	Unique
chicken:100K	1	picoides_pubescens	39,388,194	39,393,279	Unique
chicken:100K	1	corvus_brachyrhynchos:chinese_alligator	39,614,591	39,615,487	Reuse
chicken:100K	1	chinese_alligator:corvus_brachyrhynchos	39,614,591	39,615,487	Reuse
chicken:100K	1	falco_peregrinus	39,765,373	39,767,039	Unique
chicken:100K	1	anas_platyrhynchos	40,082,741	40,090,886	Unique
chicken:100K	1	picoides_pubescens	40,120,353	40,125,728	Unique
chicken:100K	1	meleagris_gallopavo	40,165,675	40,174,839	Unique
chicken:100K	1	meleagris_gallopavo	40,395,952	40,396,175	Unique
chicken:100K	1	nipponia_nippon	40,806,714	40,810,675	Unique
chicken:100K	1	meleagris_gallopavo	41,240,313	41,241,582	Unique
chicken:100K	1	picoides_pubescens	41,514,440	41,521,301	Unique
chicken:100K	1	meleagris_gallopavo	41,602,310	41,751,941	Unique
chicken:100K	1	meleagris_gallopavo	42,134,726	42,138,808	Unique
chicken:100K	1	chrysemys_picta	42,323,195	42,364,573	Unique
chicken:100K	1	picoides_pubescens	44,018,146	44,025,385	Unique
chicken:100K	1	picoides_pubescens	44,421,424	44,426,650	Unique
chicken:100K	1	melopsittacus_undulatus	44,579,230	44,581,535	Unique
chicken:100K	1	aptenodytes_forsteri	45,012,081	45,015,014	Unique
chicken:100K	1	picoides_pubescens	45,381,685	45,384,307	Unique
chicken:100K	1	melopsittacus_undulatus	46,671,762	46,673,244	Unique
chicken:100K	1	calypte_anna	46,987,355	47,063,550	Unique
chicken:100K	1	cuculus_canorus:columba_livia	47,070,875	47,073,188	Reuse
chicken:100K	1	columba_livia:cuculus_canorus	47,070,875	47,073,188	Reuse
chicken:100K	1	chinese_alligator	47,107,494	47,294,066	Unique
chicken:100K	1	opossum:falco_peregrinus	47,570,007	47,572,106	Reuse
chicken:100K	1	falco_peregrinus:opossum	47,570,007	47,572,106	Reuse
chicken:100K	1	cuculus_canorus:columba_livia	47,775,936	47,778,704	Reuse
chicken:100K	1	columba_livia:cuculus_canorus	47,775,936	47,778,704	Reuse
chicken:100K	1	anas_platyrhynchos	47,905,042	47,906,033	Unique
chicken:100K	1	anolis_carolinensis	48,103,016	48,107,037	Unique
chicken:100K	1	meleagris_gallopavo	48,609,502	48,610,293	Unique
chicken:100K	1	chrysemys_picta	48,816,763	48,822,539	Unique
chicken:100K	1	anolis_carolinensis	49,068,545	49,154,316	Unique
chicken:100K	1	opossum:anas_platyrhynchos:chinese_alligator	49,275,117	49,295,547	Reuse
chicken:100K	1	chinese_alligator:opossum:anas_platyrhynchos	49,275,117	49,295,547	Reuse
chicken:100K	1	anas_platyrhynchos:chinese_alligator:opossum	49,275,117	49,295,547	Reuse
chicken:100K	1	anas_platyrhynchos	49,628,455	49,650,033	Unique
chicken:100K	1	anas_platyrhynchos	49,799,759	49,801,747	Unique
chicken:100K	1	anas_platyrhynchos	49,940,034	49,944,301	Unique
chicken:100K	1	chinese_alligator:charadrius_vociferus	50,106,803	50,554,275	Reuse
chicken:100K	1	charadrius_vociferus:chinese_alligator	50,106,803	50,554,275	Reuse
chicken:100K	1	anolis_carolinensis	50,731,151	50,738,392	Unique
chicken:100K	1	chinese_alligator	50,860,981	50,868,337	Unique
chicken:100K	1	anolis_carolinensis	50,989,729	50,991,934	Unique
chicken:100K	1	chinese_alligator	51,374,318	51,376,713	Unique
chicken:100K	1	meleagris_gallopavo	51,597,899	51,598,769	Unique
chicken:100K	1	chinese_alligator	51,683,030	51,698,191	Unique
chicken:100K	1	melopsittacus_undulatus	51,999,326	52,006,775	Unique

chicken:100K	1	nipponia_nippon	52,181,801	52,182,602	Unique
chicken:100K	1	pygoscelis_adeliae	52,234,403	52,239,417	Unique
chicken:100K	1	cuculus_canorus	52,836,116	52,976,300	Unique
chicken:100K	1	melopsittacus_undulatus	53,246,928	53,251,145	Unique
chicken:100K	1	columba_livia	54,851,095	54,854,430	Unique
chicken:100K	1	geospiza_fortis	54,881,392	54,891,581	Unique
chicken:100K	1	anolis_carolinensis	56,484,852	56,488,897	Unique
chicken:100K	1	passeroidea	56,527,911	56,530,955	Unique
chicken:100K	1	corvus_brachyrhynchos	56,534,882	56,538,791	Unique
chicken:100K	1	taeniopygia_guttata:opossum	56,811,292	56,816,501	Reuse
chicken:100K	1	opossum:taeniopygia_guttata	56,811,292	56,816,501	Reuse
chicken:100K	1	anolis_carolinensis	57,664,886	57,680,258	Unique
chicken:100K	1	meleagris_gallopavo	57,814,614	57,815,867	Unique
chicken:100K	1	meleagris_gallopavo	57,994,820	58,034,841	Unique
chicken:100K	1	melopsittacus_undulatus	58,166,174	58,171,797	Unique
chicken:100K	1	columba_livia	58,475,637	58,476,339	Unique
chicken:100K	1	opossum:cuculus_canorus	58,564,257	58,573,569	Reuse
chicken:100K	1	cuculus_canorus:opossum	58,564,257	58,573,569	Reuse
chicken:100K	1	chrysemys_picta	59,344,475	59,350,658	Unique
chicken:100K	1	picoides_pubescens	59,805,225	59,839,027	Unique
chicken:100K	1	passeroidea + corvoidea	60,038,442	60,044,624	Unique
chicken:100K	1	aptenodytes_forsteri	60,568,105	60,570,116	Unique
chicken:100K	1	ophisthocomus_hoazin	61,924,677	61,926,780	Unique
chicken:100K	1	manacus_vitellinus	62,204,668	62,215,162	Unique
chicken:100K	1	calypte_anna	62,229,438	62,234,790	Unique
chicken:100K	1	columba_livia	62,272,341	62,287,957	Unique
chicken:100K	1	melopsittacus_undulatus	62,317,314	62,324,002	Unique
chicken:100K	1	meleagris_gallopavo:anolis_carolinensis	62,674,073	62,765,188	Reuse
chicken:100K	1	anolis_carolinensis:meleagris_gallopavo	62,674,073	62,765,188	Reuse
chicken:100K	1	meleagris_gallopavo	62,990,975	62,992,771	Unique
chicken:100K	1	struthio_camelus	63,062,564	63,065,493	Unique
chicken:100K	1	struthio_camelus	63,240,303	63,243,560	Unique
chicken:100K	1	anolis_carolinensis	63,948,062	63,961,828	Unique
chicken:100K	1	chrysemys_picta	64,818,138	64,834,488	Unique
chicken:100K	1	anas_platyrhynchos	65,315,081	65,317,342	Unique
chicken:100K	1	taeniopygia_guttata	65,336,665	65,345,112	Unique
chicken:100K	1	meleagris_gallopavo	66,340,921	66,345,507	Unique
chicken:100K	1	meleagris_gallopavo	66,504,822	66,531,391	Unique
chicken:100K	1	taeniopygia_guttata	66,647,351	66,653,559	Unique
chicken:100K	1	passeroidea + corvoidea	66,927,502	66,949,983	Unique
chicken:100K	1	manacus_vitellinus	66,998,101	67,016,676	Unique
chicken:100K	1	chaetura_pelagica	67,141,660	67,161,128	Unique
chicken:100K	1	geospiza_fortis	67,310,487	67,312,400	Unique
chicken:100K	1	taeniopygia_guttata	67,313,916	67,314,001	Unique
chicken:100K	1	picoides_pubescens	67,327,395	67,332,193	Unique
chicken:100K	1	meleagris_gallopavo	67,388,926	67,395,506	Unique
chicken:100K	1	meleagris_gallopavo	67,499,851	67,502,648	Unique
chicken:100K	1	meleagris_gallopavo	67,738,563	67,744,455	Unique
chicken:100K	1	picoides_pubescens	68,145,384	68,149,673	Unique
chicken:100K	1	passeroidea + corvoidea	68,318,267	68,322,940	Unique
chicken:100K	1	chaetura_pelagica	68,442,053	68,445,290	Unique
chicken:100K	1	meleagris_gallopavo	68,600,040	68,658,029	Unique
chicken:100K	1	melopsittacus_undulatus:chaetura_pelagica	69,211,222	69,212,497	Reuse
chicken:100K	1	chaetura_pelagica:melopsittacus_undulatus	69,211,222	69,212,497	Reuse
chicken:100K	1	meleagris_gallopavo	69,406,978	69,412,807	Unique
chicken:100K	1	corvus_brachyrhynchos:chaetura_pelagica	69,803,287	69,814,376	Reuse
chicken:100K	1	chaetura_pelagica:corvus_brachyrhynchos	69,803,287	69,814,376	Reuse
chicken:100K	1	meleagris_gallopavo	69,951,079	69,953,442	Unique
chicken:100K	1	melopsittacus_undulatus	70,069,637	70,078,216	Unique
chicken:100K	1	ophisthocomus_hoazin	70,586,371	70,589,485	Unique
chicken:100K	1	anolis_carolinensis	70,705,010	70,730,451	Unique
chicken:100K	1	aptenodytes_forsteri	71,255,588	71,258,256	Unique
chicken:100K	1	picoides_pubescens	71,526,640	71,531,025	Unique
chicken:100K	1	taeniopygia_guttata	71,782,945	71,783,449	Unique
chicken:100K	1	chrysemys_picta	71,861,324	71,872,803	Unique
chicken:100K	1	chaetura_pelagica	71,979,442	71,984,121	Unique

chicken:100K	1	chicken	72,413,370	72,414,784	Unique
chicken:100K	1	meleagris_gallopavo	72,578,552	72,581,041	Unique
chicken:100K	1	meleagris_gallopavo	72,792,993	72,801,495	Unique
chicken:100K	1	galliformes	73,165,012	73,168,005	Unique
chicken:100K	1	passeroidea + corvoidea	73,509,077	73,521,425	Unique
chicken:100K	1	meleagris_gallopavo	73,847,983	73,983,136	Unique
chicken:100K	1	galliformes	74,630,982	74,632,558	Unique
chicken:100K	1	anolis_carolinensis	74,991,515	75,065,524	Unique
chicken:100K	1	columba_livia	75,209,128	75,211,537	Unique
chicken:100K	1	chinese_alligator	75,378,563	75,389,757	Unique
chicken:100K	1	chaetura_pelagica	75,523,810	75,532,686	Unique
chicken:100K	1	struthio_camelus	75,669,978	75,700,383	Unique
chicken:100K	1	galliformes	75,869,273	75,870,953	Unique
chicken:100K	1	melopsittacus_undulatus	76,251,962	76,255,885	Unique
chicken:100K	1	nipponia_nippon	76,337,047	76,341,241	Unique
chicken:100K	1	chaetura_pelagica	76,364,861	76,373,558	Unique
chicken:100K	1	calypte_anna	76,390,001	76,395,313	Unique
chicken:100K	1	nipponia_nippon	76,731,313	76,731,781	Unique
chicken:100K	1	ophisthocomus_hoazin	77,424,293	77,452,958	Unique
chicken:100K	1	galliformes	77,935,542	77,950,129	Unique
chicken:100K	1	struthio_camelus	78,464,720	78,465,944	Unique
chicken:100K	1	birds_crocs_turtles	78,598,220	78,617,687	Unique
chicken:100K	1	columba_livia	79,606,378	79,608,088	Unique
chicken:100K	1	nipponia_nippon	80,302,797	80,304,615	Unique
chicken:100K	1	aptenodytes_forsteri	82,024,764	82,026,502	Unique
chicken:100K	1	egretta_garzetta	82,310,897	82,312,599	Unique
chicken:100K	1	opossum:melopsittacus_undulatus	82,457,408	82,628,930	Reuse
chicken:100K	1	melopsittacus_undulatus:opossum	82,457,408	82,628,930	Reuse
chicken:100K	1	egretta_garzetta	82,644,612	82,645,387	Unique
chicken:100K	1	taeniopygia_guttata	83,916,726	83,917,179	Unique
chicken:100K	1	pygoscelis_adeliae	84,096,533	84,098,016	Unique
chicken:100K	1	melopsittacus_undulatus	85,145,349	85,171,567	Unique
chicken:100K	1	melopsittacus_undulatus	85,340,731	85,342,392	Unique
chicken:100K	1	meleagris_gallopavo	85,592,016	85,627,964	Unique
chicken:100K	1	melopsittacus_undulatus	86,337,199	86,343,158	Unique
chicken:100K	1	opossum:melopsittacus_undulatus	86,742,609	86,812,735	Reuse
chicken:100K	1	melopsittacus_undulatus:opossum	86,742,609	86,812,735	Reuse
chicken:100K	1	melopsittacus_undulatus:columba_livia	86,979,579	86,980,488	Reuse
chicken:100K	1	columba_livia:melopsittacus_undulatus	86,979,579	86,980,488	Reuse
chicken:100K	1	chaetura_pelagica	87,942,689	87,944,890	Unique
chicken:100K	1	manacus_vitellinus	88,092,859	88,101,278	Unique
chicken:100K	1	passeroidea + corvoidea	88,139,067	88,147,556	Unique
chicken:100K	1	melopsittacus_undulatus	88,792,289	88,834,841	Unique
chicken:100K	1	melopsittacus_undulatus	89,719,217	89,732,493	Unique
chicken:100K	1	calypte_anna	90,906,153	90,911,569	Unique
chicken:100K	1	columba_livia	91,170,101	91,175,236	Unique
chicken:100K	1	manacus_vitellinus	92,418,689	92,422,087	Unique
chicken:100K	1	meleagris_gallopavo	92,718,577	92,736,825	Unique
chicken:100K	1	meleagris_gallopavo	93,116,435	93,118,032	Unique
chicken:100K	1	meleagris_gallopavo	93,253,574	93,256,840	Unique
chicken:100K	1	melopsittacus_undulatus	93,390,936	93,401,182	Unique
chicken:100K	1	struthio_camelus	93,635,353	93,644,203	Unique
chicken:100K	1	pygoscelis_adeliae	94,168,594	94,171,061	Unique
chicken:100K	1	meleagris_gallopavo:chrysemys_picta	94,802,543	94,816,836	Reuse
chicken:100K	1	chrysemys_picta:meleagris_gallopavo	94,802,543	94,816,836	Reuse
chicken:100K	1	melopsittacus_undulatus:manacus_vitellinus	94,863,188	94,866,901	Reuse
chicken:100K	1	manacus_vitellinus:melopsittacus_undulatus	94,863,188	94,866,901	Reuse
chicken:100K	1	struthio_camelus	95,024,959	95,025,186	Unique
chicken:100K	1	chrysemys_picta	95,493,711	95,495,054	Unique
chicken:100K	1	anolis_carolinensis	96,165,257	96,173,877	Unique
chicken:100K	1	melopsittacus_undulatus	97,207,251	97,210,939	Unique
chicken:100K	1	melopsittacus_undulatus	97,356,773	97,365,487	Unique
chicken:100K	1	anolis_carolinensis	97,826,401	97,831,430	Unique
chicken:100K	1	columba_livia	98,957,586	98,963,992	Unique
chicken:100K	1	corvus_brachyrhynchos	99,006,517	99,009,063	Unique
chicken:100K	1	chaetura_pelagica	99,059,598	99,068,148	Unique

chicken:100K	1	meleagris_gallopavo	99,640,339	99,640,651	Unique
chicken:100K	1	meleagris_gallopavo	99,891,664	99,896,114	Unique
chicken:100K	1	falco_peregrinus	100,037,604	100,113,306	Unique
chicken:100K	1	meleagris_gallopavo	100,448,099	100,541,967	Unique
chicken:100K	1	meleagris_gallopavo	100,658,381	100,658,586	Unique
chicken:100K	1	corvus_brachyrhynchos	100,856,867	100,860,287	Unique
chicken:100K	1	chrysemys_picta	101,817,876	101,835,358	Unique
chicken:100K	1	chrysemys_picta	102,001,814	102,030,917	Unique
chicken:100K	1	falco_peregrinus	102,080,440	102,130,311	Unique
chicken:100K	1	chrysemys_picta	102,416,298	102,426,636	Unique
chicken:100K	1	chrysemys_picta	103,204,782	103,229,251	Unique
chicken:100K	1	passeroidea + corvoidea	103,279,914	103,284,400	Unique
chicken:100K	1	passeroidea + corvoidea	103,453,722	103,455,135	Unique
chicken:100K	1	meleagris_gallopavo	103,734,890	103,740,002	Unique
chicken:100K	1	pygoscelis_adeliae	103,786,999	103,788,821	Unique
chicken:100K	1	corvus_brachyrhynchos	104,029,738	104,041,632	Unique
chicken:100K	1	falco_peregrinus	104,196,110	104,199,261	Unique
chicken:100K	1	anolis_carolinensis	104,525,322	104,528,565	Unique
chicken:100K	1	taeniopygia_guttata:chaetura_pelagica	105,282,458	105,318,568	Reuse
chicken:100K	1	chaetura_pelagica:taeniopygia_guttata	105,282,458	105,318,568	Reuse
chicken:100K	1	geospiza_fortis:corvus_brachyrhynchos	105,370,435	105,384,938	Reuse
chicken:100K	1	corvus_brachyrhynchos:geospiza_fortis	105,370,435	105,384,938	Reuse
chicken:100K	1	meleagris_gallopavo	106,138,711	106,150,991	Unique
chicken:100K	1	struthio_camelus:chrysemys_picta	106,540,239	106,548,269	Reuse
chicken:100K	1	chrysemys_picta:struthio_camelus	106,540,239	106,548,269	Reuse
chicken:100K	1	columba_livia	107,975,084	107,977,673	Unique
chicken:100K	1	opossum:chrysemys_picta	109,663,273	109,755,472	Reuse
chicken:100K	1	chrysemys_picta:opossum	109,663,273	109,755,472	Reuse
chicken:100K	1	corvus_brachyrhynchos	109,808,314	109,809,271	Unique
chicken:100K	1	meleagris_gallopavo	111,692,534	111,706,804	Unique
chicken:100K	1	meleagris_gallopavo:anas_platyrhynchos	111,885,484	111,887,345	Reuse
chicken:100K	1	anas_platyrhynchos:meleagris_gallopavo	111,885,484	111,887,345	Reuse
chicken:100K	1	melopsittacus_undulatus	111,939,623	111,942,334	Unique
chicken:100K	1	anas_platyrhynchos	112,706,301	112,733,100	Unique
chicken:100K	1	columba_livia	112,920,855	112,922,899	Unique
chicken:100K	1	anolis_carolinensis	113,298,640	113,743,498	Unique
chicken:100K	1	chrysemys_picta	114,514,702	114,521,911	Unique
chicken:100K	1	columba_livia	115,147,007	115,151,802	Unique
chicken:100K	1	corvus_brachyrhynchos	115,159,850	115,165,845	Unique
chicken:100K	1	melopsittacus_undulatus	115,217,491	115,220,744	Unique
chicken:100K	1	columba_livia	115,308,753	115,312,173	Unique
chicken:100K	1	nipponia_nippon:chrysemys_picta	117,163,254	117,164,167	Reuse
chicken:100K	1	chrysemys_picta:nipponia_nippon	117,163,254	117,164,167	Reuse
chicken:100K	1	chrysemys_picta	117,606,594	117,610,371	Unique
chicken:100K	1	anas_platyrhynchos	117,818,423	117,821,824	Unique
chicken:100K	1	struthio_camelus	119,043,798	119,046,822	Unique
chicken:100K	1	struthio_camelus	119,190,830	119,193,413	Unique
chicken:100K	1	chrysemys_picta:anas_platyrhynchos	119,250,727	119,256,036	Reuse
chicken:100K	1	anas_platyrhynchos:chrysemys_picta	119,250,727	119,256,036	Reuse
chicken:100K	1	chrysemys_picta	120,147,406	120,157,358	Unique
chicken:100K	1	meleagris_gallopavo	120,278,042	120,309,961	Unique
chicken:100K	1	chrysemys_picta	120,521,278	120,801,657	Unique
chicken:100K	1	meleagris_gallopavo	121,097,355	121,101,994	Unique
chicken:100K	1	picoides_pubescens	121,246,007	121,496,985	Unique
chicken:100K	1	meleagris_gallopavo	121,766,898	121,769,973	Unique
chicken:100K	1	picoides_pubescens:meleagris_gallopavo	121,938,636	122,095,363	Reuse
chicken:100K	1	meleagris_gallopavo:picoides_pubescens	121,938,636	122,095,363	Reuse
chicken:100K	1	chinese_alligator	122,945,588	123,548,477	Unique
chicken:100K	1	egretta_garzetta	124,850,187	124,851,217	Unique
chicken:100K	1	chrysemys_picta	125,451,718	125,468,894	Unique
chicken:100K	1	meleagris_gallopavo	125,783,117	125,787,300	Unique
chicken:100K	1	meleagris_gallopavo	126,359,979	126,394,041	Unique
chicken:100K	1	meleagris_gallopavo	126,659,733	126,672,746	Unique
chicken:100K	1	meleagris_gallopavo	126,872,401	126,952,924	Unique
chicken:100K	1	geospiza_fortis:chaetura_pelagica	127,174,897	127,198,979	Reuse
chicken:100K	1	chaetura_pelagica:geospiza_fortis	127,174,897	127,198,979	Reuse

chicken:100K	1	geospiza_fortis	127,343,515	127,353,709	Unique
chicken:100K	1	chrysemys_picta	127,570,251	127,574,959	Unique
chicken:100K	1	passeroidea + corvoidea	127,801,451	127,804,749	Unique
chicken:100K	1	falco_peregrinus	127,861,470	127,863,234	Unique
chicken:100K	1	chrysemys_picta	128,084,524	128,113,700	Unique
chicken:100K	1	chinese_alligator	128,806,952	128,830,522	Unique
chicken:100K	1	chinese_alligator	129,630,958	129,850,112	Unique
chicken:100K	1	chrysemys_picta	131,024,742	131,039,327	Unique
chicken:100K	1	aptenodytes_forsteri	131,109,300	131,111,243	Unique
chicken:100K	1	picoides_pubescens	131,338,446	131,344,399	Unique
chicken:100K	1	columba_livia:anolis_carolinensis:chaetura_pelagica	131,790,976	131,884,438	Reuse
chicken:100K	1	chaetura_pelagica:columba_livia:anolis_carolinensis	131,790,976	131,884,438	Reuse
chicken:100K	1	anolis_carolinensis:chaetura_pelagica:columba_livia	131,790,976	131,884,438	Reuse
chicken:100K	1	meleagris_gallopavo	131,908,115	131,911,152	Unique
chicken:100K	1	meleagris_gallopavo	132,501,353	132,504,105	Unique
chicken:100K	1	chrysemys_picta	132,770,391	132,820,347	Unique
chicken:100K	1	anolis_carolinensis	134,421,246	134,451,426	Unique
chicken:100K	1	chrysemys_picta	136,591,631	136,601,732	Unique
chicken:100K	1	galloanserae	137,313,202	137,315,743	Unique
chicken:100K	1	meleagris_gallopavo	137,507,947	137,513,954	Unique
chicken:100K	1	cuculus_canorus	138,424,473	138,434,054	Unique
chicken:100K	1	melopsittacus_undulatus	138,566,658	138,572,517	Unique
chicken:100K	1	meleagris_gallopavo	139,032,177	139,066,771	Unique
chicken:100K	1	galloanserae	139,331,778	139,333,713	Unique
chicken:100K	1	meleagris_gallopavo	139,842,686	139,846,487	Unique
chicken:100K	1	meleagris_gallopavo	140,008,655	140,009,632	Unique
chicken:100K	1	meleagris_gallopavo	140,509,115	140,662,653	Unique
chicken:100K	1	chrysemys_picta	141,157,806	141,164,111	Unique
chicken:100K	1	meleagris_gallopavo	141,184,444	141,228,056	Unique
chicken:100K	1	meleagris_gallopavo	141,330,913	141,339,837	Unique
chicken:100K	1	egretta_garzetta	143,295,040	143,297,229	Unique
chicken:100K	1	meleagris_gallopavo	143,824,297	143,870,527	Unique
chicken:100K	1	meleagris_gallopavo	144,008,045	144,010,420	Unique
chicken:100K	1	chrysemys_picta	144,097,493	144,099,792	Unique
chicken:100K	1	meleagris_gallopavo	144,138,823	144,142,169	Unique
chicken:100K	1	anolis_carolinensis	144,398,996	144,430,816	Unique
chicken:100K	1	anolis_carolinensis	146,621,774	146,939,079	Unique
chicken:100K	1	chinese_alligator	147,068,497	147,077,576	Unique
chicken:100K	1	pygoscelis_adeliae	147,639,784	147,643,788	Unique
chicken:100K	1	cuculus_canorus	147,891,866	147,903,737	Unique
chicken:100K	1	cuculus_canorus	148,117,485	148,120,199	Unique
chicken:100K	1	ophisthocomus_hoazin	148,506,655	148,508,706	Unique
chicken:100K	1	meleagris_gallopavo	148,702,788	148,706,738	Unique
chicken:100K	1	spheniscidae	148,827,032	148,838,834	Unique
chicken:100K	1	meleagris_gallopavo	148,896,283	148,903,607	Unique
chicken:100K	1	meleagris_gallopavo:manacus_vitellinus	149,786,086	149,793,206	Reuse
chicken:100K	1	manacus_vitellinus:meleagris_gallopavo	149,786,086	149,793,206	Reuse
chicken:100K	1	meleagris_gallopavo	149,964,413	149,974,872	Unique
chicken:100K	1	chinese_alligator	150,316,542	150,377,272	Unique
chicken:100K	1	ophisthocomus_hoazin:meleagris_gallopavo:anas_platyrhynchos	150,664,299	150,938,871	Reuse
chicken:100K	1	meleagris_gallopavo:anas_platyrhynchos:ophisthocomus_hoazin	150,664,299	150,938,871	Reuse
chicken:100K	1	anas_platyrhynchos:ophisthocomus_hoazin:meleagris_gallopavo	150,664,299	150,938,871	Reuse
chicken:100K	1	pygoscelis_adeliae	151,079,499	151,083,119	Unique
chicken:100K	1	meleagris_gallopavo	151,211,750	151,360,628	Unique
chicken:100K	1	ophisthocomus_hoazin	153,015,186	153,019,601	Unique
chicken:100K	1	cuculus_canorus	153,036,353	153,059,491	Unique
chicken:100K	1	anolis_carolinensis	153,179,137	153,181,703	Unique
chicken:100K	1	meleagris_gallopavo	153,282,439	153,307,991	Unique
chicken:100K	1	meleagris_gallopavo	153,455,226	153,457,000	Unique
chicken:100K	1	meleagris_gallopavo	153,649,315	153,653,256	Unique
chicken:100K	1	ophisthocomus_hoazin	154,605,462	154,606,823	Unique
chicken:100K	1	meleagris_gallopavo	155,408,010	155,427,592	Unique
chicken:100K	1	meleagris_gallopavo:anolis_carolinensis	155,543,145	155,598,060	Reuse
chicken:100K	1	anolis_carolinensis:meleagris_gallopavo	155,543,145	155,598,060	Reuse
chicken:100K	1	manacus_vitellinus	157,125,833	157,171,552	Unique
chicken:100K	1	aptenodytes_forsteri	157,928,941	157,936,327	Unique

chicken:100K	1	chrysemys_picta	158,035,267	158,049,200	Unique
chicken:100K	1	meleagris_gallopavo	159,009,013	159,012,865	Unique
chicken:100K	1	melopsittacus_undulatus	159,620,982	159,680,092	Unique
chicken:100K	1	meleagris_gallopavo	160,315,229	160,401,516	Unique
chicken:100K	1	meleagris_gallopavo	160,564,731	160,570,570	Unique
chicken:100K	1	meleagris_gallopavo	160,806,383	160,810,508	Unique
chicken:100K	1	columba_livia:chrysemys_picta	161,025,358	161,426,374	Reuse
chicken:100K	1	chrysemys_picta:columba_livia	161,025,358	161,426,374	Reuse
chicken:100K	1	egretta_garzetta	161,803,049	161,805,497	Unique
chicken:100K	1	meleagris_gallopavo:chinese_alligator	163,198,089	163,753,859	Reuse
chicken:100K	1	chinese_alligator:meleagris_gallopavo	163,198,089	163,753,859	Reuse
chicken:100K	1	meleagris_gallopavo	164,356,702	164,485,533	Unique
chicken:100K	1	aptenodytes_forsteri	167,067,659	167,069,432	Unique
chicken:100K	1	anolis_carolinensis	167,712,802	167,845,368	Unique
chicken:100K	1	aptenodytes_forsteri	168,105,793	168,109,658	Unique
chicken:100K	1	anolis_carolinensis	168,173,308	168,447,283	Unique
chicken:100K	1	egretta_garzetta:aptenodytes_forsteri	169,048,476	169,052,939	Reuse
chicken:100K	1	aptenodytes_forsteri:egretta_garzetta	169,048,476	169,052,939	Reuse
chicken:100K	1	meleagris_gallopavo	169,133,510	169,135,697	Unique
chicken:100K	1	meleagris_gallopavo	170,367,879	170,372,048	Unique
chicken:100K	1	meleagris_gallopavo	170,726,415	170,727,584	Unique
chicken:100K	1	picoides_pubescens	170,800,863	170,805,414	Unique
chicken:100K	1	manacus_vitellinus:anolis_carolinensis	171,370,264	171,611,382	Reuse
chicken:100K	1	anolis_carolinensis:manacus_vitellinus	171,370,264	171,611,382	Reuse
chicken:100K	1	meleagris_gallopavo	172,559,335	172,589,547	Unique
chicken:100K	1	meleagris_gallopavo	172,835,231	172,838,864	Unique
chicken:100K	1	chinese_alligator	174,964,634	175,095,783	Unique
chicken:100K	1	anolis_carolinensis	175,314,049	175,317,750	Unique
chicken:100K	1	anolis_carolinensis	176,431,415	176,586,809	Unique
chicken:100K	1	cuculus_canorus	177,114,830	177,115,846	Unique
chicken:100K	1	anas_platyrhynchos	177,969,321	177,970,708	Unique
chicken:100K	1	chrysemys_picta:anas_platyrhynchos	178,166,498	178,168,570	Reuse
chicken:100K	1	anas_platyrhynchos:chrysemys_picta	178,166,498	178,168,570	Reuse
chicken:100K	1	pygoscelis_adeliae	178,357,941	178,360,818	Unique
chicken:100K	1	struthio_camelus	178,401,152	178,404,387	Unique
chicken:100K	1	opossum:aptenodytes_forsteri	178,802,043	178,804,700	Reuse
chicken:100K	1	aptenodytes_forsteri:opossum	178,802,043	178,804,700	Reuse
chicken:100K	1	taeniopygia_guttata	178,954,904	178,957,416	Unique
chicken:100K	1	anas_platyrhynchos	178,996,820	178,999,435	Unique
chicken:100K	1	aptenodytes_forsteri:anolis_carolinensis	179,397,623	179,400,319	Reuse
chicken:100K	1	anolis_carolinensis:aptenodytes_forsteri	179,397,623	179,400,319	Reuse
chicken:100K	1	chrysemys_picta	181,365,602	181,405,805	Unique
chicken:100K	1	cuculus_canorus	182,035,358	182,047,794	Unique
chicken:100K	1	aptenodytes_forsteri	182,496,529	182,498,353	Unique
chicken:100K	1	struthio_camelus	184,894,333	184,914,526	Unique
chicken:100K	1	falco_peregrinus:boa_constrictor	185,061,724	185,631,215	Reuse
chicken:100K	1	boa_constrictor:falco_peregrinus	185,061,724	185,631,215	Reuse
chicken:100K	1	falco_peregrinus	186,379,419	186,478,734	Unique
chicken:100K	1	cuculus_canorus	187,950,216	187,955,312	Unique
chicken:100K	1	meleagris_gallopavo	189,216,863	189,220,443	Unique
chicken:100K	1	meleagris_gallopavo	189,342,431	189,346,173	Unique
chicken:100K	1	picoides_pubescens:cuculus_canorus	189,472,481	189,477,198	Reuse
chicken:100K	1	cuculus_canorus:picoides_pubescens	189,472,481	189,477,198	Reuse
chicken:100K	1	cuculus_canorus	189,685,072	189,685,075	Unique
chicken:100K	1	melopsittacus_undulatus	189,928,555	189,931,148	Unique
chicken:100K	1	melopsittacus_undulatus	191,772,698	191,810,248	Unique
chicken:100K	1	corvus_brachyrhynchos	192,771,002	192,776,115	Unique
chicken:100K	1	passeroidea	192,816,997	192,823,275	Unique
chicken:100K	1	falco_peregrinus	194,070,521	194,076,222	Unique
chicken:100K	1	struthio_camelus	194,499,219	194,511,990	Unique
chicken:100K	1	struthio_camelus	194,875,397	194,879,028	Unique
chicken:100K	1	opossum:chrysemys_picta	194,925,801	194,966,782	Reuse
chicken:100K	1	chrysemys_picta:opossum	194,925,801	194,966,782	Reuse
chicken:100K	1	manacus_vitellinus	195,078,425	195,082,449	Unique
chicken:100K	2	struthio_camelus	546,437	552,084	Unique
chicken:100K	2	boa_constrictor	701,112	710,099	Unique

chicken:100K	2	picoides_pubescens	747,576	776,766	Unique
chicken:100K	2	non_galloanserae	931,613	971,175	Unique
chicken:100K	2	anas_platyrhynchos	1,311,923	1,313,433	Unique
chicken:100K	2	struthio_camelus	1,313,616	1,318,624	Unique
chicken:100K	2	struthio_camelus	1,474,219	1,476,280	Unique
chicken:100K	2	corvus_brachyrhynchos	3,303,403	3,339,754	Unique
chicken:100K	2	corvus_brachyrhynchos	3,771,320	3,781,270	Unique
chicken:100K	2	galliformes	4,047,084	4,051,112	Unique
chicken:100K	2	anas_platyrhynchos	4,342,846	4,343,779	Unique
chicken:100K	2	aptenodytes_forsteri	5,324,469	5,328,194	Unique
chicken:100K	2	anolis_carolinensis	5,578,470	5,713,301	Unique
chicken:100K	2	melopsittacus_undulatus	7,833,126	7,839,039	Unique
chicken:100K	2	falco_peregrinus	7,985,176	8,014,362	Unique
chicken:100K	2	picoides_pubescens	9,571,681	9,606,591	Unique
chicken:100K	2	picoides_pubescens	10,233,846	10,245,986	Unique
chicken:100K	2	chrysemys_picta	10,915,967	10,926,467	Unique
chicken:100K	2	struthio_camelus	13,749,906	13,750,122	Unique
chicken:100K	2	aptenodytes_forsteri	14,004,515	14,021,020	Unique
chicken:100K	2	struthio_camelus	14,047,155	14,048,043	Unique
chicken:100K	2	picoides_pubescens	14,896,790	14,917,682	Unique
chicken:100K	2	pygoscelis_adeliae	15,097,979	15,100,449	Unique
chicken:100K	2	nipponia_nippon	15,282,537	15,286,480	Unique
chicken:100K	2	picoides_pubescens	16,337,599	16,356,696	Unique
chicken:100K	2	picoides_pubescens	16,808,918	16,816,583	Unique
chicken:100K	2	meleagris_gallopavo	18,178,070	18,180,136	Unique
chicken:100K	2	meleagris_gallopavo:falco_peregrinus	18,297,468	18,300,410	Reuse
chicken:100K	2	falco_peregrinus:meleagris_gallopavo	18,297,468	18,300,410	Reuse
chicken:100K	2	chrysemys_picta	19,573,392	19,589,903	Unique
chicken:100K	2	chrysemys_picta	19,958,817	19,974,224	Unique
chicken:100K	2	picoides_pubescens	20,197,364	20,199,078	Unique
chicken:100K	2	chrysemys_picta	20,511,139	20,532,421	Unique
chicken:100K	2	chrysemys_picta	20,703,659	20,717,484	Unique
chicken:100K	2	chrysemys_picta	21,420,367	21,455,567	Unique
chicken:100K	2	falco_peregrinus	22,035,433	22,155,209	Unique
chicken:100K	2	chrysemys_picta	22,263,207	22,285,644	Unique
chicken:100K	2	picoides_pubescens	22,503,208	22,525,357	Unique
chicken:100K	2	picoides_pubescens	22,626,371	22,640,197	Unique
chicken:100K	2	chrysemys_picta	22,741,879	22,763,386	Unique
chicken:100K	2	meleagris_gallopavo:aptenodytes_forsteri	23,179,268	23,180,895	Reuse
chicken:100K	2	aptenodytes_forsteri:meleagris_gallopavo	23,179,268	23,180,895	Reuse
chicken:100K	2	chinese_alligator	24,303,538	24,371,574	Unique
chicken:100K	2	falco_peregrinus:chinese_alligator	24,647,861	24,857,238	Reuse
chicken:100K	2	chinese_alligator:falco_peregrinus	24,647,861	24,857,238	Reuse
chicken:100K	2	falco_peregrinus	25,653,762	25,736,273	Unique
chicken:100K	2	taeniopygia_guttata	26,116,750	26,118,526	Unique
chicken:100K	2	aptenodytes_forsteri	26,451,737	26,455,906	Unique
chicken:100K	2	chrysemys_picta	26,847,221	26,865,625	Unique
chicken:100K	2	chrysemys_picta	27,442,276	27,450,273	Unique
chicken:100K	2	chinese_alligator	28,476,729	28,558,014	Unique
chicken:100K	2	chrysemys_picta:chinese_alligator	29,249,212	29,287,617	Reuse
chicken:100K	2	chinese_alligator:chrysemys_picta	29,249,212	29,287,617	Reuse
chicken:100K	2	meleagris_gallopavo	29,699,195	29,701,454	Unique
chicken:100K	2	meleagris_gallopavo	29,840,816	29,845,706	Unique
chicken:100K	2	meleagris_gallopavo	29,961,051	29,981,907	Unique
chicken:100K	2	meleagris_gallopavo	30,176,002	30,184,486	Unique
chicken:100K	2	meleagris_gallopavo	30,326,765	30,332,573	Unique
chicken:100K	2	meleagris_gallopavo	30,951,854	30,997,509	Unique
chicken:100K	2	melopsittacus_undulatus	31,014,934	31,023,417	Unique
chicken:100K	2	chinese_alligator	32,269,016	32,316,488	Unique
chicken:100K	2	chinese_alligator:anolis_carolinensis	32,494,079	32,719,313	Reuse
chicken:100K	2	anolis_carolinensis:chinese_alligator	32,494,079	32,719,313	Reuse
chicken:100K	2	ophisthocomus_hoazin:chinese_alligator	33,361,796	33,616,476	Reuse
chicken:100K	2	chinese_alligator:ophisthocomus_hoazin	33,361,796	33,616,476	Reuse
chicken:100K	2	anolis_carolinensis	34,287,241	34,369,134	Unique
chicken:100K	2	chinese_alligator	35,119,041	35,120,158	Unique
chicken:100K	2	meleagris_gallopavo	36,068,634	36,070,984	Unique

chicken:100K	2	meleagris_gallopavo	36,184,586	36,195,276	Unique
chicken:100K	2	meleagris_gallopavo	36,458,966	36,462,064	Unique
chicken:100K	2	melopsittacus_undulatus	36,652,868	36,658,497	Unique
chicken:100K	2	anolis_carolinensis	36,924,075	36,942,352	Unique
chicken:100K	2	columba_livia	37,400,122	37,403,989	Unique
chicken:100K	2	meleagris_gallopavo	38,555,121	38,555,876	Unique
chicken:100K	2	meleagris_gallopavo:chinese_alligator	38,802,415	38,887,003	Reuse
chicken:100K	2	chinese_alligator:meleagris_gallopavo	38,802,415	38,887,003	Reuse
chicken:100K	2	cuculus_canorus	38,980,849	38,982,141	Unique
chicken:100K	2	meleagris_gallopavo	38,992,841	39,019,780	Unique
chicken:100K	2	meleagris_gallopavo	39,276,276	39,414,735	Unique
chicken:100K	2	chinese_alligator	40,480,537	40,480,719	Unique
chicken:100K	2	chinese_alligator	41,107,005	41,142,228	Unique
chicken:100K	2	melopsittacus_undulatus:anolis_carolinensis	41,675,716	41,678,790	Reuse
chicken:100K	2	anolis_carolinensis:melopsittacus_undulatus	41,675,716	41,678,790	Reuse
chicken:100K	2	picoides_pubescens:melopsittacus_undulatus	41,792,490	41,795,104	Reuse
chicken:100K	2	melopsittacus_undulatus:picoides_pubescens	41,792,490	41,795,104	Reuse
chicken:100K	2	melopsittacus_undulatus	41,910,217	41,913,576	Unique
chicken:100K	2	calypte_anna	42,025,610	42,026,328	Unique
chicken:100K	2	cuculus_canorus	42,179,882	42,182,768	Unique
chicken:100K	2	picoides_pubescens	42,201,703	42,214,258	Unique
chicken:100K	2	charadrius_vociferus	42,749,690	42,759,851	Unique
chicken:100K	2	melopsittacus_undulatus	42,920,424	42,952,033	Unique
chicken:100K	2	melopsittacus_undulatus	43,553,597	43,638,388	Unique
chicken:100K	2	boa_constrictor	43,658,775	43,708,358	Unique
chicken:100K	2	ophisthocomus_hoazin	43,888,449	43,893,406	Unique
chicken:100K	2	pygoscelis_adeliae	44,016,868	44,019,713	Unique
chicken:100K	2	anas_platyrhynchos	44,412,270	44,413,811	Unique
chicken:100K	2	anas_platyrhynchos	44,551,569	44,551,699	Unique
chicken:100K	2	anas_platyrhynchos	44,867,790	44,869,794	Unique
chicken:100K	2	anas_platyrhynchos	44,982,243	44,984,828	Unique
chicken:100K	2	anas_platyrhynchos	45,388,302	45,395,092	Unique
chicken:100K	2	anolis_carolinensis	45,515,455	45,603,600	Unique
chicken:100K	2	taeniopygia_guttata	46,454,805	46,458,276	Unique
chicken:100K	2	anas_platyrhynchos	46,603,514	46,604,008	Unique
chicken:100K	2	neognathae	46,618,768	46,622,427	Unique
chicken:100K	2	geospiza_fortis	46,731,249	46,737,496	Unique
chicken:100K	2	boa_constrictor	46,839,186	46,974,595	Unique
chicken:100K	2	chrysemys_picta	47,565,140	47,577,214	Unique
chicken:100K	2	anas_platyrhynchos	48,465,472	48,467,389	Unique
chicken:100K	2	cuculus_canorus	48,695,859	48,697,674	Unique
chicken:100K	2	passeroidea	49,116,974	49,127,358	Unique
chicken:100K	2	cuculus_canorus	49,539,261	49,539,440	Unique
chicken:100K	2	neognathae	49,698,798	49,723,557	Unique
chicken:100K	2	nipponia_nippon	50,323,769	50,326,808	Unique
chicken:100K	2	columba_livia	50,470,981	50,473,358	Unique
chicken:100K	2	chrysemys_picta	51,643,783	51,658,799	Unique
chicken:100K	2	chaetura_pelagica	52,387,606	52,390,295	Unique
chicken:100K	2	meleagris_gallopavo	52,718,147	52,750,067	Unique
chicken:100K	2	egretta_garzetta	52,917,440	52,923,878	Unique
chicken:100K	2	picoides_pubescens	53,056,663	53,111,087	Unique
chicken:100K	2	columba_livia	53,183,252	53,188,285	Unique
chicken:100K	2	meleagris_gallopavo	53,638,964	53,639,489	Unique
chicken:100K	2	meleagris_gallopavo	53,867,960	53,874,284	Unique
chicken:100K	2	picoides_pubescens	54,299,648	54,303,259	Unique
chicken:100K	2	taeniopygia_guttata	55,095,224	55,098,773	Unique
chicken:100K	2	columba_livia	55,420,152	55,420,379	Unique
chicken:100K	2	meleagris_gallopavo	55,529,343	55,538,628	Unique
chicken:100K	2	non_galloanserae	55,626,666	55,637,198	Unique
chicken:100K	2	anas_platyrhynchos	55,764,133	55,768,204	Unique
chicken:100K	2	struthio_camelus:columba_livia	56,143,114	56,146,951	Reuse
chicken:100K	2	columba_livia:struthio_camelus	56,143,114	56,146,951	Reuse
chicken:100K	2	chinese_alligator	57,399,218	57,411,661	Unique
chicken:100K	2	neognathae	57,784,333	58,153,457	Unique
chicken:100K	2	meleagris_gallopavo	59,207,282	59,209,076	Unique
chicken:100K	2	calypte_anna	59,709,285	59,712,321	Unique

chicken:100K	2	meleagris_gallopavo:chrysemys_picta	59,850,771	59,852,075	Reuse
chicken:100K	2	chrysemys_picta:meleagris_gallopavo	59,850,771	59,852,075	Reuse
chicken:100K	2	ophisthocomus_hoazin	60,296,742	60,300,829	Unique
chicken:100K	2	chrysemys_picta	60,691,648	60,712,604	Unique
chicken:100K	2	meleagris_gallopavo	61,123,409	61,123,994	Unique
chicken:100K	2	meleagris_gallopavo	61,246,358	61,256,004	Unique
chicken:100K	2	chrysemys_picta	61,840,209	61,849,122	Unique
chicken:100K	2	anolis_carolinensis	62,660,002	62,661,514	Unique
chicken:100K	2	anas_platyrhynchos	63,042,531	63,047,547	Unique
chicken:100K	2	taeniopygia_guttata	64,083,924	64,085,056	Unique
chicken:100K	2	picoides_pubescens	64,425,924	64,427,189	Unique
chicken:100K	2	boa_constrictor	64,715,251	64,732,828	Unique
chicken:100K	2	anas_platyrhynchos	66,011,079	66,012,678	Unique
chicken:100K	2	ophisthocomus_hoazin	66,397,705	66,403,239	Unique
chicken:100K	2	neognathae	67,713,281	67,769,302	Unique
chicken:100K	2	geospiza_fortis	67,926,125	67,927,167	Unique
chicken:100K	2	columba_livia:chrysemys_picta	69,060,390	69,068,417	Reuse
chicken:100K	2	chrysemys_picta:columba_livia	69,060,390	69,068,417	Reuse
chicken:100K	2	ophisthocomus_hoazin	69,470,177	69,474,153	Unique
chicken:100K	2	chrysemys_picta	70,390,043	70,394,995	Unique
chicken:100K	2	falco_peregrinus	70,532,135	70,564,371	Unique
chicken:100K	2	chrysemys_picta	71,356,398	71,367,163	Unique
chicken:100K	2	ophisthocomus_hoazin	71,507,736	71,512,665	Unique
chicken:100K	2	ophisthocomus_hoazin	71,734,305	71,798,145	Unique
chicken:100K	2	chrysemys_picta	71,842,011	71,902,474	Unique
chicken:100K	2	columba_livia	72,385,540	72,386,007	Unique
chicken:100K	2	chrysemys_picta	72,432,050	72,471,990	Unique
chicken:100K	2	meleagris_gallopavo	72,571,783	72,621,766	Unique
chicken:100K	2	taeniopygia_guttata	73,477,811	73,484,371	Unique
chicken:100K	2	chrysemys_picta	73,496,510	73,500,422	Unique
chicken:100K	2	taeniopygia_guttata	74,651,350	74,668,050	Unique
chicken:100K	2	taeniopygia_guttata	74,842,005	74,928,513	Unique
chicken:100K	2	anas_platyrhynchos	76,265,023	76,266,817	Unique
chicken:100K	2	falco_peregrinus:calypte_anna	76,763,556	76,765,646	Reuse
chicken:100K	2	calypte_anna:falco_peregrinus	76,763,556	76,765,646	Reuse
chicken:100K	2	calypte_anna	77,038,873	77,076,986	Unique
chicken:100K	2	neognathae	79,806,445	80,200,414	Unique
chicken:100K	2	struthio_camelus	81,212,704	81,215,768	Unique
chicken:100K	2	chrysemys_picta	81,296,190	81,314,003	Unique
chicken:100K	2	meleagris_gallopavo	82,679,599	82,683,987	Unique
chicken:100K	2	meleagris_gallopavo	82,869,678	82,874,288	Unique
chicken:100K	2	chrysemys_picta	83,282,252	83,286,535	Unique
chicken:100K	2	opossum:chaetura_pelagica	83,489,784	83,606,369	Reuse
chicken:100K	2	chaetura_pelagica:opossum	83,489,784	83,606,369	Reuse
chicken:100K	2	birds	85,698,636	85,709,753	Unique
chicken:100K	2	anolis_carolinensis	86,425,531	86,455,551	Unique
chicken:100K	2	chaetura_pelagica	88,883,527	88,884,062	Unique
chicken:100K	2	chrysemys_picta	89,119,372	89,123,243	Unique
chicken:100K	2	falco_peregrinus	89,704,859	89,709,731	Unique
chicken:100K	2	neognathae	89,782,722	89,784,600	Unique
chicken:100K	2	meleagris_gallopavo	91,182,901	91,191,892	Unique
chicken:100K	2	meleagris_gallopavo	91,393,097	91,398,262	Unique
chicken:100K	2	falco_peregrinus	91,706,282	91,708,332	Unique
chicken:100K	2	meleagris_gallopavo	91,949,483	91,950,854	Unique
chicken:100K	2	picoides_pubescens	91,979,356	91,988,403	Unique
chicken:100K	2	meleagris_gallopavo	92,055,850	92,057,635	Unique
chicken:100K	2	meleagris_gallopavo	93,188,632	93,192,231	Unique
chicken:100K	2	chinese_alligator	93,285,164	93,294,025	Unique
chicken:100K	2	meleagris_gallopavo	93,322,726	93,327,110	Unique
chicken:100K	2	falco_peregrinus	93,814,414	93,816,927	Unique
chicken:100K	2	meleagris_gallopavo	94,160,544	94,162,236	Unique
chicken:100K	2	taeniopygia_guttata	94,197,054	94,203,449	Unique
chicken:100K	2	meleagris_gallopavo	94,561,868	94,565,497	Unique
chicken:100K	2	taeniopygia_guttata	94,827,570	94,834,761	Unique
chicken:100K	2	aptenodytes_forsteri	95,114,680	95,117,372	Unique
chicken:100K	2	pygoscelis_adeliae	96,014,959	96,016,688	Unique

chicken:100K	2	anas_platyrhynchos	96,880,614	96,886,939	Unique
chicken:100K	2	anolis_carolinensis	96,955,191	97,006,722	Unique
chicken:100K	2	struthio_camelus	97,945,564	97,949,845	Unique
chicken:100K	2	pygoscelis_adeliae	98,868,998	98,870,932	Unique
chicken:100K	2	anolis_carolinensis	99,533,379	99,851,865	Unique
chicken:100K	2	pygoscelis_adeliae	100,374,238	100,379,864	Unique
chicken:100K	2	chrysemys_picta	100,616,775	100,626,901	Unique
chicken:100K	2	anolis_carolinensis	100,796,456	100,822,169	Unique
chicken:100K	2	meleagris_gallopavo	101,153,355	101,160,328	Unique
chicken:100K	2	chrysemys_picta	101,281,603	101,307,355	Unique
chicken:100K	2	meleagris_gallopavo	101,403,632	101,408,667	Unique
chicken:100K	2	struthio_camelus	101,786,946	101,791,034	Unique
chicken:100K	2	struthio_camelus	101,935,934	101,939,169	Unique
chicken:100K	2	anas_platyrhynchos	102,943,406	102,945,373	Unique
chicken:100K	2	corvus_brachyrhynchos	105,216,697	105,219,735	Unique
chicken:100K	2	corvus_brachyrhynchos	105,337,771	105,342,189	Unique
chicken:100K	2	meleagris_gallopavo:falco_peregrinus	105,369,935	105,400,009	Reuse
chicken:100K	2	falco_peregrinus:meleagris_gallopavo	105,369,935	105,400,009	Reuse
chicken:100K	2	anolis_carolinensis	105,512,238	105,519,654	Unique
chicken:100K	2	struthio_camelus	105,901,491	105,905,922	Unique
chicken:100K	2	melopsittacus_undulatus	107,559,592	107,562,725	Unique
chicken:100K	2	pygoscelis_adeliae	108,425,862	108,427,776	Unique
chicken:100K	2	struthio_camelus	109,201,569	109,208,436	Unique
chicken:100K	2	pygoscelis_adeliae	109,557,779	109,559,063	Unique
chicken:100K	2	anas_platyrhynchos	109,781,749	109,784,465	Unique
chicken:100K	2	anolis_carolinensis	111,020,239	111,155,440	Unique
chicken:100K	2	chrysemys_picta	112,189,482	112,198,970	Unique
chicken:100K	2	geospiza_fortis	112,225,596	112,226,472	Unique
chicken:100K	2	melopsittacus_undulatus	114,277,052	114,278,756	Unique
chicken:100K	2	meleagris_gallopavo	114,556,849	114,560,833	Unique
chicken:100K	2	meleagris_gallopavo	114,745,984	114,752,754	Unique
chicken:100K	2	chinese_alligator	115,575,258	115,629,458	Unique
chicken:100K	2	struthio_camelus	116,406,396	116,407,730	Unique
chicken:100K	2	picoides_pubescens	117,065,527	117,076,823	Unique
chicken:100K	2	picoides_pubescens	117,688,316	117,744,886	Unique
chicken:100K	2	picoides_pubescens	117,906,972	117,959,215	Unique
chicken:100K	2	picoides_pubescens	118,141,768	118,158,589	Unique
chicken:100K	2	anolis_carolinensis	118,487,810	118,498,553	Unique
chicken:100K	2	meleagris_gallopavo	118,932,806	118,960,026	Unique
chicken:100K	2	meleagris_gallopavo	119,101,057	119,103,043	Unique
chicken:100K	2	picoides_pubescens	119,947,980	119,976,619	Unique
chicken:100K	2	falco_peregrinus	120,099,558	120,101,405	Unique
chicken:100K	2	struthio_camelus:chrysemys_picta	122,444,754	122,449,354	Reuse
chicken:100K	2	chrysemys_picta:struthio_camelus	122,444,754	122,449,354	Reuse
chicken:100K	2	nipponia_nippon	123,291,867	123,293,778	Unique
chicken:100K	2	chrysemys_picta	124,264,540	124,282,656	Unique
chicken:100K	2	picoides_pubescens	124,360,497	124,371,353	Unique
chicken:100K	2	anolis_carolinensis	124,530,222	124,554,773	Unique
chicken:100K	2	meleagris_gallopavo	125,044,597	125,046,816	Unique
chicken:100K	2	meleagris_gallopavo	125,157,170	125,160,506	Unique
chicken:100K	2	picoides_pubescens	127,250,893	127,255,289	Unique
chicken:100K	2	anolis_carolinensis	127,543,498	127,591,374	Unique
chicken:100K	2	pygoscelis_adeliae	127,901,162	127,901,403	Unique
chicken:100K	2	chrysemys_picta	128,279,856	128,287,485	Unique
chicken:100K	2	aptenodytes_forsteri	128,340,629	128,343,689	Unique
chicken:100K	2	anolis_carolinensis	128,382,165	128,416,026	Unique
chicken:100K	2	struthio_camelus	128,418,469	128,422,204	Unique
chicken:100K	2	meleagris_gallopavo	128,664,062	128,666,320	Unique
chicken:100K	2	picoides_pubescens	128,800,714	128,811,439	Unique
chicken:100K	2	aptenodytes_forsteri	129,424,372	129,428,230	Unique
chicken:100K	2	chrysemys_picta	130,016,627	130,032,147	Unique
chicken:100K	2	anas_platyrhynchos	130,652,087	130,750,987	Unique
chicken:100K	2	struthio_camelus	131,246,844	131,249,972	Unique
chicken:100K	2	chrysemys_picta	131,310,674	131,313,073	Unique
chicken:100K	2	anas_platyrhynchos	131,337,813	131,340,418	Unique
chicken:100K	2	picoides_pubescens	131,634,467	131,653,656	Unique

chicken:100K	2	meleagris_gallopavo	132,025,369	132,026,764	Unique
chicken:100K	2	manacus_vitellinus	132,109,270	132,114,257	Unique
chicken:100K	2	meleagris_gallopavo	132,151,577	132,153,950	Unique
chicken:100K	2	nipponia_nippon:chinese_alligator	132,246,419	132,334,133	Reuse
chicken:100K	2	chinese_alligator:nipponia_nippon	132,246,419	132,334,133	Reuse
chicken:100K	2	chrysemys_picta:chinese_alligator	132,590,839	132,596,715	Reuse
chicken:100K	2	chinese_alligator:chrysemys_picta	132,590,839	132,596,715	Reuse
chicken:100K	2	meleagris_gallopavo	132,930,814	132,933,062	Unique
chicken:100K	2	meleagris_gallopavo	133,134,841	133,146,223	Unique
chicken:100K	2	manacus_vitellinus:columba_livia	135,294,284	135,350,693	Reuse
chicken:100K	2	columba_livia:manacus_vitellinus	135,294,284	135,350,693	Reuse
chicken:100K	2	manacus_vitellinus	135,509,821	135,619,303	Unique
chicken:100K	2	chrysemys_picta	135,864,383	135,878,776	Unique
chicken:100K	2	picoides_pubescens	135,972,434	136,021,056	Unique
chicken:100K	2	chinese_alligator	136,749,435	136,750,158	Unique
chicken:100K	2	chinese_alligator:boa_constrictor	137,383,491	137,387,678	Reuse
chicken:100K	2	boa_constrictor:chinese_alligator	137,383,491	137,387,678	Reuse
chicken:100K	2	picoides_pubescens	137,870,814	137,878,624	Unique
chicken:100K	2	meleagris_gallopavo	139,781,765	139,894,702	Unique
chicken:100K	2	struthio_camelus	140,138,239	140,146,851	Unique
chicken:100K	2	anolis_carolinensis	140,718,237	140,720,664	Unique
chicken:100K	2	struthio_camelus	141,033,454	141,037,107	Unique
chicken:100K	2	struthio_camelus	141,200,439	141,226,824	Unique
chicken:100K	2	meleagris_gallopavo	143,312,821	143,318,425	Unique
chicken:100K	2	meleagris_gallopavo:anolis_carolinensis	143,366,456	143,647,212	Reuse
chicken:100K	2	anolis_carolinensis:meleagris_gallopavo	143,366,456	143,647,212	Reuse
chicken:100K	2	struthio_camelus	144,835,742	144,841,026	Unique
chicken:100K	2	falco_peregrinus	145,287,794	145,290,476	Unique
chicken:100K	2	struthio_camelus	146,842,177	146,845,526	Unique
chicken:100K	2	pygoscelis_adeliae	147,466,569	147,467,486	Unique
chicken:100K	2	falco_peregrinus	147,840,856	147,846,389	Unique
chicken:100K	3	chrysemys_picta	206,420	217,236	Unique
chicken:100K	3	struthio_camelus	357,985	359,236	Unique
chicken:100K	3	egretta_garzetta	366,397	369,617	Unique
chicken:100K	3	anolis_carolinensis	1,980,426	1,982,126	Unique
chicken:100K	3	chicken	2,394,598	2,405,742	Unique
chicken:100K	3	chrysemys_picta	2,580,824	2,626,970	Unique
chicken:100K	3	struthio_camelus	2,995,213	2,998,253	Unique
chicken:100K	3	calypte_anna	3,034,655	3,038,742	Unique
chicken:100K	3	struthio_camelus:picoides_pubescens	3,210,986	3,213,458	Reuse
chicken:100K	3	picoides_pubescens:struthio_camelus	3,210,986	3,213,458	Reuse
chicken:100K	3	struthio_camelus:chaetura_pelagica	3,859,910	3,862,216	Reuse
chicken:100K	3	chaetura_pelagica:struthio_camelus	3,859,910	3,862,216	Reuse
chicken:100K	3	melopsittacus_undulatus	3,872,731	3,873,106	Unique
chicken:100K	3	anolis_carolinensis	4,625,699	4,749,875	Unique
chicken:100K	3	pygoscelis_adeliae	4,840,723	4,840,871	Unique
chicken:100K	3	chicken	5,601,794	5,606,401	Unique
chicken:100K	3	nipponia_nippon	5,923,564	5,925,175	Unique
chicken:100K	3	chrysemys_picta	6,284,321	6,301,569	Unique
chicken:100K	3	chrysemys_picta	6,783,447	6,804,463	Unique
chicken:100K	3	taeniopygia_guttata:cuculus_canorus	7,786,661	7,788,843	Reuse
chicken:100K	3	cuculus_canorus:taeniopygia_guttata	7,786,661	7,788,843	Reuse
chicken:100K	3	anas_platyrhynchos	8,243,627	8,254,163	Unique
chicken:100K	3	chaetura_pelagica	8,416,949	8,438,550	Unique
chicken:100K	3	birds	8,522,203	8,524,853	Unique
chicken:100K	3	anolis_carolinensis	9,561,036	9,575,521	Unique
chicken:100K	3	picoides_pubescens	9,831,945	9,844,759	Unique
chicken:100K	3	chrysemys_picta	10,559,458	10,564,450	Unique
chicken:100K	3	struthio_camelus:falco_peregrinus	11,266,142	11,268,796	Reuse
chicken:100K	3	falco_peregrinus:struthio_camelus	11,266,142	11,268,796	Reuse
chicken:100K	3	calypte_anna	14,907,306	14,909,142	Unique
chicken:100K	3	columba_livia	15,111,847	15,113,579	Unique
chicken:100K	3	columba_livia	15,816,754	15,819,287	Unique
chicken:100K	3	chrysemys_picta	15,888,274	15,895,851	Unique
chicken:100K	3	melopsittacus_undulatus:egretta_garzetta	16,082,207	16,090,698	Reuse
chicken:100K	3	egretta_garzetta:melopsittacus_undulatus	16,082,207	16,090,698	Reuse

chicken:100K	3	calypte_anna	16,094,332	16,095,097	Unique
chicken:100K	3	anas_platyrhynchos	16,559,682	16,561,494	Unique
chicken:100K	3	anolis_carolinensis	16,952,136	16,964,605	Unique
chicken:100K	3	passeroidea	17,042,231	17,043,946	Unique
chicken:100K	3	corvus_brachyrhynchos	17,188,768	17,189,038	Unique
chicken:100K	3	calypte_anna	17,201,733	17,204,955	Unique
chicken:100K	3	struthio_camelus	17,509,222	17,525,202	Unique
chicken:100K	3	columba_livia	17,758,951	17,766,813	Unique
chicken:100K	3	egretta_garzetta	18,084,088	18,085,541	Unique
chicken:100K	3	melopsittacus_undulatus	19,980,016	19,983,687	Unique
chicken:100K	3	melopsittacus_undulatus:chrysemys_picta	20,103,407	20,119,178	Reuse
chicken:100K	3	chrysemys_picta:melopsittacus_undulatus	20,103,407	20,119,178	Reuse
chicken:100K	3	picoides_pubescens	20,356,657	20,392,546	Unique
chicken:100K	3	melopsittacus_undulatus:chrysemys_picta	21,248,586	21,255,599	Reuse
chicken:100K	3	chrysemys_picta:melopsittacus_undulatus	21,248,586	21,255,599	Reuse
chicken:100K	3	melopsittacus_undulatus	21,385,627	21,399,488	Unique
chicken:100K	3	anas_platyrhynchos	21,477,513	21,479,402	Unique
chicken:100K	3	anas_platyrhynchos	22,191,356	22,195,202	Unique
chicken:100K	3	anolis_carolinensis	22,895,372	23,039,116	Unique
chicken:100K	3	chrysemys_picta	23,386,252	23,407,456	Unique
chicken:100K	3	melopsittacus_undulatus	24,447,524	24,475,770	Unique
chicken:100K	3	anolis_carolinensis	25,985,032	26,040,209	Unique
chicken:100K	3	taeniopygia_guttata	26,122,615	26,127,210	Unique
chicken:100K	3	birds	26,543,594	26,565,518	Unique
chicken:100K	3	taeniopygia_guttata	28,438,993	28,441,126	Unique
chicken:100K	3	egretta_garzetta	28,484,486	28,493,547	Unique
chicken:100K	3	anolis_carolinensis	28,776,057	28,776,349	Unique
chicken:100K	3	nipponia_nippon	29,963,955	29,967,318	Unique
chicken:100K	3	anolis_carolinensis	30,131,681	30,172,432	Unique
chicken:100K	3	passeroidea + corvoidea	30,203,130	30,204,703	Unique
chicken:100K	3	anolis_carolinensis	33,363,688	33,366,912	Unique
chicken:100K	3	cuculus_canorus:anolis_carolinensis	34,679,881	34,709,495	Reuse
chicken:100K	3	anolis_carolinensis:cuculus_canorus	34,679,881	34,709,495	Reuse
chicken:100K	3	corvus_brachyrhynchos	35,004,482	35,006,708	Unique
chicken:100K	3	struthio_camelus	37,224,096	37,226,700	Unique
chicken:100K	3	chinese_alligator	37,429,454	37,523,724	Unique
chicken:100K	3	anas_platyrhynchos	37,995,711	38,115,541	Unique
chicken:100K	3	chinese_alligator	38,487,280	38,599,391	Unique
chicken:100K	3	meleagris_gallopavo	38,939,317	38,940,078	Unique
chicken:100K	3	meleagris_gallopavo	39,049,871	39,055,317	Unique
chicken:100K	3	chrysemys_picta	40,151,590	40,166,529	Unique
chicken:100K	3	meleagris_gallopavo	40,945,279	40,965,111	Unique
chicken:100K	3	meleagris_gallopavo	41,102,531	41,111,066	Unique
chicken:100K	3	struthio_camelus	41,747,124	41,750,271	Unique
chicken:100K	3	melopsittacus_undulatus	41,860,916	41,870,781	Unique
chicken:100K	3	meleagris_gallopavo:anolis_carolinensis:calypte_anna	42,163,634	42,165,311	Reuse
chicken:100K	3	calypte_anna:meleagris_gallopavo:anolis_carolinensis	42,163,634	42,165,311	Reuse
chicken:100K	3	anolis_carolinensis:calypte_anna:meleagris_gallopavo	42,163,634	42,165,311	Reuse
chicken:100K	3	anolis_carolinensis	42,668,168	42,674,342	Unique
chicken:100K	3	chinese_alligator	43,098,505	43,188,933	Unique
chicken:100K	3	chrysemys_picta	44,186,247	44,199,568	Unique
chicken:100K	3	ophisthocomus_hoazin	44,428,797	44,430,222	Unique
chicken:100K	3	passeroidea + corvoidea	44,664,812	44,675,084	Unique
chicken:100K	3	egretta_garzetta	45,136,132	45,139,692	Unique
chicken:100K	3	passeroidea + corvoidea	45,151,265	45,152,236	Unique
chicken:100K	3	anolis_carolinensis	47,216,829	47,328,830	Unique
chicken:100K	3	passeroidea + corvoidea	47,422,091	47,423,361	Unique
chicken:100K	3	chinese_alligator	47,795,396	47,796,872	Unique
chicken:100K	3	anolis_carolinensis	48,741,127	48,747,343	Unique
chicken:100K	3	struthio_camelus	49,023,122	49,024,553	Unique
chicken:100K	3	struthio_camelus	49,170,953	49,175,303	Unique
chicken:100K	3	chrysemys_picta	51,152,886	51,186,793	Unique
chicken:100K	3	calypte_anna	51,453,134	51,493,822	Unique
chicken:100K	3	meleagris_gallopavo	52,084,061	52,087,557	Unique
chicken:100K	3	meleagris_gallopavo	52,333,143	52,337,502	Unique
chicken:100K	3	melopsittacus_undulatus	52,415,801	52,432,139	Unique

chicken:100K	3	anolis_carolinensis	52,897,085	52,899,412	Unique
chicken:100K	3	chaetura_pelagica	54,136,314	54,149,774	Unique
chicken:100K	3	chaetura_pelagica	54,471,798	54,477,699	Unique
chicken:100K	3	corvus_brachyrhynchos	54,847,632	54,849,749	Unique
chicken:100K	3	passeroidea	54,850,519	54,851,694	Unique
chicken:100K	3	picoides_pubescens:anolis_carolinensis	56,263,734	56,447,731	Reuse
chicken:100K	3	anolis_carolinensis:picoides_pubescens	56,263,734	56,447,731	Reuse
chicken:100K	3	anolis_carolinensis	56,942,998	56,963,359	Unique
chicken:100K	3	struthio_camelus	58,590,235	58,592,262	Unique
chicken:100K	3	anolis_carolinensis	58,648,633	58,719,814	Unique
chicken:100K	3	falco_peregrinus	60,739,931	60,748,654	Unique
chicken:100K	3	falco_peregrinus	61,923,831	61,930,612	Unique
chicken:100K	3	chinese_alligator	63,138,363	63,202,739	Unique
chicken:100K	3	chrysemys_picta	64,194,437	64,222,418	Unique
chicken:100K	3	chaetura_pelagica	64,225,811	64,231,525	Unique
chicken:100K	3	anolis_carolinensis	65,191,583	65,203,410	Unique
chicken:100K	3	struthio_camelus	65,645,379	65,651,342	Unique
chicken:100K	3	geospiza_fortis	67,404,827	67,409,769	Unique
chicken:100K	3	anolis_carolinensis	68,001,732	68,128,932	Unique
chicken:100K	3	struthio_camelus:meleagris_gallopavo:anolis_carolinensis	68,820,595	69,219,602	Reuse
chicken:100K	3	meleagris_gallopavo:anolis_carolinensis:struthio_camelus	68,820,595	69,219,602	Reuse
chicken:100K	3	anolis_carolinensis:struthio_camelus:meleagris_gallopavo	68,820,595	69,219,602	Reuse
chicken:100K	3	falco_peregrinus	70,640,657	70,678,115	Unique
chicken:100K	3	chinese_alligator	70,982,392	71,295,480	Unique
chicken:100K	3	struthio_camelus:chinese_alligator	71,697,966	71,700,928	Reuse
chicken:100K	3	chinese_alligator:struthio_camelus	71,697,966	71,700,928	Reuse
chicken:100K	3	picoides_pubescens	71,758,857	71,768,026	Unique
chicken:100K	3	picoides_pubescens	72,156,059	72,157,656	Unique
chicken:100K	3	picoides_pubescens:calypte_anna	72,595,143	72,625,072	Reuse
chicken:100K	3	calypte_anna:picoides_pubescens	72,595,143	72,625,072	Reuse
chicken:100K	3	melopsittacus_undulatus	72,670,426	72,673,346	Unique
chicken:100K	3	geospiza_fortis	72,951,449	72,960,997	Unique
chicken:100K	3	meleagris_gallopavo	73,963,450	73,973,424	Unique
chicken:100K	3	melopsittacus_undulatus	74,145,584	74,152,981	Unique
chicken:100K	3	meleagris_gallopavo	74,163,971	74,169,449	Unique
chicken:100K	3	chrysemys_picta	74,404,804	74,419,563	Unique
chicken:100K	3	struthio_camelus	74,816,295	74,819,040	Unique
chicken:100K	3	geospiza_fortis	75,149,623	75,159,429	Unique
chicken:100K	3	egretta_garzetta	75,252,206	75,256,243	Unique
chicken:100K	3	anolis_carolinensis	76,397,482	76,407,626	Unique
chicken:100K	3	chaetura_pelagica	76,762,299	76,768,353	Unique
chicken:100K	3	chaetura_pelagica	77,016,747	77,022,986	Unique
chicken:100K	3	meleagris_gallopavo	77,163,175	77,165,202	Unique
chicken:100K	3	meleagris_gallopavo	77,399,039	77,406,586	Unique
chicken:100K	3	struthio_camelus	79,690,856	79,695,187	Unique
chicken:100K	3	anolis_carolinensis	80,710,111	80,828,467	Unique
chicken:100K	3	calypte_anna	80,926,603	80,929,093	Unique
chicken:100K	3	meleagris_gallopavo	81,156,050	81,161,815	Unique
chicken:100K	3	anolis_carolinensis	81,501,222	81,550,132	Unique
chicken:100K	3	picoides_pubescens	81,819,702	81,827,118	Unique
chicken:100K	3	meleagris_gallopavo	83,102,151	83,113,994	Unique
chicken:100K	3	meleagris_gallopavo	83,349,361	83,362,088	Unique
chicken:100K	3	meleagris_gallopavo	84,220,817	84,340,978	Unique
chicken:100K	3	chinese_alligator	84,572,656	84,611,763	Unique
chicken:100K	3	chinese_alligator	85,094,776	85,318,493	Unique
chicken:100K	3	geospiza_fortis:egretta_garzetta	86,132,809	86,144,687	Reuse
chicken:100K	3	egretta_garzetta:geospiza_fortis	86,132,809	86,144,687	Reuse
chicken:100K	3	chinese_alligator:anolis_carolinensis	86,633,549	86,667,053	Reuse
chicken:100K	3	anolis_carolinensis:chinese_alligator	86,633,549	86,667,053	Reuse
chicken:100K	3	aptenodytes_forsteri	87,175,111	87,177,148	Unique
chicken:100K	3	anolis_carolinensis	87,531,459	87,535,261	Unique
chicken:100K	3	nipponia_nippon	87,633,045	87,636,252	Unique
chicken:100K	3	meleagris_gallopavo:anolis_carolinensis	89,012,517	89,028,451	Reuse
chicken:100K	3	anolis_carolinensis:meleagris_gallopavo	89,012,517	89,028,451	Reuse
chicken:100K	3	struthio_camelus	89,250,610	89,251,944	Unique
chicken:100K	3	ophisthocomus_hoazin	89,596,487	89,598,043	Unique

chicken:100K	3	corvus_brachyrhynchos	90,967,476	90,968,359	Unique
chicken:100K	3	ophisthocomus_hoazin	91,751,499	91,757,455	Unique
chicken:100K	3	meleagris_gallopavo:chinese_alligator	91,924,864	92,470,186	Reuse
chicken:100K	3	chinese_alligator:meleagris_gallopavo	91,924,864	92,470,186	Reuse
chicken:100K	3	picoides_pubescens:anolis_carolinensis	93,364,309	93,487,684	Reuse
chicken:100K	3	anolis_carolinensis:picoides_pubescens	93,364,309	93,487,684	Reuse
chicken:100K	3	anolis_carolinensis	94,181,274	94,227,755	Unique
chicken:100K	3	cuculus_canorus	95,639,534	95,643,712	Unique
chicken:100K	3	cuculus_canorus	96,052,801	96,059,765	Unique
chicken:100K	3	melopsittacus_undulatus	96,354,759	96,361,423	Unique
chicken:100K	3	melopsittacus_undulatus	96,683,466	96,689,726	Unique
chicken:100K	3	struthio_camelus	96,759,713	96,762,581	Unique
chicken:100K	3	chrysemys_picta	97,683,903	97,774,830	Unique
chicken:100K	3	meleagris_gallopavo	98,960,308	99,013,214	Unique
chicken:100K	3	meleagris_gallopavo	99,164,341	99,206,559	Unique
chicken:100K	3	meleagris_gallopavo	99,349,703	99,384,302	Unique
chicken:100K	3	anolis_carolinensis	99,992,424	100,123,044	Unique
chicken:100K	3	chrysemys_picta	100,950,259	100,976,877	Unique
chicken:100K	3	falco_peregrinus:egretta_garzetta	101,387,330	101,389,414	Reuse
chicken:100K	3	egretta_garzetta:falco_peregrinus	101,387,330	101,389,414	Reuse
chicken:100K	3	meleagris_gallopavo:chinese_alligator	101,471,381	101,759,989	Reuse
chicken:100K	3	chinese_alligator:meleagris_gallopavo	101,471,381	101,759,989	Reuse
chicken:100K	3	meleagris_gallopavo:chinese_alligator	101,897,145	101,900,054	Reuse
chicken:100K	3	chinese_alligator:meleagris_gallopavo	101,897,145	101,900,054	Reuse
chicken:100K	3	anolis_carolinensis	102,130,372	102,184,683	Unique
chicken:100K	3	picoides_pubescens	104,504,016	104,512,618	Unique
chicken:100K	3	aptenodytes_forsteri	106,249,832	106,252,426	Unique
chicken:100K	3	chrysemys_picta	108,140,443	108,145,403	Unique
chicken:100K	3	picoides_pubescens	108,411,758	108,419,510	Unique
chicken:100K	3	geospiza_fortis	109,477,105	109,482,975	Unique
chicken:100K	3	struthio_camelus	109,498,583	109,502,406	Unique
chicken:100K	3	meleagris_gallopavo	109,996,565	109,998,189	Unique
chicken:100K	3	struthio_camelus	110,035,357	110,036,998	Unique
chicken:100K	3	struthio_camelus	110,182,870	110,187,257	Unique
chicken:100K	4	melopsittacus_undulatus	350,855	371,562	Unique
chicken:100K	4	meleagris_gallopavo	427,423	504,124	Unique
chicken:100K	4	meleagris_gallopavo	812,908	813,289	Unique
chicken:100K	4	falco_peregrinus	1,179,991	1,186,651	Unique
chicken:100K	4	calypte_anna	1,360,868	1,361,473	Unique
chicken:100K	4	galliformes	1,506,626	1,508,524	Unique
chicken:100K	4	meleagris_gallopavo	1,811,290	1,814,060	Unique
chicken:100K	4	galliformes	1,871,562	1,872,515	Unique
chicken:100K	4	columba_livia	2,017,777	2,021,691	Unique
chicken:100K	4	meleagris_gallopavo:boa_constrictor	2,377,327	2,377,626	Reuse
chicken:100K	4	boa_constrictor:meleagris_gallopavo	2,377,327	2,377,626	Reuse
chicken:100K	4	boa_constrictor	2,507,118	2,510,915	Unique
chicken:100K	4	picoides_pubescens	2,549,081	2,668,478	Unique
chicken:100K	4	passeroidea + corvoidea	2,895,581	2,896,977	Unique
chicken:100K	4	passeroidea + corvoidea	3,388,884	3,392,334	Unique
chicken:100K	4	picoides_pubescens	3,406,167	3,459,595	Unique
chicken:100K	4	picoides_pubescens	3,567,539	3,570,037	Unique
chicken:100K	4	melopsittacus_undulatus	4,091,249	4,093,342	Unique
chicken:100K	4	passeroidea + corvoidea	4,260,909	4,263,947	Unique
chicken:100K	4	chaetura_pelagica	4,337,257	4,340,935	Unique
chicken:100K	4	chinese_alligator	5,370,877	5,443,291	Unique
chicken:100K	4	picoides_pubescens:opossum	6,080,917	6,116,204	Reuse
chicken:100K	4	opossum:picoides_pubescens	6,080,917	6,116,204	Reuse
chicken:100K	4	meleagris_gallopavo	6,441,867	6,442,671	Unique
chicken:100K	4	struthio_camelus	6,760,632	6,762,877	Unique
chicken:100K	4	chrysemys_picta	7,563,985	7,567,574	Unique
chicken:100K	4	struthio_camelus	8,150,883	8,155,344	Unique
chicken:100K	4	struthio_camelus	8,301,512	8,305,390	Unique
chicken:100K	4	aptenodytes_forsteri	8,415,160	8,419,605	Unique
chicken:100K	4	melopsittacus_undulatus	8,675,439	8,684,130	Unique
chicken:100K	4	columba_livia	8,690,791	8,707,251	Unique
chicken:100K	4	struthio_camelus	8,950,221	8,953,754	Unique

chicken:100K	4	galliformes	9,481,895	9,484,589	Unique
chicken:100K	4	struthio_camelus	9,593,532	9,594,700	Unique
chicken:100K	4	struthio_camelus	10,147,290	10,153,808	Unique
chicken:100K	4	chrysemys_picta	10,355,948	10,375,102	Unique
chicken:100K	4	passeroidea + corvoidea	10,431,499	10,437,532	Unique
chicken:100K	4	galliformes	10,936,371	10,942,146	Unique
chicken:100K	4	melopsittacus_undulatus	11,125,860	11,129,794	Unique
chicken:100K	4	cuculus_canorus	11,236,734	11,244,208	Unique
chicken:100K	4	melopsittacus_undulatus	11,377,362	11,382,339	Unique
chicken:100K	4	pygoscelis_adeliae	12,165,129	12,167,935	Unique
chicken:100K	4	cuculus_canorus	12,169,894	12,173,621	Unique
chicken:100K	4	chaetura_pelagica	12,507,219	12,509,445	Unique
chicken:100K	4	ophisthocomus_hoazin	12,525,494	12,527,715	Unique
chicken:100K	4	columba_livia	12,835,131	12,836,249	Unique
chicken:100K	4	manacus_vitellinus	13,170,258	13,172,768	Unique
chicken:100K	4	melopsittacus_undulatus	13,246,243	13,283,396	Unique
chicken:100K	4	passeroidea + corvoidea	13,340,755	13,345,292	Unique
chicken:100K	4	columba_livia	13,756,707	13,792,817	Unique
chicken:100K	4	columba_livia	13,912,312	13,932,288	Unique
chicken:100K	4	melopsittacus_undulatus:anas_platyrhynchos	14,061,412	14,063,254	Reuse
chicken:100K	4	anas_platyrhynchos:melopsittacus_undulatus	14,061,412	14,063,254	Reuse
chicken:100K	4	birds_crocs_turtles	14,093,664	14,104,963	Unique
chicken:100K	4	birds_crocs_turtles	14,266,768	14,277,534	Unique
chicken:100K	4	pygoscelis_adeliae	14,751,165	14,752,285	Unique
chicken:100K	4	chrysemys_picta	15,023,667	15,035,077	Unique
chicken:100K	4	columba_livia	15,343,102	15,345,468	Unique
chicken:100K	4	boa_constrictor	15,484,465	15,532,803	Unique
chicken:100K	4	falco_peregrinus	15,690,999	15,704,255	Unique
chicken:100K	4	opossum:nipponia_nippon	16,069,619	16,075,663	Reuse
chicken:100K	4	nipponia_nippon:opossum	16,069,619	16,075,663	Reuse
chicken:100K	4	columba_livia:anas_platyrhynchos	16,436,980	16,445,038	Reuse
chicken:100K	4	anas_platyrhynchos:columba_livia	16,436,980	16,445,038	Reuse
chicken:100K	4	taeniopygia_guttata	17,284,492	17,286,345	Unique
chicken:100K	4	geospiza_fortis	17,286,387	17,286,591	Unique
chicken:100K	4	calypte_anna	17,527,648	17,530,858	Unique
chicken:100K	4	melopsittacus_undulatus	17,972,089	17,974,013	Unique
chicken:100K	4	columba_livia	17,983,103	18,147,698	Unique
chicken:100K	4	chicken	19,197,800	19,204,786	Unique
chicken:100K	4	pygoscelis_adeliae	20,473,226	20,476,609	Unique
chicken:100K	4	galliformes	20,643,509	20,643,611	Unique
chicken:100K	4	egretta_garzetta:corvus_brachyrhynchos	22,618,556	22,670,686	Reuse
chicken:100K	4	corvus_brachyrhynchos:egretta_garzetta	22,618,556	22,670,686	Reuse
chicken:100K	4	chrysemys_picta	23,069,953	23,120,351	Unique
chicken:100K	4	melopsittacus_undulatus	23,169,805	23,185,692	Unique
chicken:100K	4	anolis_carolinensis	25,685,215	25,803,724	Unique
chicken:100K	4	meleagris_gallopavo	27,387,931	27,394,237	Unique
chicken:100K	4	pygoscelis_adeliae	27,504,866	27,507,960	Unique
chicken:100K	4	non_galloanserae	27,578,603	27,578,778	Unique
chicken:100K	4	chrysemys_picta	27,640,522	27,664,849	Unique
chicken:100K	4	taeniopygia_guttata	28,822,025	28,824,435	Unique
chicken:100K	4	passeroidea + corvoidea	30,097,133	30,102,301	Unique
chicken:100K	4	melopsittacus_undulatus:chinese_alligator	30,420,803	30,426,405	Reuse
chicken:100K	4	chinese_alligator:melopsittacus_undulatus	30,420,803	30,426,405	Reuse
chicken:100K	4	chinese_alligator:anolis_carolinensis	30,767,868	30,973,071	Reuse
chicken:100K	4	anolis_carolinensis:chinese_alligator	30,767,868	30,973,071	Reuse
chicken:100K	4	picoides_pubescens:chinese_alligator	31,426,037	31,426,428	Reuse
chicken:100K	4	chinese_alligator:picoides_pubescens	31,426,037	31,426,428	Reuse
chicken:100K	4	non_galloanserae	32,678,060	32,682,020	Unique
chicken:100K	4	chinese_alligator	32,771,900	32,774,979	Unique
chicken:100K	4	chinese_alligator	33,108,595	33,360,888	Unique
chicken:100K	4	galliformes	33,678,769	33,680,734	Unique
chicken:100K	4	melopsittacus_undulatus:anolis_carolinensis	33,910,120	33,918,223	Reuse
chicken:100K	4	anolis_carolinensis:melopsittacus_undulatus	33,910,120	33,918,223	Reuse
chicken:100K	4	galliformes	34,041,961	34,043,296	Unique
chicken:100K	4	picoides_pubescens:columba_livia	34,798,938	34,800,687	Reuse
chicken:100K	4	columba_livia:picoides_pubescens	34,798,938	34,800,687	Reuse

chicken:100K	4	galliformes	36,711,308	36,715,137	Unique
chicken:100K	4	anolis_carolinensis	37,171,141	37,208,386	Unique
chicken:100K	4	non_galloanserae	38,688,770	38,696,302	Unique
chicken:100K	4	picoides_pubescens	39,233,077	39,252,203	Unique
chicken:100K	4	anolis_carolinensis	39,417,530	39,426,773	Unique
chicken:100K	4	anolis_carolinensis	39,732,598	39,766,567	Unique
chicken:100K	4	chrysemys_picta	40,162,034	40,165,426	Unique
chicken:100K	4	melopsittacus_undulatus	40,497,561	40,504,359	Unique
chicken:100K	4	galliformes	41,740,401	41,746,362	Unique
chicken:100K	4	anas_platyrhynchos	43,704,832	43,707,372	Unique
chicken:100K	4	anolis_carolinensis	44,504,381	44,506,617	Unique
chicken:100K	4	chaetura_pelagica	44,735,362	44,742,253	Unique
chicken:100K	4	cuculus_canorus	45,218,802	45,225,642	Unique
chicken:100K	4	neognathae	45,357,081	45,396,544	Unique
chicken:100K	4	aptenodytes_forsteri	45,517,540	45,518,456	Unique
chicken:100K	4	chrysemys_picta	45,675,798	45,690,262	Unique
chicken:100K	4	galloanserae	45,834,325	45,835,272	Unique
chicken:100K	4	anas_platyrhynchos	45,892,671	45,894,847	Unique
chicken:100K	4	taeniopygia_guttata	46,253,311	46,253,393	Unique
chicken:100K	4	galliformes	46,436,850	46,444,272	Unique
chicken:100K	4	melopsittacus_undulatus	46,691,091	46,694,234	Unique
chicken:100K	4	columba_livia:chaetura_pelagica	46,896,853	46,897,652	Reuse
chicken:100K	4	chaetura_pelagica:columba_livia	46,896,853	46,897,652	Reuse
chicken:100K	4	columba_livia	47,187,807	47,198,613	Unique
chicken:100K	4	galliformes	48,573,136	48,577,729	Unique
chicken:100K	4	geospiza_fortis	48,930,411	48,932,882	Unique
chicken:100K	4	galliformes	49,042,431	49,044,177	Unique
chicken:100K	4	opossum:meleagris_gallopavo	49,141,083	49,201,284	Reuse
chicken:100K	4	meleagris_gallopavo:opossum	49,141,083	49,201,284	Reuse
chicken:100K	4	melopsittacus_undulatus	49,553,668	49,555,398	Unique
chicken:100K	4	ophisthocomus_hoazin	50,131,438	50,141,635	Unique
chicken:100K	4	manacus_vitellinus	50,266,677	50,279,477	Unique
chicken:100K	4	calypte_anna	50,683,241	50,687,737	Unique
chicken:100K	4	calypte_anna	50,820,926	50,825,225	Unique
chicken:100K	4	columba_livia	51,018,370	51,028,380	Unique
chicken:100K	4	galliformes	51,215,264	51,216,208	Unique
chicken:100K	4	charadrius_vociferus	51,952,799	51,959,633	Unique
chicken:100K	4	melopsittacus_undulatus	52,379,462	52,406,329	Unique
chicken:100K	4	falco_peregrinus	53,138,363	53,144,877	Unique
chicken:100K	4	melopsittacus_undulatus:chinese_alligator	53,413,857	53,415,016	Reuse
chicken:100K	4	chinese_alligator:melopsittacus_undulatus	53,413,857	53,415,016	Reuse
chicken:100K	4	calypte_anna	53,862,071	53,866,748	Unique
chicken:100K	4	aptenodytes_forsteri	54,323,333	54,325,858	Unique
chicken:100K	4	anas_platyrhynchos	54,333,412	54,385,406	Unique
chicken:100K	4	melopsittacus_undulatus	54,952,620	54,954,385	Unique
chicken:100K	4	anas_platyrhynchos	55,401,395	55,403,856	Unique
chicken:100K	4	geospiza_fortis:calypte_anna	56,324,208	56,325,606	Reuse
chicken:100K	4	calypte_anna:geospiza_fortis	56,324,208	56,325,606	Reuse
chicken:100K	4	chrysemys_picta	56,633,851	56,660,299	Unique
chicken:100K	4	non_galloanserae/non_columbiformes	56,811,761	56,814,510	Unique
chicken:100K	4	galliformes	57,799,990	57,801,833	Unique
chicken:100K	4	anolis_carolinensis	58,463,159	58,603,740	Unique
chicken:100K	4	struthio_camelus	59,491,056	59,495,659	Unique
chicken:100K	4	geospiza_fortis:anas_platyrhynchos	59,574,836	59,582,676	Reuse
chicken:100K	4	anas_platyrhynchos:geospiza_fortis	59,574,836	59,582,676	Reuse
chicken:100K	4	calypte_anna	59,617,719	59,629,956	Unique
chicken:100K	4	chrysemys_picta	60,447,123	60,462,530	Unique
chicken:100K	4	falco_peregrinus	60,900,023	60,902,283	Unique
chicken:100K	4	anas_platyrhynchos	61,229,717	61,233,280	Unique
chicken:100K	4	falco_peregrinus	61,295,871	61,303,265	Unique
chicken:100K	4	anas_platyrhynchos	62,815,625	62,817,700	Unique
chicken:100K	4	picoides_pubescens	62,931,516	62,990,410	Unique
chicken:100K	4	anas_platyrhynchos	63,850,150	63,927,173	Unique
chicken:100K	4	chrysemys_picta	64,055,029	64,077,495	Unique
chicken:100K	4	picoides_pubescens	64,616,993	64,631,141	Unique
chicken:100K	4	anolis_carolinensis	65,443,231	65,502,021	Unique

chicken:100K	4	anolis_carolinensis	66,618,822	66,621,015	Unique
chicken:100K	4	pygoscelis_adeliae	66,957,086	66,959,875	Unique
chicken:100K	4	ophisthocomus_hoazin	67,182,750	67,188,828	Unique
chicken:100K	4	chrysemys_picta	68,383,438	68,405,576	Unique
chicken:100K	4	falco_peregrinus	68,582,526	68,583,205	Unique
chicken:100K	4	meleagris_gallopavo	69,534,009	69,536,818	Unique
chicken:100K	4	chrysemys_picta	69,624,766	69,634,790	Unique
chicken:100K	4	egretta_garzetta	69,903,299	69,905,503	Unique
chicken:100K	4	chrysemys_picta	70,703,860	70,717,596	Unique
chicken:100K	4	melopsittacus_undulatus	70,906,639	71,104,923	Unique
chicken:100K	4	meleagris_gallopavo:chrysemys_picta	71,223,933	71,238,522	Reuse
chicken:100K	4	chrysemys_picta:meleagris_gallopavo	71,223,933	71,238,522	Reuse
chicken:100K	4	chaetura_pelagica	71,820,265	71,835,054	Unique
chicken:100K	4	chaetura_pelagica	71,949,677	71,953,594	Unique
chicken:100K	4	picoides_pubescens	72,985,398	72,991,240	Unique
chicken:100K	4	anolis_carolinensis	73,136,251	73,253,034	Unique
chicken:100K	4	pygoscelis_adeliae	73,555,682	73,572,126	Unique
chicken:100K	4	chinese_alligator	75,215,000	75,224,696	Unique
chicken:100K	4	chinese_alligator	75,338,157	75,341,080	Unique
chicken:100K	4	falco_peregrinus:anolis_carolinensis	75,554,298	75,569,958	Reuse
chicken:100K	4	anolis_carolinensis:falco_peregrinus	75,554,298	75,569,958	Reuse
chicken:100K	4	picoides_pubescens	76,310,639	76,321,489	Unique
chicken:100K	4	anolis_carolinensis	77,148,485	77,251,561	Unique
chicken:100K	4	calypte_anna	77,455,079	77,457,798	Unique
chicken:100K	4	calypte_anna	77,588,336	77,592,319	Unique
chicken:100K	4	boa_constrictor	78,550,227	79,297,682	Unique
chicken:100K	4	picoides_pubescens:calypte_anna	79,518,738	79,526,898	Reuse
chicken:100K	4	calypte_anna:picoides_pubescens	79,518,738	79,526,898	Reuse
chicken:100K	4	chrysemys_picta	79,805,612	79,827,283	Unique
chicken:100K	4	meleagris_gallopavo	79,978,630	79,979,564	Unique
chicken:100K	4	chrysemys_picta	80,394,815	80,405,085	Unique
chicken:100K	4	meleagris_gallopavo	80,965,077	80,969,237	Unique
chicken:100K	4	pygoscelis_adeliae	84,316,611	84,321,659	Unique
chicken:100K	4	picoides_pubescens	84,825,333	84,858,437	Unique
chicken:100K	4	anolis_carolinensis	85,225,245	85,278,542	Unique
chicken:100K	4	picoides_pubescens	88,166,627	88,170,273	Unique
chicken:100K	4	manacus_vitellinus:chinese_alligator	90,019,083	90,025,186	Reuse
chicken:100K	4	chinese_alligator:manacus_vitellinus	90,019,083	90,025,186	Reuse
chicken:100K	4	anas_platyrhynchos	90,053,050	90,053,324	Unique
chicken:100K	5	cuculus_canorus	765,260	766,113	Unique
chicken:100K	5	chrysemys_picta	868,172	905,494	Unique
chicken:100K	5	Passeriformes + Psittaciformes + Falconiformes + Piciformes + Ciconiiformes + Sphenisciformes + Charadriiformes + Opisthocomiformes	1,027,610	1,064,987	Unique
chicken:100K	5	picoides_pubescens	2,125,295	2,132,702	Unique
chicken:100K	5	chrysemys_picta	2,304,870	2,309,984	Unique
chicken:100K	5	egretta_garzetta	2,721,940	2,726,022	Unique
chicken:100K	5	passeroidea	3,544,477	3,564,577	Unique
chicken:100K	5	chrysemys_picta	3,824,468	3,832,483	Unique
chicken:100K	5	geospiza_fortis	3,919,089	3,919,845	Unique
chicken:100K	5	taeniopygia_guttata	3,920,049	3,922,152	Unique
chicken:100K	5	chrysemys_picta	4,045,737	4,065,269	Unique
chicken:100K	5	passeroidea	4,225,299	4,227,824	Unique
chicken:100K	5	anolis_carolinensis	4,316,712	4,503,798	Unique
chicken:100K	5	cuculus_canorus	4,587,874	4,590,422	Unique
chicken:100K	5	chicken	5,679,219	5,833,354	Unique
chicken:100K	5	chicken	6,518,476	6,520,088	Unique
chicken:100K	5	anas_platyrhynchos	6,693,728	6,697,344	Unique
chicken:100K	5	melopsittacus_undulatus	6,743,376	6,752,713	Unique
chicken:100K	5	anas_platyrhynchos	6,856,003	6,859,157	Unique
chicken:100K	5	melopsittacus_undulatus	7,667,880	7,674,323	Unique
chicken:100K	5	anolis_carolinensis	7,894,600	7,911,483	Unique
chicken:100K	5	passeroidea + corvoidea	8,135,601	8,136,881	Unique
chicken:100K	5	chrysemys_picta	8,267,136	8,287,747	Unique
chicken:100K	5	melopsittacus_undulatus	8,632,625	8,635,023	Unique
chicken:100K	5	chinese_alligator	8,790,208	8,805,358	Unique
chicken:100K	5	ophisthocomus_hoazin	8,921,151	8,923,103	Unique

chicken:100K	5	anolis_carolinensis	9,169,792	9,178,301	Unique
chicken:100K	5	pygoscelis_adeliae	9,626,194	9,627,370	Unique
chicken:100K	5	chrysemys_picta:anolis_carolinensis	9,926,444	9,938,611	Reuse
chicken:100K	5	anolis_carolinensis:chrysemys_picta	9,926,444	9,938,611	Reuse
chicken:100K	5	pygoscelis_adeliae	10,083,985	10,087,654	Unique
chicken:100K	5	anas_platyrhynchos	11,834,485	11,835,095	Unique
chicken:100K	5	picoides_pubescens	11,869,538	11,876,973	Unique
chicken:100K	5	falco_peregrinus	11,919,510	11,925,310	Unique
chicken:100K	5	melopsittacus_undulatus	12,271,223	12,273,217	Unique
chicken:100K	5	meleagris_gallopavo	12,280,486	12,286,154	Unique
chicken:100K	5	chrysemys_picta	12,487,037	12,488,179	Unique
chicken:100K	5	picoides_pubescens	13,363,741	13,366,143	Unique
chicken:100K	5	picoides_pubescens	13,572,030	13,576,897	Unique
chicken:100K	5	falco_peregrinus	13,689,059	13,693,819	Unique
chicken:100K	5	falco_peregrinus	13,977,455	13,980,292	Unique
chicken:100K	5	calypte_anna:anolis_carolinensis	14,131,378	14,139,637	Reuse
chicken:100K	5	anolis_carolinensis:calypte_anna	14,131,378	14,139,637	Reuse
chicken:100K	5	anas_platyrhynchos	14,417,500	14,449,735	Unique
chicken:100K	5	chrysemys_picta	15,127,066	15,141,956	Unique
chicken:100K	5	calypte_anna	15,289,619	15,298,392	Unique
chicken:100K	5	melopsittacus_undulatus	15,656,769	15,673,725	Unique
chicken:100K	5	anas_platyrhynchos	15,830,289	15,834,356	Unique
chicken:100K	5	galloanserae	16,416,680	16,419,624	Unique
chicken:100K	5	anolis_carolinensis	17,071,363	17,082,930	Unique
chicken:100K	5	melopsittacus_undulatus	17,115,509	17,116,760	Unique
chicken:100K	5	meleagris_gallopavo	17,818,304	17,823,440	Unique
chicken:100K	5	meleagris_gallopavo	17,956,187	17,960,109	Unique
chicken:100K	5	non_galloanserae	18,001,580	18,002,530	Unique
chicken:100K	5	anolis_carolinensis	18,203,045	18,208,443	Unique
chicken:100K	5	picoides_pubescens	18,324,388	18,332,785	Unique
chicken:100K	5	picoides_pubescens	18,439,450	18,443,825	Unique
chicken:100K	5	aptenodytes_forsteri	18,887,941	18,890,453	Unique
chicken:100K	5	picoides_pubescens:melopsittacus_undulatus	19,009,540	19,532,308	Reuse
chicken:100K	5	melopsittacus_undulatus:picoides_pubescens	19,009,540	19,532,308	Reuse
chicken:100K	5	anolis_carolinensis	19,720,786	19,741,068	Unique
chicken:100K	5	meleagris_gallopavo	20,442,017	20,486,433	Unique
chicken:100K	5	meleagris_gallopavo	20,627,816	20,635,605	Unique
chicken:100K	5	melopsittacus_undulatus	20,700,162	20,702,847	Unique
chicken:100K	5	anolis_carolinensis	20,910,906	20,954,787	Unique
chicken:100K	5	picoides_pubescens	21,304,496	21,307,358	Unique
chicken:100K	5	falco_peregrinus	22,196,828	22,198,798	Unique
chicken:100K	5	anas_platyrhynchos	22,450,240	22,452,053	Unique
chicken:100K	5	anolis_carolinensis	23,109,978	23,166,405	Unique
chicken:100K	5	melopsittacus_undulatus	23,198,288	23,217,253	Unique
chicken:100K	5	meleagris_gallopavo	23,916,351	23,917,972	Unique
chicken:100K	5	meleagris_gallopavo	24,030,454	24,041,047	Unique
chicken:100K	5	cuculus_canorus:chrysemys_picta	24,154,193	24,157,428	Reuse
chicken:100K	5	chrysemys_picta:cuculus_canorus	24,154,193	24,157,428	Reuse
chicken:100K	5	chrysemys_picta	24,427,705	24,458,703	Unique
chicken:100K	5	calypte_anna	24,489,237	24,572,118	Unique
chicken:100K	5	falco_peregrinus	24,751,245	24,843,688	Unique
chicken:100K	5	boa_constrictor	25,459,124	25,459,863	Unique
chicken:100K	5	melopsittacus_undulatus	25,468,023	25,480,055	Unique
chicken:100K	5	melopsittacus_undulatus	25,757,681	25,760,117	Unique
chicken:100K	5	chrysemys_picta	26,058,171	26,067,282	Unique
chicken:100K	5	melopsittacus_undulatus	26,818,758	26,820,709	Unique
chicken:100K	5	calypte_anna	27,252,898	27,255,611	Unique
chicken:100K	5	picoides_pubescens	28,318,936	28,326,476	Unique
chicken:100K	5	birds	28,573,512	28,617,189	Unique
chicken:100K	5	anolis_carolinensis	28,908,090	28,956,673	Unique
chicken:100K	5	aptenodytes_forsteri	29,655,314	29,659,106	Unique
chicken:100K	5	chrysemys_picta	30,022,977	30,029,577	Unique
chicken:100K	5	chrysemys_picta	32,357,176	32,366,436	Unique
chicken:100K	5	meleagris_gallopavo	33,160,597	33,164,011	Unique
chicken:100K	5	anolis_carolinensis	33,433,365	33,437,213	Unique
chicken:100K	5	picoides_pubescens	34,346,931	34,353,081	Unique

chicken:100K	5	meleagris_gallopavo	34,867,481	34,872,954	Unique
chicken:100K	5	meleagris_gallopavo	35,006,689	35,013,251	Unique
chicken:100K	5	struthio_camelus	35,340,069	35,343,565	Unique
chicken:100K	5	anas_platyrhynchos	35,400,231	35,402,003	Unique
chicken:100K	5	chrysemys_picta	35,445,149	35,470,094	Unique
chicken:100K	5	anolis_carolinensis	35,512,215	35,565,936	Unique
chicken:100K	5	meleagris_gallopavo	35,576,058	35,589,312	Unique
chicken:100K	5	melopsittacus_undulatus	35,619,779	35,622,065	Unique
chicken:100K	5	anas_platyrhynchos	35,997,809	36,000,902	Unique
chicken:100K	5	chrysemys_picta	36,428,161	36,433,460	Unique
chicken:100K	5	anolis_carolinensis	36,776,202	36,873,114	Unique
chicken:100K	5	cuculus_canorus	38,160,605	38,163,150	Unique
chicken:100K	5	chrysemys_picta	39,036,097	39,040,675	Unique
chicken:100K	5	struthio_camelus	39,478,845	39,483,743	Unique
chicken:100K	5	cuculus_canorus	39,877,213	39,879,963	Unique
chicken:100K	5	melopsittacus_undulatus	40,606,713	40,607,711	Unique
chicken:100K	5	columba_livia	40,665,767	40,666,475	Unique
chicken:100K	5	columba_livia	40,909,649	40,912,128	Unique
chicken:100K	5	chrysemys_picta	41,225,010	41,229,777	Unique
chicken:100K	5	melopsittacus_undulatus	42,216,442	42,247,279	Unique
chicken:100K	5	falco_peregrinus	42,299,220	42,302,334	Unique
chicken:100K	5	egretta_garzetta	43,289,358	43,292,153	Unique
chicken:100K	5	charadrius_vociferus	43,528,031	43,530,038	Unique
chicken:100K	5	chrysemys_picta	45,355,383	45,379,615	Unique
chicken:100K	5	charadrius_vociferus	45,872,562	45,879,177	Unique
chicken:100K	5	meleagris_gallopavo	47,151,314	47,286,084	Unique
chicken:100K	5	meleagris_gallopavo	47,675,693	47,700,934	Unique
chicken:100K	5	anolis_carolinensis	47,867,617	47,870,321	Unique
chicken:100K	5	chrysemys_picta	47,950,958	47,996,300	Unique
chicken:100K	5	chrysemys_picta	48,582,743	48,605,251	Unique
chicken:100K	5	chrysemys_picta	49,911,179	49,927,169	Unique
chicken:100K	5	egretta_garzetta	50,079,800	50,081,094	Unique
chicken:100K	5	anolis_carolinensis	50,250,870	50,254,191	Unique
chicken:100K	5	falco_peregrinus	50,533,007	50,535,907	Unique
chicken:100K	5	cuculus_canorus	51,321,714	51,324,489	Unique
chicken:100K	5	cuculus_canorus	51,613,362	51,616,661	Unique
chicken:100K	5	chrysemys_picta	51,735,453	51,736,113	Unique
chicken:100K	5	boa_constrictor	51,784,742	51,799,655	Unique
chicken:100K	5	corvus_brachyrhynchos	52,026,935	52,030,607	Unique
chicken:100K	5	chrysemys_picta	52,773,660	52,786,177	Unique
chicken:100K	5	ophisthocomus_hoazin	52,958,989	52,961,637	Unique
chicken:100K	5	chinese_alligator	53,672,846	53,750,240	Unique
chicken:100K	5	anolis_carolinensis	54,033,130	54,036,038	Unique
chicken:100K	5	chrysemys_picta	54,214,985	54,216,340	Unique
chicken:100K	5	chrysemys_picta	54,462,789	54,480,287	Unique
chicken:100K	5	melopsittacus_undulatus:cuculus_canorus	54,830,177	54,830,778	Reuse
chicken:100K	5	cuculus_canorus:melopsittacus_undulatus	54,830,177	54,830,778	Reuse
chicken:100K	5	chrysemys_picta	54,929,523	54,942,828	Unique
chicken:100K	5	columba_livia:boa_constrictor:charadrius_vociferus	55,173,216	55,177,208	Reuse
chicken:100K	5	charadrius_vociferus:columba_livia:boa_constrictor	55,173,216	55,177,208	Reuse
chicken:100K	5	boa_constrictor:charadrius_vociferus:columba_livia	55,173,216	55,177,208	Reuse
chicken:100K	5	melopsittacus_undulatus	55,196,523	55,198,363	Unique
chicken:100K	5	cuculus_canorus	55,425,572	55,430,766	Unique
chicken:100K	5	pygoscelis_adeliae	55,487,902	55,489,159	Unique
chicken:100K	5	meleagris_gallopavo	55,799,598	55,802,625	Unique
chicken:100K	5	ophisthocomus_hoazin	55,936,856	55,939,468	Unique
chicken:100K	5	boa_constrictor	56,032,958	56,056,367	Unique
chicken:100K	5	meleagris_gallopavo	56,158,749	56,162,953	Unique
chicken:100K	5	meleagris_gallopavo	56,325,865	56,338,919	Unique
chicken:100K	5	galliformes	56,369,968	56,371,735	Unique
chicken:100K	5	meleagris_gallopavo	57,143,387	57,144,279	Unique
chicken:100K	5	melopsittacus_undulatus	57,288,091	57,289,380	Unique
chicken:100K	5	meleagris_gallopavo	57,316,908	57,318,211	Unique
chicken:100K	5	chrysemys_picta:chinese_alligator	57,565,322	57,584,067	Reuse
chicken:100K	5	chinese_alligator:chrysemys_picta	57,565,322	57,584,067	Reuse
chicken:100K	5	columba_livia:boa_constrictor	57,913,559	57,915,279	Reuse

chicken:100K	5	boa_constrictor:columba_livia	57,913,559	57,915,279	Reuse
chicken:100K	5	chinese_alligator	57,966,350	57,979,046	Unique
chicken:100K	5	cuculus_canorus	58,109,709	58,112,939	Unique
chicken:100K	5	chaetura_pelagica	58,118,755	58,124,910	Unique
chicken:100K	5	boa_constrictor	58,265,095	58,286,222	Unique
chicken:100K	5	chrysemys_picta	58,287,512	58,306,393	Unique
chicken:100K	5	galliformes	58,601,543	58,603,094	Unique
chicken:100K	5	meleagris_gallopavo:chinese_alligator	58,782,991	58,894,072	Reuse
chicken:100K	5	chinese_alligator:meleagris_gallopavo	58,782,991	58,894,072	Reuse
chicken:100K	5	meleagris_gallopavo	59,009,816	59,015,918	Unique
chicken:100K	6	cuculus_canorus	466,944	524,005	Unique
chicken:100K	6	chrysemys_picta	797,738	1,471,274	Unique
chicken:100K	6	opossum:columba_livia	2,110,699	2,130,723	Reuse
chicken:100K	6	columba_livia:opossum	2,110,699	2,130,723	Reuse
chicken:100K	6	melopsittacus_undulatus:chrysemys_picta	4,003,881	4,013,900	Reuse
chicken:100K	6	chrysemys_picta:melopsittacus_undulatus	4,003,881	4,013,900	Reuse
chicken:100K	6	chicken	6,070,296	6,073,929	Unique
chicken:100K	6	picoides_pubescens	6,615,697	6,617,856	Unique
chicken:100K	6	picoides_pubescens	6,803,568	6,807,109	Unique
chicken:100K	6	passeroidea	7,198,198	7,210,644	Unique
chicken:100K	6	meleagris_gallopavo	7,364,323	7,365,442	Unique
chicken:100K	6	meleagris_gallopavo	7,525,715	7,663,286	Unique
chicken:100K	6	meleagris_gallopavo	7,984,345	8,108,082	Unique
chicken:100K	6	chicken	8,674,888	8,794,826	Unique
chicken:100K	6	columba_livia	8,907,088	8,912,893	Unique
chicken:100K	6	meleagris_gallopavo	10,185,847	10,205,763	Unique
chicken:100K	6	cuculus_canorus	10,355,289	10,370,772	Unique
chicken:100K	6	picoides_pubescens	10,391,775	10,394,627	Unique
chicken:100K	6	chrysemys_picta	10,415,989	10,423,923	Unique
chicken:100K	6	passeroidea + corvoidea	11,150,025	11,152,942	Unique
chicken:100K	6	pygoscelis_adeliae	11,882,368	11,884,640	Unique
chicken:100K	6	chrysemys_picta	12,325,044	12,337,513	Unique
chicken:100K	6	columba_livia	12,714,330	12,716,660	Unique
chicken:100K	6	meleagris_gallopavo	13,028,041	13,028,127	Unique
chicken:100K	6	chrysemys_picta	13,192,400	13,207,895	Unique
chicken:100K	6	ophisthocomus_hoazin	13,358,851	13,361,102	Unique
chicken:100K	6	meleagris_gallopavo	13,789,971	13,790,546	Unique
chicken:100K	6	chrysemys_picta	13,923,466	13,937,220	Unique
chicken:100K	6	pygoscelis_adeliae	14,310,596	14,315,071	Unique
chicken:100K	6	falco_peregrinus	14,954,274	14,959,018	Unique
chicken:100K	6	chrysemys_picta	15,089,676	15,092,620	Unique
chicken:100K	6	melopsittacus_undulatus	15,571,507	15,572,968	Unique
chicken:100K	6	cuculus_canorus	16,119,290	16,124,876	Unique
chicken:100K	6	meleagris_gallopavo	16,708,639	16,725,881	Unique
chicken:100K	6	calypte_anna	17,069,464	17,076,358	Unique
chicken:100K	6	falco_peregrinus	17,179,805	17,225,601	Unique
chicken:100K	6	chrysemys_picta	19,929,389	19,942,492	Unique
chicken:100K	6	pygoscelis_adeliae	20,287,657	20,292,438	Unique
chicken:100K	6	anolis_carolinensis	21,865,643	21,932,868	Unique
chicken:100K	6	charadrius_vociferus	22,000,257	22,002,311	Unique
chicken:100K	6	melopsittacus_undulatus	22,070,821	22,074,658	Unique
chicken:100K	6	anolis_carolinensis	22,620,021	22,622,920	Unique
chicken:100K	6	charadrius_vociferus	22,717,531	22,721,730	Unique
chicken:100K	6	chinese_alligator	22,959,518	22,961,467	Unique
chicken:100K	6	cuculus_canorus	25,352,314	25,354,524	Unique
chicken:100K	6	anolis_carolinensis	26,133,517	26,136,405	Unique
chicken:100K	6	anolis_carolinensis	29,729,668	29,766,626	Unique
chicken:100K	6	falco_peregrinus	29,922,315	29,936,724	Unique
chicken:100K	6	anolis_carolinensis	30,915,593	30,918,433	Unique
chicken:100K	6	meleagris_gallopavo	31,230,122	31,234,210	Unique
chicken:100K	6	anolis_carolinensis	32,351,678	32,352,864	Unique
chicken:100K	6	meleagris_gallopavo	33,928,861	33,933,761	Unique
chicken:100K	6	chrysemys_picta	34,056,707	34,087,323	Unique
chicken:100K	6	nipponia_nippon	34,209,014	34,214,391	Unique
chicken:100K	6	nipponia_nippon:melopsittacus_undulatus	34,559,088	34,561,656	Reuse
chicken:100K	6	melopsittacus_undulatus:nipponia_nippon	34,559,088	34,561,656	Reuse

chicken:100K	7	cuculus_canorus	287,391	297,039	Unique
chicken:100K	7	manacus_vitellinus	398,412	404,927	Unique
chicken:100K	7	chrysemys_picta	463,663	498,861	Unique
chicken:100K	7	falco_peregrinus:cuculus_canorus	844,854	996,135	Reuse
chicken:100K	7	cuculus_canorus:falco_peregrinus	844,854	996,135	Reuse
chicken:100K	7	chrysemys_picta	1,339,266	1,351,094	Unique
chicken:100K	7	columba_livia	1,394,819	1,395,144	Unique
chicken:100K	7	picoides_pubescens:passeroidea + corvoidea	2,187,265	2,190,166	Reuse
chicken:100K	7	passeroidea + corvoidea:picoides_pubescens	2,187,265	2,190,166	Reuse
chicken:100K	7	manacus_vitellinus	2,204,681	2,210,435	Unique
chicken:100K	7	picoides_pubescens	2,762,751	2,772,057	Unique
chicken:100K	7	taeniopygia_guttata:meleagris_gallopavo:picoides_pubescens	3,530,070	3,533,623	Reuse
chicken:100K	7	picoides_pubescens:taeniopygia_guttata:meleagris_gallopavo	3,530,070	3,533,623	Reuse
chicken:100K	7	meleagris_gallopavo:picoides_pubescens:taeniopygia_guttata	3,530,070	3,533,623	Reuse
chicken:100K	7	taeniopygia_guttata	4,500,197	4,500,975	Unique
chicken:100K	7	manacus_vitellinus	4,739,224	4,748,519	Unique
chicken:100K	7	pygoscelis_adeliae	4,885,804	4,889,908	Unique
chicken:100K	7	columba_livia	5,334,400	5,334,885	Unique
chicken:100K	7	manacus_vitellinus:cuculus_canorus	5,540,284	5,542,374	Reuse
chicken:100K	7	cuculus_canorus:manacus_vitellinus	5,540,284	5,542,374	Reuse
chicken:100K	7	anas_platyrhynchos	5,561,037	5,565,900	Unique
chicken:100K	7	cuculus_canorus	5,941,257	5,955,407	Unique
chicken:100K	7	chrysemys_picta	6,089,300	6,117,895	Unique
chicken:100K	7	galliformes	6,895,405	6,897,471	Unique
chicken:100K	7	chicken	7,336,101	7,339,689	Unique
chicken:100K	7	picoides_pubescens	7,608,142	7,618,019	Unique
chicken:100K	7	aptenodytes_forsteri	8,788,746	8,793,058	Unique
chicken:100K	7	calypte_anna:anolis_carolinensis	8,848,669	8,852,184	Reuse
chicken:100K	7	anolis_carolinensis:calypte_anna	8,848,669	8,852,184	Reuse
chicken:100K	7	calypte_anna	8,988,832	8,994,642	Unique
chicken:100K	7	chrysemys_picta	9,081,644	9,115,515	Unique
chicken:100K	7	columba_livia	9,275,107	9,281,206	Unique
chicken:100K	7	picoides_pubescens	9,513,620	9,526,053	Unique
chicken:100K	7	galliformes	9,812,874	9,813,453	Unique
chicken:100K	7	chinese_alligator	10,076,038	10,087,726	Unique
chicken:100K	7	anas_platyrhynchos	10,268,890	10,270,242	Unique
chicken:100K	7	chrysemys_picta	10,329,157	10,333,823	Unique
chicken:100K	7	picoides_pubescens	10,773,307	10,778,768	Unique
chicken:100K	7	falco_peregrinus:calypte_anna	10,953,137	10,958,987	Reuse
chicken:100K	7	calypte_anna:falco_peregrinus	10,953,137	10,958,987	Reuse
chicken:100K	7	anas_platyrhynchos	11,000,835	11,003,302	Unique
chicken:100K	7	calypte_anna	11,089,455	11,095,279	Unique
chicken:100K	7	columba_livia	11,465,499	11,473,934	Unique
chicken:100K	7	birds	11,514,164	11,525,238	Unique
chicken:100K	7	calypte_anna	12,032,684	12,035,702	Unique
chicken:100K	7	calypte_anna	12,802,524	12,852,167	Unique
chicken:100K	7	struthio_camelus	13,376,264	13,377,564	Unique
chicken:100K	7	columba_livia	13,394,943	13,403,466	Unique
chicken:100K	7	aptenodytes_forsteri	13,832,970	13,838,513	Unique
chicken:100K	7	anas_platyrhynchos	14,086,550	14,087,974	Unique
chicken:100K	7	boa_constrictor	15,382,491	15,399,827	Unique
chicken:100K	7	chinese_alligator	16,675,493	16,684,252	Unique
chicken:100K	7	chinese_alligator	17,071,211	17,083,971	Unique
chicken:100K	7	anolis_carolinensis	17,482,103	17,508,920	Unique
chicken:100K	7	meleagris_gallopavo	17,590,714	17,647,529	Unique
chicken:100K	7	meleagris_gallopavo:anolis_carolinensis	17,707,240	17,708,440	Reuse
chicken:100K	7	anolis_carolinensis:meleagris_gallopavo	17,707,240	17,708,440	Reuse
chicken:100K	7	chinese_alligator	19,228,443	19,334,980	Unique
chicken:100K	7	chinese_alligator	19,540,591	19,715,877	Unique
chicken:100K	7	anolis_carolinensis	20,093,862	20,103,747	Unique
chicken:100K	7	anolis_carolinensis	20,371,545	20,396,084	Unique
chicken:100K	7	melopsittacus_undulatus	21,369,589	21,372,225	Unique
chicken:100K	7	birds	21,419,119	21,428,132	Unique
chicken:100K	7	anas_platyrhynchos	21,708,370	21,710,524	Unique
chicken:100K	7	boa_constrictor	21,735,202	21,739,895	Unique
chicken:100K	7	anolis_carolinensis	21,942,643	21,948,734	Unique

chicken:100K	7	cuculus_canorus	22,059,168	22,063,546	Unique
chicken:100K	7	nipponia_nippon:anolis_carolinensis	22,753,402	22,756,738	Reuse
chicken:100K	7	anolis_carolinensis:nipponia_nippon	22,753,402	22,756,738	Reuse
chicken:100K	7	anolis_carolinensis	23,576,628	23,596,428	Unique
chicken:100K	7	anolis_carolinensis	26,139,183	26,142,096	Unique
chicken:100K	7	taeniopygia_guttata	27,786,737	27,787,078	Unique
chicken:100K	7	chrysemys_picta	27,914,687	27,927,763	Unique
chicken:100K	7	chrysemys_picta	28,528,179	28,541,883	Unique
chicken:100K	7	chrysemys_picta	28,729,777	28,737,932	Unique
chicken:100K	7	struthio_camelus	29,155,877	29,161,171	Unique
chicken:100K	7	anolis_carolinensis	29,205,132	29,208,212	Unique
chicken:100K	7	anolis_carolinensis	29,709,927	29,765,425	Unique
chicken:100K	7	chrysemys_picta	30,080,452	30,081,723	Unique
chicken:100K	7	chrysemys_picta	30,584,850	30,592,770	Unique
chicken:100K	7	picoides_pubescens	30,722,936	30,726,898	Unique
chicken:100K	7	anolis_carolinensis	33,294,489	33,304,904	Unique
chicken:100K	7	chrysemys_picta	33,391,485	33,397,087	Unique
chicken:100K	7	meleagris_gallopavo	33,547,558	33,550,795	Unique
chicken:100K	7	meleagris_gallopavo	34,739,968	34,750,782	Unique
chicken:100K	7	chrysemys_picta	35,080,690	35,287,415	Unique
chicken:100K	7	meleagris_gallopavo	35,533,125	35,544,927	Unique
chicken:100K	8	meleagris_gallopavo	419,399	424,683	Unique
chicken:100K	8	anolis_carolinensis	1,107,900	1,172,406	Unique
chicken:100K	8	ophisthocomus_hoazin	1,251,591	1,253,849	Unique
chicken:100K	8	meleagris_gallopavo	1,280,561	1,281,898	Unique
chicken:100K	8	falco_peregrinus	1,300,251	1,303,401	Unique
chicken:100K	8	egretta_garzetta	1,337,573	1,339,293	Unique
chicken:100K	8	melopsittacus_undulatus	1,704,944	1,706,600	Unique
chicken:100K	8	falco_peregrinus	2,637,724	2,641,020	Unique
chicken:100K	8	spheniscidae	2,851,682	2,851,984	Unique
chicken:100K	8	melopsittacus_undulatus	3,619,749	3,628,508	Unique
chicken:100K	8	struthio_camelus	3,968,737	3,981,901	Unique
chicken:100K	8	cuculus_canorus	4,286,765	4,296,557	Unique
chicken:100K	8	picoides_pubescens	4,797,639	4,817,837	Unique
chicken:100K	8	columba_livia	5,071,736	5,073,539	Unique
chicken:100K	8	chinese_alligator	5,551,036	5,561,008	Unique
chicken:100K	8	melopsittacus_undulatus	6,050,534	6,058,098	Unique
chicken:100K	8	calypte_anna	6,062,393	6,067,710	Unique
chicken:100K	8	anolis_carolinensis	6,082,835	6,116,739	Unique
chicken:100K	8	anolis_carolinensis	6,606,968	6,609,310	Unique
chicken:100K	8	falco_peregrinus	6,979,710	7,023,495	Unique
chicken:100K	8	chaetura_pelagica	7,059,111	7,060,255	Unique
chicken:100K	8	birds	7,400,330	7,409,400	Unique
chicken:100K	8	falco_peregrinus:anas_platyrhynchos	7,609,130	7,612,117	Reuse
chicken:100K	8	anas_platyrhynchos:falco_peregrinus	7,609,130	7,612,117	Reuse
chicken:100K	8	columba_livia	7,706,183	7,709,483	Unique
chicken:100K	8	chaetura_pelagica	7,753,472	7,759,223	Unique
chicken:100K	8	melopsittacus_undulatus	9,620,987	9,623,773	Unique
chicken:100K	8	chicken	9,981,443	9,997,875	Unique
chicken:100K	8	chrysemys_picta	10,449,755	10,459,160	Unique
chicken:100K	8	ophisthocomus_hoazin	10,593,359	10,597,301	Unique
chicken:100K	8	cuculus_canorus	10,597,710	10,601,550	Unique
chicken:100K	8	picoides_pubescens	10,875,162	10,879,300	Unique
chicken:100K	8	melopsittacus_undulatus	11,136,138	11,138,691	Unique
chicken:100K	8	chrysemys_picta	11,638,060	11,675,878	Unique
chicken:100K	8	meleagris_gallopavo	11,885,274	11,887,182	Unique
chicken:100K	8	melopsittacus_undulatus	12,486,680	12,489,487	Unique
chicken:100K	8	melopsittacus_undulatus	12,729,848	12,737,159	Unique
chicken:100K	8	cuculus_canorus	12,744,631	12,746,639	Unique
chicken:100K	8	struthio_camelus	12,809,192	12,811,568	Unique
chicken:100K	8	melopsittacus_undulatus:anas_platyrhynchos	13,898,955	13,900,642	Reuse
chicken:100K	8	anas_platyrhynchos:melopsittacus_undulatus	13,898,955	13,900,642	Reuse
chicken:100K	8	struthio_camelus	14,545,573	14,549,643	Unique
chicken:100K	8	chrysemys_picta	14,887,099	14,900,884	Unique
chicken:100K	8	melopsittacus_undulatus:chrysemys_picta	15,103,103	15,107,360	Reuse
chicken:100K	8	chrysemys_picta:melopsittacus_undulatus	15,103,103	15,107,360	Reuse

chicken:100K	8	melopsittacus_undulatus	15,279,967	15,290,565	Unique
chicken:100K	8	manacus_vitellinus	15,376,281	15,386,615	Unique
chicken:100K	8	chrysemys_picta	15,625,081	15,667,023	Unique
chicken:100K	8	struthio_camelus	15,782,263	15,786,273	Unique
chicken:100K	8	chrysemys_picta	15,977,562	15,986,709	Unique
chicken:100K	8	corvus_brachyrhynchos	16,578,881	16,580,645	Unique
chicken:100K	8	struthio_camelus	17,503,033	17,505,016	Unique
chicken:100K	8	columba_livia	18,506,022	18,506,040	Unique
chicken:100K	8	picoides_pubescens	18,548,061	18,554,640	Unique
chicken:100K	8	anolis_carolinensis	19,692,669	19,698,245	Unique
chicken:100K	8	boa_constrictor	19,918,668	19,930,887	Unique
chicken:100K	8	chrysemys_picta	20,496,234	20,541,208	Unique
chicken:100K	8	columba_livia	20,586,001	20,588,474	Unique
chicken:100K	8	anolis_carolinensis	20,715,723	20,746,966	Unique
chicken:100K	8	geospiza_fortis	21,490,647	21,491,764	Unique
chicken:100K	8	pygoscelis_adeliae	21,628,314	21,631,240	Unique
chicken:100K	8	meleagris_gallopavo	21,857,413	21,861,831	Unique
chicken:100K	8	geospiza_fortis	21,886,161	21,891,971	Unique
chicken:100K	8	meleagris_gallopavo	22,036,665	22,042,283	Unique
chicken:100K	8	anolis_carolinensis	22,181,314	22,238,521	Unique
chicken:100K	8	picoides_pubescens	22,872,510	22,884,898	Unique
chicken:100K	8	columba_livia	23,002,549	23,005,948	Unique
chicken:100K	8	chrysemys_picta	23,113,815	23,114,945	Unique
chicken:100K	8	corvus_brachyrhynchos	23,290,564	23,292,328	Unique
chicken:100K	8	pygoscelis_adeliae	23,768,033	23,780,360	Unique
chicken:100K	8	anolis_carolinensis	24,009,013	24,023,724	Unique
chicken:100K	8	chrysemys_picta	24,288,232	24,296,277	Unique
chicken:100K	8	chrysemys_picta	25,569,632	25,598,820	Unique
chicken:100K	8	chrysemys_picta	26,810,563	26,825,266	Unique
chicken:100K	8	anolis_carolinensis	27,305,689	27,516,549	Unique
chicken:100K	8	picoides_pubescens	27,562,241	27,570,586	Unique
chicken:100K	8	passeroidea + corvoidea	27,692,998	27,702,399	Unique
chicken:100K	8	chrysemys_picta	27,715,794	27,741,107	Unique
chicken:100K	8	picoides_pubescens	27,904,174	27,914,293	Unique
chicken:100K	9	calypte_anna	190,363	194,651	Unique
chicken:100K	9	melopsittacus_undulatus:calypte_anna	803,621	805,103	Reuse
chicken:100K	9	calypte_anna:melopsittacus_undulatus	803,621	805,103	Reuse
chicken:100K	9	passeroidea + corvoidea	905,234	911,027	Unique
chicken:100K	9	Trochiliformes + Apodiformes	1,054,267	1,058,851	Unique
chicken:100K	9	anas_platyrhynchos	1,714,064	1,731,126	Unique
chicken:100K	9	calypte_anna	1,789,034	1,798,054	Unique
chicken:100K	9	picoides_pubescens	2,002,526	2,011,901	Unique
chicken:100K	9	pygoscelis_adeliae	2,147,048	2,148,659	Unique
chicken:100K	9	boa_constrictor	2,200,632	2,202,632	Unique
chicken:100K	9	pygoscelis_adeliae	2,292,117	2,294,337	Unique
chicken:100K	9	chicken	2,892,620	2,958,453	Unique
chicken:100K	9	cuculus_canorus	3,082,238	3,087,866	Unique
chicken:100K	9	chinese_alligator:boa_constrictor	3,164,343	3,167,621	Reuse
chicken:100K	9	boa_constrictor:chinese_alligator	3,164,343	3,167,621	Reuse
chicken:100K	9	anolis_carolinensis	3,228,395	3,241,302	Unique
chicken:100K	9	struthio_camelus	3,893,908	3,896,629	Unique
chicken:100K	9	melopsittacus_undulatus	4,181,015	4,187,137	Unique
chicken:100K	9	galloanserae	4,322,998	4,323,141	Unique
chicken:100K	9	galliformes	4,713,046	4,717,342	Unique
chicken:100K	9	anolis_carolinensis	4,730,628	4,744,566	Unique
chicken:100K	9	cuculus_canorus	4,970,243	4,978,867	Unique
chicken:100K	9	chaetura_pelagica	5,588,899	5,599,095	Unique
chicken:100K	9	anolis_carolinensis	5,916,909	5,979,705	Unique
chicken:100K	9	melopsittacus_undulatus:columba_livia	6,289,875	6,373,739	Reuse
chicken:100K	9	columba_livia:melopsittacus_undulatus	6,289,875	6,373,739	Reuse
chicken:100K	9	melopsittacus_undulatus	6,510,631	6,511,923	Unique
chicken:100K	9	anolis_carolinensis	7,037,589	7,060,700	Unique
chicken:100K	9	aptenodytes_forsteri	7,144,984	7,146,580	Unique
chicken:100K	9	aptenodytes_forsteri	7,595,227	7,598,513	Unique
chicken:100K	9	nipponia_nippon	8,372,133	8,374,940	Unique
chicken:100K	9	calypte_anna	8,423,705	8,425,709	Unique

chicken:100K	9	melopsittacus_undulatus:calypte_anna	8,751,134	8,754,782	Reuse
chicken:100K	9	calypte_anna:melopsittacus_undulatus	8,751,134	8,754,782	Reuse
chicken:100K	9	picoides_pubescens	9,034,844	9,077,937	Unique
chicken:100K	9	calypte_anna	9,248,220	9,259,023	Unique
chicken:100K	9	struthio_camelus:picoides_pubescens	9,516,293	9,519,032	Reuse
chicken:100K	9	picoides_pubescens:struthio_camelus	9,516,293	9,519,032	Reuse
chicken:100K	9	struthio_camelus	9,657,033	9,659,195	Unique
chicken:100K	9	picoides_pubescens	10,035,640	10,040,961	Unique
chicken:100K	9	nipponia_nippon	10,207,876	10,211,038	Unique
chicken:100K	9	meleagris_gallopavo	10,358,255	10,359,913	Unique
chicken:100K	9	meleagris_gallopavo	10,494,202	10,504,281	Unique
chicken:100K	9	meleagris_gallopavo:anolis_carolinensis	10,650,254	10,875,246	Reuse
chicken:100K	9	anolis_carolinensis:meleagris_gallopavo	10,650,254	10,875,246	Reuse
chicken:100K	9	anolis_carolinensis	11,342,619	11,353,663	Unique
chicken:100K	9	anas_platyrhynchos	11,939,732	11,941,765	Unique
chicken:100K	9	columba_livia	12,390,618	12,397,138	Unique
chicken:100K	9	chrysemys_picta	12,417,617	12,421,639	Unique
chicken:100K	9	melopsittacus_undulatus	12,715,948	12,715,985	Unique
chicken:100K	9	melopsittacus_undulatus	13,441,937	13,445,979	Unique
chicken:100K	9	opossum:egretta_garzetta	14,734,979	14,742,449	Reuse
chicken:100K	9	egretta_garzetta:opossum	14,734,979	14,742,449	Reuse
chicken:100K	9	chrysemys_picta	15,102,233	15,125,934	Unique
chicken:100K	9	columba_livia	15,160,944	15,165,067	Unique
chicken:100K	9	meleagris_gallopavo	18,477,569	18,483,278	Unique
chicken:100K	9	meleagris_gallopavo	18,658,748	18,665,606	Unique
chicken:100K	9	aptenodytes_forsteri	18,887,314	18,890,092	Unique
chicken:100K	9	columba_livia	19,340,917	19,343,880	Unique
chicken:100K	9	picoides_pubescens	20,216,705	20,218,444	Unique
chicken:100K	9	melopsittacus_undulatus	20,310,029	20,311,879	Unique
chicken:100K	9	melopsittacus_undulatus	20,540,840	20,542,557	Unique
chicken:100K	9	falco_peregrinus	22,750,595	22,801,936	Unique
chicken:100K	9	chrysemys_picta	23,101,182	23,109,479	Unique
chicken:100K	10	calypte_anna	802,397	806,827	Unique
chicken:100K	10	cuculus_canorus:chinese_alligator	871,599	898,295	Reuse
chicken:100K	10	chinese_alligator:cuculus_canorus	871,599	898,295	Reuse
chicken:100K	10	boa_constrictor:anolis_carolinensis	1,100,881	1,116,975	Reuse
chicken:100K	10	anolis_carolinensis:boa_constrictor	1,100,881	1,116,975	Reuse
chicken:100K	10	galloanserae	1,824,471	1,838,259	Unique
chicken:100K	10	columba_livia	1,992,992	1,996,840	Unique
chicken:100K	10	anas_platyrhynchos	2,035,057	2,050,944	Unique
chicken:100K	10	boa_constrictor	2,154,396	2,156,924	Unique
chicken:100K	10	opossum:anas_platyrhynchos	2,206,963	2,213,274	Reuse
chicken:100K	10	anas_platyrhynchos:opossum	2,206,963	2,213,274	Reuse
chicken:100K	10	falco_peregrinus	2,454,559	2,455,512	Unique
chicken:100K	10	struthio_camelus	2,479,255	2,482,284	Unique
chicken:100K	10	columba_livia:calypte_anna	3,528,267	3,535,513	Reuse
chicken:100K	10	calypte_anna:columba_livia	3,528,267	3,535,513	Reuse
chicken:100K	10	passeroidea	3,606,688	3,614,022	Unique
chicken:100K	10	columba_livia	4,539,435	4,542,050	Unique
chicken:100K	10	picoides_pubescens	4,719,159	4,723,126	Unique
chicken:100K	10	geospiza_fortis	5,130,398	5,131,698	Unique
chicken:100K	10	melopsittacus_undulatus	6,299,680	6,302,508	Unique
chicken:100K	10	cuculus_canorus	6,362,183	6,373,897	Unique
chicken:100K	10	spheniscidae	7,154,861	7,159,073	Unique
chicken:100K	10	corvus_brachyrhynchos:columba_livia	7,420,438	7,420,666	Reuse
chicken:100K	10	columba_livia:corvus_brachyrhynchos	7,420,438	7,420,666	Reuse
chicken:100K	10	cuculus_canorus	7,523,168	7,526,342	Unique
chicken:100K	10	calypte_anna	7,535,601	7,538,411	Unique
chicken:100K	10	chrysemys_picta	7,560,856	7,607,998	Unique
chicken:100K	10	melopsittacus_undulatus	9,634,831	9,640,376	Unique
chicken:100K	10	chrysemys_picta	10,114,861	10,126,178	Unique
chicken:100K	10	anas_platyrhynchos	10,224,248	10,225,564	Unique
chicken:100K	10	picoides_pubescens	11,164,464	11,164,870	Unique
chicken:100K	10	cuculus_canorus	11,261,853	11,263,604	Unique
chicken:100K	10	boa_constrictor	14,416,785	14,476,025	Unique
chicken:100K	10	pygoscelis_adeliae	15,569,181	15,571,720	Unique

chicken:100K	10	aptenodytes_forsteri	16,099,850	16,101,191	Unique
chicken:100K	10	boa_constrictor	16,376,918	16,403,220	Unique
chicken:100K	10	picoides_pubescens	17,149,686	17,157,759	Unique
chicken:100K	10	columba_livia	17,204,679	17,205,848	Unique
chicken:100K	10	boa_constrictor	17,512,766	17,520,124	Unique
chicken:100K	10	chrysemys_picta	18,068,255	18,070,116	Unique
chicken:100K	10	meleagris_gallopavo	19,011,590	19,036,315	Unique
chicken:100K	10	picoides_pubescens	19,256,119	19,263,227	Unique
chicken:100K	10	picoides_pubescens:meleagris_gallopavo	19,385,837	19,560,687	Reuse
chicken:100K	10	meleagris_gallopavo:picoides_pubescens	19,385,837	19,560,687	Reuse
chicken:100K	10	meleagris_gallopavo	19,738,455	19,740,357	Unique
chicken:100K	11	galliformes	338,797	348,453	Unique
chicken:100K	11	passeroidea + corvoidea	759,313	761,173	Unique
chicken:100K	11	anas_platyrhynchos	762,806	777,722	Unique
chicken:100K	11	falco_peregrinus	847,165	851,802	Unique
chicken:100K	11	melopsittacus_undulatus	852,864	862,092	Unique
chicken:100K	11	passeroidea + corvoidea	880,360	885,539	Unique
chicken:100K	11	melopsittacus_undulatus	1,456,402	1,461,750	Unique
chicken:100K	11	ophisthocomus_hoazin:falco_peregrinus:cuculus_canorus	1,638,585	1,639,678	Reuse
chicken:100K	11	falco_peregrinus:cuculus_canorus:ophisthocomus_hoazin	1,638,585	1,639,678	Reuse
chicken:100K	11	cuculus_canorus:ophisthocomus_hoazin:falco_peregrinus	1,638,585	1,639,678	Reuse
chicken:100K	11	nipponia_nippon	1,915,188	1,918,551	Unique
chicken:100K	11	galloanserae	2,314,757	2,316,921	Unique
chicken:100K	11	chicken	2,655,301	2,701,594	Unique
chicken:100K	11	ophisthocomus_hoazin	2,905,239	2,908,838	Unique
chicken:100K	11	birds_crocs_turtles	3,013,079	3,018,298	Unique
chicken:100K	11	columba_livia	3,160,639	3,163,523	Unique
chicken:100K	11	pygoscelis_adeliae	5,711,060	5,714,402	Unique
chicken:100K	11	taeniopygia_guttata	5,982,285	5,991,052	Unique
chicken:100K	11	anolis_carolinensis	6,784,532	6,859,319	Unique
chicken:100K	11	melopsittacus_undulatus:cuculus_canorus	6,912,222	6,922,567	Reuse
chicken:100K	11	cuculus_canorus:melopsittacus_undulatus	6,912,222	6,922,567	Reuse
chicken:100K	11	manacus_vitellinus	7,217,517	7,218,272	Unique
chicken:100K	11	chinese_alligator	7,271,195	7,414,232	Unique
chicken:100K	11	melopsittacus_undulatus	7,847,347	7,847,951	Unique
chicken:100K	11	picoides_pubescens:charadrius_vociferus:ophisthocomus_hoazin	9,333,607	9,339,798	Reuse
chicken:100K	11	ophisthocomus_hoazin:picoides_pubescens:charadrius_vociferus	9,333,607	9,339,798	Reuse
chicken:100K	11	charadrius_vociferus:ophisthocomus_hoazin:picoides_pubescens	9,333,607	9,339,798	Reuse
chicken:100K	11	anas_platyrhynchos	9,423,866	9,426,047	Unique
chicken:100K	11	egretta_garzetta	9,470,672	9,471,134	Unique
chicken:100K	11	boa_constrictor	10,374,800	10,377,100	Unique
chicken:100K	11	ophisthocomus_hoazin	10,766,395	10,773,399	Unique
chicken:100K	11	cuculus_canorus	10,782,151	10,789,126	Unique
chicken:100K	11	picoides_pubescens	10,902,869	10,906,745	Unique
chicken:100K	11	ophisthocomus_hoazin	11,050,743	11,053,927	Unique
chicken:100K	11	taeniopygia_guttata:anas_platyrhynchos	11,966,948	11,967,127	Reuse
chicken:100K	11	anas_platyrhynchos:taeniopygia_guttata	11,966,948	11,967,127	Reuse
chicken:100K	11	chrysemys_picta	12,174,503	12,177,375	Unique
chicken:100K	11	anolis_carolinensis	12,404,420	12,429,749	Unique
chicken:100K	11	passeroidea + corvoidea	13,424,369	13,515,770	Unique
chicken:100K	11	pygoscelis_adeliae	13,579,131	13,581,776	Unique
chicken:100K	11	anolis_carolinensis	13,986,639	14,000,710	Unique
chicken:100K	11	columba_livia	14,586,082	14,586,231	Unique
chicken:100K	11	picoides_pubescens	14,855,478	14,860,828	Unique
chicken:100K	11	ophisthocomus_hoazin	16,239,001	16,239,773	Unique
chicken:100K	11	taeniopygia_guttata	17,098,255	17,102,722	Unique
chicken:100K	11	melopsittacus_undulatus	17,903,320	17,920,718	Unique
chicken:100K	11	cuculus_canorus	18,308,117	18,310,349	Unique
chicken:100K	11	chrysemys_picta	18,660,496	18,667,801	Unique
chicken:100K	11	chaetura_pelagica	18,893,883	18,896,674	Unique
chicken:100K	12	meleagris_gallopavo	371,657	375,235	Unique
chicken:100K	12	struthio_camelus	664,445	668,816	Unique
chicken:100K	12	picoides_pubescens:meleagris_gallopavo	1,009,849	1,122,939	Reuse
chicken:100K	12	meleagris_gallopavo:picoides_pubescens	1,009,849	1,122,939	Reuse
chicken:100K	12	taeniopygia_guttata	1,197,274	1,200,040	Unique
chicken:100K	12	falco_peregrinus	1,349,567	1,353,798	Unique

chicken:100K	12	chinese_alligator	1,578,054	1,581,340	Unique
chicken:100K	12	struthio_camelus:chrysemys_picta	1,705,129	1,707,655	Reuse
chicken:100K	12	chrysemys_picta:struthio_camelus	1,705,129	1,707,655	Reuse
chicken:100K	12	meleagris_gallopavo	1,820,901	1,824,531	Unique
chicken:100K	12	chinese_alligator	1,883,402	1,944,611	Unique
chicken:100K	12	falco_peregrinus	2,057,477	2,062,823	Unique
chicken:100K	12	columba_livia	2,486,857	2,488,604	Unique
chicken:100K	12	chicken	2,729,314	2,747,184	Unique
chicken:100K	12	meleagris_gallopavo	2,894,320	2,905,602	Unique
chicken:100K	12	galliformes	2,918,037	2,922,092	Unique
chicken:100K	12	galliformes	3,233,766	3,238,558	Unique
chicken:100K	12	melopsittacus_undulatus	3,465,725	3,465,867	Unique
chicken:100K	12	nipponia_nippon	3,467,109	3,469,104	Unique
chicken:100K	12	manacus_vitellinus:cuculus_canorus	4,343,399	4,350,745	Reuse
chicken:100K	12	cuculus_canorus:manacus_vitellinus	4,343,399	4,350,745	Reuse
chicken:100K	12	falco_peregrinus:columba_livia	4,368,564	4,375,410	Reuse
chicken:100K	12	columba_livia:falco_peregrinus	4,368,564	4,375,410	Reuse
chicken:100K	12	picoides_pubescens	5,121,280	5,207,208	Unique
chicken:100K	12	anolis_carolinensis	5,555,508	5,709,052	Unique
chicken:100K	12	egretta_garzetta	5,735,062	5,735,649	Unique
chicken:100K	12	picoides_pubescens:calypte_anna	5,882,626	5,883,382	Reuse
chicken:100K	12	calypte_anna:picoides_pubescens	5,882,626	5,883,382	Reuse
chicken:100K	12	egretta_garzetta	6,088,555	6,092,300	Unique
chicken:100K	12	columba_livia	6,368,382	6,375,173	Unique
chicken:100K	12	nipponia_nippon	7,237,069	7,238,911	Unique
chicken:100K	12	columba_livia	8,361,157	8,367,304	Unique
chicken:100K	12	opossum:chrysemys_picta:anas_platyrhynchos	8,586,196	8,737,555	Reuse
chicken:100K	12	chrysemys_picta:anas_platyrhynchos:opossum	8,586,196	8,737,555	Reuse
chicken:100K	12	anas_platyrhynchos:opossum:chrysemys_picta	8,586,196	8,737,555	Reuse
chicken:100K	12	struthio_camelus	9,086,320	9,092,642	Unique
chicken:100K	12	melopsittacus_undulatus	9,115,755	9,123,046	Unique
chicken:100K	12	picoides_pubescens	11,227,412	11,231,272	Unique
chicken:100K	12	birds	11,244,729	11,253,382	Unique
chicken:100K	12	melopsittacus_undulatus	11,624,885	11,632,490	Unique
chicken:100K	12	cuculus_canorus	11,828,105	11,830,688	Unique
chicken:100K	12	melopsittacus_undulatus:chaetura_pelagica	11,935,462	11,935,510	Reuse
chicken:100K	12	chaetura_pelagica:melopsittacus_undulatus	11,935,462	11,935,510	Reuse
chicken:100K	12	chrysemys_picta	11,979,190	11,987,050	Unique
chicken:100K	12	anolis_carolinensis	12,575,129	12,581,558	Unique
chicken:100K	12	struthio_camelus	12,935,614	12,936,217	Unique
chicken:100K	12	melopsittacus_undulatus	13,605,943	13,607,116	Unique
chicken:100K	12	pygoscelis_adeliae:chinese_alligator	14,172,195	14,175,795	Reuse
chicken:100K	12	chinese_alligator:pygoscelis_adeliae	14,172,195	14,175,795	Reuse
chicken:100K	12	chaetura_pelagica:anolis_carolinensis	14,330,677	14,336,569	Reuse
chicken:100K	12	anolis_carolinensis:chaetura_pelagica	14,330,677	14,336,569	Reuse
chicken:100K	12	struthio_camelus	15,305,398	15,310,490	Unique
chicken:100K	12	aptenodytes_forsteri	16,159,856	16,160,615	Unique
chicken:100K	12	picoides_pubescens	16,751,878	16,755,561	Unique
chicken:100K	12	chrysemys_picta	17,438,096	17,439,740	Unique
chicken:100K	12	columba_livia	18,620,394	18,626,803	Unique
chicken:100K	12	ophisthocomus_hoazin	19,081,623	19,085,468	Unique
chicken:100K	12	melopsittacus_undulatus	19,251,300	19,254,763	Unique
chicken:100K	12	anolis_carolinensis	19,675,860	19,677,454	Unique
chicken:100K	13	anolis_carolinensis:anas_platyrhynchos	263,863	301,076	Reuse
chicken:100K	13	anas_platyrhynchos:anolis_carolinensis	263,863	301,076	Reuse
chicken:100K	13	cuculus_canorus	383,304	383,519	Unique
chicken:100K	13	corvus_brachyrhynchos	807,462	813,058	Unique
chicken:100K	13	meleagris_gallopavo	1,211,023	1,214,459	Unique
chicken:100K	13	meleagris_gallopavo	1,345,724	1,350,080	Unique
chicken:100K	13	melopsittacus_undulatus	1,390,992	1,398,605	Unique
chicken:100K	13	calypte_anna	1,962,786	1,963,317	Unique
chicken:100K	13	columba_livia	2,655,460	2,658,550	Unique
chicken:100K	13	boa_constrictor	2,826,161	2,865,236	Unique
chicken:100K	13	pygoscelis_adeliae:calypte_anna	3,497,210	3,583,875	Reuse
chicken:100K	13	calypte_anna:pygoscelis_adeliae	3,497,210	3,583,875	Reuse
chicken:100K	13	columba_livia:calypte_anna	4,070,726	4,095,041	Reuse

chicken:100K	13	calypte_anna:columba_livia	4,070,726	4,095,041	Reuse
chicken:100K	13	chrysemys_picta	4,479,331	4,492,085	Unique
chicken:100K	13	picoides_pubescens:melopsittacus_undulatus	6,042,883	6,046,713	Reuse
chicken:100K	13	melopsittacus_undulatus:picoides_pubescens	6,042,883	6,046,713	Reuse
chicken:100K	13	manacus_vitellinus	6,082,360	6,087,114	Unique
chicken:100K	13	anolis_carolinensis	6,828,576	6,868,941	Unique
chicken:100K	13	passeroidea + corvoidea	7,156,929	7,174,015	Unique
chicken:100K	13	columba_livia	7,516,337	7,519,931	Unique
chicken:100K	13	struthio_camelus	7,767,021	7,768,547	Unique
chicken:100K	13	anas_platyrhynchos	7,920,338	7,922,917	Unique
chicken:100K	13	opossum:falco_peregrinus	7,941,525	7,979,004	Reuse
chicken:100K	13	falco_peregrinus:opossum	7,941,525	7,979,004	Reuse
chicken:100K	13	chinese_alligator:anolis_carolinensis	8,632,070	8,636,757	Reuse
chicken:100K	13	anolis_carolinensis:chinese_alligator	8,632,070	8,636,757	Reuse
chicken:100K	13	struthio_camelus:anas_platyrhynchos	9,472,507	9,482,291	Reuse
chicken:100K	13	anas_platyrhynchos:struthio_camelus	9,472,507	9,482,291	Reuse
chicken:100K	13	falco_peregrinus	9,542,096	9,594,522	Unique
chicken:100K	13	struthio_camelus:anas_platyrhynchos	9,669,865	9,672,898	Reuse
chicken:100K	13	anas_platyrhynchos:struthio_camelus	9,669,865	9,672,898	Reuse
chicken:100K	13	calypte_anna	10,588,216	10,645,619	Unique
chicken:100K	13	melopsittacus_undulatus	10,816,294	10,817,247	Unique
chicken:100K	13	meleagris_gallopavo	10,949,615	10,953,489	Unique
chicken:100K	13	chrysemys_picta	11,960,636	11,971,043	Unique
chicken:100K	13	anolis_carolinensis	12,328,743	12,343,054	Unique
chicken:100K	13	struthio_camelus	12,462,337	12,467,832	Unique
chicken:100K	13	opossum:meleagris_gallopavo	12,650,896	12,677,916	Reuse
chicken:100K	13	meleagris_gallopavo:opossum	12,650,896	12,677,916	Reuse
chicken:100K	13	calypte_anna	13,453,567	13,456,869	Unique
chicken:100K	13	anolis_carolinensis	13,854,417	14,197,911	Unique
chicken:100K	13	manacus_vitellinus	14,986,619	14,992,119	Unique
chicken:100K	13	anolis_carolinensis	15,098,457	15,106,937	Unique
chicken:100K	13	chrysemys_picta	15,330,416	15,337,387	Unique
chicken:100K	13	manacus_vitellinus	15,365,684	15,369,484	Unique
chicken:100K	13	picoides_pubescens	15,373,900	15,386,157	Unique
chicken:100K	13	egretta_garzetta	15,865,413	15,866,065	Unique
chicken:100K	13	cuculus_canorus	15,966,691	15,973,874	Unique
chicken:100K	13	columba_livia	16,162,870	16,166,111	Unique
chicken:100K	13	pygoscelis_adeliae	16,331,160	16,335,126	Unique
chicken:100K	13	cuculus_canorus	16,546,835	16,553,197	Unique
chicken:100K	13	falco_peregrinus	16,561,057	16,574,270	Unique
chicken:100K	13	anolis_carolinensis	16,905,043	16,982,463	Unique
chicken:100K	13	chrysemys_picta	17,065,754	17,071,918	Unique
chicken:100K	13	columba_livia	17,498,360	17,504,128	Unique
chicken:100K	13	boa_constrictor	17,529,690	17,544,924	Unique
chicken:100K	13	struthio_camelus	17,575,355	17,578,428	Unique
chicken:100K	14	falco_peregrinus:anas_platyrhynchos	649,245	666,465	Reuse
chicken:100K	14	anas_platyrhynchos:falco_peregrinus	649,245	666,465	Reuse
chicken:100K	14	nipponia_nippon	755,888	759,970	Unique
chicken:100K	14	galliformes	937,476	938,425	Unique
chicken:100K	14	egretta_garzetta	1,641,570	1,643,681	Unique
chicken:100K	14	ophisthocomus_hoazin	2,488,620	2,491,297	Unique
chicken:100K	14	birds_crocs_turtles	4,654,653	4,660,836	Unique
chicken:100K	14	struthio_camelus:opossum	5,143,295	5,147,591	Reuse
chicken:100K	14	opossum:struthio_camelus	5,143,295	5,147,591	Reuse
chicken:100K	14	anolis_carolinensis	5,912,054	5,971,025	Unique
chicken:100K	14	columba_livia	6,204,122	6,209,403	Unique
chicken:100K	14	picoides_pubescens:opossum	6,493,509	6,499,276	Reuse
chicken:100K	14	opossum:picoides_pubescens	6,493,509	6,499,276	Reuse
chicken:100K	14	picoides_pubescens	7,275,190	7,280,143	Unique
chicken:100K	14	galliformes	7,458,779	7,459,817	Unique
chicken:100K	14	ophisthocomus_hoazin	7,728,668	7,737,528	Unique
chicken:100K	14	taeniopygia_guttata	8,319,574	8,323,624	Unique
chicken:100K	14	galliformes	8,541,115	8,541,575	Unique
chicken:100K	14	columba_livia	8,710,117	8,723,732	Unique
chicken:100K	14	anas_platyrhynchos	8,860,158	8,888,316	Unique
chicken:100K	14	galloanserae	8,900,871	8,901,116	Unique

chicken:100K	14	cuculus_canorus	9,056,883	9,059,601	Unique
chicken:100K	14	cuculus_canorus	9,223,401	9,250,484	Unique
chicken:100K	14	boa_constrictor	9,806,105	9,822,187	Unique
chicken:100K	14	anolis_carolinensis:anas_platyrhynchos	10,013,943	10,015,219	Reuse
chicken:100K	14	anas_platyrhynchos:anolis_carolinensis	10,013,943	10,015,219	Reuse
chicken:100K	14	calypte_anna	10,111,290	10,117,562	Unique
chicken:100K	14	charadrius_vociferus	10,702,958	10,718,811	Unique
chicken:100K	14	melopsittacus_undulatus	11,443,743	11,445,309	Unique
chicken:100K	14	anas_platyrhynchos	11,782,460	11,783,564	Unique
chicken:100K	14	chinese_alligator	11,784,149	11,784,802	Unique
chicken:100K	14	picoides_pubescens	12,118,430	12,122,367	Unique
chicken:100K	14	chrysemys_picta	12,123,664	12,132,246	Unique
chicken:100K	14	columba_livia	12,236,872	12,238,885	Unique
chicken:100K	14	boa_constrictor	12,278,007	12,283,824	Unique
chicken:100K	14	picoides_pubescens:nipponia_nippon	12,301,965	12,306,104	Reuse
chicken:100K	14	nipponia_nippon:picoides_pubescens	12,301,965	12,306,104	Reuse
chicken:100K	14	boa_constrictor	12,559,332	12,561,539	Unique
chicken:100K	14	melopsittacus_undulatus:cuculus_canorus	12,728,478	12,732,885	Reuse
chicken:100K	14	cuculus_canorus:melopsittacus_undulatus	12,728,478	12,732,885	Reuse
chicken:100K	14	cuculus_canorus	13,368,008	13,381,535	Unique
chicken:100K	14	chicken	13,622,820	13,671,914	Unique
chicken:100K	14	picoides_pubescens:cuculus_canorus	13,848,491	13,888,730	Reuse
chicken:100K	14	cuculus_canorus:picoides_pubescens	13,848,491	13,888,730	Reuse
chicken:100K	14	chrysemys_picta	13,891,216	13,895,530	Unique
chicken:100K	14	galliformes	14,455,092	14,459,985	Unique
chicken:100K	14	columba_livia	14,662,043	14,667,249	Unique
chicken:100K	15	manacus_vitellinus:boa_constrictor	327,468	332,568	Reuse
chicken:100K	15	boa_constrictor:manacus_vitellinus	327,468	332,568	Reuse
chicken:100K	15	egretta_garzetta	460,592	463,395	Unique
chicken:100K	15	opossum:columba_livia	1,413,015	1,415,700	Reuse
chicken:100K	15	columba_livia:opossum	1,413,015	1,415,700	Reuse
chicken:100K	15	chrysemys_picta	1,673,970	1,676,568	Unique
chicken:100K	15	passeroidea + corvoidea	2,705,003	2,710,900	Unique
chicken:100K	15	geospiza_fortis	2,840,783	2,846,309	Unique
chicken:100K	15	geospiza_fortis:chinese_alligator	2,998,970	3,003,077	Reuse
chicken:100K	15	chinese_alligator:geospiza_fortis	2,998,970	3,003,077	Reuse
chicken:100K	15	chinese_alligator	3,181,198	3,192,356	Unique
chicken:100K	15	melopsittacus_undulatus	3,343,726	3,353,807	Unique
chicken:100K	15	calypte_anna	3,465,155	3,479,161	Unique
chicken:100K	15	chinese_alligator	4,172,256	4,222,347	Unique
chicken:100K	15	anas_platyrhynchos	4,418,960	4,482,717	Unique
chicken:100K	15	cuculus_canorus	5,193,948	5,197,887	Unique
chicken:100K	15	chinese_alligator	5,252,656	5,264,122	Unique
chicken:100K	15	anolis_carolinensis	5,427,638	5,457,578	Unique
chicken:100K	15	egretta_garzetta	5,592,766	5,596,355	Unique
chicken:100K	15	opossum:chinese_alligator	6,472,223	6,496,613	Reuse
chicken:100K	15	chinese_alligator:opossum	6,472,223	6,496,613	Reuse
chicken:100K	15	struthio_camelus:opossum	7,189,703	7,193,891	Reuse
chicken:100K	15	opossum:struthio_camelus	7,189,703	7,193,891	Reuse
chicken:100K	15	charadrius_vociferus	7,293,626	7,298,658	Unique
chicken:100K	15	picoides_pubescens	7,671,963	7,678,711	Unique
chicken:100K	15	charadrius_vociferus	7,714,135	7,717,525	Unique
chicken:100K	15	galliformes	7,846,314	7,847,872	Unique
chicken:100K	15	egretta_garzetta	8,052,923	8,058,859	Unique
chicken:100K	15	cuculus_canorus	8,080,248	8,081,743	Unique
chicken:100K	15	calypte_anna	8,091,933	8,092,850	Unique
chicken:100K	15	picoides_pubescens	8,421,611	8,430,784	Unique
chicken:100K	15	anolis_carolinensis	9,093,119	9,151,715	Unique
chicken:100K	15	picoides_pubescens	9,174,257	9,179,984	Unique
chicken:100K	15	cuculus_canorus	9,254,385	9,257,275	Unique
chicken:100K	15	picoides_pubescens	9,280,591	9,286,292	Unique
chicken:100K	15	anolis_carolinensis	9,673,595	9,679,213	Unique
chicken:100K	15	galliformes	9,909,445	9,910,262	Unique
chicken:100K	15	struthio_camelus	10,487,534	10,591,127	Unique
chicken:100K	15	passeroidea	11,437,746	11,439,211	Unique
chicken:100K	15	cuculus_canorus	12,206,972	12,215,638	Unique

chicken:100K	17	melopsittacus_undulatus	516,891	518,972	Unique
chicken:100K	17	calypte_anna	546,766	550,426	Unique
chicken:100K	17	aptenodytes_forsteri	872,533	874,379	Unique
chicken:100K	17	opossum:chinese_alligator	937,525	949,635	Reuse
chicken:100K	17	chinese_alligator:opossum	937,525	949,635	Reuse
chicken:100K	17	meleagris_gallopavo	1,347,144	1,349,252	Unique
chicken:100K	17	boa_constrictor	1,441,126	1,468,044	Unique
chicken:100K	17	meleagris_gallopavo	1,585,547	1,588,133	Unique
chicken:100K	17	meleagris_gallopavo:boa_constrictor:chinese_alligator	1,759,025	1,798,999	Reuse
chicken:100K	17	chinese_alligator:meleagris_gallopavo:boa_constrictor	1,759,025	1,798,999	Reuse
chicken:100K	17	boa_constrictor:chinese_alligator:meleagris_gallopavo	1,759,025	1,798,999	Reuse
chicken:100K	17	melopsittacus_undulatus	1,899,922	1,902,822	Unique
chicken:100K	17	opossum:melopsittacus_undulatus:chinese_alligator	2,300,163	2,619,522	Reuse
chicken:100K	17	melopsittacus_undulatus:chinese_alligator:opossum	2,300,163	2,619,522	Reuse
chicken:100K	17	chinese_alligator:opossum:melopsittacus_undulatus	2,300,163	2,619,522	Reuse
chicken:100K	17	calypte_anna	2,871,262	2,877,534	Unique
chicken:100K	17	birds_crocs_turtles	4,606,476	4,612,484	Unique
chicken:100K	17	anas_platyrhynchos	4,993,187	4,994,737	Unique
chicken:100K	17	boa_constrictor	5,189,252	5,249,649	Unique
chicken:100K	17	calypte_anna	6,169,421	6,173,610	Unique
chicken:100K	17	melopsittacus_undulatus	6,771,755	6,773,186	Unique
chicken:100K	17	boa_constrictor	7,634,401	7,637,037	Unique
chicken:100K	17	anas_platyrhynchos	7,716,805	7,720,537	Unique
chicken:100K	17	meleagris_gallopavo:boa_constrictor	7,972,517	8,102,391	Reuse
chicken:100K	17	boa_constrictor:meleagris_gallopavo	7,972,517	8,102,391	Reuse
chicken:100K	17	opossum:chinese_alligator	8,227,719	8,268,026	Reuse
chicken:100K	17	chinese_alligator:opossum	8,227,719	8,268,026	Reuse
chicken:100K	17	calypte_anna	8,306,975	8,319,100	Unique
chicken:100K	17	chinese_alligator:boa_constrictor	8,357,724	8,382,522	Reuse
chicken:100K	17	boa_constrictor:chinese_alligator	8,357,724	8,382,522	Reuse
chicken:100K	17	struthio_camelus	8,683,710	8,686,100	Unique
chicken:100K	17	struthio_camelus	8,865,443	8,867,381	Unique
chicken:100K	17	chrysemys_picta	9,200,662	9,208,405	Unique
chicken:100K	17	boa_constrictor	9,518,089	9,519,068	Unique
chicken:100K	17	calypte_anna	10,242,897	10,250,143	Unique
chicken:100K	18	meleagris_gallopavo:chrysemys_picta	344,992	474,796	Reuse
chicken:100K	18	chrysemys_picta:meleagris_gallopavo	344,992	474,796	Reuse
chicken:100K	18	chrysemys_picta	1,493,256	1,497,330	Unique
chicken:100K	18	chrysemys_picta:calypte_anna	1,641,724	1,667,345	Reuse
chicken:100K	18	calypte_anna:chrysemys_picta	1,641,724	1,667,345	Reuse
chicken:100K	18	taeniopygia_guttata	2,175,722	2,177,813	Unique
chicken:100K	18	melopsittacus_undulatus	2,197,952	2,212,185	Unique
chicken:100K	18	nipponia_nippon	2,536,969	2,538,782	Unique
chicken:100K	18	passeroidea + corvoidea	2,721,575	2,730,879	Unique
chicken:100K	18	nipponia_nippon	2,795,267	2,797,787	Unique
chicken:100K	18	cuculus_canorus	2,937,327	2,972,724	Unique
chicken:100K	18	struthio_camelus:anas_platyrhynchos:picoides_pubescens	2,977,399	2,978,963	Reuse
chicken:100K	18	picoides_pubescens:struthio_camelus:anas_platyrhynchos	2,977,399	2,978,963	Reuse
chicken:100K	18	anas_platyrhynchos:picoides_pubescens:struthio_camelus	2,977,399	2,978,963	Reuse
chicken:100K	18	falco_peregrinus:cuculus_canorus	4,438,865	4,439,102	Reuse
chicken:100K	18	cuculus_canorus:falco_peregrinus	4,438,865	4,439,102	Reuse
chicken:100K	18	columba_livia	4,441,169	4,449,652	Unique
chicken:100K	18	calypte_anna	4,576,367	4,580,715	Unique
chicken:100K	18	cuculus_canorus:columba_livia	4,598,838	4,600,098	Reuse
chicken:100K	18	columba_livia:cuculus_canorus	4,598,838	4,600,098	Reuse
chicken:100K	18	chicken	5,037,149	5,038,379	Unique
chicken:100K	18	nipponia_nippon	5,139,838	5,142,374	Unique
chicken:100K	18	anolis_carolinensis	5,399,487	5,403,769	Unique
chicken:100K	18	chrysemys_picta	5,678,684	5,683,509	Unique
chicken:100K	18	columba_livia	6,003,591	6,012,147	Unique
chicken:100K	18	nipponia_nippon	6,229,685	6,230,515	Unique
chicken:100K	18	calypte_anna	6,249,462	6,255,483	Unique
chicken:100K	18	taeniopygia_guttata	6,541,531	6,543,304	Unique
chicken:100K	18	anas_platyrhynchos	6,961,049	6,965,328	Unique
chicken:100K	18	taeniopygia_guttata:chinese_alligator	7,980,593	8,250,097	Reuse
chicken:100K	18	chinese_alligator:taeniopygia_guttata	7,980,593	8,250,097	Reuse

chicken:100K	18	chinese_alligator	8,612,970	8,643,699	Unique
chicken:100K	18	anas_platyrhynchos	9,203,920	9,320,239	Unique
chicken:100K	18	melopsittacus_undulatus	9,770,064	9,771,895	Unique
chicken:100K	18	meleagris_gallopavo	9,910,767	9,916,607	Unique
chicken:100K	18	cuculus_canorus	10,052,903	10,056,333	Unique
chicken:100K	18	meleagris_gallopavo	10,069,872	10,071,013	Unique
chicken:100K	18	chicken	10,250,901	10,252,190	Unique
chicken:100K	18	anas_platyrhynchos	10,593,362	10,687,316	Unique
chicken:100K	18	struthio_camelus	10,917,258	10,923,536	Unique
chicken:100K	19	melopsittacus_undulatus	306,545	311,205	Unique
chicken:100K	19	chrysemys_picta	651,866	840,830	Unique
chicken:100K	19	calypte_anna	841,648	841,700	Unique
chicken:100K	19	boa_constrictor	848,670	849,259	Unique
chicken:100K	19	columba_livia:calypte_anna	1,338,151	1,340,845	Reuse
chicken:100K	19	calypte_anna:columba_livia	1,338,151	1,340,845	Reuse
chicken:100K	19	chrysemys_picta	2,451,348	2,463,993	Unique
chicken:100K	19	taeniopygia_guttata	2,486,281	2,487,993	Unique
chicken:100K	19	nipponia_nippon	2,615,152	2,617,239	Unique
chicken:100K	19	picoides_pubescens:cuculus_canorus	3,131,482	3,131,770	Reuse
chicken:100K	19	cuculus_canorus:picoides_pubescens	3,131,482	3,131,770	Reuse
chicken:100K	19	boa_constrictor	3,208,604	3,209,047	Unique
chicken:100K	19	melopsittacus_undulatus	3,410,958	3,416,346	Unique
chicken:100K	19	anolis_carolinensis	3,425,933	3,439,350	Unique
chicken:100K	19	calypte_anna	3,501,969	3,506,751	Unique
chicken:100K	19	boa_constrictor	4,171,950	4,183,215	Unique
chicken:100K	19	nipponia_nippon	4,793,009	4,798,436	Unique
chicken:100K	19	cuculus_canorus	4,811,775	4,813,037	Unique
chicken:100K	19	picoides_pubescens	4,818,618	4,823,942	Unique
chicken:100K	19	cuculus_canorus	4,930,197	4,939,586	Unique
chicken:100K	19	melopsittacus_undulatus	5,017,036	5,021,729	Unique
chicken:100K	19	cuculus_canorus	5,156,089	5,163,504	Unique
chicken:100K	19	calypte_anna	5,337,152	5,338,106	Unique
chicken:100K	19	aptenodytes_forsteri	5,475,338	5,477,227	Unique
chicken:100K	19	melopsittacus_undulatus	5,805,617	5,809,568	Unique
chicken:100K	19	picoides_pubescens	6,309,023	6,331,671	Unique
chicken:100K	19	boa_constrictor	6,503,158	6,562,323	Unique
chicken:100K	19	chrysemys_picta	7,072,415	7,074,641	Unique
chicken:100K	19	melopsittacus_undulatus	7,183,359	7,189,699	Unique
chicken:100K	19	anolis_carolinensis	7,212,731	7,220,830	Unique
chicken:100K	19	melopsittacus_undulatus	7,402,645	7,406,182	Unique
chicken:100K	19	picoides_pubescens:opossum	8,082,390	8,086,089	Reuse
chicken:100K	19	opossum:picoides_pubescens	8,082,390	8,086,089	Reuse
chicken:100K	19	anolis_carolinensis	8,098,835	8,122,562	Unique
chicken:100K	19	melopsittacus_undulatus	8,164,070	8,168,121	Unique
chicken:100K	19	pygoscelis_adeliae	8,246,490	8,248,115	Unique
chicken:100K	19	chaetura_pelagica	9,295,612	9,297,214	Unique
chicken:100K	19	cuculus_canorus	9,576,601	9,578,416	Unique
chicken:100K	19	chrysemys_picta:boa_constrictor	9,756,596	9,763,391	Reuse
chicken:100K	19	boa_constrictor:chrysemys_picta	9,756,596	9,763,391	Reuse
chicken:100K	19	nipponia_nippon	9,795,173	9,798,772	Unique
chicken:100K	20	calypte_anna	235,675	276,011	Unique
chicken:100K	20	cuculus_canorus	484,031	492,010	Unique
chicken:100K	20	falco_peregrinus	796,692	802,021	Unique
chicken:100K	20	Passeriformes + Psittaciformes + Falconiformes + Piciformes + Ciconiiformes + Sphenisciformes + Charadriiformes + Opisthocomiformes	1,398,442	1,398,761	Unique
chicken:100K	20	anas_platyrhynchos	1,644,750	1,645,629	Unique
chicken:100K	20	cuculus_canorus	1,670,125	1,766,237	Unique
chicken:100K	20	anas_platyrhynchos	1,834,378	1,834,506	Unique
chicken:100K	20	columba_livia	1,969,527	1,971,364	Unique
chicken:100K	20	meleagris_gallopavo:chrysemys_picta	2,307,928	2,327,908	Reuse
chicken:100K	20	chrysemys_picta:meleagris_gallopavo	2,307,928	2,327,908	Reuse
chicken:100K	20	falco_peregrinus:calypte_anna	2,571,334	2,588,316	Reuse
chicken:100K	20	calypte_anna:falco_peregrinus	2,571,334	2,588,316	Reuse
chicken:100K	20	opossum:falco_peregrinus	2,880,041	2,896,404	Reuse
chicken:100K	20	falco_peregrinus:opossum	2,880,041	2,896,404	Reuse
chicken:100K	20	falco_peregrinus	2,912,249	2,917,570	Unique

chicken:100K	20	meleagris_gallopavo	3,854,032	3,881,165	Unique
chicken:100K	20	egret_ibis	3,949,238	3,951,311	Unique
chicken:100K	20	meleagris_gallopavo	4,100,036	4,105,396	Unique
chicken:100K	20	calypte_anna	4,198,205	4,202,476	Unique
chicken:100K	20	meleagris_gallopavo	4,701,205	4,706,556	Unique
chicken:100K	20	anolis_carolinensis	4,827,323	4,853,153	Unique
chicken:100K	20	melopsittacus_undulatus	5,360,290	5,365,107	Unique
chicken:100K	20	cuculus_canorus	5,454,676	5,457,252	Unique
chicken:100K	20	anas_platyrhynchos	5,489,432	5,490,512	Unique
chicken:100K	20	calypte_anna	5,599,490	5,605,320	Unique
chicken:100K	20	calypte_anna	5,725,393	5,730,394	Unique
chicken:100K	20	passeroidea + corvoidea	6,170,415	6,170,853	Unique
chicken:100K	20	chaetura_pelagica	6,529,277	6,530,709	Unique
chicken:100K	20	chaetura_pelagica	6,705,834	6,705,974	Unique
chicken:100K	20	egret_ibis	6,709,214	6,712,380	Unique
chicken:100K	20	galliformes	7,001,727	7,075,274	Unique
chicken:100K	20	melopsittacus_undulatus	8,006,032	8,020,568	Unique
chicken:100K	20	geospiza_fortis	8,196,647	8,198,501	Unique
chicken:100K	20	falco_peregrinus:anolis_carolinensis	8,967,048	9,138,134	Reuse
chicken:100K	20	anolis_carolinensis:falco_peregrinus	8,967,048	9,138,134	Reuse
chicken:100K	20	chrysemys_picta	9,554,002	9,563,534	Unique
chicken:100K	20	struthio_camelus	10,963,493	10,979,362	Unique
chicken:100K	20	nipponia_nippon	11,240,887	11,243,035	Unique
chicken:100K	20	struthio_camelus	11,594,178	11,598,067	Unique
chicken:100K	20	charadrius_vociferus	11,924,949	11,925,766	Unique
chicken:100K	20	chrysemys_picta	12,080,537	12,086,867	Unique
chicken:100K	20	chrysemys_picta	13,642,431	13,644,382	Unique
chicken:100K	20	calypte_anna:anolis_carolinensis	13,944,863	13,952,897	Reuse
chicken:100K	20	anolis_carolinensis:calypte_anna	13,944,863	13,952,897	Reuse
chicken:100K	20	columba_livia	14,060,815	14,064,820	Unique
chicken:100K	21	non_galloanserae	170,338	171,219	Unique
chicken:100K	21	struthio_camelus	596,195	620,429	Unique
chicken:100K	21	chrysemys_picta	630,979	635,118	Unique
chicken:100K	21	cuculus_canorus	729,102	730,882	Unique
chicken:100K	21	cuculus_canorus	910,264	911,990	Unique
chicken:100K	21	melopsittacus_undulatus	1,372,775	1,376,469	Unique
chicken:100K	21	calypte_anna	1,410,962	1,411,557	Unique
chicken:100K	21	melopsittacus_undulatus	1,798,827	1,807,395	Unique
chicken:100K	21	melopsittacus_undulatus	1,966,938	1,971,747	Unique
chicken:100K	21	manacus_vitellinus	1,973,004	1,975,004	Unique
chicken:100K	21	anas_platyrhynchos	2,040,160	2,069,200	Unique
chicken:100K	21	chinese_alligator	2,069,546	2,083,439	Unique
chicken:100K	21	geospiza_fortis	2,174,693	2,275,137	Unique
chicken:100K	21	taeniopygia_guttata	2,446,661	2,451,283	Unique
chicken:100K	21	calypte_anna	2,510,908	2,526,321	Unique
chicken:100K	21	falco_peregrinus	2,544,212	2,546,344	Unique
chicken:100K	21	falco_peregrinus	3,028,949	3,033,449	Unique
chicken:100K	21	aptenodytes_forsteri	3,112,908	3,114,261	Unique
chicken:100K	21	melopsittacus_undulatus	3,208,191	3,215,015	Unique
chicken:100K	21	calypte_anna	3,446,651	3,456,223	Unique
chicken:100K	21	galliformes	4,422,693	4,425,519	Unique
chicken:100K	21	meleagris_gallopavo	4,438,039	4,439,338	Unique
chicken:100K	21	meleagris_gallopavo	4,547,338	4,553,578	Unique
chicken:100K	21	manacus_vitellinus	4,854,797	4,859,960	Unique
chicken:100K	21	struthio_camelus	4,923,548	4,925,376	Unique
chicken:100K	21	passeroidea + corvoidea	4,942,710	4,943,208	Unique
chicken:100K	21	chrysemys_picta	4,968,374	4,978,322	Unique
chicken:100K	21	melopsittacus_undulatus	5,099,035	5,101,452	Unique
chicken:100K	21	chinese_alligator	5,170,832	5,249,659	Unique
chicken:100K	21	chinese_alligator	5,457,281	5,470,481	Unique
chicken:100K	21	melopsittacus_undulatus	5,485,420	5,493,585	Unique
chicken:100K	21	galliformes	5,833,290	5,834,593	Unique
chicken:100K	21	chrysemys_picta:chinese_alligator:anas_platyrhynchos	6,522,875	6,529,219	Reuse
chicken:100K	21	chinese_alligator:anas_platyrhynchos:chrysemys_picta	6,522,875	6,529,219	Reuse
chicken:100K	21	anas_platyrhynchos:chrysemys_picta:chinese_alligator	6,522,875	6,529,219	Reuse
chicken:100K	22	picoides_pubescens	254,131	254,641	Unique

chicken:100K	22	galliformes	453,912	456,227	Unique
chicken:100K	22	picoides_pubescens	908,609	915,253	Unique
chicken:100K	22	picoides_pubescens	1,156,519	1,160,322	Unique
chicken:100K	22	chrysemys_picta	1,690,414	1,842,704	Unique
chicken:100K	22	chinese_alligator	2,183,705	2,189,991	Unique
chicken:100K	22	calypte_anna	2,261,958	2,264,533	Unique
chicken:100K	22	chrysemys_picta:boa_constrictor	2,285,754	2,513,009	Reuse
chicken:100K	22	boa_constrictor:chrysemys_picta	2,285,754	2,513,009	Reuse
chicken:100K	22	meleagris_gallopavo	3,292,777	3,297,287	Unique
chicken:100K	22	melopsittacus_undulatus	3,300,428	3,301,421	Unique
chicken:100K	22	columba_livia	3,753,123	3,755,859	Unique
chicken:100K	23	galloanserae	228,113	239,938	Unique
chicken:100K	23	meleagris_gallopavo	425,790	431,142	Unique
chicken:100K	23	calypte_anna	556,147	565,853	Unique
chicken:100K	23	melopsittacus_undulatus	1,266,161	1,282,010	Unique
chicken:100K	23	columba_livia	1,463,199	1,469,656	Unique
chicken:100K	23	chinese_alligator	1,526,506	1,527,549	Unique
chicken:100K	23	taeniopygia_guttata	1,599,809	1,606,961	Unique
chicken:100K	23	galloanserae	1,763,321	1,777,800	Unique
chicken:100K	23	pygoscelis_adeliae:nipponia_nippon	2,754,344	2,755,434	Reuse
chicken:100K	23	nipponia_nippon:pygoscelis_adeliae	2,754,344	2,755,434	Reuse
chicken:100K	23	chaetura_pelagica	3,523,113	3,533,463	Unique
chicken:100K	23	calypte_anna	3,596,925	3,603,867	Unique
chicken:100K	23	boa_constrictor	3,956,555	4,032,841	Unique
chicken:100K	23	boa_constrictor	4,294,434	4,300,110	Unique
chicken:100K	23	falco_peregrinus	4,405,342	4,407,222	Unique
chicken:100K	23	calypte_anna	5,408,493	5,412,016	Unique
chicken:100K	23	boa_constrictor	5,486,866	5,488,933	Unique
chicken:100K	23	melopsittacus_undulatus	5,556,171	5,564,572	Unique
chicken:100K	24	cuculus_canorus	403,455	415,574	Unique
chicken:100K	24	taeniopygia_guttata	655,637	661,276	Unique
chicken:100K	24	picoides_pubescens	721,923	818,367	Unique
chicken:100K	24	chrysemys_picta	933,920	946,992	Unique
chicken:100K	24	melopsittacus_undulatus	955,036	955,840	Unique
chicken:100K	24	melopsittacus_undulatus:egretta_garzetta	1,142,703	1,188,004	Reuse
chicken:100K	24	egretta_garzetta:melopsittacus_undulatus	1,142,703	1,188,004	Reuse
chicken:100K	24	anolis_carolinensis	1,409,081	1,415,863	Unique
chicken:100K	24	melopsittacus_undulatus	1,473,741	1,532,736	Unique
chicken:100K	24	passeroidea + corvoidea	1,593,925	1,594,107	Unique
chicken:100K	24	manacus_vitellinus	1,791,493	1,794,698	Unique
chicken:100K	24	struthio_camelus	1,799,944	1,803,933	Unique
chicken:100K	24	picoides_pubescens	2,001,954	2,002,332	Unique
chicken:100K	24	chrysemys_picta	2,110,625	2,114,029	Unique
chicken:100K	24	picoides_pubescens	2,197,988	2,199,717	Unique
chicken:100K	24	anolis_carolinensis	2,477,199	2,479,898	Unique
chicken:100K	24	anas_platyrhynchos	2,790,365	2,792,800	Unique
chicken:100K	24	cuculus_canorus	2,824,376	2,825,898	Unique
chicken:100K	24	melopsittacus_undulatus	2,826,835	2,832,754	Unique
chicken:100K	24	anolis_carolinensis	2,852,747	2,858,524	Unique
chicken:100K	24	taeniopygia_guttata	2,899,692	2,907,440	Unique
chicken:100K	24	picoides_pubescens:egretta_garzetta	3,073,782	3,077,748	Reuse
chicken:100K	24	egretta_garzetta:picoides_pubescens	3,073,782	3,077,748	Reuse
chicken:100K	24	falco_peregrinus:anas_platyrhynchos	3,193,795	3,194,492	Reuse
chicken:100K	24	anas_platyrhynchos:falco_peregrinus	3,193,795	3,194,492	Reuse
chicken:100K	24	columba_livia	3,782,241	3,789,548	Unique
chicken:100K	24	charadrius_vociferus	4,159,948	4,161,418	Unique
chicken:100K	24	geospiza_fortis	4,213,346	4,219,256	Unique
chicken:100K	24	galloanserae	4,335,746	4,337,871	Unique
chicken:100K	24	anas_platyrhynchos	4,368,901	4,372,600	Unique
chicken:100K	24	chrysemys_picta	4,863,560	4,871,337	Unique
chicken:100K	24	boa_constrictor	5,267,389	5,269,111	Unique
chicken:100K	24	anas_platyrhynchos	5,723,893	5,725,770	Unique
chicken:100K	25	cuculus_canorus:corvus_brachyrhynchos	254,587	316,976	Reuse
chicken:100K	25	corvus_brachyrhynchos:cuculus_canorus	254,587	316,976	Reuse
chicken:100K	25	geospiza_fortis	856,092	869,911	Unique
chicken:100K	25	chrysemys_picta	1,928,912	1,953,193	Unique

chicken:100K	26	anas_platyrhynchos	170,057	171,550	Unique
chicken:100K	26	calypte_anna:anolis_carolinensis	466,987	500,577	Reuse
chicken:100K	26	anolis_carolinensis:calypte_anna	466,987	500,577	Reuse
chicken:100K	26	melopsittacus_undulatus	605,276	618,736	Unique
chicken:100K	26	manacus_vitellinus	848,645	853,075	Unique
chicken:100K	26	birds_crocs_turtles	894,522	897,704	Unique
chicken:100K	26	columba_livia	1,056,366	1,062,734	Unique
chicken:100K	26	manacus_vitellinus:anolis_carolinensis	1,458,544	1,461,062	Reuse
chicken:100K	26	anolis_carolinensis:manacus_vitellinus	1,458,544	1,461,062	Reuse
chicken:100K	26	galliformes	1,618,791	1,620,435	Unique
chicken:100K	26	meleagris_gallopavo	1,876,237	1,878,486	Unique
chicken:100K	26	meleagris_gallopavo	1,999,997	2,002,408	Unique
chicken:100K	26	melopsittacus_undulatus	2,022,753	2,029,249	Unique
chicken:100K	26	anolis_carolinensis	2,035,775	2,037,062	Unique
chicken:100K	26	anas_platyrhynchos	2,181,962	2,205,379	Unique
chicken:100K	26	columba_livia	2,425,598	2,428,684	Unique
chicken:100K	26	passeroidea	2,582,558	2,589,751	Unique
chicken:100K	26	anolis_carolinensis	2,958,696	2,976,621	Unique
chicken:100K	26	chrysemys_picta	2,994,251	2,997,324	Unique
chicken:100K	26	melopsittacus_undulatus	3,195,974	3,202,316	Unique
chicken:100K	26	columba_livia	3,415,651	3,418,980	Unique
chicken:100K	26	melopsittacus_undulatus	3,742,876	3,743,208	Unique
chicken:100K	26	galliformes	4,017,086	4,020,180	Unique
chicken:100K	26	melopsittacus_undulatus	4,214,960	4,217,943	Unique
chicken:100K	26	anas_platyrhynchos	4,355,525	4,362,359	Unique
chicken:100K	26	opossum:meleagris_gallopavo	4,625,184	4,629,145	Reuse
chicken:100K	26	meleagris_gallopavo:opossum	4,625,184	4,629,145	Reuse
chicken:100K	26	anas_platyrhynchos	4,674,161	4,676,644	Unique
chicken:100K	26	chinese_alligator	4,790,146	4,792,652	Unique
chicken:100K	26	geospiza_fortis	5,008,533	5,019,693	Unique
chicken:100K	26	calypte_anna	5,053,689	5,056,425	Unique
chicken:100K	26	cuculus_canorus	5,100,118	5,104,890	Unique
chicken:100K	26	meleagris_gallopavo	5,141,455	5,148,269	Unique
chicken:100K	27	taeniopygia_guttata:meleagris_gallopavo	878,954	912,153	Reuse
chicken:100K	27	meleagris_gallopavo:taeniopygia_guttata	878,954	912,153	Reuse
chicken:100K	27	meleagris_gallopavo	1,083,682	1,088,529	Unique
chicken:100K	27	chinese_alligator	1,235,430	1,248,285	Unique
chicken:100K	27	cuculus_canorus	1,693,185	1,694,897	Unique
chicken:100K	27	ophisthocomus_hoazin	1,722,065	1,724,940	Unique
chicken:100K	27	calypte_anna:anas_platyrhynchos	2,181,828	2,187,469	Reuse
chicken:100K	27	anas_platyrhynchos:calypte_anna	2,181,828	2,187,469	Reuse
chicken:100K	27	nipponia_nippon	2,277,023	2,278,313	Unique
chicken:100K	27	cuculus_canorus	2,298,926	2,303,220	Unique
chicken:100K	27	columba_livia	2,317,250	2,325,812	Unique
chicken:100K	27	calypte_anna	2,637,119	2,641,748	Unique
chicken:100K	27	egret_ibis	2,760,380	2,762,465	Unique
chicken:100K	27	cuculus_canorus	2,834,293	2,836,563	Unique
chicken:100K	27	columba_livia	3,116,604	3,122,896	Unique
chicken:100K	27	non_galloanserae	3,237,630	3,240,347	Unique
chicken:100K	27	non_galloanserae	3,364,993	3,366,583	Unique
chicken:100K	27	struthio_camelus	4,183,489	4,206,134	Unique
chicken:100K	27	boa_constrictor	4,647,828	4,665,241	Unique
chicken:100K	27	boa_constrictor	4,819,731	4,823,199	Unique
chicken:100K	27	struthio_camelus	4,864,777	4,875,926	Unique
chicken:100K	27	anolis_carolinensis	4,956,920	4,966,986	Unique
chicken:100K	27	cuculus_canorus	4,972,028	4,977,310	Unique
chicken:100K	28	melopsittacus_undulatus:meleagris_gallopavo	473,029	479,584	Reuse
chicken:100K	28	meleagris_gallopavo:melopsittacus_undulatus	473,029	479,584	Reuse
chicken:100K	28	non_galloanserae	533,261	536,215	Unique
chicken:100K	28	melopsittacus_undulatus	667,124	679,174	Unique
chicken:100K	28	meleagris_gallopavo	693,728	698,852	Unique
chicken:100K	28	falco_peregrinus	844,208	850,023	Unique
chicken:100K	28	calypte_anna	1,620,721	1,625,088	Unique
chicken:100K	28	boa_constrictor	1,709,399	1,720,976	Unique
chicken:100K	28	columba_livia	2,024,696	2,037,041	Unique
chicken:100K	28	picoides_pubescens	2,065,579	2,065,713	Unique

chicken:100K	28	boa_constrictor	2,165,760	2,168,715	Unique
chicken:100K	28	cuculus_canorus	2,822,910	2,823,417	Unique
chicken:100K	28	taeniopygia_guttata	2,824,571	2,828,864	Unique
chicken:100K	28	chrysemys_picta	2,907,132	2,909,392	Unique
chicken:100K	28	struthio_camelus	2,952,658	3,017,357	Unique
chicken:100K	28	falco_peregrinus	3,095,762	3,098,865	Unique
chicken:100K	28	falco_peregrinus	3,699,604	3,717,926	Unique
chicken:100K	28	egretta_garzetta	3,818,220	3,819,681	Unique
chicken:100K	28	opossum:melopsittacus_undulatus	3,911,542	3,922,094	Reuse
chicken:100K	28	melopsittacus_undulatus:opossum	3,911,542	3,922,094	Reuse
chicken:100K	28	boa_constrictor	3,975,264	3,980,778	Unique
chicken:100K	28	falco_peregrinus	4,041,766	4,042,931	Unique
chicken:100K	28	passeroidea + corvoidea	4,082,571	4,084,406	Unique
chicken:100K	28	picoides_pubescens	4,170,415	4,173,921	Unique
chicken:100K	28	columba_livia	4,183,707	4,187,436	Unique
chicken:100K	28	chinese_alligator	4,200,685	4,209,256	Unique
chicken:100K	28	calypte_anna	4,223,004	4,223,314	Unique
chicken:100K	Z	galloanserae	325,642	327,438	Unique
chicken:100K	Z	galloanserae	608,534	608,895	Unique
chicken:100K	Z	taeniopygia_guttata	1,067,653	1,078,488	Unique
chicken:100K	Z	corvus_brachyrhynchos	1,382,037	1,382,563	Unique
chicken:100K	Z	anolis_carolinensis	1,695,743	1,715,567	Unique
chicken:100K	Z	taeniopygia_guttata	1,812,765	1,822,812	Unique
chicken:100K	Z	melopsittacus_undulatus	2,272,653	2,280,566	Unique
chicken:100K	Z	chrysemys_picta	2,924,745	2,941,753	Unique
chicken:100K	Z	corvus_brachyrhynchos	3,121,610	3,126,617	Unique
chicken:100K	Z	corvus_brachyrhynchos	3,927,362	3,929,972	Unique
chicken:100K	Z	ophisthocomus_hoazin	4,048,204	4,054,715	Unique
chicken:100K	Z	chinese_alligator	4,353,096	4,377,399	Unique
chicken:100K	Z	melopsittacus_undulatus	5,340,642	5,344,172	Unique
chicken:100K	Z	birds	9,091,238	9,146,281	Unique
chicken:100K	Z	cuculus_canorus	9,924,640	9,927,805	Unique
chicken:100K	Z	charadrius_vociferus	9,958,193	9,963,047	Unique
chicken:100K	Z	cuculus_canorus	10,957,554	10,959,036	Unique
chicken:100K	Z	manacus_vitellinus:chrysemys_picta	11,114,924	11,130,427	Reuse
chicken:100K	Z	chrysemys_picta:manacus_vitellinus	11,114,924	11,130,427	Reuse
chicken:100K	Z	chrysemys_picta	11,532,395	11,547,344	Unique
chicken:100K	Z	chrysemys_picta	11,742,978	11,757,275	Unique
chicken:100K	Z	columba_livia	12,774,353	12,775,785	Unique
chicken:100K	Z	falco_peregrinus:chrysemys_picta	12,845,288	12,919,881	Reuse
chicken:100K	Z	chrysemys_picta:falco_peregrinus	12,845,288	12,919,881	Reuse
chicken:100K	Z	manacus_vitellinus	13,157,442	13,169,520	Unique
chicken:100K	Z	columba_livia	13,183,001	13,184,811	Unique
chicken:100K	Z	anas_platyrhynchos	15,099,812	15,104,572	Unique
chicken:100K	Z	anas_platyrhynchos	15,221,821	15,224,714	Unique
chicken:100K	Z	chrysemys_picta	16,296,065	16,312,363	Unique
chicken:100K	Z	chinese_alligator:charadrius_vociferus	16,791,441	16,958,754	Reuse
chicken:100K	Z	charadrius_vociferus:chinese_alligator	16,791,441	16,958,754	Reuse
chicken:100K	Z	chinese_alligator	17,234,271	17,579,994	Unique
chicken:100K	Z	picoides_pubescens:egretta_garzetta	17,898,245	17,903,166	Reuse
chicken:100K	Z	egretta_garzetta:picoides_pubescens	17,898,245	17,903,166	Reuse
chicken:100K	Z	picoides_pubescens	18,428,896	18,433,027	Unique
chicken:100K	Z	falco_peregrinus	18,529,109	18,530,964	Unique
chicken:100K	Z	picoides_pubescens	18,592,547	18,594,533	Unique
chicken:100K	Z	nipponia_nippon	18,972,468	18,975,804	Unique
chicken:100K	Z	chrysemys_picta	19,258,514	19,261,682	Unique
chicken:100K	Z	calypte_anna	19,335,100	19,340,788	Unique
chicken:100K	Z	corvus_brachyrhynchos	20,972,795	20,981,761	Unique
chicken:100K	Z	egretta_garzetta	21,467,684	21,469,946	Unique
chicken:100K	Z	struthio_camelus	21,487,641	21,492,981	Unique
chicken:100K	Z	corvus_brachyrhynchos	21,600,514	21,600,731	Unique
chicken:100K	Z	melopsittacus_undulatus	22,287,753	22,295,551	Unique
chicken:100K	Z	falco_peregrinus:cuculus_canorus	22,632,674	22,637,108	Reuse
chicken:100K	Z	cuculus_canorus:falco_peregrinus	22,632,674	22,637,108	Reuse
chicken:100K	Z	opossum:chinese_alligator	23,106,846	23,189,406	Reuse
chicken:100K	Z	chinese_alligator:opossum	23,106,846	23,189,406	Reuse

chicken:100K	Z	ophisthocomus_hoazin	23,600,905	23,615,192	Unique
chicken:100K	Z	taeniopygia_guttata	23,642,099	23,650,576	Unique
chicken:100K	Z	falco_peregrinus	23,815,997	23,847,664	Unique
chicken:100K	Z	calypte_anna	24,780,748	24,786,609	Unique
chicken:100K	Z	neognathae	25,356,301	25,452,746	Unique
chicken:100K	Z	calypte_anna	26,878,435	26,878,975	Unique
chicken:100K	Z	struthio_camelus	26,888,319	26,895,919	Unique
chicken:100K	Z	cuculus_canorus	26,996,252	27,278,069	Unique
chicken:100K	Z	calypte_anna	27,334,778	27,339,400	Unique
chicken:100K	Z	chinese_alligator	27,484,034	27,511,122	Unique
chicken:100K	Z	chrysemys_picta	27,673,440	27,683,327	Unique
chicken:100K	Z	struthio_camelus:chinese_alligator	29,530,084	29,533,997	Reuse
chicken:100K	Z	chinese_alligator:struthio_camelus	29,530,084	29,533,997	Reuse
chicken:100K	Z	birds_crocs_turtles	30,613,453	30,617,971	Unique
chicken:100K	Z	birds_crocs_turtles	30,720,647	30,721,433	Unique
chicken:100K	Z	manacus_vitellinus	31,676,732	31,690,177	Unique
chicken:100K	Z	struthio_camelus	32,461,385	32,462,900	Unique
chicken:100K	Z	cuculus_canorus	33,012,869	33,017,315	Unique
chicken:100K	Z	struthio_camelus	33,033,246	33,033,746	Unique
chicken:100K	Z	struthio_camelus:anolis_carolinensis	33,165,950	33,168,839	Reuse
chicken:100K	Z	anolis_carolinensis:struthio_camelus	33,165,950	33,168,839	Reuse
chicken:100K	Z	chaetura_pelagica	33,292,535	33,294,102	Unique
chicken:100K	Z	galliformes	34,265,683	34,279,915	Unique
chicken:100K	Z	non_galloanserae	34,509,432	34,510,537	Unique
chicken:100K	Z	taeniopygia_guttata	34,662,126	34,677,129	Unique
chicken:100K	Z	struthio_camelus	34,804,736	34,807,244	Unique
chicken:100K	Z	corvus_brachyrhynchos	35,540,785	35,545,313	Unique
chicken:100K	Z	struthio_camelus	35,706,016	35,909,246	Unique
chicken:100K	Z	struthio_camelus	36,501,140	36,600,739	Unique
chicken:100K	Z	nipponia_nippon:chaetura_pelagica	37,150,908	37,160,526	Reuse
chicken:100K	Z	chaetura_pelagica:nipponia_nippon	37,150,908	37,160,526	Reuse
chicken:100K	Z	charadrius_vociferus	37,186,791	37,188,946	Unique
chicken:100K	Z	egretta_garzetta	37,313,246	37,315,499	Unique
chicken:100K	Z	struthio_camelus	37,687,917	37,715,962	Unique
chicken:100K	Z	struthio_camelus	38,143,602	38,144,306	Unique
chicken:100K	Z	charadrius_vociferus	38,863,464	38,872,578	Unique
chicken:100K	Z	struthio_camelus	39,437,000	39,440,035	Unique
chicken:100K	Z	chaetura_pelagica	39,566,286	39,574,221	Unique
chicken:100K	Z	struthio_camelus	39,582,432	39,585,051	Unique
chicken:100K	Z	cuculus_canorus	39,647,312	39,669,436	Unique
chicken:100K	Z	egretta_garzetta	39,727,313	39,729,024	Unique
chicken:100K	Z	chrysemys_picta	40,268,452	40,288,690	Unique
chicken:100K	Z	manacus_vitellinus	41,328,030	41,329,949	Unique
chicken:100K	Z	struthio_camelus	41,473,395	41,497,917	Unique
chicken:100K	Z	struthio_camelus	42,026,747	42,028,443	Unique
chicken:100K	Z	geospiza_fortis	42,535,101	42,538,115	Unique
chicken:100K	Z	opossum:anas_platyrhynchos	42,823,083	42,961,910	Reuse
chicken:100K	Z	anas_platyrhynchos:opossum	42,823,083	42,961,910	Reuse
chicken:100K	Z	nipponia_nippon	43,149,034	43,151,602	Unique
chicken:100K	Z	nipponia_nippon	43,708,800	43,711,721	Unique
chicken:100K	Z	charadrius_vociferus:anas_platyrhynchos	43,748,521	43,859,572	Reuse
chicken:100K	Z	anas_platyrhynchos:charadrius_vociferus	43,748,521	43,859,572	Reuse
chicken:100K	Z	chrysemys_picta	44,016,009	44,025,941	Unique
chicken:100K	Z	charadrius_vociferus	44,349,558	44,352,363	Unique
chicken:100K	Z	cuculus_canorus:charadrius_vociferus	45,122,815	45,128,419	Reuse
chicken:100K	Z	charadrius_vociferus:cuculus_canorus	45,122,815	45,128,419	Reuse
chicken:100K	Z	galliformes	45,523,252	45,523,589	Unique
chicken:100K	Z	opossum:cuculus_canorus	46,138,042	46,139,744	Reuse
chicken:100K	Z	cuculus_canorus:opossum	46,138,042	46,139,744	Reuse
chicken:100K	Z	opossum:melopsittacus_undulatus	47,410,012	47,444,681	Reuse
chicken:100K	Z	melopsittacus_undulatus:opossum	47,410,012	47,444,681	Reuse
chicken:100K	Z	passeroidea	49,156,076	49,165,289	Unique
chicken:100K	Z	passeroidea	49,579,443	49,582,430	Unique
chicken:100K	Z	ophisthocomus_hoazin	49,861,076	49,866,122	Unique
chicken:100K	Z	pygoscelis_adeliae	50,098,109	50,099,945	Unique
chicken:100K	Z	geospiza_fortis	50,196,682	50,303,462	Unique

chicken:100K	Z	anas_platyrhynchos	50,499,258	50,499,707	Unique
chicken:100K	Z	anas_platyrhynchos	50,619,010	50,620,947	Unique
chicken:100K	Z	melopsittacus_undulatus	51,061,434	51,067,128	Unique
chicken:100K	Z	neognathae	51,459,086	51,601,930	Unique
chicken:100K	Z	egretta_garzetta	52,549,762	52,557,037	Unique
chicken:100K	Z	charadrius_vociferus	52,658,680	52,660,807	Unique
chicken:100K	Z	columba_livia	52,766,676	52,770,272	Unique
chicken:100K	Z	Trochiliformes + Apodiformes	53,287,877	53,293,168	Unique
chicken:100K	Z	anolis_carolinensis	53,696,971	53,705,247	Unique
chicken:100K	Z	corvus_brachyrhynchos	53,818,831	53,822,710	Unique
chicken:100K	Z	chrysemys_picta	54,473,359	54,530,403	Unique
chicken:100K	Z	anolis_carolinensis	54,718,872	54,720,917	Unique
chicken:100K	Z	passeroidea + corvoidea	54,824,372	54,824,429	Unique
chicken:100K	Z	chrysemys_picta	55,196,032	55,196,940	Unique
chicken:100K	Z	falco_peregrinus	55,443,709	55,447,243	Unique
chicken:100K	Z	spheniscidae:charadrius_vociferus	55,849,715	55,852,885	Reuse
chicken:100K	Z	charadrius_vociferus:spheniscidae	55,849,715	55,852,885	Reuse
chicken:100K	Z	struthio_camelus:charadrius_vociferus	55,991,606	56,005,251	Reuse
chicken:100K	Z	charadrius_vociferus:struthio_camelus	55,991,606	56,005,251	Reuse
chicken:100K	Z	cuculus_canorus	56,016,673	56,021,987	Unique
chicken:100K	Z	egretta_garzetta:cuculus_canorus	56,157,152	56,159,590	Reuse
chicken:100K	Z	cuculus_canorus:egretta_garzetta	56,157,152	56,159,590	Reuse
chicken:100K	Z	egretta_garzetta:cuculus_canorus	56,274,378	56,281,943	Reuse
chicken:100K	Z	cuculus_canorus:egretta_garzetta	56,274,378	56,281,943	Reuse
chicken:100K	Z	galliformes	56,823,968	56,827,515	Unique
chicken:100K	Z	chrysemys_picta	58,068,459	58,075,465	Unique
chicken:100K	Z	picoides_pubescens	58,545,512	58,550,274	Unique
chicken:100K	Z	picoides_pubescens	59,232,148	59,236,375	Unique
chicken:100K	Z	pygoscelis_adeliae	60,633,093	60,814,567	Unique
chicken:100K	Z	anas_platyrhynchos	60,937,290	60,939,297	Unique
chicken:100K	Z	passeroidea + corvoidea	60,958,865	61,003,525	Unique
chicken:100K	Z	anas_platyrhynchos	61,094,383	61,097,356	Unique
chicken:100K	Z	pygoscelis_adeliae	61,210,181	61,222,681	Unique
chicken:100K	Z	anas_platyrhynchos	61,308,104	61,309,074	Unique
chicken:100K	Z	corvus_brachyrhynchos	61,413,082	61,426,868	Unique
chicken:100K	Z	geospiza_fortis	61,745,006	61,751,428	Unique
chicken:100K	Z	geospiza_fortis	61,918,969	61,919,857	Unique
chicken:100K	Z	struthio_camelus	62,465,280	62,467,138	Unique
chicken:100K	Z	struthio_camelus	62,585,435	62,607,002	Unique
chicken:100K	Z	cuculus_canorus:anas_platyrhynchos	63,567,420	63,567,662	Reuse
chicken:100K	Z	anas_platyrhynchos:cuculus_canorus	63,567,420	63,567,662	Reuse
chicken:100K	Z	corvus_brachyrhynchos	63,655,276	63,660,650	Unique
chicken:100K	Z	birds_crocs_turtles	65,314,512	65,319,293	Unique
chicken:100K	Z	chaetura_pelagica	65,841,300	65,849,246	Unique
chicken:100K	Z	picoides_pubescens:opossum	66,298,329	66,326,285	Reuse
chicken:100K	Z	opossum:picoides_pubescens	66,298,329	66,326,285	Reuse
chicken:100K	Z	struthio_camelus	67,454,337	67,504,692	Unique
chicken:100K	Z	struthio_camelus:chrysemys_picta	68,069,168	68,075,403	Reuse
chicken:100K	Z	chrysemys_picta:struthio_camelus	68,069,168	68,075,403	Reuse
chicken:100K	Z	corvus_brachyrhynchos:chaetura_pelagica	68,421,708	68,435,486	Reuse
chicken:100K	Z	chaetura_pelagica:corvus_brachyrhynchos	68,421,708	68,435,486	Reuse
chicken:100K	Z	passeroidea	69,449,563	69,450,662	Unique
chicken:100K	Z	geospiza_fortis	69,573,761	69,576,790	Unique
chicken:100K	Z	aptenodytes_forsteri	69,580,942	69,584,173	Unique
chicken:100K	Z	melopsittacus_undulatus	69,916,741	69,920,985	Unique
chicken:100K	Z	galloanserae	70,827,839	70,848,054	Unique
chicken:100K	Z	galloanserae	72,994,135	72,998,425	Unique
chicken:100K	Z	anolis_carolinensis	78,723,240	78,731,314	Unique
chicken:100K	Z	taeniopygia_guttata	78,832,244	78,902,768	Unique
chicken:100K	Z	geospiza_fortis	81,463,790	81,479,690	Unique
chicken:300K	1	struthio_camelus	423,780	671,547	Unique
chicken:300K	1	geospiza_fortis	1,125,949	1,145,018	Unique
chicken:300K	1	falco_peregrinus	1,162,520	1,326,161	Unique
chicken:300K	1	anolis_carolinensis	4,281,085	4,285,281	Unique
chicken:300K	1	chrysemys_picta	4,507,338	4,516,008	Unique
chicken:300K	1	meleagris_gallopavo	5,510,025	5,513,465	Unique

chicken:300K	1	falco_peregrinus	6,029,336	6,031,512	Unique
chicken:300K	1	birds_crocs_turtles	6,075,032	6,083,842	Unique
chicken:300K	1	anolis_carolinensis:struthio_camelus	7,232,548	7,236,580	Reuse
chicken:300K	1	struthio_camelus:anolis_carolinensis	7,232,548	7,236,580	Reuse
chicken:300K	1	falco_peregrinus	7,654,682	7,915,408	Unique
chicken:300K	1	chrysemys_picta	8,347,267	8,351,964	Unique
chicken:300K	1	nipponia_nippon	8,408,427	8,410,084	Unique
chicken:300K	1	chicken	8,882,547	8,887,758	Unique
chicken:300K	1	chicken_turkey	9,219,587	9,223,977	Unique
chicken:300K	1	meleagris_gallopavo	9,283,764	9,299,189	Unique
chicken:300K	1	falco_peregrinus	9,887,440	9,888,690	Unique
chicken:300K	1	anolis_carolinensis	9,926,144	9,951,090	Unique
chicken:300K	1	struthio_camelus	10,911,607	10,916,109	Unique
chicken:300K	1	chaetura_pelagica	12,726,035	12,729,399	Unique
chicken:300K	1	birds_crocs_turtles	15,674,033	15,769,959	Unique
chicken:300K	1	chrysemys_picta	16,127,058	16,443,669	Unique
chicken:300K	1	melopsittacus_undulatus	18,411,634	18,414,857	Unique
chicken:300K	1	anolis_carolinensis	18,447,833	18,490,318	Unique
chicken:300K	1	struthio_camelus	19,429,651	19,433,176	Unique
chicken:300K	1	melopsittacus_undulatus	19,576,458	19,747,900	Unique
chicken:300K	1	anolis_carolinensis	19,760,133	19,877,001	Unique
chicken:300K	1	anolis_carolinensis	22,845,398	23,050,166	Unique
chicken:300K	1	anas_platyrhynchos	23,926,371	23,927,139	Unique
chicken:300K	1	boa_constrictor	25,012,787	25,054,385	Unique
chicken:300K	1	melopsittacus_undulatus	25,804,207	25,806,627	Unique
chicken:300K	1	pygoscelis_adeliae	26,026,802	26,031,490	Unique
chicken:300K	1	chrysemys_picta	26,537,742	27,212,312	Unique
chicken:300K	1	melopsittacus_undulatus	28,694,712	28,701,534	Unique
chicken:300K	1	aptenodytes_forsteri	29,059,748	29,062,983	Unique
chicken:300K	1	chrysemys_picta	32,420,483	32,432,940	Unique
chicken:300K	1	cuculus_canorus	33,346,065	33,628,144	Unique
chicken:300K	1	picoides_pubescens:melopsittacus_undulatus	33,803,844	33,809,977	Reuse
chicken:300K	1	melopsittacus_undulatus:picoides_pubescens	33,803,844	33,809,977	Reuse
chicken:300K	1	anolis_carolinensis	34,037,387	34,061,098	Unique
chicken:300K	1	chrysemys_picta	34,668,164	34,677,307	Unique
chicken:300K	1	chinese_alligator	34,872,919	35,013,957	Unique
chicken:300K	1	coockoo_humingbird_swift	35,388,849	35,396,628	Unique
chicken:300K	1	anolis_carolinensis	35,772,105	35,807,731	Unique
chicken:300K	1	melopsittacus_undulatus	36,330,037	36,335,101	Unique
chicken:300K	1	chrysemys_picta	37,335,309	37,343,883	Unique
chicken:300K	1	melopsittacus_undulatus	37,370,851	37,373,049	Unique
chicken:300K	1	picoides_pubescens	37,500,853	38,057,719	Unique
chicken:300K	1	charadrius_vociferus	38,075,586	38,080,977	Unique
chicken:300K	1	columba_livia	38,301,854	38,369,907	Unique
chicken:300K	1	anolis_carolinensis	39,188,146	39,223,463	Unique
chicken:300K	1	chrysemys_picta	39,271,206	39,282,534	Unique
chicken:300K	1	picoides_pubescens	39,388,194	39,393,279	Unique
chicken:300K	1	chinese_alligator:corvus_brachyrhynchos	39,614,591	39,615,487	Reuse
chicken:300K	1	corvus_brachyrhynchos:chinese_alligator	39,614,591	39,615,487	Reuse
chicken:300K	1	falco_peregrinus	39,765,373	39,767,039	Unique
chicken:300K	1	picoides_pubescens:anas_platyrhynchos	40,082,741	40,090,886	Reuse
chicken:300K	1	anas_platyrhynchos:picoides_pubescens	40,082,741	40,090,886	Reuse
chicken:300K	1	meleagris_gallopavo	40,165,675	40,174,839	Unique
chicken:300K	1	nipponia_nippon	40,806,714	40,810,675	Unique
chicken:300K	1	meleagris_gallopavo	41,240,313	41,241,582	Unique
chicken:300K	1	picoides_pubescens	41,514,440	41,521,301	Unique
chicken:300K	1	chrysemys_picta	42,323,195	42,364,573	Unique
chicken:300K	1	calypte_anna	42,448,909	42,680,501	Unique
chicken:300K	1	melopsittacus_undulatus	44,579,230	44,581,535	Unique
chicken:300K	1	aptenodytes_forsteri	45,012,081	45,015,014	Unique
chicken:300K	1	picoides_pubescens	45,374,102	45,400,745	Unique
chicken:300K	1	calypte_anna	46,987,355	47,063,550	Unique
chicken:300K	1	melopsittacus_undulatus	47,775,553	47,812,526	Unique
chicken:300K	1	anas_platyrhynchos	47,905,042	47,906,033	Unique
chicken:300K	1	anolis_carolinensis	48,103,016	48,107,037	Unique
chicken:300K	1	meleagris_gallopavo	48,609,502	48,610,293	Unique

chicken:300K	1	chrysemys_picta	48,816,763	48,822,539	Unique
chicken:300K	1	chinese_alligator	49,272,288	49,285,121	Unique
chicken:300K	1	anas_platyrhynchos	49,293,025	49,297,846	Unique
chicken:300K	1	anas_platyrhynchos	49,614,627	49,650,033	Unique
chicken:300K	1	chinese_alligator	50,132,260	50,162,740	Unique
chicken:300K	1	charadrius_vociferus	50,554,275	50,556,346	Unique
chicken:300K	1	birds_crocs_turtles	50,757,319	50,995,734	Unique
chicken:300K	1	meleagris_gallopavo	51,597,899	51,598,769	Unique
chicken:300K	1	melopsittacus_undulatus	51,999,326	52,006,775	Unique
chicken:300K	1	nipponia_nippon	52,181,801	52,182,602	Unique
chicken:300K	1	pygoscelis_adeliae	52,234,403	52,239,417	Unique
chicken:300K	1	cuculus_canorus	52,836,116	52,976,300	Unique
chicken:300K	1	melopsittacus_undulatus	53,228,651	53,251,145	Unique
chicken:300K	1	columba_livia	54,633,829	54,854,430	Unique
chicken:300K	1	geospiza_fortis	54,881,392	54,891,581	Unique
chicken:300K	1	melopsittacus_undulatus	56,339,424	56,348,948	Unique
chicken:300K	1	anolis_carolinensis	56,484,852	56,488,897	Unique
chicken:300K	1	corvus_brachyrhynchos	56,534,882	56,538,791	Unique
chicken:300K	1	anolis_carolinensis	57,664,886	57,680,258	Unique
chicken:300K	1	melopsittacus_undulatus	58,166,174	58,171,797	Unique
chicken:300K	1	columba_livia	58,475,637	58,476,339	Unique
chicken:300K	1	chrysemys_picta	59,325,160	59,400,045	Unique
chicken:300K	1	picoides_pubescens	59,805,225	59,839,027	Unique
chicken:300K	1	mgfinch_finch_crow	60,038,442	60,044,624	Unique
chicken:300K	1	aptenodytes_forsteri	60,568,105	60,570,116	Unique
chicken:300K	1	calypte_anna	62,229,438	62,234,790	Unique
chicken:300K	1	columba_livia	62,266,385	62,311,473	Unique
chicken:300K	1	melopsittacus_undulatus	62,315,860	62,324,002	Unique
chicken:300K	1	anolis_carolinensis	62,597,420	62,885,782	Unique
chicken:300K	1	anolis_carolinensis	63,948,060	63,979,107	Unique
chicken:300K	1	chrysemys_picta	64,818,138	64,834,488	Unique
chicken:300K	1	anas_platyrhynchos	65,315,081	65,317,342	Unique
chicken:300K	1	taeniopygia_guttata	65,336,665	65,345,112	Unique
chicken:300K	1	meleagris_gallopavo	66,340,921	66,345,507	Unique
chicken:300K	1	Passeriformes	66,927,502	66,949,983	Unique
chicken:300K	1	chaetura_pelagica	67,134,060	67,185,805	Unique
chicken:300K	1	geospiza_fortis	67,310,487	67,312,400	Unique
chicken:300K	1	taeniopygia_guttata	67,313,916	67,316,840	Unique
chicken:300K	1	picoides_pubescens	67,327,395	67,332,193	Unique
chicken:300K	1	birds_crocs_turtles	67,388,926	67,562,411	Unique
chicken:300K	1	chaetura_pelagica	68,440,857	68,445,290	Unique
chicken:300K	1	meleagris_gallopavo	68,600,040	68,886,196	Unique
chicken:300K	1	melopsittacus_undulatus:chaetura_pelagica	69,211,222	69,212,497	Reuse
chicken:300K	1	chaetura_pelagica:melopsittacus_undulatus	69,211,222	69,212,497	Reuse
chicken:300K	1	meleagris_gallopavo	69,406,978	69,412,807	Unique
chicken:300K	1	chrysemys_picta	69,566,647	69,787,779	Unique
chicken:300K	1	chaetura_pelagica	69,803,287	69,814,376	Unique
chicken:300K	1	meleagris_gallopavo	69,951,079	69,953,442	Unique
chicken:300K	1	melopsittacus_undulatus	70,069,637	70,078,216	Unique
chicken:300K	1	aptenodytes_forsteri	71,255,588	71,258,256	Unique
chicken:300K	1	taeniopygia_guttata	71,782,945	71,828,445	Unique
chicken:300K	1	chrysemys_picta	71,861,324	71,872,803	Unique
chicken:300K	1	chicken	72,413,370	72,414,784	Unique
chicken:300K	1	chicken_turkey	73,159,165	73,168,005	Unique
chicken:300K	1	mgfinch_finch_crow	73,509,077	73,521,425	Unique
chicken:300K	1	meleagris_gallopavo	73,847,983	74,057,896	Unique
chicken:300K	1	chicken_turkey	74,630,982	74,632,558	Unique
chicken:300K	1	chaetura_pelagica	75,523,810	75,532,686	Unique
chicken:300K	1	chicken_turkey	75,870,953	75,948,624	Unique
chicken:300K	1	melopsittacus_undulatus	76,251,962	76,255,885	Unique
chicken:300K	1	nipponia_nippon	76,336,783	76,341,241	Unique
chicken:300K	1	chaetura_pelagica	76,364,861	76,373,558	Unique
chicken:300K	1	calypte_anna	76,390,001	76,395,313	Unique
chicken:300K	1	nipponia_nippon	76,691,643	76,758,826	Unique
chicken:300K	1	melopsittacus_undulatus	76,924,726	76,958,973	Unique
chicken:300K	1	chicken_turkey	77,734,671	77,950,129	Unique

chicken:300K	1	struthio_camelus	78,464,720	78,465,944	Unique
chicken:300K	1	birds_crocs_turtles	78,617,687	78,620,588	Unique
chicken:300K	1	corvus_brachyrhynchos	78,772,630	78,773,124	Unique
chicken:300K	1	melopsittacus_undulatus:columba_livia	79,606,378	79,608,088	Reuse
chicken:300K	1	columba_livia:melopsittacus_undulatus	79,606,378	79,608,088	Reuse
chicken:300K	1	anolis_carolinensis	79,697,945	80,057,392	Unique
chicken:300K	1	nipponia_nippon	80,302,797	80,304,615	Unique
chicken:300K	1	aptenodytes_forsteri	82,024,764	82,026,502	Unique
chicken:300K	1	egretta_garzetta	82,310,897	82,312,599	Unique
chicken:300K	1	melopsittacus_undulatus	82,455,433	82,457,408	Unique
chicken:300K	1	egretta_garzetta	82,644,612	82,645,387	Unique
chicken:300K	1	chaetura_pelagica:opossum	83,538,268	83,564,175	Reuse
chicken:300K	1	opossum:chaetura_pelagica	83,538,268	83,564,175	Reuse
chicken:300K	1	taeniopygia_guttata	83,916,726	83,917,179	Unique
chicken:300K	1	mgfinch_finch_crow	83,977,286	83,984,032	Unique
chicken:300K	1	pygoscelis_adeliae	84,096,533	84,098,016	Unique
chicken:300K	1	melopsittacus_undulatus	86,337,199	86,343,158	Unique
chicken:300K	1	columba_livia:opossum	86,742,609	86,871,590	Reuse
chicken:300K	1	opossum:columba_livia	86,742,609	86,871,590	Reuse
chicken:300K	1	chaetura_pelagica	87,942,689	87,944,890	Unique
chicken:300K	1	manacus_vitellinus	88,092,859	88,101,278	Unique
chicken:300K	1	mgfinch_finch_crow	88,139,067	88,151,044	Unique
chicken:300K	1	melopsittacus_undulatus	88,716,773	89,732,493	Unique
chicken:300K	1	calypte_anna	90,906,153	90,911,569	Unique
chicken:300K	1	columba_livia	91,170,101	91,175,236	Unique
chicken:300K	1	chrysemys_picta:picoides_pubescens:opossum	92,050,007	92,196,865	Reuse
chicken:300K	1	picoides_pubescens:opossum:chrysemys_picta	92,050,007	92,196,865	Reuse
chicken:300K	1	opossum:chrysemys_picta:picoides_pubescens	92,050,007	92,196,865	Reuse
chicken:300K	1	manacus_vitellinus	92,418,689	92,422,087	Unique
chicken:300K	1	melopsittacus_undulatus	93,390,936	93,401,182	Unique
chicken:300K	1	struthio_camelus	93,635,353	93,789,678	Unique
chicken:300K	1	pygoscelis_adeliae	94,168,594	94,171,061	Unique
chicken:300K	1	chrysemys_picta	94,790,830	94,820,847	Unique
chicken:300K	1	manacus_vitellinus:melopsittacus_undulatus	94,863,188	94,866,901	Reuse
chicken:300K	1	melopsittacus_undulatus:manacus_vitellinus	94,863,188	94,866,901	Reuse
chicken:300K	1	struthio_camelus	95,024,959	95,031,570	Unique
chicken:300K	1	anolis_carolinensis	96,158,583	96,182,938	Unique
chicken:300K	1	melopsittacus_undulatus:opossum	97,207,251	97,365,487	Reuse
chicken:300K	1	opossum:melopsittacus_undulatus	97,207,251	97,365,487	Reuse
chicken:300K	1	anolis_carolinensis	97,784,831	97,856,036	Unique
chicken:300K	1	columba_livia	98,957,586	98,963,992	Unique
chicken:300K	1	chaetura_pelagica	99,059,598	99,068,148	Unique
chicken:300K	1	chrysemys_picta:meleagris_gallopavo	99,602,540	99,934,246	Reuse
chicken:300K	1	meleagris_gallopavo:chrysemys_picta	99,602,540	99,934,246	Reuse
chicken:300K	1	meleagris_gallopavo	100,658,381	100,658,586	Unique
chicken:300K	1	chrysemys_picta	101,817,876	102,030,917	Unique
chicken:300K	1	falco_peregrinus	102,080,440	102,130,311	Unique
chicken:300K	1	mgfinch_finch_crow	102,310,866	102,324,116	Unique
chicken:300K	1	chrysemys_picta	102,416,298	102,426,636	Unique
chicken:300K	1	chrysemys_picta	103,204,782	103,229,251	Unique
chicken:300K	1	Passeriformes	103,444,559	103,455,752	Unique
chicken:300K	1	meleagris_gallopavo	103,734,890	103,740,002	Unique
chicken:300K	1	pygoscelis_adeliae	103,786,999	103,788,821	Unique
chicken:300K	1	falco_peregrinus	104,195,564	104,199,261	Unique
chicken:300K	1	anolis_carolinensis	104,525,322	104,716,505	Unique
chicken:300K	1	taeniopygia_guttata	105,211,515	105,349,269	Unique
chicken:300K	1	mgfinch_finch_crow	105,370,435	105,384,938	Unique
chicken:300K	1	geospiza_fortis:opossum	105,954,993	105,969,101	Reuse
chicken:300K	1	opossum:geospiza_fortis	105,954,993	105,969,101	Reuse
chicken:300K	1	meleagris_gallopavo	106,138,711	106,150,991	Unique
chicken:300K	1	chrysemys_picta	106,506,944	106,549,393	Unique
chicken:300K	1	anas_platyrhynchos:columba_livia	107,975,084	107,977,673	Reuse
chicken:300K	1	columba_livia:anas_platyrhynchos	107,975,084	107,977,673	Reuse
chicken:300K	1	chrysemys_picta:opossum	109,641,070	109,716,768	Reuse
chicken:300K	1	opossum:chrysemys_picta	109,641,070	109,716,768	Reuse
chicken:300K	1	corvus_brachyrhynchos	109,808,314	109,809,271	Unique

chicken:300K	1	meleagris_gallopavo:anas_platyrhynchos	111,885,484	111,887,345	Reuse
chicken:300K	1	anas_platyrhynchos:meleagris_gallopavo	111,885,484	111,887,345	Reuse
chicken:300K	1	melopsittacus_undulatus	111,939,623	111,942,334	Unique
chicken:300K	1	anas_platyrhynchos	112,706,301	112,733,100	Unique
chicken:300K	1	columba_livia	112,920,855	112,922,899	Unique
chicken:300K	1	anolis_carolinensis	113,288,049	113,743,498	Unique
chicken:300K	1	chrysemys_picta	114,351,955	114,871,847	Unique
chicken:300K	1	corvus_brachyrhynchos	115,159,850	115,165,845	Unique
chicken:300K	1	melopsittacus_undulatus	115,217,491	115,220,744	Unique
chicken:300K	1	anolis_carolinensis	115,802,911	116,878,343	Unique
chicken:300K	1	nipponia_nippon:chrysemys_picta	117,163,254	117,164,167	Reuse
chicken:300K	1	chrysemys_picta:nipponia_nippon	117,163,254	117,164,167	Reuse
chicken:300K	1	chrysemys_picta	117,606,594	117,610,371	Unique
chicken:300K	1	anas_platyrhynchos	117,818,423	117,821,824	Unique
chicken:300K	1	chrysemys_picta:anas_platyrhynchos	119,250,727	119,256,036	Reuse
chicken:300K	1	anas_platyrhynchos:chrysemys_picta	119,250,727	119,256,036	Reuse
chicken:300K	1	chrysemys_picta	120,147,406	120,157,358	Unique
chicken:300K	1	chrysemys_picta	120,521,278	121,097,355	Unique
chicken:300K	1	picoides_pubescens	121,246,007	121,496,985	Unique
chicken:300K	1	meleagris_gallopavo:picoides_pubescens	122,091,699	122,243,299	Reuse
chicken:300K	1	picoides_pubescens:meleagris_gallopavo	122,091,699	122,243,299	Reuse
chicken:300K	1	chrysemys_picta	124,379,007	124,402,723	Unique
chicken:300K	1	chrysemys_picta	125,451,718	125,536,504	Unique
chicken:300K	1	meleagris_gallopavo	125,783,117	125,787,300	Unique
chicken:300K	1	meleagris_gallopavo	126,277,164	126,288,559	Unique
chicken:300K	1	chrysemys_picta	127,485,646	127,578,313	Unique
chicken:300K	1	Passeriformes	127,801,451	127,804,749	Unique
chicken:300K	1	falco_peregrinus	127,818,501	128,001,910	Unique
chicken:300K	1	chrysemys_picta	128,084,524	128,113,700	Unique
chicken:300K	1	chinese_alligator	129,564,984	129,870,220	Unique
chicken:300K	1	chrysemys_picta	131,024,742	131,039,327	Unique
chicken:300K	1	picoides_pubescens	131,338,446	131,344,399	Unique
chicken:300K	1	anolis_carolinensis:columba_livia:chaetura_pelagica	131,823,156	131,829,869	Reuse
chicken:300K	1	columba_livia:chaetura_pelagica:anolis_carolinensis	131,823,156	131,829,869	Reuse
chicken:300K	1	chaetura_pelagica:anolis_carolinensis:columba_livia	131,823,156	131,829,869	Reuse
chicken:300K	1	meleagris_gallopavo	131,908,115	131,911,152	Unique
chicken:300K	1	meleagris_gallopavo	132,501,353	132,504,105	Unique
chicken:300K	1	chrysemys_picta	132,770,391	132,820,347	Unique
chicken:300K	1	anolis_carolinensis	134,421,246	134,451,426	Unique
chicken:300K	1	chicken_turkey_duck	137,313,100	137,315,743	Unique
chicken:300K	1	meleagris_gallopavo	137,507,947	137,513,954	Unique
chicken:300K	1	cuculus_canorus	138,424,473	138,434,054	Unique
chicken:300K	1	melopsittacus_undulatus	138,566,658	138,572,517	Unique
chicken:300K	1	meleagris_gallopavo	139,032,177	139,066,771	Unique
chicken:300K	1	chicken_turkey_duck	139,331,778	139,333,713	Unique
chicken:300K	1	anolis_carolinensis	139,485,298	139,588,983	Unique
chicken:300K	1	chrysemys_picta	141,157,806	141,164,111	Unique
chicken:300K	1	egretta_garzetta	143,294,320	143,297,229	Unique
chicken:300K	1	chrysemys_picta	144,075,331	144,099,792	Unique
chicken:300K	1	anolis_carolinensis	144,392,892	144,525,842	Unique
chicken:300K	1	aptenodytes_forsteri	144,714,100	144,717,779	Unique
chicken:300K	1	anolis_carolinensis	146,602,292	147,002,228	Unique
chicken:300K	1	chinese_alligator	147,068,497	147,077,576	Unique
chicken:300K	1	pygoscelis_adeliae	147,639,784	147,643,788	Unique
chicken:300K	1	taeniopygia_guttata	149,441,310	149,468,635	Unique
chicken:300K	1	manacus_vitellinus	149,782,988	149,810,958	Unique
chicken:300K	1	ophisthocomus_hoazin:anolis_carolinensis	150,607,459	150,837,396	Reuse
chicken:300K	1	anolis_carolinensis:ophisthocomus_hoazin	150,607,459	150,837,396	Reuse
chicken:300K	1	pygoscelis_adeliae	151,079,499	151,083,119	Unique
chicken:300K	1	ophisthocomus_hoazin	153,015,186	153,019,601	Unique
chicken:300K	1	cuculus_canorus	153,036,353	153,059,491	Unique
chicken:300K	1	anolis_carolinensis	153,137,336	153,198,748	Unique
chicken:300K	1	ophisthocomus_hoazin	154,604,994	154,610,388	Unique
chicken:300K	1	anolis_carolinensis	155,532,367	155,603,409	Unique
chicken:300K	1	manacus_vitellinus	157,083,652	157,172,969	Unique
chicken:300K	1	aptenodytes_forsteri	157,928,941	157,936,327	Unique

chicken:300K	1	chrysemys_picta	158,034,167	158,057,106	Unique
chicken:300K	1	melopsittacus_undulatus	159,620,982	159,680,092	Unique
chicken:300K	1	chrysemys_picta	160,933,521	161,484,070	Unique
chicken:300K	1	egretta_garzetta	161,803,049	161,805,497	Unique
chicken:300K	1	meleagris_gallopavo	163,249,257	163,870,382	Unique
chicken:300K	1	aptenodytes_forsteri	167,067,659	167,069,432	Unique
chicken:300K	1	anolis_carolinensis	167,711,869	167,974,172	Unique
chicken:300K	1	aptenodytes_forsteri	168,105,793	168,109,658	Unique
chicken:300K	1	egretta_garzetta:aptenodytes_forsteri	169,048,476	169,052,939	Reuse
chicken:300K	1	aptenodytes_forsteri:egretta_garzetta	169,048,476	169,052,939	Reuse
chicken:300K	1	meleagris_gallopavo	169,133,510	169,135,697	Unique
chicken:300K	1	meleagris_gallopavo	170,726,415	170,727,584	Unique
chicken:300K	1	manacus_vitellinus	171,611,382	171,612,419	Unique
chicken:300K	1	anolis_carolinensis	175,314,049	175,317,750	Unique
chicken:300K	1	anolis_carolinensis	176,381,785	176,592,647	Unique
chicken:300K	1	cuculus_canorus	177,114,830	177,144,749	Unique
chicken:300K	1	chrysemys_picta	178,094,496	178,308,958	Unique
chicken:300K	1	pygoscelis_adeliae	178,357,941	178,360,818	Unique
chicken:300K	1	struthio_camelus	178,401,152	178,407,963	Unique
chicken:300K	1	taeniopygia_guttata	178,954,904	178,957,416	Unique
chicken:300K	1	anolis_carolinensis	179,164,984	179,793,950	Unique
chicken:300K	1	chrysemys_picta	181,365,602	181,405,805	Unique
chicken:300K	1	cuculus_canorus	182,035,358	182,047,794	Unique
chicken:300K	1	struthio_camelus	184,894,333	184,914,526	Unique
chicken:300K	1	boa_constrictor	185,050,604	185,654,655	Unique
chicken:300K	1	falco_peregrinus	186,379,419	186,478,734	Unique
chicken:300K	1	neoavians	187,544,423	187,546,090	Unique
chicken:300K	1	cuculus_canorus	187,950,216	187,955,312	Unique
chicken:300K	1	cuculus_canorus:picoides_pubescens	189,472,481	189,477,787	Reuse
chicken:300K	1	picoides_pubescens:cuculus_canorus	189,472,481	189,477,787	Reuse
chicken:300K	1	melopsittacus_undulatus	189,928,555	189,931,148	Unique
chicken:300K	1	corvus_brachyrhynchos	192,771,002	192,776,115	Unique
chicken:300K	1	mgfinch_finch	192,816,997	192,823,275	Unique
chicken:300K	1	mgfinch_finch	194,820,489	194,821,907	Unique
chicken:300K	2	non_galloanserae	928,914	971,175	Unique
chicken:300K	2	anas_platyrhynchos	1,311,923	1,313,433	Unique
chicken:300K	2	chicken_turkey	4,047,084	4,051,112	Unique
chicken:300K	2	anolis_carolinensis	5,578,470	5,838,444	Unique
chicken:300K	2	falco_peregrinus	7,651,391	7,657,374	Unique
chicken:300K	2	melopsittacus_undulatus	7,833,126	7,839,039	Unique
chicken:300K	2	falco_peregrinus	7,985,176	8,014,362	Unique
chicken:300K	2	picoides_pubescens	9,565,330	9,612,065	Unique
chicken:300K	2	picoides_pubescens	10,233,846	10,245,986	Unique
chicken:300K	2	chrysemys_picta	10,915,967	10,926,467	Unique
chicken:300K	2	struthio_camelus:aptenodytes_forsteri	14,004,515	14,021,020	Reuse
chicken:300K	2	aptenodytes_forsteri:struthio_camelus	14,004,515	14,021,020	Reuse
chicken:300K	2	picoides_pubescens	14,896,790	14,917,682	Unique
chicken:300K	2	pygoscelis_adeliae	15,097,979	15,100,449	Unique
chicken:300K	2	picoides_pubescens	16,337,599	16,356,696	Unique
chicken:300K	2	picoides_pubescens	16,808,918	16,816,583	Unique
chicken:300K	2	falco_peregrinus	18,278,136	18,282,859	Unique
chicken:300K	2	chrysemys_picta	19,573,392	19,598,295	Unique
chicken:300K	2	chrysemys_picta	19,958,817	19,974,224	Unique
chicken:300K	2	chrysemys_picta	20,484,525	20,717,484	Unique
chicken:300K	2	chrysemys_picta	21,420,367	21,455,567	Unique
chicken:300K	2	chrysemys_picta	22,263,207	22,285,644	Unique
chicken:300K	2	picoides_pubescens	22,741,879	22,763,386	Unique
chicken:300K	2	taeniopygia_guttata	26,116,750	26,118,526	Unique
chicken:300K	2	aptenodytes_forsteri	26,451,737	26,455,906	Unique
chicken:300K	2	chrysemys_picta	26,829,030	26,877,788	Unique
chicken:300K	2	chrysemys_picta	27,442,276	27,450,273	Unique
chicken:300K	2	chinese_alligator:chrysemys_picta	29,197,699	29,249,212	Reuse
chicken:300K	2	chrysemys_picta:chinese_alligator	29,197,699	29,249,212	Reuse
chicken:300K	2	melopsittacus_undulatus	31,014,934	31,023,417	Unique
chicken:300K	2	anolis_carolinensis	32,508,397	32,513,114	Unique
chicken:300K	2	ophisthocomus_hoazin	33,487,563	33,492,713	Unique

chicken:300K	2	anolis_carolinensis	34,287,241	34,369,134	Unique
chicken:300K	2	chinese_alligator	34,956,592	35,236,521	Unique
chicken:300K	2	melopsittacus_undulatus	36,652,868	36,658,497	Unique
chicken:300K	2	anolis_carolinensis	36,924,075	37,359,222	Unique
chicken:300K	2	columba_livia	37,400,122	37,403,989	Unique
chicken:300K	2	anolis_carolinensis	38,230,885	38,971,496	Unique
chicken:300K	2	cuculus_canorus	38,980,849	38,982,141	Unique
chicken:300K	2	anolis_carolinensis:melopsittacus_undulatus	41,675,716	41,683,071	Reuse
chicken:300K	2	melopsittacus_undulatus:anolis_carolinensis	41,675,716	41,683,071	Reuse
chicken:300K	2	picoides_pubescens	41,735,154	41,815,395	Unique
chicken:300K	2	cuculus_canorus	42,115,413	42,182,965	Unique
chicken:300K	2	picoides_pubescens	42,201,703	42,324,228	Unique
chicken:300K	2	charadrius_vociferus	42,749,690	42,759,851	Unique
chicken:300K	2	g7	43,374,939	43,382,016	Unique
chicken:300K	2	boa_constrictor	43,658,775	43,798,884	Unique
chicken:300K	2	ophisthocomus_hoazin	43,888,449	43,893,406	Unique
chicken:300K	2	pygoscelis_adeliae	44,016,868	44,019,713	Unique
chicken:300K	2	anas_platyrhynchos	44,412,270	44,984,828	Unique
chicken:300K	2	anas_platyrhynchos	45,388,302	45,395,092	Unique
chicken:300K	2	anolis_carolinensis	45,513,934	45,689,972	Unique
chicken:300K	2	taeniopygia_guttata	46,454,805	46,458,276	Unique
chicken:300K	2	anas_platyrhynchos	46,592,363	46,604,008	Unique
chicken:300K	2	neoavians	46,618,768	46,626,138	Unique
chicken:300K	2	chrysemys_picta	47,564,452	47,577,214	Unique
chicken:300K	2	cuculus_canorus	48,654,167	48,697,674	Unique
chicken:300K	2	taeniopygia_guttata	49,116,974	49,129,175	Unique
chicken:300K	2	cuculus_canorus	49,539,261	49,539,440	Unique
chicken:300K	2	neoavians	49,684,730	49,732,044	Unique
chicken:300K	2	nipponia_nippon	50,323,769	50,331,860	Unique
chicken:300K	2	columba_livia	50,464,370	50,473,358	Unique
chicken:300K	2	chrysemys_picta	51,643,783	51,658,799	Unique
chicken:300K	2	columba_livia:picoides_pubescens	51,753,929	51,761,990	Reuse
chicken:300K	2	picoides_pubescens:columba_livia	51,753,929	51,761,990	Reuse
chicken:300K	2	chaetura_pelagica	52,387,606	52,390,295	Unique
chicken:300K	2	meleagris_gallopavo	52,718,147	52,750,067	Unique
chicken:300K	2	nipponia_nippon:chrysemys_picta	52,808,248	52,811,678	Reuse
chicken:300K	2	chrysemys_picta:nipponia_nippon	52,808,248	52,811,678	Reuse
chicken:300K	2	egretta_garzetta	52,917,440	52,923,878	Unique
chicken:300K	2	nipponia_nippon	53,328,504	53,359,492	Unique
chicken:300K	2	picoides_pubescens	54,271,250	54,315,000	Unique
chicken:300K	2	taeniopygia_guttata	55,061,661	55,098,773	Unique
chicken:300K	2	struthio_camelus	56,143,114	56,148,510	Unique
chicken:300K	2	neoavians	57,834,983	57,839,617	Unique
chicken:300K	2	meleagris_gallopavo	59,207,282	59,209,076	Unique
chicken:300K	2	calypte_anna	59,709,285	59,712,321	Unique
chicken:300K	2	meleagris_gallopavo:chrysemys_picta	59,850,771	59,852,075	Reuse
chicken:300K	2	chrysemys_picta:meleagris_gallopavo	59,850,771	59,852,075	Reuse
chicken:300K	2	ophisthocomus_hoazin	60,296,742	60,300,829	Unique
chicken:300K	2	chrysemys_picta	60,691,648	60,712,604	Unique
chicken:300K	2	anolis_carolinensis	62,619,229	62,677,603	Unique
chicken:300K	2	anas_platyrhynchos	63,025,671	63,079,431	Unique
chicken:300K	2	taeniopygia_guttata	64,083,924	64,085,056	Unique
chicken:300K	2	picoides_pubescens	64,401,902	64,472,781	Unique
chicken:300K	2	anas_platyrhynchos	66,011,079	66,012,678	Unique
chicken:300K	2	picoides_pubescens:chrysemys_picta	67,748,794	67,823,609	Reuse
chicken:300K	2	chrysemys_picta:picoides_pubescens	67,748,794	67,823,609	Reuse
chicken:300K	2	struthio_camelus	67,859,264	67,866,482	Unique
chicken:300K	2	geospiza_fortis	67,926,125	67,927,167	Unique
chicken:300K	2	columba_livia:chrysemys_picta	69,060,390	69,237,940	Reuse
chicken:300K	2	chrysemys_picta:columba_livia	69,060,390	69,237,940	Reuse
chicken:300K	2	ophisthocomus_hoazin	69,470,177	69,474,153	Unique
chicken:300K	2	chrysemys_picta	70,387,946	70,406,245	Unique
chicken:300K	2	falco_peregrinus	70,532,135	70,564,371	Unique
chicken:300K	2	chrysemys_picta	71,356,398	71,367,163	Unique
chicken:300K	2	chrysemys_picta	71,842,011	71,902,474	Unique
chicken:300K	2	columba_livia	72,385,540	72,386,007	Unique

chicken:300K	2	chrysemys_picta	72,412,147	72,472,029	Unique
chicken:300K	2	meleagris_gallopavo	72,565,588	72,660,123	Unique
chicken:300K	2	taeniopygia_guttata	73,462,415	73,494,799	Unique
chicken:300K	2	chrysemys_picta	73,496,510	73,557,281	Unique
chicken:300K	2	anolis_carolinensis	74,438,667	75,239,376	Unique
chicken:300K	2	anas_platyrhynchos	76,265,023	76,266,817	Unique
chicken:300K	2	falco_peregrinus	76,709,182	76,790,650	Unique
chicken:300K	2	birds:ophisthocomus_hoazin	78,382,275	78,396,083	Reuse
chicken:300K	2	ophisthocomus_hoazin:birds	78,382,275	78,396,083	Reuse
chicken:300K	2	neoavians	79,878,460	79,888,334	Unique
chicken:300K	2	struthio_camelus	81,209,875	81,215,770	Unique
chicken:300K	2	chrysemys_picta	81,296,190	81,314,003	Unique
chicken:300K	2	chrysemys_picta	83,279,801	83,290,012	Unique
chicken:300K	2	chaetura_pelagica	83,506,387	83,507,695	Unique
chicken:300K	2	birds	85,698,636	85,709,753	Unique
chicken:300K	2	anolis_carolinensis	86,425,531	86,455,551	Unique
chicken:300K	2	anolis_carolinensis:chrysemys_picta	88,378,699	88,434,628	Reuse
chicken:300K	2	chrysemys_picta:anolis_carolinensis	88,378,699	88,434,628	Reuse
chicken:300K	2	chaetura_pelagica	88,883,527	88,884,062	Unique
chicken:300K	2	chrysemys_picta	89,119,372	89,123,243	Unique
chicken:300K	2	falco_peregrinus	89,660,923	89,668,960	Unique
chicken:300K	2	neoavians	89,782,722	89,784,600	Unique
chicken:300K	2	neoavians	90,256,252	90,289,209	Unique
chicken:300K	2	picoides_pubescens	91,979,356	91,988,403	Unique
chicken:300K	2	falco_peregrinus	93,814,414	93,816,927	Unique
chicken:300K	2	meleagris_gallopavo	94,160,544	94,162,236	Unique
chicken:300K	2	taeniopygia_guttata	94,197,054	94,203,449	Unique
chicken:300K	2	meleagris_gallopavo	94,561,868	94,565,497	Unique
chicken:300K	2	taeniopygia_guttata	94,827,570	94,834,761	Unique
chicken:300K	2	aptenodytes_forsteri	95,114,680	95,117,372	Unique
chicken:300K	2	pygoscelis_adeliae	96,014,959	96,016,688	Unique
chicken:300K	2	anas_platyrhynchos	96,880,614	96,886,939	Unique
chicken:300K	2	anolis_carolinensis	96,955,191	97,677,649	Unique
chicken:300K	2	struthio_camelus	97,945,564	97,949,845	Unique
chicken:300K	2	pygoscelis_adeliae	98,868,998	98,870,932	Unique
chicken:300K	2	anolis_carolinensis	99,533,379	99,851,865	Unique
chicken:300K	2	pygoscelis_adeliae	100,337,346	100,380,203	Unique
chicken:300K	2	chrysemys_picta	100,616,775	100,626,901	Unique
chicken:300K	2	anolis_carolinensis	100,762,686	100,835,482	Unique
chicken:300K	2	chrysemys_picta	101,281,603	101,307,355	Unique
chicken:300K	2	anas_platyrhynchos	102,943,406	102,945,373	Unique
chicken:300K	2	falco_peregrinus	105,357,995	105,413,652	Unique
chicken:300K	2	anolis_carolinensis	105,489,595	105,536,739	Unique
chicken:300K	2	struthio_camelus	105,901,491	105,905,922	Unique
chicken:300K	2	melopsittacus_undulatus	107,559,592	107,562,725	Unique
chicken:300K	2	pygoscelis_adeliae	108,425,862	108,427,776	Unique
chicken:300K	2	struthio_camelus	109,201,569	109,208,436	Unique
chicken:300K	2	pygoscelis_adeliae	109,557,779	109,559,063	Unique
chicken:300K	2	anas_platyrhynchos	109,781,749	109,784,465	Unique
chicken:300K	2	anolis_carolinensis	111,008,260	111,387,305	Unique
chicken:300K	2	chrysemys_picta	112,189,482	112,198,970	Unique
chicken:300K	2	geospiza_fortis	112,225,596	112,226,472	Unique
chicken:300K	2	melopsittacus_undulatus	114,277,052	114,278,756	Unique
chicken:300K	2	chinese_alligator	115,575,258	115,688,810	Unique
chicken:300K	2	struthio_camelus	116,406,396	116,407,730	Unique
chicken:300K	2	anolis_carolinensis	118,487,810	118,498,553	Unique
chicken:300K	2	chinese_alligator:picoides_pubescens	119,947,980	119,976,619	Reuse
chicken:300K	2	picoides_pubescens:chinese_alligator	119,947,980	119,976,619	Reuse
chicken:300K	2	chrysemys_picta:struthio_camelus	122,444,754	122,449,354	Reuse
chicken:300K	2	struthio_camelus:chrysemys_picta	122,444,754	122,449,354	Reuse
chicken:300K	2	nipponia_nippon	123,291,867	123,293,778	Unique
chicken:300K	2	chrysemys_picta	124,264,540	124,282,656	Unique
chicken:300K	2	anolis_carolinensis	124,530,222	124,554,787	Unique
chicken:300K	2	picoides_pubescens	127,244,401	127,255,289	Unique
chicken:300K	2	anolis_carolinensis	127,543,498	127,591,374	Unique
chicken:300K	2	pygoscelis_adeliae	127,901,162	127,901,403	Unique

chicken:300K	2	chrysemys_picta	128,279,856	128,287,485	Unique
chicken:300K	2	aptenodytes_forsteri	128,340,629	128,343,689	Unique
chicken:300K	2	anolis_carolinensis	128,382,165	128,416,026	Unique
chicken:300K	2	struthio_camelus	128,418,469	128,422,204	Unique
chicken:300K	2	picoides_pubescens	128,701,548	128,858,113	Unique
chicken:300K	2	aptenodytes_forsteri	129,424,372	129,428,230	Unique
chicken:300K	2	chrysemys_picta	130,016,627	130,032,147	Unique
chicken:300K	2	struthio_camelus	131,246,844	131,249,972	Unique
chicken:300K	2	chrysemys_picta	131,305,924	131,315,078	Unique
chicken:300K	2	picoides_pubescens	131,634,467	131,653,656	Unique
chicken:300K	2	manacus_vitellinus	132,109,270	132,114,257	Unique
chicken:300K	2	chrysemys_picta	132,538,681	132,757,194	Unique
chicken:300K	2	birds	134,972,234	135,006,285	Unique
chicken:300K	2	chrysemys_picta	135,864,383	135,878,776	Unique
chicken:300K	2	picoides_pubescens	135,972,434	136,021,056	Unique
chicken:300K	2	birds	137,383,491	137,387,678	Unique
chicken:300K	2	picoides_pubescens	137,870,814	137,878,624	Unique
chicken:300K	2	struthio_camelus	140,138,239	140,146,851	Unique
chicken:300K	2	anolis_carolinensis	140,575,554	140,851,304	Unique
chicken:300K	2	anolis_carolinensis	143,321,917	143,679,266	Unique
chicken:300K	2	struthio_camelus	144,835,742	144,841,026	Unique
chicken:300K	2	falco_peregrinus	145,287,794	145,290,476	Unique
chicken:300K	2	birds_crocs_turtles	146,182,101	146,192,494	Unique
chicken:300K	2	struthio_camelus	146,842,177	146,845,526	Unique
chicken:300K	2	pygoscelis_adeliae	147,466,569	147,467,486	Unique
chicken:300K	2	falco_peregrinus	147,541,362	147,846,389	Unique
chicken:300K	3	struthio_camelus	357,985	359,236	Unique
chicken:300K	3	egretta_garzetta	366,397	369,617	Unique
chicken:300K	3	anolis_carolinensis	1,980,426	2,039,359	Unique
chicken:300K	3	chicken	2,394,598	2,405,742	Unique
chicken:300K	3	picoides_pubescens	3,210,895	3,312,595	Unique
chicken:300K	3	taeniopygia_guttata	4,109,773	4,123,676	Unique
chicken:300K	3	pygoscelis_adeliae	4,840,723	4,840,871	Unique
chicken:300K	3	chicken	5,598,823	5,606,401	Unique
chicken:300K	3	nipponia_nippon	5,923,564	5,925,175	Unique
chicken:300K	3	chrysemys_picta	6,783,447	6,804,463	Unique
chicken:300K	3	chicken_turkey	7,564,535	7,578,446	Unique
chicken:300K	3	anas_platyrhynchos	8,243,627	8,254,311	Unique
chicken:300K	3	chaetura_pelagica	8,387,148	8,438,550	Unique
chicken:300K	3	birds	8,520,863	8,535,637	Unique
chicken:300K	3	picoides_pubescens:anolis_carolinensis	9,561,036	9,575,521	Reuse
chicken:300K	3	anolis_carolinensis:picoides_pubescens	9,561,036	9,575,521	Reuse
chicken:300K	3	falco_peregrinus:struthio_camelus	11,264,680	11,269,331	Reuse
chicken:300K	3	struthio_camelus:falco_peregrinus	11,264,680	11,269,331	Reuse
chicken:300K	3	meleagris_gallopavo	11,579,794	11,585,862	Unique
chicken:300K	3	chinese_alligator	16,098,805	16,259,464	Unique
chicken:300K	3	anolis_carolinensis	16,952,136	16,964,605	Unique
chicken:300K	3	corvus_brachyrhynchos	17,188,768	17,189,038	Unique
chicken:300K	3	struthio_camelus	17,470,948	17,525,202	Unique
chicken:300K	3	columba_livia	17,758,951	17,766,813	Unique
chicken:300K	3	egretta_garzetta	18,083,048	18,085,541	Unique
chicken:300K	3	chrysemys_picta:melopsittacus_undulatus	20,095,193	20,134,530	Reuse
chicken:300K	3	melopsittacus_undulatus:chrysemys_picta	20,095,193	20,134,530	Reuse
chicken:300K	3	picoides_pubescens	20,356,657	20,392,546	Unique
chicken:300K	3	chrysemys_picta	21,101,532	21,268,093	Unique
chicken:300K	3	corvus_brachyrhynchos	21,477,513	21,479,402	Unique
chicken:300K	3	anolis_carolinensis	22,895,372	23,190,113	Unique
chicken:300K	3	chrysemys_picta	23,378,425	23,464,347	Unique
chicken:300K	3	anolis_carolinensis	25,985,032	26,040,209	Unique
chicken:300K	3	taeniopygia_guttata	26,122,615	26,127,210	Unique
chicken:300K	3	birds	26,543,594	26,565,518	Unique
chicken:300K	3	birds_crocs_turtles	27,300,432	27,636,343	Unique
chicken:300K	3	taeniopygia_guttata	28,438,993	28,441,126	Unique
chicken:300K	3	egretta_garzetta	28,484,486	28,493,547	Unique
chicken:300K	3	anolis_carolinensis	28,776,057	28,776,349	Unique
chicken:300K	3	nipponia_nippon	29,963,955	29,967,318	Unique

chicken:300K	3	anolis_carolinensis	30,131,681	30,172,432	Unique
chicken:300K	3	mgfinch_finch_crow	30,203,130	30,204,703	Unique
chicken:300K	3	falco_peregrinus:egretta_garzetta:cuculus_canorus	33,172,086	33,191,956	Reuse
chicken:300K	3	egretta_garzetta:cuculus_canorus:falco_peregrinus	33,172,086	33,191,956	Reuse
chicken:300K	3	cuculus_canorus:falco_peregrinus:egretta_garzetta	33,172,086	33,191,956	Reuse
chicken:300K	3	anolis_carolinensis	33,265,404	33,525,193	Unique
chicken:300K	3	anolis_carolinensis:cuculus_canorus	34,679,881	34,709,495	Reuse
chicken:300K	3	cuculus_canorus:anolis_carolinensis	34,679,881	34,709,495	Reuse
chicken:300K	3	corvus_brachyrhynchos	35,000,702	35,006,708	Unique
chicken:300K	3	struthio_camelus	37,224,096	37,226,700	Unique
chicken:300K	3	chinese_alligator:anas_platyrhynchos	38,115,541	38,123,348	Reuse
chicken:300K	3	anas_platyrhynchos:chinese_alligator	38,115,541	38,123,348	Reuse
chicken:300K	3	struthio_camelus	41,747,124	41,750,271	Unique
chicken:300K	3	melopsittacus_undulatus	41,860,916	41,870,781	Unique
chicken:300K	3	calypte_anna:anolis_carolinensis	42,160,537	42,178,626	Reuse
chicken:300K	3	anolis_carolinensis:calypte_anna	42,160,537	42,178,626	Reuse
chicken:300K	3	anolis_carolinensis	42,621,505	42,774,697	Unique
chicken:300K	3	chinese_alligator	42,800,697	43,188,933	Unique
chicken:300K	3	chrysemys_picta	44,186,247	44,199,568	Unique
chicken:300K	3	ophisthocomus_hoazin	44,428,797	44,430,222	Unique
chicken:300K	3	mgfinch_finch_crow	44,664,812	44,675,084	Unique
chicken:300K	3	birds_crocs	45,149,621	45,152,243	Unique
chicken:300K	3	anolis_carolinensis	47,049,758	47,352,534	Unique
chicken:300K	3	mgfinch_finch_crow	47,422,091	47,423,361	Unique
chicken:300K	3	anolis_carolinensis	48,670,177	48,758,800	Unique
chicken:300K	3	chrysemys_picta	51,152,886	51,186,793	Unique
chicken:300K	3	calypte_anna	51,453,134	51,493,822	Unique
chicken:300K	3	melopsittacus_undulatus	52,415,801	52,432,139	Unique
chicken:300K	3	anolis_carolinensis	52,784,101	53,007,620	Unique
chicken:300K	3	chaetura_pelagica	54,136,314	54,477,699	Unique
chicken:300K	3	corvus_brachyrhynchos	54,847,632	54,849,749	Unique
chicken:300K	3	mgfinch_finch	54,850,519	54,851,694	Unique
chicken:300K	3	anolis_carolinensis	56,263,734	56,464,354	Unique
chicken:300K	3	anolis_carolinensis	56,942,998	56,963,359	Unique
chicken:300K	3	struthio_camelus	58,590,235	58,592,262	Unique
chicken:300K	3	anolis_carolinensis	58,604,857	58,916,847	Unique
chicken:300K	3	falco_peregrinus	61,923,831	62,055,966	Unique
chicken:300K	3	chaetura_pelagica	64,225,811	64,231,525	Unique
chicken:300K	3	anolis_carolinensis	65,191,583	65,203,410	Unique
chicken:300K	3	struthio_camelus	65,645,379	65,651,342	Unique
chicken:300K	3	geospiza_fortis	67,404,827	67,409,769	Unique
chicken:300K	3	anolis_carolinensis	68,001,732	68,128,932	Unique
chicken:300K	3	anolis_carolinensis	68,629,451	69,430,563	Unique
chicken:300K	3	struthio_camelus	69,637,970	69,639,587	Unique
chicken:300K	3	falco_peregrinus	70,640,657	70,678,115	Unique
chicken:300K	3	picoides_pubescens	71,758,857	71,768,026	Unique
chicken:300K	3	picoides_pubescens	72,156,059	72,157,656	Unique
chicken:300K	3	picoides_pubescens	72,595,143	72,625,072	Unique
chicken:300K	3	melopsittacus_undulatus	72,670,426	72,673,346	Unique
chicken:300K	3	geospiza_fortis	72,951,449	72,960,997	Unique
chicken:300K	3	anolis_carolinensis:melopsittacus_undulatus	74,145,584	74,152,981	Reuse
chicken:300K	3	melopsittacus_undulatus:anolis_carolinensis	74,145,584	74,152,981	Reuse
chicken:300K	3	chrysemys_picta	74,404,804	74,419,563	Unique
chicken:300K	3	struthio_camelus	74,816,295	74,819,040	Unique
chicken:300K	3	geospiza_fortis	75,149,623	75,159,429	Unique
chicken:300K	3	anolis_carolinensis	76,384,821	76,407,626	Unique
chicken:300K	3	struthio_camelus	79,690,856	79,695,187	Unique
chicken:300K	3	anolis_carolinensis	80,710,111	80,832,266	Unique
chicken:300K	3	anolis_carolinensis	81,501,222	81,550,132	Unique
chicken:300K	3	picoides_pubescens	81,819,702	81,827,118	Unique
chicken:300K	3	geospiza_fortis	86,132,809	86,303,133	Unique
chicken:300K	3	anolis_carolinensis	86,539,833	86,633,549	Unique
chicken:300K	3	aptenodytes_forsteri	87,175,111	87,177,148	Unique
chicken:300K	3	anolis_carolinensis	87,502,591	87,541,506	Unique
chicken:300K	3	anolis_carolinensis	88,994,470	89,077,886	Unique
chicken:300K	3	struthio_camelus	89,250,610	89,251,944	Unique

chicken:300K	3	ophisthocomus_hoazin	89,596,487	90,009,436	Unique
chicken:300K	3	corvus_brachyrhynchos	90,967,476	90,968,359	Unique
chicken:300K	3	ophisthocomus_hoazin	91,708,720	91,762,166	Unique
chicken:300K	3	chinese_alligator	91,896,786	92,499,651	Unique
chicken:300K	3	anolis_carolinensis:picoides_pubescens	93,356,963	93,364,309	Reuse
chicken:300K	3	picoides_pubescens:anolis_carolinensis	93,356,963	93,364,309	Reuse
chicken:300K	3	anolis_carolinensis	94,181,274	94,227,755	Unique
chicken:300K	3	cuculus_canorus	96,052,801	96,059,765	Unique
chicken:300K	3	melopsittacus_undulatus	96,354,759	96,361,423	Unique
chicken:300K	3	chaetura_pelagica	96,520,444	96,541,258	Unique
chicken:300K	3	melopsittacus_undulatus	96,683,466	96,689,726	Unique
chicken:300K	3	anolis_carolinensis	99,992,424	100,123,044	Unique
chicken:300K	3	chrysemys_picta	100,950,259	100,976,877	Unique
chicken:300K	3	anolis_carolinensis	102,130,372	102,184,683	Unique
chicken:300K	3	aptenodytes_forsteri	106,249,832	106,252,426	Unique
chicken:300K	3	cuculus_canorus	107,038,646	107,047,768	Unique
chicken:300K	3	picoides_pubescens	108,411,758	108,420,098	Unique
chicken:300K	3	geospiza_fortis	109,263,547	109,486,853	Unique
chicken:300K	3	struthio_camelus	109,498,583	109,502,406	Unique
chicken:300K	3	meleagris_gallopavo	109,996,565	109,998,189	Unique
chicken:300K	4	meleagris_gallopavo	427,423	504,124	Unique
chicken:300K	4	birds_crocs	587,203	595,178	Unique
chicken:300K	4	meleagris_gallopavo	812,908	813,289	Unique
chicken:300K	4	chicken_turkey	1,506,626	1,508,524	Unique
chicken:300K	4	meleagris_gallopavo	2,369,472	2,377,626	Unique
chicken:300K	4	Passeriformes	2,895,581	2,896,977	Unique
chicken:300K	4	Passeriformes	3,388,884	3,392,334	Unique
chicken:300K	4	melopsittacus_undulatus	4,091,249	4,093,342	Unique
chicken:300K	4	mgfinch_finch_crow	4,260,909	4,263,947	Unique
chicken:300K	4	chaetura_pelagica	4,337,257	4,340,935	Unique
chicken:300K	4	struthio_camelus	6,760,632	6,762,877	Unique
chicken:300K	4	chrysemys_picta	7,363,984	7,570,512	Unique
chicken:300K	4	birds_crocs	8,096,981	8,122,315	Unique
chicken:300K	4	aptenodytes_forsteri	8,415,160	8,419,605	Unique
chicken:300K	4	melopsittacus_undulatus	8,675,439	8,684,130	Unique
chicken:300K	4	columba_livia	8,690,791	8,707,251	Unique
chicken:300K	4	struthio_camelus	8,950,221	8,953,754	Unique
chicken:300K	4	chicken_turkey	9,481,895	9,530,195	Unique
chicken:300K	4	struthio_camelus	10,147,290	10,153,808	Unique
chicken:300K	4	chicken_turkey	10,936,371	10,942,800	Unique
chicken:300K	4	cuculus_canorus	12,169,894	12,173,621	Unique
chicken:300K	4	chaetura_pelagica	12,500,825	12,509,445	Unique
chicken:300K	4	Passeriformes	13,340,755	13,345,292	Unique
chicken:300K	4	columba_livia	13,756,707	13,932,288	Unique
chicken:300K	4	cuculus_canorus	14,061,412	14,063,254	Unique
chicken:300K	4	pygoscelis_adeliae	14,751,165	14,752,285	Unique
chicken:300K	4	chrysemys_picta	15,023,667	15,035,077	Unique
chicken:300K	4	columba_livia	15,343,102	15,345,468	Unique
chicken:300K	4	anolis_carolinensis:falco_peregrinus	15,690,999	15,704,255	Reuse
chicken:300K	4	falco_peregrinus:anolis_carolinensis	15,690,999	15,704,255	Reuse
chicken:300K	4	columba_livia:anas_platyrhynchos	16,436,980	16,445,038	Reuse
chicken:300K	4	anas_platyrhynchos:columba_livia	16,436,980	16,445,038	Reuse
chicken:300K	4	taeniopygia_guttata	17,284,492	17,286,345	Unique
chicken:300K	4	geospiza_fortis	17,286,387	17,286,591	Unique
chicken:300K	4	calypte_anna	17,476,955	17,530,858	Unique
chicken:300K	4	melopsittacus_undulatus	17,972,089	17,974,013	Unique
chicken:300K	4	columba_livia	17,983,103	18,147,698	Unique
chicken:300K	4	chicken	19,197,800	19,204,786	Unique
chicken:300K	4	pygoscelis_adeliae	20,473,226	20,476,609	Unique
chicken:300K	4	corvus_brachyrhynchos:egretta_garzetta	22,618,556	22,670,686	Reuse
chicken:300K	4	egretta_garzetta:corvus_brachyrhynchos	22,618,556	22,670,686	Reuse
chicken:300K	4	anolis_carolinensis:melopsittacus_undulatus	23,169,805	23,185,692	Reuse
chicken:300K	4	melopsittacus_undulatus:anolis_carolinensis	23,169,805	23,185,692	Reuse
chicken:300K	4	anolis_carolinensis	25,685,215	25,803,724	Unique
chicken:300K	4	pygoscelis_adeliae	27,504,866	27,507,960	Unique
chicken:300K	4	chrysemys_picta	27,640,522	27,664,849	Unique

chicken:300K	4	melopsittacus_undulatus	30,420,803	30,426,405	Unique
chicken:300K	4	anolis_carolinensis	30,757,909	30,767,868	Unique
chicken:300K	4	picoides_pubescens	31,426,037	31,426,428	Unique
chicken:300K	4	chicken_turkey	33,462,945	33,682,125	Unique
chicken:300K	4	chicken_turkey	34,041,841	34,044,217	Unique
chicken:300K	4	chicken_turkey	34,473,618	34,475,058	Unique
chicken:300K	4	chicken_turkey	36,711,308	36,715,137	Unique
chicken:300K	4	anolis_carolinensis	37,171,141	37,208,386	Unique
chicken:300K	4	chinese_alligator	37,744,162	37,806,859	Unique
chicken:300K	4	non_galloanserae	38,688,770	38,696,308	Unique
chicken:300K	4	picoides_pubescens	39,233,077	39,252,203	Unique
chicken:300K	4	anolis_carolinensis	39,417,530	39,766,567	Unique
chicken:300K	4	melopsittacus_undulatus	40,497,561	40,504,359	Unique
chicken:300K	4	chicken_turkey	41,740,401	41,746,757	Unique
chicken:300K	4	anas_platyrhynchos	43,640,989	43,707,372	Unique
chicken:300K	4	aptenodytes_forsteri	45,517,540	45,518,456	Unique
chicken:300K	4	chicken_turkey_duck	45,834,325	45,840,600	Unique
chicken:300K	4	anas_platyrhynchos	45,892,671	45,894,847	Unique
chicken:300K	4	chicken_turkey	46,429,996	46,434,399	Unique
chicken:300K	4	chicken_turkey	48,572,350	48,577,729	Unique
chicken:300K	4	chicken_turkey	49,042,431	49,044,177	Unique
chicken:300K	4	meleagris_gallopavo	49,141,083	49,142,075	Unique
chicken:300K	4	g9	49,792,617	49,889,986	Unique
chicken:300K	4	chicken_turkey_duck	50,405,055	50,409,644	Unique
chicken:300K	4	chicken_turkey	51,215,264	51,216,208	Unique
chicken:300K	4	charadrius_vociferus	51,794,332	51,959,633	Unique
chicken:300K	4	melopsittacus_undulatus	52,379,462	52,406,329	Unique
chicken:300K	4	falco_peregrinus	53,138,363	53,144,877	Unique
chicken:300K	4	chinese_alligator:melopsittacus_undulatus	53,412,841	53,488,469	Reuse
chicken:300K	4	melopsittacus_undulatus:chinese_alligator	53,412,841	53,488,469	Reuse
chicken:300K	4	calypte_anna	53,862,071	53,943,025	Unique
chicken:300K	4	aptenodytes_forsteri	54,323,333	54,325,858	Unique
chicken:300K	4	anas_platyrhynchos	54,333,412	54,385,406	Unique
chicken:300K	4	melopsittacus_undulatus	54,952,620	54,954,385	Unique
chicken:300K	4	anas_platyrhynchos	55,401,395	55,403,856	Unique
chicken:300K	4	calypte_anna	56,305,984	56,332,288	Unique
chicken:300K	4	g2	56,811,761	56,814,510	Unique
chicken:300K	4	chicken_turkey	57,799,990	57,801,833	Unique
chicken:300K	4	anolis_carolinensis	58,463,159	58,603,740	Unique
chicken:300K	4	struthio_camelus	59,491,056	59,495,659	Unique
chicken:300K	4	anas_platyrhynchos:geospiza_fortis	59,566,739	59,582,676	Reuse
chicken:300K	4	geospiza_fortis:anas_platyrhynchos	59,566,739	59,582,676	Reuse
chicken:300K	4	calypte_anna	59,600,516	59,632,524	Unique
chicken:300K	4	chicken_turkey	60,642,480	60,644,959	Unique
chicken:300K	4	anas_platyrhynchos	62,815,625	62,817,700	Unique
chicken:300K	4	picoides_pubescens	62,931,516	62,990,410	Unique
chicken:300K	4	anas_platyrhynchos	63,850,150	63,927,173	Unique
chicken:300K	4	chrysemys_picta	64,055,029	64,077,495	Unique
chicken:300K	4	picoides_pubescens	64,616,993	64,631,141	Unique
chicken:300K	4	anolis_carolinensis	65,443,231	65,502,021	Unique
chicken:300K	4	anolis_carolinensis	66,474,562	66,662,165	Unique
chicken:300K	4	pygoscelis_adeliae	66,957,086	66,959,875	Unique
chicken:300K	4	ophisthocomus_hoazin	67,182,750	67,188,828	Unique
chicken:300K	4	chrysemys_picta	68,383,438	68,405,576	Unique
chicken:300K	4	falco_peregrinus	68,582,526	68,583,205	Unique
chicken:300K	4	egretta_garzetta:anolis_carolinensis:chrysemys_picta	69,623,056	69,637,842	Reuse
chicken:300K	4	anolis_carolinensis:chrysemys_picta:egretta_garzetta	69,623,056	69,637,842	Reuse
chicken:300K	4	chrysemys_picta:egretta_garzetta:anolis_carolinensis	69,623,056	69,637,842	Reuse
chicken:300K	4	chrysemys_picta	70,703,860	70,717,596	Unique
chicken:300K	4	melopsittacus_undulatus	70,865,909	70,957,701	Unique
chicken:300K	4	chrysemys_picta	71,208,319	71,240,968	Unique
chicken:300K	4	picoides_pubescens	72,985,398	73,031,031	Unique
chicken:300K	4	anolis_carolinensis	73,136,251	73,253,034	Unique
chicken:300K	4	pygoscelis_adeliae	73,555,682	73,572,126	Unique
chicken:300K	4	falco_peregrinus:nipponia_nippon	75,877,708	75,879,110	Reuse
chicken:300K	4	nipponia_nippon:falco_peregrinus	75,877,708	75,879,110	Reuse

chicken:300K	4	picoides_pubescens	76,308,041	76,321,489	Unique
chicken:300K	4	boa_constrictor	78,388,800	79,334,190	Unique
chicken:300K	4	calypte_anna:picoides_pubescens	79,518,738	79,526,898	Reuse
chicken:300K	4	picoides_pubescens:calypte_anna	79,518,738	79,526,898	Reuse
chicken:300K	4	chrysemys_picta	79,805,612	79,827,283	Unique
chicken:300K	4	meleagris_gallopavo	79,978,630	79,979,564	Unique
chicken:300K	4	boa_constrictor	80,150,014	80,234,516	Unique
chicken:300K	4	meleagris_gallopavo	80,965,077	80,969,237	Unique
chicken:300K	4	anolis_carolinensis	81,701,673	81,708,094	Unique
chicken:300K	4	boa_constrictor	81,889,928	82,569,298	Unique
chicken:300K	4	pygoscelis_adeliae	84,316,611	84,321,659	Unique
chicken:300K	4	picoides_pubescens	84,825,333	84,858,437	Unique
chicken:300K	4	birds_crocs	85,954,474	85,968,096	Unique
chicken:300K	4	picoides_pubescens	88,166,627	88,173,862	Unique
chicken:300K	5	calypte_anna:falco_peregrinus:meleagris_gallopavo	499,108	518,160	Reuse
chicken:300K	5	falco_peregrinus:meleagris_gallopavo:calypte_anna	499,108	518,160	Reuse
chicken:300K	5	meleagris_gallopavo:calypte_anna:falco_peregrinus	499,108	518,160	Reuse
chicken:300K	5	picoides_pubescens:columba_livia:nipponia_nippon	1,025,137	1,064,987	Reuse
chicken:300K	5	columba_livia:nipponia_nippon:picoides_pubescens	1,025,137	1,064,987	Reuse
chicken:300K	5	nipponia_nippon:picoides_pubescens:columba_livia	1,025,137	1,064,987	Reuse
chicken:300K	5	non_galloanserae	1,654,614	1,655,891	Unique
chicken:300K	5	chrysemys_picta	2,304,870	2,309,984	Unique
chicken:300K	5	non_galloanserae	3,083,549	3,130,950	Unique
chicken:300K	5	chrysemys_picta	3,564,577	4,065,269	Unique
chicken:300K	5	anolis_carolinensis	4,316,712	4,503,798	Unique
chicken:300K	5	cuculus_canorus	4,587,874	4,590,422	Unique
chicken:300K	5	chicken	5,678,440	5,833,354	Unique
chicken:300K	5	chicken	6,518,476	6,520,088	Unique
chicken:300K	5	melopsittacus_undulatus	7,667,880	7,674,323	Unique
chicken:300K	5	anolis_carolinensis	7,894,600	7,911,483	Unique
chicken:300K	5	mgfinch_finch_crow	8,130,928	8,138,386	Unique
chicken:300K	5	chrysemys_picta	8,267,136	8,287,747	Unique
chicken:300K	5	melopsittacus_undulatus	8,632,625	8,635,023	Unique
chicken:300K	5	chinese_alligator:ophisthocomus_hoazin	8,790,208	8,805,358	Reuse
chicken:300K	5	ophisthocomus_hoazin:chinese_alligator	8,790,208	8,805,358	Reuse
chicken:300K	5	anolis_carolinensis	9,169,792	9,242,270	Unique
chicken:300K	5	pygoscelis_adeliae	9,626,194	9,735,513	Unique
chicken:300K	5	anolis_carolinensis:chrysemys_picta	9,926,444	9,938,611	Reuse
chicken:300K	5	chrysemys_picta:anolis_carolinensis	9,926,444	9,938,611	Reuse
chicken:300K	5	pygoscelis_adeliae	10,083,985	10,087,654	Unique
chicken:300K	5	anas_platyrhynchos	11,834,286	11,835,095	Unique
chicken:300K	5	picoides_pubescens	11,869,538	11,876,973	Unique
chicken:300K	5	falco_peregrinus	11,919,510	12,159,142	Unique
chicken:300K	5	chrysemys_picta	12,473,289	12,495,570	Unique
chicken:300K	5	calypte_anna	14,131,378	14,139,637	Unique
chicken:300K	5	anas_platyrhynchos	14,388,271	14,457,529	Unique
chicken:300K	5	calypte_anna	15,289,619	15,298,392	Unique
chicken:300K	5	melopsittacus_undulatus	15,641,029	15,673,725	Unique
chicken:300K	5	anas_platyrhynchos	15,830,289	15,854,361	Unique
chicken:300K	5	chicken_turkey_duck	16,416,680	16,420,174	Unique
chicken:300K	5	anolis_carolinensis	17,071,363	17,082,930	Unique
chicken:300K	5	melopsittacus_undulatus	17,115,509	17,117,046	Unique
chicken:300K	5	anolis_carolinensis	18,161,823	18,266,609	Unique
chicken:300K	5	melopsittacus_undulatus:aptenodytes_forsteri:picoides_pubescens	18,790,407	18,876,331	Reuse
chicken:300K	5	aptenodytes_forsteri:picoides_pubescens:melopsittacus_undulatus	18,790,407	18,876,331	Reuse
chicken:300K	5	picoides_pubescens:melopsittacus_undulatus:aptenodytes_forsteri	18,790,407	18,876,331	Reuse
chicken:300K	5	anolis_carolinensis	19,720,786	19,741,068	Unique
chicken:300K	5	melopsittacus_undulatus	20,700,162	20,707,153	Unique
chicken:300K	5	anolis_carolinensis	20,786,555	20,994,475	Unique
chicken:300K	5	birds_crocs	21,670,108	21,672,529	Unique
chicken:300K	5	falco_peregrinus	22,196,828	22,198,798	Unique
chicken:300K	5	anas_platyrhynchos	22,450,240	22,452,053	Unique
chicken:300K	5	anolis_carolinensis	23,109,978	23,166,405	Unique
chicken:300K	5	melopsittacus_undulatus	23,198,288	23,217,253	Unique
chicken:300K	5	chrysemys_picta:cuculus_canorus	24,157,428	24,158,554	Reuse
chicken:300K	5	cuculus_canorus:chrysemys_picta	24,157,428	24,158,554	Reuse

chicken:300K	5	calypte_anna	24,489,237	24,572,118	Unique
chicken:300K	5	falco_peregrinus	24,751,245	24,843,688	Unique
chicken:300K	5	boa_constrictor	25,434,270	25,459,863	Unique
chicken:300K	5	melopsittacus_undulatus	25,463,064	25,760,117	Unique
chicken:300K	5	chrysemys_picta	26,032,836	26,067,282	Unique
chicken:300K	5	anolis_carolinensis:opossum	26,671,056	26,732,789	Reuse
chicken:300K	5	opossum:anolis_carolinensis	26,671,056	26,732,789	Reuse
chicken:300K	5	melopsittacus_undulatus	26,818,758	26,820,709	Unique
chicken:300K	5	calypte_anna	27,252,898	27,255,611	Unique
chicken:300K	5	aptenodytes_forsteri	28,089,751	28,092,437	Unique
chicken:300K	5	picoides_pubescens	28,318,936	28,326,476	Unique
chicken:300K	5	aptenodytes_forsteri	29,655,314	29,659,106	Unique
chicken:300K	5	chrysemys_picta	30,022,977	30,029,577	Unique
chicken:300K	5	chrysemys_picta	32,357,176	32,366,436	Unique
chicken:300K	5	anolis_carolinensis	33,431,580	33,444,998	Unique
chicken:300K	5	picoides_pubescens	34,346,931	34,353,081	Unique
chicken:300K	5	struthio_camelus	35,340,069	35,343,565	Unique
chicken:300K	5	chrysemys_picta	35,445,149	35,470,094	Unique
chicken:300K	5	anolis_carolinensis	35,512,215	35,565,936	Unique
chicken:300K	5	melopsittacus_undulatus	35,619,779	35,622,065	Unique
chicken:300K	5	anas_platyrhynchos	35,997,809	36,000,902	Unique
chicken:300K	5	chrysemys_picta	36,428,161	36,433,460	Unique
chicken:300K	5	anolis_carolinensis	36,776,202	36,873,114	Unique
chicken:300K	5	cuculus_canorus	38,160,605	38,163,150	Unique
chicken:300K	5	chrysemys_picta	39,036,097	39,040,675	Unique
chicken:300K	5	struthio_camelus	39,478,845	39,483,743	Unique
chicken:300K	5	cuculus_canorus	39,877,213	39,879,963	Unique
chicken:300K	5	melopsittacus_undulatus	40,606,713	40,607,711	Unique
chicken:300K	5	chrysemys_picta	41,215,194	41,239,920	Unique
chicken:300K	5	falco_peregrinus	42,299,220	42,302,334	Unique
chicken:300K	5	egretta_garzetta	43,289,358	43,292,153	Unique
chicken:300K	5	charadrius_vociferus	43,528,031	43,530,038	Unique
chicken:300K	5	birds_crocs_turtles	44,859,392	44,944,181	Unique
chicken:300K	5	chrysemys_picta	45,355,383	45,379,615	Unique
chicken:300K	5	charadrius_vociferus	45,872,562	45,879,177	Unique
chicken:300K	5	meleagris_gallopavo	47,151,314	47,286,084	Unique
chicken:300K	5	meleagris_gallopavo	47,675,693	47,700,934	Unique
chicken:300K	5	anolis_carolinensis	47,854,136	47,878,922	Unique
chicken:300K	5	chrysemys_picta	47,950,958	47,996,300	Unique
chicken:300K	5	chrysemys_picta	48,582,743	48,605,251	Unique
chicken:300K	5	chrysemys_picta	49,911,179	49,927,169	Unique
chicken:300K	5	anolis_carolinensis	50,250,870	50,289,289	Unique
chicken:300K	5	falco_peregrinus	50,527,346	50,536,273	Unique
chicken:300K	5	boa_constrictor:anolis_carolinensis	51,346,053	51,460,274	Reuse
chicken:300K	5	anolis_carolinensis:boa_constrictor	51,346,053	51,460,274	Reuse
chicken:300K	5	chrysemys_picta	51,711,350	51,736,144	Unique
chicken:300K	5	boa_constrictor	51,784,740	51,835,513	Unique
chicken:300K	5	corvus_brachyrhynchos	52,026,935	52,030,607	Unique
chicken:300K	5	chrysemys_picta	52,773,660	52,786,177	Unique
chicken:300K	5	chinese_alligator	53,672,846	53,750,240	Unique
chicken:300K	5	anolis_carolinensis	54,010,068	54,076,230	Unique
chicken:300K	5	chrysemys_picta	54,127,743	54,480,287	Unique
chicken:300K	5	cuculus_canorus:melopsittacus_undulatus	54,830,778	54,835,515	Reuse
chicken:300K	5	melopsittacus_undulatus:cuculus_canorus	54,830,778	54,835,515	Reuse
chicken:300K	5	chrysemys_picta	54,887,928	54,942,828	Unique
chicken:300K	5	boa_constrictor:charadrius_vociferus	55,173,216	55,177,208	Reuse
chicken:300K	5	charadrius_vociferus:boa_constrictor	55,173,216	55,177,208	Reuse
chicken:300K	5	melopsittacus_undulatus	55,196,523	55,198,363	Unique
chicken:300K	5	cuculus_canorus	55,222,439	55,441,832	Unique
chicken:300K	5	pygoscelis_adeliae	55,487,902	55,489,159	Unique
chicken:300K	5	meleagris_gallopavo	55,799,598	55,802,625	Unique
chicken:300K	5	ophisthocomus_hoazin	55,810,037	55,939,468	Unique
chicken:300K	5	boa_constrictor	56,032,958	56,056,367	Unique
chicken:300K	5	meleagris_gallopavo	56,158,749	56,162,953	Unique
chicken:300K	5	chicken_turkey	56,369,968	56,371,811	Unique
chicken:300K	5	meleagris_gallopavo	56,824,648	57,144,279	Unique

chicken:300K	5	melopsittacus_undulatus	57,288,091	57,289,380	Unique
chicken:300K	5	chinese_alligator	57,551,396	57,593,646	Unique
chicken:300K	5	falco_peregrinus	57,846,368	57,854,265	Unique
chicken:300K	5	boa_constrictor	57,884,957	57,949,622	Unique
chicken:300K	5	cuculus_canorus	58,102,424	58,112,939	Unique
chicken:300K	5	chaetura_pelagica	58,118,755	58,124,910	Unique
chicken:300K	5	boa_constrictor	58,265,095	58,286,222	Unique
chicken:300K	5	chicken_turkey	58,601,543	58,603,094	Unique
chicken:300K	6	cuculus_canorus	413,791	749,841	Unique
chicken:300K	6	chrysemys_picta	753,104	1,603,957	Unique
chicken:300K	6	columba_livia:opossum	2,108,879	2,126,676	Reuse
chicken:300K	6	opossum:columba_livia	2,108,879	2,126,676	Reuse
chicken:300K	6	melopsittacus_undulatus:chrysemys_picta	4,003,881	4,013,900	Reuse
chicken:300K	6	chrysemys_picta:melopsittacus_undulatus	4,003,881	4,013,900	Reuse
chicken:300K	6	chicken	6,070,296	6,073,929	Unique
chicken:300K	6	anolis_carolinensis	6,480,390	6,887,453	Unique
chicken:300K	6	mgfinch_finch	7,198,198	7,210,644	Unique
chicken:300K	6	meleagris_gallopavo	7,364,323	7,905,977	Unique
chicken:300K	6	chicken	8,674,888	8,795,281	Unique
chicken:300K	6	chicken	10,016,053	10,021,657	Unique
chicken:300K	6	chicken	10,703,766	10,727,907	Unique
chicken:300K	6	anolis_carolinensis	11,022,584	11,121,240	Unique
chicken:300K	6	Passeriformes	11,149,634	11,153,108	Unique
chicken:300K	6	meleagris_gallopavo	11,568,201	11,578,014	Unique
chicken:300K	6	chrysemys_picta	12,325,044	12,337,513	Unique
chicken:300K	6	columba_livia	12,714,330	12,716,660	Unique
chicken:300K	6	chrysemys_picta	13,192,400	13,207,895	Unique
chicken:300K	6	ophisthocomus_hoazin	13,358,851	13,361,102	Unique
chicken:300K	6	meleagris_gallopavo	13,789,971	13,790,546	Unique
chicken:300K	6	chrysemys_picta	13,923,466	13,937,220	Unique
chicken:300K	6	pygoscelis_adeliae	14,310,596	14,315,071	Unique
chicken:300K	6	falco_peregrinus	14,954,274	14,959,018	Unique
chicken:300K	6	chrysemys_picta	15,080,377	15,129,014	Unique
chicken:300K	6	melopsittacus_undulatus	15,571,507	15,572,968	Unique
chicken:300K	6	cuculus_canorus	16,114,873	16,124,876	Unique
chicken:300K	6	meleagris_gallopavo:opossum	16,292,703	16,295,928	Reuse
chicken:300K	6	opossum:meleagris_gallopavo	16,292,703	16,295,928	Reuse
chicken:300K	6	meleagris_gallopavo	16,632,087	16,751,420	Unique
chicken:300K	6	calypte_anna	17,069,464	17,076,358	Unique
chicken:300K	6	chrysemys_picta:opossum	18,648,270	18,697,513	Reuse
chicken:300K	6	opossum:chrysemys_picta	18,648,270	18,697,513	Reuse
chicken:300K	6	chrysemys_picta	19,929,389	19,942,492	Unique
chicken:300K	6	charadrius_vociferus	22,000,257	22,002,311	Unique
chicken:300K	6	melopsittacus_undulatus	22,070,821	22,074,658	Unique
chicken:300K	6	anolis_carolinensis	22,620,021	22,623,448	Unique
chicken:300K	6	charadrius_vociferus	22,717,531	22,721,730	Unique
chicken:300K	6	cuculus_canorus	25,352,314	25,354,524	Unique
chicken:300K	6	anolis_carolinensis	25,956,351	26,158,183	Unique
chicken:300K	6	anolis_carolinensis	29,729,668	29,766,626	Unique
chicken:300K	6	falco_peregrinus	29,922,315	29,936,724	Unique
chicken:300K	6	anolis_carolinensis	30,891,725	30,924,648	Unique
chicken:300K	6	anolis_carolinensis	32,298,609	32,358,786	Unique
chicken:300K	6	chrysemys_picta	34,056,707	34,087,323	Unique
chicken:300K	6	nipponia_nippon	34,209,014	34,214,391	Unique
chicken:300K	6	nipponia_nippon	34,559,088	34,561,656	Unique
chicken:300K	7	manacus_vitellinus	398,412	404,927	Unique
chicken:300K	7	cuculus_canorus:falco_peregrinus	996,135	1,006,490	Reuse
chicken:300K	7	falco_peregrinus:cuculus_canorus	996,135	1,006,490	Reuse
chicken:300K	7	chrysemys_picta	1,339,266	1,351,094	Unique
chicken:300K	7	columba_livia	1,368,266	1,410,428	Unique
chicken:300K	7	mgfinch_finch_crow	2,184,361	2,190,166	Unique
chicken:300K	7	manacus_vitellinus	2,204,681	2,210,435	Unique
chicken:300K	7	neoavians	2,311,590	2,315,242	Unique
chicken:300K	7	taeniopygia_guttata:picoides_pubescens	3,524,626	3,548,522	Reuse
chicken:300K	7	picoides_pubescens:taeniopygia_guttata	3,524,626	3,548,522	Reuse
chicken:300K	7	taeniopygia_guttata	4,500,197	4,500,975	Unique

chicken:300K	7	manacus_vitellinus:anolis_carolinensis	4,739,224	4,878,595	Reuse
chicken:300K	7	anolis_carolinensis:manacus_vitellinus	4,739,224	4,878,595	Reuse
chicken:300K	7	pygoscelis_adeliae	4,885,804	4,889,908	Unique
chicken:300K	7	manacus_vitellinus:columba_livia	5,334,400	5,334,885	Reuse
chicken:300K	7	columba_livia:manacus_vitellinus	5,334,400	5,334,885	Reuse
chicken:300K	7	taeniopygia_guttata	5,554,442	5,571,609	Unique
chicken:300K	7	chrysemys_picta	6,089,300	6,117,895	Unique
chicken:300K	7	chicken_turkey	6,893,878	6,911,083	Unique
chicken:300K	7	chicken	7,336,101	7,339,690	Unique
chicken:300K	7	aptenodytes_forsteri	8,788,746	8,793,058	Unique
chicken:300K	7	anolis_carolinensis	8,811,258	8,858,082	Unique
chicken:300K	7	chrysemys_picta	9,081,644	9,115,515	Unique
chicken:300K	7	columba_livia	9,275,107	9,281,206	Unique
chicken:300K	7	picoides_pubescens	9,513,620	9,526,053	Unique
chicken:300K	7	chrysemys_picta	10,329,157	10,333,823	Unique
chicken:300K	7	falco_peregrinus	10,953,137	10,958,987	Unique
chicken:300K	7	birds	11,514,164	11,525,238	Unique
chicken:300K	7	calypte_anna	12,022,667	12,035,702	Unique
chicken:300K	7	struthio_camelus	13,376,264	13,378,189	Unique
chicken:300K	7	columba_livia	13,394,943	13,626,182	Unique
chicken:300K	7	aptenodytes_forsteri	13,832,970	13,838,513	Unique
chicken:300K	7	anas_platyrhynchos	14,086,550	14,087,974	Unique
chicken:300K	7	boa_constrictor	15,382,491	15,399,827	Unique
chicken:300K	7	chinese_alligator	16,675,493	16,684,252	Unique
chicken:300K	7	chinese_alligator	17,071,211	17,083,971	Unique
chicken:300K	7	melopsittacus_undulatus	21,369,589	21,372,225	Unique
chicken:300K	7	cuculus_canorus	21,708,370	21,710,524	Unique
chicken:300K	7	cuculus_canorus	22,059,168	22,063,546	Unique
chicken:300K	7	anolis_carolinensis:nipponia_nippon	22,753,402	22,756,738	Reuse
chicken:300K	7	nipponia_nippon:anolis_carolinensis	22,753,402	22,756,738	Reuse
chicken:300K	7	anolis_carolinensis	23,534,976	23,629,770	Unique
chicken:300K	7	anolis_carolinensis	26,139,183	26,187,705	Unique
chicken:300K	7	taeniopygia_guttata	27,786,737	27,787,078	Unique
chicken:300K	7	chrysemys_picta	27,914,687	27,927,763	Unique
chicken:300K	7	chrysemys_picta	28,528,179	28,737,932	Unique
chicken:300K	7	struthio_camelus	29,155,877	29,161,171	Unique
chicken:300K	7	anolis_carolinensis	29,205,132	29,208,212	Unique
chicken:300K	7	anolis_carolinensis	29,650,053	29,769,898	Unique
chicken:300K	7	chrysemys_picta	30,054,713	30,093,355	Unique
chicken:300K	7	chrysemys_picta	30,584,850	30,592,770	Unique
chicken:300K	7	picoides_pubescens	30,722,936	30,726,898	Unique
chicken:300K	7	anolis_carolinensis	33,294,489	33,304,904	Unique
chicken:300K	7	chrysemys_picta	33,391,485	33,397,087	Unique
chicken:300K	7	meleagris_gallopavo	33,538,585	33,554,365	Unique
chicken:300K	7	chinese_alligator	33,611,512	34,511,981	Unique
chicken:300K	7	meleagris_gallopavo	34,739,968	34,750,782	Unique
chicken:300K	7	chrysemys_picta:chinese_alligator	35,066,129	35,180,444	Reuse
chicken:300K	7	chinese_alligator:chrysemys_picta	35,066,129	35,180,444	Reuse
chicken:300K	7	meleagris_gallopavo	35,507,172	35,544,927	Unique
chicken:300K	8	meleagris_gallopavo	391,795	435,627	Unique
chicken:300K	8	anolis_carolinensis	1,107,900	1,172,406	Unique
chicken:300K	8	ophisthocomus_hoazin	1,251,591	1,253,849	Unique
chicken:300K	8	meleagris_gallopavo	1,280,561	1,281,898	Unique
chicken:300K	8	falco_peregrinus	1,300,251	1,303,401	Unique
chicken:300K	8	egretta_garzetta	1,337,573	1,339,293	Unique
chicken:300K	8	melopsittacus_undulatus	1,704,944	1,706,600	Unique
chicken:300K	8	falco_peregrinus	2,637,724	2,642,372	Unique
chicken:300K	8	melopsittacus_undulatus	2,795,069	3,026,473	Unique
chicken:300K	8	calypte_anna	3,561,344	3,597,538	Unique
chicken:300K	8	neoavians	3,968,737	3,995,355	Unique
chicken:300K	8	picoides_pubescens	4,797,639	4,817,837	Unique
chicken:300K	8	columba_livia	5,071,736	5,073,539	Unique
chicken:300K	8	chinese_alligator	5,551,036	5,561,008	Unique
chicken:300K	8	melopsittacus_undulatus	6,050,534	6,058,098	Unique
chicken:300K	8	calypte_anna	6,062,393	6,067,710	Unique
chicken:300K	8	anolis_carolinensis	6,082,835	6,116,739	Unique

chicken:300K	8	anolis_carolinensis	6,602,758	6,700,449	Unique
chicken:300K	8	falco_peregrinus	6,979,710	7,023,495	Unique
chicken:300K	8	chaetura_pelagica	7,055,817	7,082,127	Unique
chicken:300K	8	falco_peregrinus	7,600,945	7,612,748	Unique
chicken:300K	8	columba_livia	7,706,183	7,709,483	Unique
chicken:300K	8	chicken_turkey_duck	8,030,291	8,032,267	Unique
chicken:300K	8	falco_peregrinus	8,647,185	8,741,245	Unique
chicken:300K	8	chicken	9,981,443	9,997,875	Unique
chicken:300K	8	chrysemys_picta	10,438,962	10,459,160	Unique
chicken:300K	8	ophisthocomus_hoazin	10,593,359	10,597,301	Unique
chicken:300K	8	picoides_pubescens	10,875,162	10,879,300	Unique
chicken:300K	8	birds_crocs_turtles	10,985,404	11,017,165	Unique
chicken:300K	8	melopsittacus_undulatus	11,136,138	11,138,691	Unique
chicken:300K	8	meleagris_gallopavo	11,885,274	11,887,182	Unique
chicken:300K	8	melopsittacus_undulatus	12,729,848	12,737,159	Unique
chicken:300K	8	struthio_camelus	12,809,192	12,811,568	Unique
chicken:300K	8	melopsittacus_undulatus:anas_platyrhynchos	13,898,955	13,900,642	Reuse
chicken:300K	8	anas_platyrhynchos:melopsittacus_undulatus	13,898,955	13,900,642	Reuse
chicken:300K	8	struthio_camelus	14,545,573	14,549,643	Unique
chicken:300K	8	manacus_vitellinus	15,376,281	15,386,615	Unique
chicken:300K	8	chrysemys_picta	15,977,562	15,986,709	Unique
chicken:300K	8	corvus_brachyrhynchos	16,578,881	16,580,645	Unique
chicken:300K	8	struthio_camelus	17,503,033	17,505,016	Unique
chicken:300K	8	birds	18,506,022	18,506,040	Unique
chicken:300K	8	picoides_pubescens	18,548,061	18,554,640	Unique
chicken:300K	8	anolis_carolinensis	19,661,296	19,668,616	Unique
chicken:300K	8	anolis_carolinensis	20,085,298	20,090,925	Unique
chicken:300K	8	chrysemys_picta	20,496,234	20,541,208	Unique
chicken:300K	8	columba_livia	20,586,001	20,588,474	Unique
chicken:300K	8	anolis_carolinensis	20,715,723	20,746,966	Unique
chicken:300K	8	geospiza_fortis	21,490,637	21,492,397	Unique
chicken:300K	8	pygoscelis_adeliae	21,628,314	21,631,240	Unique
chicken:300K	8	geospiza_fortis	21,886,161	21,891,971	Unique
chicken:300K	8	anolis_carolinensis	22,181,314	22,238,521	Unique
chicken:300K	8	picoides_pubescens	22,872,510	22,884,898	Unique
chicken:300K	8	columba_livia	23,002,549	23,005,948	Unique
chicken:300K	8	chrysemys_picta	23,113,815	23,114,945	Unique
chicken:300K	8	corvus_brachyrhynchos	23,290,564	23,292,328	Unique
chicken:300K	8	chrysemys_picta	24,288,232	24,296,277	Unique
chicken:300K	8	chrysemys_picta	25,564,078	25,598,820	Unique
chicken:300K	8	chrysemys_picta	26,810,563	26,825,266	Unique
chicken:300K	8	anolis_carolinensis	27,283,078	27,559,349	Unique
chicken:300K	8	chrysemys_picta	27,715,794	27,741,107	Unique
chicken:300K	9	birds_crocs_turtles	338,389	369,169	Unique
chicken:300K	9	struthio_camelus	469,109	473,358	Unique
chicken:300K	9	melopsittacus_undulatus	803,621	809,515	Unique
chicken:300K	9	Passeriformes	905,234	911,862	Unique
chicken:300K	9	anas_platyrhynchos	1,707,342	1,731,126	Unique
chicken:300K	9	calypte_anna	1,789,034	1,798,054	Unique
chicken:300K	9	picoides_pubescens	2,002,526	2,011,901	Unique
chicken:300K	9	boa_constrictor	2,191,386	2,211,032	Unique
chicken:300K	9	chicken	2,892,620	2,958,453	Unique
chicken:300K	9	boa_constrictor	3,578,705	3,615,886	Unique
chicken:300K	9	struthio_camelus	3,893,908	3,897,365	Unique
chicken:300K	9	chicken_turkey_duck	4,322,904	4,324,027	Unique
chicken:300K	9	chicken_turkey	4,713,046	4,717,342	Unique
chicken:300K	9	chaetura_pelagica	5,588,899	5,599,095	Unique
chicken:300K	9	anolis_carolinensis	5,916,909	5,979,705	Unique
chicken:300K	9	anolis_carolinensis	7,037,589	7,060,700	Unique
chicken:300K	9	aptenodytes_forsteri	7,595,227	7,598,513	Unique
chicken:300K	9	nipponia_nippon	8,372,133	8,374,940	Unique
chicken:300K	9	calypte_anna	8,423,705	8,425,709	Unique
chicken:300K	9	melopsittacus_undulatus:calypte_anna	8,751,134	8,754,782	Reuse
chicken:300K	9	calypte_anna:melopsittacus_undulatus	8,751,134	8,754,782	Reuse
chicken:300K	9	picoides_pubescens	9,034,844	9,077,937	Unique
chicken:300K	9	calypte_anna	9,248,220	9,259,023	Unique

chicken:300K	9	struthio_camelus:picoides_pubescens	9,516,293	9,533,926	Reuse
chicken:300K	9	picoides_pubescens:struthio_camelus	9,516,293	9,533,926	Reuse
chicken:300K	9	picoides_pubescens	10,035,640	10,040,961	Unique
chicken:300K	9	nipponia_nippon	10,207,876	10,211,038	Unique
chicken:300K	9	meleagris_gallopavo	10,358,255	10,359,913	Unique
chicken:300K	9	anolis_carolinensis:meleagris_gallopavo	10,731,820	10,732,738	Reuse
chicken:300K	9	meleagris_gallopavo:anolis_carolinensis	10,731,820	10,732,738	Reuse
chicken:300K	9	anolis_carolinensis	11,342,619	11,353,663	Unique
chicken:300K	9	birds_crocs	11,939,732	11,941,765	Unique
chicken:300K	9	melopsittacus_undulatus	12,503,503	12,717,365	Unique
chicken:300K	9	melopsittacus_undulatus	13,441,937	13,445,979	Unique
chicken:300K	9	columba_livia	15,160,944	15,165,067	Unique
chicken:300K	9	aptenodytes_forsteri	18,887,314	18,890,092	Unique
chicken:300K	9	columba_livia	19,340,917	19,343,880	Unique
chicken:300K	9	picoides_pubescens	20,143,957	20,239,721	Unique
chicken:300K	9	melopsittacus_undulatus	20,310,029	20,542,557	Unique
chicken:300K	10	egret_ibis:columba_livia	1,838,259	2,135,524	Reuse
chicken:300K	10	columba_livia:egret_ibis	1,838,259	2,135,524	Reuse
chicken:300K	10	boa_constrictor	2,154,396	2,156,924	Unique
chicken:300K	10	falco_peregrinus	2,454,559	2,455,512	Unique
chicken:300K	10	struthio_camelus	2,479,255	2,482,284	Unique
chicken:300K	10	boa_constrictor	3,177,480	3,335,685	Unique
chicken:300K	10	columba_livia	3,528,267	3,535,515	Unique
chicken:300K	10	mgfinch_finch	3,606,688	3,614,022	Unique
chicken:300K	10	columba_livia	4,539,435	4,542,050	Unique
chicken:300K	10	picoides_pubescens	4,719,159	4,723,126	Unique
chicken:300K	10	geospiza_fortis	5,130,398	5,131,698	Unique
chicken:300K	10	birds_crocs_turtles:chinese_alligator	6,084,476	6,142,351	Reuse
chicken:300K	10	chinese_alligator:birds_crocs_turtles	6,084,476	6,142,351	Reuse
chicken:300K	10	melopsittacus_undulatus	6,299,680	6,302,508	Unique
chicken:300K	10	corvus_brachyrhynchos:columba_livia	7,420,438	7,420,666	Reuse
chicken:300K	10	columba_livia:corvus_brachyrhynchos	7,420,438	7,420,666	Reuse
chicken:300K	10	cuculus_canorus	7,520,590	7,526,342	Unique
chicken:300K	10	chrysemys_picta	7,560,856	7,607,998	Unique
chicken:300K	10	melopsittacus_undulatus	9,634,831	9,640,376	Unique
chicken:300K	10	chrysemys_picta	10,110,317	10,126,178	Unique
chicken:300K	10	anas_platyrhynchos	10,224,248	10,225,564	Unique
chicken:300K	10	picoides_pubescens	11,157,298	11,165,493	Unique
chicken:300K	10	boa_constrictor	13,640,138	13,702,296	Unique
chicken:300K	10	boa_constrictor	14,416,785	14,476,395	Unique
chicken:300K	10	pygoscelis_adeliae	15,569,181	15,571,720	Unique
chicken:300K	10	aptenodytes_forsteri	16,099,850	16,101,191	Unique
chicken:300K	10	boa_constrictor	16,376,918	16,403,220	Unique
chicken:300K	10	picoides_pubescens	17,149,686	17,157,759	Unique
chicken:300K	10	columba_livia	17,204,679	17,205,848	Unique
chicken:300K	10	boa_constrictor	17,512,766	17,520,124	Unique
chicken:300K	10	chrysemys_picta	18,068,255	18,076,445	Unique
chicken:300K	10	meleagris_gallopavo	19,011,590	19,036,315	Unique
chicken:300K	11	falco_peregrinus	847,165	851,802	Unique
chicken:300K	11	melopsittacus_undulatus	852,864	862,092	Unique
chicken:300K	11	melopsittacus_undulatus	1,456,402	1,461,750	Unique
chicken:300K	11	falco_peregrinus:cuculus_canorus	1,638,019	1,639,678	Reuse
chicken:300K	11	cuculus_canorus:falco_peregrinus	1,638,019	1,639,678	Reuse
chicken:300K	11	nipponia_nippon	1,915,188	1,918,551	Unique
chicken:300K	11	chicken	2,655,301	2,701,736	Unique
chicken:300K	11	boa_constrictor	3,004,213	3,034,170	Unique
chicken:300K	11	columba_livia	3,160,639	3,163,523	Unique
chicken:300K	11	pygoscelis_adeliae	5,711,060	5,714,402	Unique
chicken:300K	11	taeniopygia_guttata	5,982,285	5,993,811	Unique
chicken:300K	11	anolis_carolinensis	6,784,532	6,859,319	Unique
chicken:300K	11	cuculus_canorus:melopsittacus_undulatus	6,904,231	6,959,872	Reuse
chicken:300K	11	melopsittacus_undulatus:cuculus_canorus	6,904,231	6,959,872	Reuse
chicken:300K	11	manacus_vitellinus	7,217,517	7,360,146	Unique
chicken:300K	11	melopsittacus_undulatus	7,365,140	7,373,086	Unique
chicken:300K	11	melopsittacus_undulatus	7,847,105	7,848,961	Unique
chicken:300K	11	charadrius_vociferus:ophisthocomus_hoazin	9,333,607	9,339,798	Reuse

chicken:300K	11	ophisthocomus_hoazin:charadrius_vociferus	9,333,607	9,339,798	Reuse
chicken:300K	11	anas_platyrhynchos	9,422,811	9,426,047	Unique
chicken:300K	11	egretta_garzetta	9,470,672	9,473,804	Unique
chicken:300K	11	boa_constrictor	9,583,258	9,696,069	Unique
chicken:300K	11	cuculus_canorus	10,782,151	10,789,126	Unique
chicken:300K	11	anas_platyrhynchos:taeniopygia_guttata	11,966,948	11,967,127	Reuse
chicken:300K	11	taeniopygia_guttata:anas_platyrhynchos	11,966,948	11,967,127	Reuse
chicken:300K	11	chrysemys_picta	12,156,113	12,180,667	Unique
chicken:300K	11	anolis_carolinensis	12,404,420	12,429,749	Unique
chicken:300K	11	Passeriformes	13,422,171	13,521,153	Unique
chicken:300K	11	pygoscelis_adeliae	13,579,131	13,581,776	Unique
chicken:300K	11	anolis_carolinensis	13,986,639	14,000,710	Unique
chicken:300K	11	columba_livia	14,586,082	14,586,950	Unique
chicken:300K	11	picoides_pubescens	14,854,680	14,860,828	Unique
chicken:300K	11	ophisthocomus_hoazin	16,239,001	16,239,773	Unique
chicken:300K	11	taeniopygia_guttata	17,088,803	17,104,352	Unique
chicken:300K	11	melopsittacus_undulatus	17,903,320	18,372,530	Unique
chicken:300K	11	chrysemys_picta	18,660,496	18,667,801	Unique
chicken:300K	11	chaetura_pelagica	18,893,009	18,896,674	Unique
chicken:300K	12	struthio_camelus	664,445	668,816	Unique
chicken:300K	12	meleagris_gallopavo	955,085	1,122,939	Unique
chicken:300K	12	taeniopygia_guttata	1,197,274	1,200,040	Unique
chicken:300K	12	chrysemys_picta:struthio_camelus	1,705,129	1,707,655	Reuse
chicken:300K	12	struthio_camelus:chrysemys_picta	1,705,129	1,707,655	Reuse
chicken:300K	12	g2	3,233,282	3,241,444	Unique
chicken:300K	12	cuculus_canorus	4,328,846	4,350,745	Unique
chicken:300K	12	columba_livia:falco_peregrinus	4,375,410	4,379,409	Reuse
chicken:300K	12	falco_peregrinus:columba_livia	4,375,410	4,379,409	Reuse
chicken:300K	12	picoides_pubescens	5,094,613	5,210,566	Unique
chicken:300K	12	anolis_carolinensis	5,449,787	5,722,957	Unique
chicken:300K	12	egretta_garzetta	5,735,062	5,735,719	Unique
chicken:300K	12	calypte_anna	5,877,180	5,893,702	Unique
chicken:300K	12	egretta_garzetta	6,087,991	6,092,567	Unique
chicken:300K	12	anas_platyrhynchos:opossum	8,582,113	8,597,309	Reuse
chicken:300K	12	opossum:anas_platyrhynchos	8,582,113	8,597,309	Reuse
chicken:300K	12	melopsittacus_undulatus:opossum:struthio_camelus	9,086,320	9,092,642	Reuse
chicken:300K	12	opossum:struthio_camelus:melopsittacus_undulatus	9,086,320	9,092,642	Reuse
chicken:300K	12	struthio_camelus:melopsittacus_undulatus:opossum	9,086,320	9,092,642	Reuse
chicken:300K	12	picoides_pubescens	11,227,412	11,231,272	Unique
chicken:300K	12	birds	11,244,729	11,253,382	Unique
chicken:300K	12	melopsittacus_undulatus	11,551,029	11,683,198	Unique
chicken:300K	12	cuculus_canorus	11,828,105	11,830,688	Unique
chicken:300K	12	chrysemys_picta	11,979,190	11,987,050	Unique
chicken:300K	12	anolis_carolinensis	12,554,291	12,606,146	Unique
chicken:300K	12	struthio_camelus	12,933,015	12,936,217	Unique
chicken:300K	12	melopsittacus_undulatus	13,605,943	13,607,116	Unique
chicken:300K	12	pygoscelis_adeliae:chinese_alligator	14,172,195	14,175,795	Reuse
chicken:300K	12	chinese_alligator:pygoscelis_adeliae	14,172,195	14,175,795	Reuse
chicken:300K	12	chaetura_pelagica:anolis_carolinensis	14,330,677	14,336,569	Reuse
chicken:300K	12	anolis_carolinensis:chaetura_pelagica	14,330,677	14,336,569	Reuse
chicken:300K	12	struthio_camelus	15,305,398	15,310,490	Unique
chicken:300K	12	aptenodytes_forsteri	16,159,856	16,160,615	Unique
chicken:300K	12	picoides_pubescens	16,751,878	16,755,561	Unique
chicken:300K	12	chrysemys_picta	17,432,270	17,449,746	Unique
chicken:300K	12	anolis_carolinensis	19,072,030	19,301,606	Unique
chicken:300K	13	birds_crocs_turtles	413,196	421,889	Unique
chicken:300K	13	anolis_carolinensis:columba_livia	1,098,736	1,111,239	Reuse
chicken:300K	13	columba_livia:anolis_carolinensis	1,098,736	1,111,239	Reuse
chicken:300K	13	columba_livia	2,655,460	2,658,550	Unique
chicken:300K	13	calypte_anna	3,514,739	3,533,688	Unique
chicken:300K	13	calypte_anna:columba_livia	4,070,726	4,095,041	Reuse
chicken:300K	13	columba_livia:calypte_anna	4,070,726	4,095,041	Reuse
chicken:300K	13	melopsittacus_undulatus:picoides_pubescens	6,042,883	6,046,713	Reuse
chicken:300K	13	picoides_pubescens:melopsittacus_undulatus	6,042,883	6,046,713	Reuse
chicken:300K	13	manacus_vitellinus	6,082,360	6,117,838	Unique
chicken:300K	13	anolis_carolinensis	6,828,576	6,868,941	Unique

chicken:300K	13	Passeriformes	7,174,015	7,179,113	Unique
chicken:300K	13	anolis_carolinensis:calypte_anna	7,469,700	7,476,414	Reuse
chicken:300K	13	calypte_anna:anolis_carolinensis	7,469,700	7,476,414	Reuse
chicken:300K	13	columba_livia	7,515,964	7,720,606	Unique
chicken:300K	13	struthio_camelus	7,767,021	7,768,547	Unique
chicken:300K	13	anas_platyrhynchos	7,919,172	7,922,917	Unique
chicken:300K	13	chinese_alligator:anolis_carolinensis	8,632,070	8,636,757	Reuse
chicken:300K	13	anolis_carolinensis:chinese_alligator	8,632,070	8,636,757	Reuse
chicken:300K	13	melopsittacus_undulatus	10,816,294	10,817,247	Unique
chicken:300K	13	chrysemys_picta	11,960,636	11,971,043	Unique
chicken:300K	13	struthio_camelus	12,462,337	12,467,832	Unique
chicken:300K	13	anolis_carolinensis	13,847,048	14,316,699	Unique
chicken:300K	13	anolis_carolinensis	15,098,457	15,106,937	Unique
chicken:300K	13	chrysemys_picta	15,330,416	15,337,387	Unique
chicken:300K	13	picoides_pubescens	15,373,900	15,386,157	Unique
chicken:300K	13	egretta_garzetta	15,865,413	15,866,065	Unique
chicken:300K	13	cuculus_canorus	15,966,691	15,973,874	Unique
chicken:300K	13	columba_livia	16,162,870	16,166,111	Unique
chicken:300K	13	pygoscelis_adeliae	16,331,160	16,335,126	Unique
chicken:300K	13	Passeriformes	16,445,939	16,449,308	Unique
chicken:300K	13	cuculus_canorus	16,546,835	16,553,197	Unique
chicken:300K	13	falco_peregrinus	16,561,057	16,583,412	Unique
chicken:300K	13	chinese_alligator:anolis_carolinensis	16,905,043	16,982,463	Reuse
chicken:300K	13	anolis_carolinensis:chinese_alligator	16,905,043	16,982,463	Reuse
chicken:300K	14	chicken_turkey	937,476	938,425	Unique
chicken:300K	14	boa_constrictor	2,257,536	2,262,242	Unique
chicken:300K	14	ophisthocomus_hoazin	2,488,620	2,491,297	Unique
chicken:300K	14	boa_constrictor	4,648,820	4,665,036	Unique
chicken:300K	14	boa_constrictor:egretta_garzetta	5,544,062	5,545,003	Reuse
chicken:300K	14	egretta_garzetta:boa_constrictor	5,544,062	5,545,003	Reuse
chicken:300K	14	boa_constrictor	5,912,054	5,955,081	Unique
chicken:300K	14	columba_livia	6,074,132	6,209,403	Unique
chicken:300K	14	corvus_brachyrhynchos	7,260,399	7,262,151	Unique
chicken:300K	14	chicken_turkey	7,458,779	7,459,817	Unique
chicken:300K	14	taeniopygia_guttata	8,319,574	8,323,624	Unique
chicken:300K	14	chicken_turkey_duck	8,900,871	8,901,116	Unique
chicken:300K	14	columba_livia	9,806,105	9,822,187	Unique
chicken:300K	14	anas_platyrhynchos	10,013,943	10,015,219	Unique
chicken:300K	14	calypte_anna	10,111,015	10,137,040	Unique
chicken:300K	14	charadrius_vociferus	10,702,958	10,723,508	Unique
chicken:300K	14	melopsittacus_undulatus	11,443,743	11,445,309	Unique
chicken:300K	14	anas_platyrhynchos	11,771,133	11,783,564	Unique
chicken:300K	14	egretta_garzetta:struthio_camelus	11,784,001	11,784,802	Reuse
chicken:300K	14	struthio_camelus:egretta_garzetta	11,784,001	11,784,802	Reuse
chicken:300K	14	columba_livia	12,236,837	12,238,885	Unique
chicken:300K	14	nipponia_nippon	12,301,965	12,306,775	Unique
chicken:300K	14	melopsittacus_undulatus	12,685,505	12,732,885	Unique
chicken:300K	14	chicken	13,622,820	13,672,177	Unique
chicken:300K	14	meleagris_gallopavo:columba_livia	14,294,380	14,305,879	Reuse
chicken:300K	14	columba_livia:meleagris_gallopavo	14,294,380	14,305,879	Reuse
chicken:300K	15	egretta_garzetta	460,592	463,395	Unique
chicken:300K	15	pygoscelis_adeliae:opossum	528,053	539,431	Reuse
chicken:300K	15	opossum:pygoscelis_adeliae	528,053	539,431	Reuse
chicken:300K	15	mgfinch_finch_crow	2,705,003	2,710,900	Unique
chicken:300K	15	melopsittacus_undulatus	3,343,726	3,353,807	Unique
chicken:300K	15	calypte_anna	3,465,155	3,479,161	Unique
chicken:300K	15	anas_platyrhynchos	4,411,747	4,506,261	Unique
chicken:300K	15	chinese_alligator	5,252,656	5,264,122	Unique
chicken:300K	15	anolis_carolinensis	5,427,638	5,457,578	Unique
chicken:300K	15	egretta_garzetta	5,592,766	5,596,355	Unique
chicken:300K	15	chinese_alligator:falco_peregrinus	6,833,532	6,924,047	Reuse
chicken:300K	15	falco_peregrinus:chinese_alligator	6,833,532	6,924,047	Reuse
chicken:300K	15	chicken_turkey	7,846,314	7,848,556	Unique
chicken:300K	15	picoides_pubescens	8,421,611	8,430,784	Unique
chicken:300K	15	anolis_carolinensis:opossum	9,093,119	9,151,715	Reuse
chicken:300K	15	opossum:anolis_carolinensis	9,093,119	9,151,715	Reuse

chicken:300K	15	cuculus_canorus:picoides_pubescens	9,254,385	9,257,275	Reuse
chicken:300K	15	picoides_pubescens:cuculus_canorus	9,254,385	9,257,275	Reuse
chicken:300K	15	chicken_turkey	9,909,430	9,910,262	Unique
chicken:300K	15	struthio_camelus	10,487,534	10,591,127	Unique
chicken:300K	15	birds	11,176,022	11,201,829	Unique
chicken:300K	15	cuculus_canorus	12,206,972	12,215,638	Unique
chicken:300K	17	melopsittacus_undulatus	516,891	519,359	Unique
chicken:300K	17	aptenodytes_forsteri	872,533	874,379	Unique
chicken:300K	17	chinese_alligator:opossum	1,026,618	1,053,835	Reuse
chicken:300K	17	opossum:chinese_alligator	1,026,618	1,053,835	Reuse
chicken:300K	17	chinese_alligator:melopsittacus_undulatus	1,759,025	1,837,988	Reuse
chicken:300K	17	melopsittacus_undulatus:chinese_alligator	1,759,025	1,837,988	Reuse
chicken:300K	17	calypte_anna	2,871,262	2,877,534	Unique
chicken:300K	17	anas_platyrhynchos	4,993,187	4,994,737	Unique
chicken:300K	17	boa_constrictor	5,189,252	5,591,546	Unique
chicken:300K	17	calypte_anna	6,169,421	6,173,610	Unique
chicken:300K	17	melopsittacus_undulatus	6,770,260	6,773,186	Unique
chicken:300K	17	anas_platyrhynchos	7,716,805	7,720,537	Unique
chicken:300K	17	meleagris_gallopavo	8,016,445	8,022,259	Unique
chicken:300K	17	calypte_anna	8,306,975	8,319,100	Unique
chicken:300K	17	meleagris_gallopavo	8,352,305	8,357,724	Unique
chicken:300K	17	chrysemys_picta	9,200,662	9,208,405	Unique
chicken:300K	17	boa_constrictor	9,518,089	9,526,306	Unique
chicken:300K	18	birds_crocs_turtles	1,000,267	1,015,247	Unique
chicken:300K	18	melopsittacus_undulatus	2,197,952	2,212,185	Unique
chicken:300K	18	cuculus_canorus	2,937,327	2,972,724	Unique
chicken:300K	18	struthio_camelus:anas_platyrhynchos	2,977,071	2,978,963	Reuse
chicken:300K	18	anas_platyrhynchos:struthio_camelus	2,977,071	2,978,963	Reuse
chicken:300K	18	boa_constrictor:anolis_carolinensis:chaetura_pelagica	3,331,503	3,345,661	Reuse
chicken:300K	18	anolis_carolinensis:chaetura_pelagica:boa_constrictor	3,331,503	3,345,661	Reuse
chicken:300K	18	chaetura_pelagica:boa_constrictor:anolis_carolinensis	3,331,503	3,345,661	Reuse
chicken:300K	18	falco_peregrinus	4,435,889	4,439,358	Unique
chicken:300K	18	chicken	5,035,833	5,038,911	Unique
chicken:300K	18	chrysemys_picta	5,678,684	5,683,509	Unique
chicken:300K	18	columba_livia	6,003,591	6,012,147	Unique
chicken:300K	18	calypte_anna	6,249,462	6,255,483	Unique
chicken:300K	18	taeniopygia_guttata	6,541,531	6,543,304	Unique
chicken:300K	18	birds_crocs	6,722,599	6,731,131	Unique
chicken:300K	18	anas_platyrhynchos	6,961,049	6,965,328	Unique
chicken:300K	18	taeniopygia_guttata	8,250,097	8,460,549	Unique
chicken:300K	18	anolis_carolinensis	9,189,826	9,244,180	Unique
chicken:300K	18	chicken	10,250,901	10,252,269	Unique
chicken:300K	18	picoides_pubescens:opossum	10,678,959	10,705,566	Reuse
chicken:300K	18	opossum:picoides_pubescens	10,678,959	10,705,566	Reuse
chicken:300K	19	boa_constrictor	848,670	993,924	Unique
chicken:300K	19	calypte_anna	1,332,504	1,341,119	Unique
chicken:300K	19	chrysemys_picta	2,451,348	2,463,993	Unique
chicken:300K	19	nipponia_nippon	2,615,152	2,617,239	Unique
chicken:300K	19	cuculus_canorus	3,131,482	3,134,678	Unique
chicken:300K	19	melopsittacus_undulatus	3,410,958	3,416,346	Unique
chicken:300K	19	calypte_anna	3,501,969	3,506,751	Unique
chicken:300K	19	boa_constrictor	4,171,937	4,183,215	Unique
chicken:300K	19	nipponia_nippon	4,793,009	4,798,436	Unique
chicken:300K	19	melopsittacus_undulatus	5,017,036	5,021,729	Unique
chicken:300K	19	calypte_anna	5,337,152	5,338,106	Unique
chicken:300K	19	chinese_alligator:opossum	5,623,045	5,627,633	Reuse
chicken:300K	19	opossum:chinese_alligator	5,623,045	5,627,633	Reuse
chicken:300K	19	melopsittacus_undulatus	5,745,795	5,950,769	Unique
chicken:300K	19	chinese_alligator:opossum	6,059,160	6,061,955	Reuse
chicken:300K	19	opossum:chinese_alligator	6,059,160	6,061,955	Reuse
chicken:300K	19	picoides_pubescens	6,306,946	6,313,159	Unique
chicken:300K	19	boa_constrictor:melopsittacus_undulatus	6,616,426	6,625,487	Reuse
chicken:300K	19	melopsittacus_undulatus:boa_constrictor	6,616,426	6,625,487	Reuse
chicken:300K	19	chrysemys_picta	7,072,415	7,074,641	Unique
chicken:300K	19	melopsittacus_undulatus	7,183,359	7,406,182	Unique
chicken:300K	19	picoides_pubescens:opossum	8,082,390	8,086,089	Reuse

chicken:300K	19	opossum:picoides_pubescens	8,082,390	8,086,089	Reuse
chicken:300K	19	melopsittacus_undulatus	8,164,070	8,168,121	Unique
chicken:300K	19	pygoscelis_adeliae	8,246,490	8,248,115	Unique
chicken:300K	19	chaetura_pelagica	9,253,270	9,297,214	Unique
chicken:300K	20	cuculus_canorus:boa_constrictor:chrysemys_picta	586,686	596,162	Reuse
chicken:300K	20	boa_constrictor:chrysemys_picta:cuculus_canorus	586,686	596,162	Reuse
chicken:300K	20	chrysemys_picta:cuculus_canorus:boa_constrictor	586,686	596,162	Reuse
chicken:300K	20	g7	1,383,033	1,401,694	Unique
chicken:300K	20	columba_livia	1,969,527	2,002,143	Unique
chicken:300K	20	meleagris_gallopavo	2,307,928	2,327,908	Unique
chicken:300K	20	calypte_anna	2,588,316	2,593,218	Unique
chicken:300K	20	falco_peregrinus	2,912,249	2,917,570	Unique
chicken:300K	20	chinese_alligator	3,382,017	3,397,302	Unique
chicken:300K	20	anolis_carolinensis	4,827,323	4,853,153	Unique
chicken:300K	20	melopsittacus_undulatus	5,360,290	5,365,107	Unique
chicken:300K	20	anolis_carolinensis	5,489,432	5,490,512	Unique
chicken:300K	20	calypte_anna	5,599,490	5,730,428	Unique
chicken:300K	20	Passeriformes	6,166,901	6,184,998	Unique
chicken:300K	20	chicken_turkey	7,001,727	7,075,274	Unique
chicken:300K	20	melopsittacus_undulatus	8,006,032	8,020,568	Unique
chicken:300K	20	falco_peregrinus	8,748,268	9,173,608	Unique
chicken:300K	20	chicken_turkey_duck	11,104,293	11,105,591	Unique
chicken:300K	20	struthio_camelus	11,594,178	11,598,067	Unique
chicken:300K	20	charadrius_vociferus	11,924,949	11,925,766	Unique
chicken:300K	20	chrysemys_picta	12,080,537	12,086,867	Unique
chicken:300K	20	chrysemys_picta	13,545,923	13,725,161	Unique
chicken:300K	21	chrysemys_picta	630,979	635,629	Unique
chicken:300K	21	melopsittacus_undulatus	1,372,775	1,376,469	Unique
chicken:300K	21	calypte_anna	1,410,962	1,411,557	Unique
chicken:300K	21	picoides_pubescens:chrysemys_picta	1,746,111	1,746,443	Reuse
chicken:300K	21	chrysemys_picta:picoides_pubescens	1,746,111	1,746,443	Reuse
chicken:300K	21	melopsittacus_undulatus	1,798,827	1,971,747	Unique
chicken:300K	21	manacus_vitellinus	1,973,004	1,975,004	Unique
chicken:300K	21	anas_platyrhynchos	2,040,160	2,069,200	Unique
chicken:300K	21	chinese_alligator	2,069,546	2,083,439	Unique
chicken:300K	21	taeniopygia_guttata	2,446,661	2,451,283	Unique
chicken:300K	21	calypte_anna	2,510,908	2,526,321	Unique
chicken:300K	21	aptenodytes_forsteri	3,112,908	3,114,261	Unique
chicken:300K	21	calypte_anna	3,446,651	3,456,223	Unique
chicken:300K	21	chicken_turkey	4,201,222	4,202,505	Unique
chicken:300K	21	manacus_vitellinus	4,854,797	4,859,960	Unique
chicken:300K	21	struthio_camelus	4,923,548	4,925,686	Unique
chicken:300K	21	mgfinch_finch_crow	4,942,710	4,943,208	Unique
chicken:300K	21	chrysemys_picta	4,968,374	4,987,906	Unique
chicken:300K	21	melopsittacus_undulatus	5,099,035	5,101,452	Unique
chicken:300K	21	melopsittacus_undulatus	5,485,420	5,493,585	Unique
chicken:300K	21	chicken_turkey	5,833,290	5,834,593	Unique
chicken:300K	21	calypte_anna	6,401,917	6,436,320	Unique
chicken:300K	22	g3	453,912	456,227	Unique
chicken:300K	22	chrysemys_picta:chinese_alligator	1,690,414	1,842,704	Reuse
chicken:300K	22	chinese_alligator:chrysemys_picta	1,690,414	1,842,704	Reuse
chicken:300K	22	falco_peregrinus:opossum	2,750,346	2,783,488	Reuse
chicken:300K	22	opossum:falco_peregrinus	2,750,346	2,783,488	Reuse
chicken:300K	22	melopsittacus_undulatus	3,300,388	3,507,000	Unique
chicken:300K	23	calypte_anna	556,147	565,853	Unique
chicken:300K	23	chicken_turkey_duck	1,763,319	1,777,800	Unique
chicken:300K	23	boa_constrictor	3,725,702	4,300,110	Unique
chicken:300K	23	chrysemys_picta:taeniopygia_guttata	5,022,027	5,034,081	Reuse
chicken:300K	23	taeniopygia_guttata:chrysemys_picta	5,022,027	5,034,081	Reuse
chicken:300K	24	taeniopygia_guttata	655,637	661,276	Unique
chicken:300K	24	egretta_garzetta	1,138,903	1,223,435	Unique
chicken:300K	24	anolis_carolinensis	1,409,081	1,418,698	Unique
chicken:300K	24	mgfinch_finch_crow	1,587,957	1,594,703	Unique
chicken:300K	24	manacus_vitellinus	1,791,493	1,794,698	Unique
chicken:300K	24	struthio_camelus	1,799,944	1,803,933	Unique
chicken:300K	24	chrysemys_picta	2,110,625	2,117,486	Unique

chicken:300K	24	anolis_carolinensis	2,471,399	2,488,528	Unique
chicken:300K	24	anas_platyrhynchos	2,790,365	2,792,800	Unique
chicken:300K	24	melopsittacus_undulatus:opossum	2,826,835	2,832,754	Reuse
chicken:300K	24	opossum:melopsittacus_undulatus	2,826,835	2,832,754	Reuse
chicken:300K	24	taeniopygia_guttata	2,899,692	2,907,440	Unique
chicken:300K	24	anas_platyrhynchos:egretta_garzetta	3,176,974	3,264,182	Reuse
chicken:300K	24	egretta_garzetta:anas_platyrhynchos	3,176,974	3,264,182	Reuse
chicken:300K	24	columba_livia	3,782,241	3,789,548	Unique
chicken:300K	24	chicken_turkey_duck	4,335,317	4,338,440	Unique
chicken:300K	24	anas_platyrhynchos	4,368,901	4,372,600	Unique
chicken:300K	24	chrysemys_picta	4,863,560	4,871,337	Unique
chicken:300K	24	boa_constrictor	5,267,389	5,269,111	Unique
chicken:300K	25	boa_constrictor:anas_platyrhynchos	489,197	513,126	Reuse
chicken:300K	25	anas_platyrhynchos:boa_constrictor	489,197	513,126	Reuse
chicken:300K	26	columba_livia	1,056,366	1,062,734	Unique
chicken:300K	26	non_galloanserae	1,618,791	1,620,819	Unique
chicken:300K	26	anolis_carolinensis	2,033,357	2,058,871	Unique
chicken:300K	26	anas_platyrhynchos:columba_livia	2,205,379	2,227,503	Reuse
chicken:300K	26	columba_livia:anas_platyrhynchos	2,205,379	2,227,503	Reuse
chicken:300K	26	anolis_carolinensis	2,958,696	2,976,621	Unique
chicken:300K	26	columba_livia	3,183,469	3,418,980	Unique
chicken:300K	26	chicken_turkey	4,017,070	4,020,457	Unique
chicken:300K	26	anas_platyrhynchos	4,349,541	4,366,515	Unique
chicken:300K	26	anolis_carolinensis:anas_platyrhynchos	4,674,161	4,676,644	Reuse
chicken:300K	26	anas_platyrhynchos:anolis_carolinensis	4,674,161	4,676,644	Reuse
chicken:300K	27	taeniopygia_guttata	837,886	1,048,658	Unique
chicken:300K	27	taeniopygia_guttata	1,549,207	1,590,407	Unique
chicken:300K	27	struthio_camelus:columba_livia	1,662,631	1,663,045	Reuse
chicken:300K	27	columba_livia:struthio_camelus	1,662,631	1,663,045	Reuse
chicken:300K	27	cuculus_canorus	1,693,185	1,695,062	Unique
chicken:300K	27	anas_platyrhynchos:calypte_anna	2,181,828	2,187,469	Reuse
chicken:300K	27	calypte_anna:anas_platyrhynchos	2,181,828	2,187,469	Reuse
chicken:300K	27	nipponia_nippon	2,277,023	2,278,313	Unique
chicken:300K	27	cuculus_canorus	2,298,926	2,303,220	Unique
chicken:300K	27	columba_livia	2,317,250	2,325,812	Unique
chicken:300K	27	calypte_anna	2,637,119	2,642,466	Unique
chicken:300K	27	anas_platyrhynchos	3,861,328	3,908,419	Unique
chicken:300K	27	anolis_carolinensis:boa_constrictor	4,012,023	4,123,959	Reuse
chicken:300K	27	boa_constrictor:anolis_carolinensis	4,012,023	4,123,959	Reuse
chicken:300K	28	falco_peregrinus	2,406,901	2,411,742	Unique
chicken:300K	28	falco_peregrinus	4,041,766	4,042,931	Unique
chicken:300K	28	corvus_brachyrhynchos	4,081,899	4,084,794	Unique
chicken:300K	28	columba_livia	4,183,707	4,187,436	Unique
chicken:300K	28	chrysemys_picta:chinese_alligator:opossum	4,200,683	4,209,256	Reuse
chicken:300K	28	chinese_alligator:opossum:chrysemys_picta	4,200,683	4,209,256	Reuse
chicken:300K	28	opossum:chrysemys_picta:chinese_alligator	4,200,683	4,209,256	Reuse
chicken:300K	z	chrysemys_picta:charadrius_vociferus:egretta_garzetta:falco_peregrinus:struthio_camelus	325,000	609,316	Reuse
chicken:300K	z	charadrius_vociferus:egretta_garzetta:falco_peregrinus:struthio_camelus:chrysemys_picta	325,000	609,316	Reuse
chicken:300K	z	egretta_garzetta:falco_peregrinus:struthio_camelus:chrysemys_picta:charadrius_vociferus	325,000	609,316	Reuse
chicken:300K	z	falco_peregrinus:struthio_camelus:chrysemys_picta:charadrius_vociferus:egretta_garzetta	325,000	609,316	Reuse
chicken:300K	z	struthio_camelus:chrysemys_picta:charadrius_vociferus:egretta_garzetta:falco_peregrinus	325,000	609,316	Reuse
chicken:300K	z	neoavians	979,749	984,626	Unique
chicken:300K	z	corvus_brachyrhynchos	1,382,037	1,383,613	Unique
chicken:300K	z	anolis_carolinensis	1,680,442	1,724,562	Unique
chicken:300K	z	taeniopygia_guttata	1,812,765	1,822,812	Unique
chicken:300K	z	melopsittacus_undulatus	2,272,653	2,280,566	Unique
chicken:300K	z	ophisthocomus_hoazin	4,048,204	4,054,715	Unique
chicken:300K	z	anolis_carolinensis	4,294,217	4,323,596	Unique
chicken:300K	z	chinese_alligator	4,353,096	4,377,399	Unique
chicken:300K	z	melopsittacus_undulatus	5,340,642	5,344,172	Unique
chicken:300K	z	anas_platyrhynchos	5,862,360	6,160,056	Unique
chicken:300K	z	melopsittacus_undulatus	8,914,063	9,028,447	Unique

chicken:300K	z	cuculus_canorus	9,924,640	9,927,805	Unique
chicken:300K	z	charadrius_vociferus	9,958,193	9,963,494	Unique
chicken:300K	z	cuculus_canorus	10,901,016	10,961,395	Unique
chicken:300K	z	chrysemys_picta:manacus_vitellinus	11,114,924	11,130,427	Reuse
chicken:300K	z	manacus_vitellinus:chrysemys_picta	11,114,924	11,130,427	Reuse
chicken:300K	z	chrysemys_picta	11,532,395	11,757,275	Unique
chicken:300K	z	columba_livia	12,770,794	12,776,924	Unique
chicken:300K	z	falco_peregrinus:chrysemys_picta	12,845,288	12,919,881	Reuse
chicken:300K	z	chrysemys_picta:falco_peregrinus	12,845,288	12,919,881	Reuse
chicken:300K	z	manacus_vitellinus	13,143,856	13,169,663	Unique
chicken:300K	z	columba_livia	13,183,001	13,210,325	Unique
chicken:300K	z	chrysemys_picta	16,296,065	16,312,363	Unique
chicken:300K	z	chinese_alligator:charadrius_vociferus	16,782,557	16,791,441	Reuse
chicken:300K	z	charadrius_vociferus:chinese_alligator	16,782,557	16,791,441	Reuse
chicken:300K	z	picoides_pubescens	17,404,717	17,573,855	Unique
chicken:300K	z	egretta_garzetta	17,898,245	17,903,166	Unique
chicken:300K	z	falco_peregrinus	18,478,315	18,544,132	Unique
chicken:300K	z	picoides_pubescens	18,592,547	18,594,533	Unique
chicken:300K	z	chrysemys_picta	19,248,033	19,302,284	Unique
chicken:300K	z	calypte_anna	19,335,100	19,340,788	Unique
chicken:300K	z	boa_constrictor:anolis_carolinensis	19,414,315	19,853,613	Reuse
chicken:300K	z	anolis_carolinensis:boa_constrictor	19,414,315	19,853,613	Reuse
chicken:300K	z	corvus_brachyrhynchos	20,972,795	20,981,761	Unique
chicken:300K	z	struthio_camelus	21,487,641	21,492,981	Unique
chicken:300K	z	mgfinch_finch_crow	21,600,514	21,820,648	Unique
chicken:300K	z	melopsittacus_undulatus	22,287,753	22,295,551	Unique
chicken:300K	z	cuculus_canorus	22,632,674	22,637,108	Unique
chicken:300K	z	ophisthocomus_hoazin	23,600,905	23,615,192	Unique
chicken:300K	z	taeniopygia_guttata	23,642,099	23,650,576	Unique
chicken:300K	z	neoavians	25,352,141	25,356,301	Unique
chicken:300K	z	struthio_camelus	26,888,103	26,895,919	Unique
chicken:300K	z	columba_livia:cuculus_canorus	26,996,252	27,341,257	Reuse
chicken:300K	z	cuculus_canorus:columba_livia	26,996,252	27,341,257	Reuse
chicken:300K	z	chrysemys_picta	27,673,440	27,683,327	Unique
chicken:300K	z	anolis_carolinensis	28,916,020	30,816,623	Unique
chicken:300K	z	manacus_vitellinus	31,676,732	31,690,177	Unique
chicken:300K	z	struthio_camelus	32,461,385	32,462,900	Unique
chicken:300K	z	cuculus_canorus	33,012,869	33,017,315	Unique
chicken:300K	z	anolis_carolinensis	33,064,455	33,195,926	Unique
chicken:300K	z	chaetura_pelagica	33,292,535	33,294,102	Unique
chicken:300K	z	struthio_camelus	34,804,736	35,168,928	Unique
chicken:300K	z	corvus_brachyrhynchos	35,540,785	35,545,313	Unique
chicken:300K	z	struthio_camelus	35,704,684	36,600,739	Unique
chicken:300K	z	nipponia_nippon:chaetura_pelagica	37,154,439	37,161,935	Reuse
chicken:300K	z	chaetura_pelagica:nipponia_nippon	37,154,439	37,161,935	Reuse
chicken:300K	z	Passeriformes	37,182,429	37,190,619	Unique
chicken:300K	z	columba_livia	37,194,831	37,197,546	Unique
chicken:300K	z	charadrius_vociferus	38,863,464	38,872,578	Unique
chicken:300K	z	egretta_garzetta:chrysemys_picta:cuculus_canorus	39,647,312	39,669,436	Reuse
chicken:300K	z	chrysemys_picta:cuculus_canorus:egretta_garzetta	39,647,312	39,669,436	Reuse
chicken:300K	z	cuculus_canorus:egretta_garzetta:chrysemys_picta	39,647,312	39,669,436	Reuse
chicken:300K	z	manacus_vitellinus	41,328,030	41,329,949	Unique
chicken:300K	z	anas_platyrhynchos:opossum	42,933,766	42,995,895	Reuse
chicken:300K	z	opossum:anas_platyrhynchos	42,933,766	42,995,895	Reuse
chicken:300K	z	anas_platyrhynchos:opossum:anolis_carolinensis	43,745,812	43,886,883	Reuse
chicken:300K	z	opossum:anolis_carolinensis:anas_platyrhynchos	43,745,812	43,886,883	Reuse
chicken:300K	z	anolis_carolinensis:anas_platyrhynchos:opossum	43,745,812	43,886,883	Reuse
chicken:300K	z	chrysemys_picta	44,016,009	44,025,941	Unique
chicken:300K	z	charadrius_vociferus:cuculus_canorus	45,122,815	45,128,419	Reuse
chicken:300K	z	cuculus_canorus:charadrius_vociferus	45,122,815	45,128,419	Reuse
chicken:300K	z	chicken_turkey	45,523,252	45,523,589	Unique
chicken:300K	z	mgfinch_finch	49,156,076	49,165,289	Unique
chicken:300K	z	mgfinch_finch	49,579,443	49,591,307	Unique
chicken:300K	z	ophisthocomus_hoazin	49,860,775	49,866,122	Unique
chicken:300K	z	columba_livia	50,619,010	51,067,128	Unique
chicken:300K	z	neoavians	51,481,116	51,652,509	Unique

chicken:300K	z	melopsittacus_undulatus	52,396,104	52,434,452	Unique
chicken:300K	z	egretta_garzetta	52,549,550	52,561,383	Unique
chicken:300K	z	charadrius_vociferus:columba_livia	52,766,676	52,770,272	Reuse
chicken:300K	z	columba_livia:charadrius_vociferus	52,766,676	52,770,272	Reuse
chicken:300K	z	hummingbird_swift	53,286,858	53,293,895	Unique
chicken:300K	z	anolis_carolinensis:cuculus_canorus	53,402,670	53,405,606	Reuse
chicken:300K	z	cuculus_canorus:anolis_carolinensis	53,402,670	53,405,606	Reuse
chicken:300K	z	corvus_brachyrhynchos	53,818,831	53,822,710	Unique
chicken:300K	z	chrysemys_picta	54,473,359	54,530,403	Unique
chicken:300K	z	anolis_carolinensis	54,596,800	54,761,360	Unique
chicken:300K	z	Passeriformes	54,824,372	54,824,429	Unique
chicken:300K	z	struthio_camelus	55,990,603	56,006,779	Unique
chicken:300K	z	chicken_turkey	56,821,813	56,827,515	Unique
chicken:300K	z	chrysemys_picta	58,068,459	58,080,321	Unique
chicken:300K	z	pygoscelis_adeliae	60,633,093	60,814,767	Unique
chicken:300K	z	Passeriformes	60,954,531	60,958,865	Unique
chicken:300K	z	pygoscelis_adeliae	61,186,862	61,222,814	Unique
chicken:300K	z	cuculus_canorus	63,565,651	63,567,662	Unique
chicken:300K	z	corvus_brachyrhynchos	63,655,276	63,660,650	Unique
chicken:300K	z	taeniopygia_guttata:chrysemys_picta:struthio_camelus	64,002,577	64,098,619	Reuse
chicken:300K	z	chrysemys_picta:struthio_camelus:taeniopygia_guttata	64,002,577	64,098,619	Reuse
chicken:300K	z	struthio_camelus:taeniopygia_guttata:chrysemys_picta	64,002,577	64,098,619	Reuse
chicken:300K	z	struthio_camelus	64,563,384	64,564,932	Unique
chicken:300K	z	g6	65,319,293	65,323,872	Unique
chicken:300K	z	chaetura_pelagica	65,753,630	65,849,246	Unique
chicken:300K	z	neoavians	66,941,666	66,948,009	Unique
chicken:300K	z	struthio_camelus	67,454,337	67,504,692	Unique
chicken:300K	z	struthio_camelus	68,069,168	68,075,403	Unique
chicken:300K	z	anolis_carolinensis	68,421,169	68,655,306	Unique
chicken:300K	z	aptenodytes_forsteri	69,580,942	69,584,173	Unique
chicken:300K	z	melopsittacus_undulatus	69,916,741	69,920,985	Unique
chicken:300K	z	chicken_turkey_duck	70,825,623	70,827,839	Unique
chicken:300K	z	chicken_turkey_duck	72,985,809	73,023,355	Unique
chicken:300K	z	charadrius_vociferus	78,573,617	78,575,593	Unique
chicken:300K	z	columba_livia	78,740,986	78,750,445	Unique
chicken:300K	z	chicken_turkey_duck	79,987,451	80,647,861	Unique
chicken:300K	z	chicken_turkey_duck	81,361,319	81,364,283	Unique
chicken:300K	z	geospiza_fortis	81,463,790	81,479,690	Unique
chicken:500K	1	falco_peregrinus	1,162,520	1,326,161	Unique
chicken:500K	1	anolis_carolinensis	4,281,085	4,285,281	Unique
chicken:500K	1	chrysemys_picta	4,507,338	4,516,008	Unique
chicken:500K	1	meleagris_gallopavo	5,510,025	5,513,465	Unique
chicken:500K	1	falco_peregrinus	6,029,336	6,031,512	Unique
chicken:500K	1	birds_crocs_turtles	6,075,032	6,083,842	Unique
chicken:500K	1	anolis_carolinensis:struthio_camelus	7,232,548	7,236,580	Reuse
chicken:500K	1	struthio_camelus:anolis_carolinensis	7,232,548	7,236,580	Reuse
chicken:500K	1	falco_peregrinus	7,654,682	7,915,408	Unique
chicken:500K	1	chrysemys_picta	8,347,267	8,351,964	Unique
chicken:500K	1	nipponia_nippon:chrysemys_picta:meleagris_gallopavo	8,864,711	9,224,002	Reuse
chicken:500K	1	chrysemys_picta:meleagris_gallopavo:nipponia_nippon	8,864,711	9,224,002	Reuse
chicken:500K	1	meleagris_gallopavo:nipponia_nippon:chrysemys_picta	8,864,711	9,224,002	Reuse
chicken:500K	1	falco_peregrinus	9,887,440	9,888,690	Unique
chicken:500K	1	anolis_carolinensis	9,926,144	9,951,090	Unique
chicken:500K	1	struthio_camelus	10,911,607	10,916,109	Unique
chicken:500K	1	chaetura_pelagica	12,726,035	12,729,399	Unique
chicken:500K	1	chrysemys_picta	14,766,599	14,783,639	Unique
chicken:500K	1	anolis_carolinensis	15,674,033	15,769,959	Unique
chicken:500K	1	chrysemys_picta	16,127,058	16,443,669	Unique
chicken:500K	1	melopsittacus_undulatus	18,411,634	18,414,857	Unique
chicken:500K	1	anolis_carolinensis	18,447,833	18,490,318	Unique
chicken:500K	1	struthio_camelus	19,429,651	19,433,176	Unique
chicken:500K	1	melopsittacus_undulatus	19,576,458	19,747,900	Unique
chicken:500K	1	anolis_carolinensis	19,760,133	19,877,001	Unique
chicken:500K	1	anolis_carolinensis	22,845,398	23,050,166	Unique
chicken:500K	1	anas_platyrhynchos	23,926,371	23,927,139	Unique
chicken:500K	1	python_molurus	25,012,787	25,054,385	Unique

chicken:500K	1	melopsittacus_undulatus	25,804,207	25,806,627	Unique
chicken:500K	1	pygoscelis_adeliae	26,026,802	26,031,490	Unique
chicken:500K	1	chrysemys_picta	26,537,742	27,212,312	Unique
chicken:500K	1	melopsittacus_undulatus	28,694,712	28,701,534	Unique
chicken:500K	1	aptenodytes_forsteri	29,059,748	29,062,983	Unique
chicken:500K	1	chrysemys_picta	32,420,483	32,432,940	Unique
chicken:500K	1	cuculus_canorus	33,346,065	33,628,144	Unique
chicken:500K	1	melopsittacus_undulatus:anolis_carolinensis	34,037,387	34,061,098	Reuse
chicken:500K	1	anolis_carolinensis:melopsittacus_undulatus	34,037,387	34,061,098	Reuse
chicken:500K	1	chrysemys_picta	34,668,164	34,677,307	Unique
chicken:500K	1	chinese_alligator	34,872,919	35,013,957	Unique
chicken:500K	1	Cuculiformes + Trochiliformes + Apodiformes	35,388,849	35,396,628	Unique
chicken:500K	1	anolis_carolinensis	35,772,105	35,807,731	Unique
chicken:500K	1	melopsittacus_undulatus	36,330,037	36,335,101	Unique
chicken:500K	1	chrysemys_picta	37,335,309	37,343,883	Unique
chicken:500K	1	melopsittacus_undulatus	37,370,851	37,373,049	Unique
chicken:500K	1	charadrius_vociferus	38,075,586	38,080,977	Unique
chicken:500K	1	columba_livia	38,301,854	38,369,907	Unique
chicken:500K	1	anolis_carolinensis	39,188,146	39,223,463	Unique
chicken:500K	1	corvus_brachyrhynchos	39,609,677	39,615,487	Unique
chicken:500K	1	falco_peregrinus	39,765,373	39,767,039	Unique
chicken:500K	1	anas_platyrhynchos	40,082,741	40,090,886	Unique
chicken:500K	1	meleagris_gallopavo	40,165,675	40,174,839	Unique
chicken:500K	1	nipponia_nippon	40,806,714	40,810,675	Unique
chicken:500K	1	meleagris_gallopavo	41,240,313	41,241,582	Unique
chicken:500K	1	picoides_pubescens	41,514,440	41,521,301	Unique
chicken:500K	1	calypte_anna:chrysemys_picta	42,323,195	42,364,573	Reuse
chicken:500K	1	chrysemys_picta:calypte_anna	42,323,195	42,364,573	Reuse
chicken:500K	1	melopsittacus_undulatus	44,579,230	44,581,535	Unique
chicken:500K	1	aptenodytes_forsteri	45,012,081	45,015,014	Unique
chicken:500K	1	picoides_pubescens	45,374,102	45,400,745	Unique
chicken:500K	1	calypte_anna	46,987,355	47,063,550	Unique
chicken:500K	1	melopsittacus_undulatus	47,775,553	47,812,526	Unique
chicken:500K	1	anas_platyrhynchos	47,905,042	47,906,033	Unique
chicken:500K	1	anolis_carolinensis	48,103,016	48,107,037	Unique
chicken:500K	1	meleagris_gallopavo	48,609,502	48,610,293	Unique
chicken:500K	1	birds	48,956,147	49,154,316	Unique
chicken:500K	1	anas_platyrhynchos	49,293,025	49,650,033	Unique
chicken:500K	1	chinese_alligator	50,132,260	50,162,740	Unique
chicken:500K	1	birds_crocs_turtles	50,757,319	50,995,734	Unique
chicken:500K	1	meleagris_gallopavo	51,597,899	51,598,769	Unique
chicken:500K	1	melopsittacus_undulatus	51,999,326	52,006,775	Unique
chicken:500K	1	nipponia_nippon	52,181,801	52,182,602	Unique
chicken:500K	1	pygoscelis_adeliae	52,234,403	52,239,417	Unique
chicken:500K	1	cuculus_canorus	52,836,116	52,976,300	Unique
chicken:500K	1	melopsittacus_undulatus	53,228,651	53,251,145	Unique
chicken:500K	1	columba_livia	54,633,829	54,854,430	Unique
chicken:500K	1	geospiza_fortis	54,881,392	54,891,581	Unique
chicken:500K	1	anolis_carolinensis	56,484,852	56,488,897	Unique
chicken:500K	1	geospiza_fortis	56,527,911	56,530,955	Unique
chicken:500K	1	corvus_brachyrhynchos	56,534,882	56,538,791	Unique
chicken:500K	1	melopsittacus_undulatus	58,166,174	58,171,797	Unique
chicken:500K	1	picoides_pubescens	59,805,225	59,839,027	Unique
chicken:500K	1	passeroidea + corvoidea	60,038,442	60,044,624	Unique
chicken:500K	1	aptenodytes_forsteri	60,568,105	60,570,116	Unique
chicken:500K	1	calypte_anna	61,880,377	62,234,790	Unique
chicken:500K	1	columba_livia	62,266,385	62,311,473	Unique
chicken:500K	1	melopsittacus_undulatus	62,315,860	62,324,002	Unique
chicken:500K	1	anolis_carolinensis	62,597,420	62,885,782	Unique
chicken:500K	1	anolis_carolinensis	63,948,060	63,979,107	Unique
chicken:500K	1	chrysemys_picta	64,818,138	64,834,488	Unique
chicken:500K	1	anas_platyrhynchos	65,315,081	65,317,342	Unique
chicken:500K	1	taeniopygia_guttata	65,336,665	65,345,112	Unique
chicken:500K	1	meleagris_gallopavo	66,340,921	66,531,391	Unique
chicken:500K	1	passeriformes	66,927,502	66,949,983	Unique
chicken:500K	1	chaetura_pelagica	67,134,060	67,185,805	Unique

chicken:500K	1	picoides_pubescens	67,327,395	67,332,193	Unique
chicken:500K	1	birds_crocs_turtles	67,388,926	67,562,411	Unique
chicken:500K	1	chaetura_pelagica	68,440,857	68,445,290	Unique
chicken:500K	1	manacus_vitellinus	68,547,210	68,555,948	Unique
chicken:500K	1	meleagris_gallopavo	68,600,040	68,886,196	Unique
chicken:500K	1	melopsittacus_undulatus:chaetura_pelagica	69,211,222	69,212,497	Reuse
chicken:500K	1	chaetura_pelagica:melopsittacus_undulatus	69,211,222	69,212,497	Reuse
chicken:500K	1	meleagris_gallopavo	69,406,978	69,412,807	Unique
chicken:500K	1	chrysemys_picta	69,566,647	69,787,779	Unique
chicken:500K	1	chaetura_pelagica	69,803,287	69,814,376	Unique
chicken:500K	1	meleagris_gallopavo	69,951,079	69,953,442	Unique
chicken:500K	1	melopsittacus_undulatus	70,069,637	70,078,216	Unique
chicken:500K	1	taeniopygia_guttata	71,782,945	71,828,445	Unique
chicken:500K	1	chrysemys_picta	71,861,324	71,872,803	Unique
chicken:500K	1	chaetura_pelagica	71,979,442	72,280,429	Unique
chicken:500K	1	meleagris_gallopavo	72,412,203	72,963,977	Unique
chicken:500K	1	galliformes	73,159,165	73,168,005	Unique
chicken:500K	1	meleagris_gallopavo	73,847,983	74,057,896	Unique
chicken:500K	1	galliformes	74,630,982	74,632,558	Unique
chicken:500K	1	columba_livia	75,208,622	75,249,872	Unique
chicken:500K	1	galliformes	75,870,953	75,949,704	Unique
chicken:500K	1	melopsittacus_undulatus	76,924,726	76,958,973	Unique
chicken:500K	1	galliformes	77,734,671	77,950,129	Unique
chicken:500K	1	struthio_camelus	78,464,720	78,465,944	Unique
chicken:500K	1	python_molurus	79,606,378	79,608,088	Unique
chicken:500K	1	aptenodytes_forsteri	82,024,764	82,026,502	Unique
chicken:500K	1	chaetura_pelagica:opossum:falco_peregrinus	83,538,268	83,564,175	Reuse
chicken:500K	1	opossum:falco_peregrinus:chaetura_pelagica	83,538,268	83,564,175	Reuse
chicken:500K	1	falco_peregrinus:chaetura_pelagica:opossum	83,538,268	83,564,175	Reuse
chicken:500K	1	taeniopygia_guttata	83,916,726	83,917,179	Unique
chicken:500K	1	passeroidea + corvoidea	83,977,286	83,984,032	Unique
chicken:500K	1	melopsittacus_undulatus	84,993,558	86,343,158	Unique
chicken:500K	1	columba_livia:opossum	86,742,609	86,871,590	Reuse
chicken:500K	1	opossum:columba_livia	86,742,609	86,871,590	Reuse
chicken:500K	1	chaetura_pelagica	87,942,689	87,944,890	Unique
chicken:500K	1	manacus_vitellinus	88,092,859	88,101,278	Unique
chicken:500K	1	passeroidea + corvoidea	88,139,067	88,151,044	Unique
chicken:500K	1	melopsittacus_undulatus	88,716,773	89,732,493	Unique
chicken:500K	1	calypte_anna	90,906,153	90,911,569	Unique
chicken:500K	1	columba_livia	91,170,101	91,175,236	Unique
chicken:500K	1	manacus_vitellinus	92,418,689	92,422,087	Unique
chicken:500K	1	struthio_camelus	93,635,353	93,789,678	Unique
chicken:500K	1	pygoscelis_adeliae	94,168,594	94,171,061	Unique
chicken:500K	1	manacus_vitellinus	94,856,972	94,879,666	Unique
chicken:500K	1	struthio_camelus	95,024,959	95,031,570	Unique
chicken:500K	1	anolis_carolinensis	96,158,583	96,182,938	Unique
chicken:500K	1	melopsittacus_undulatus:opossum	97,207,251	97,365,487	Reuse
chicken:500K	1	opossum:melopsittacus_undulatus	97,207,251	97,365,487	Reuse
chicken:500K	1	anolis_carolinensis	97,784,831	97,856,036	Unique
chicken:500K	1	columba_livia	98,957,586	98,963,992	Unique
chicken:500K	1	chrysemys_picta:meleagris_gallopavo	99,602,540	99,934,246	Reuse
chicken:500K	1	meleagris_gallopavo:chrysemys_picta	99,602,540	99,934,246	Reuse
chicken:500K	1	meleagris_gallopavo	100,658,381	100,658,586	Unique
chicken:500K	1	Passeriformes + Psittaciformes + Falconiformes + Piciformes + Ciconiiformes + Sphenisciformes + Charadriiformes + Opisthocomiformes	102,320,517	102,323,550	Unique
chicken:500K	1	chrysemys_picta	103,204,782	103,229,251	Unique
chicken:500K	1	passeroidea + corvoidea	103,453,722	103,455,135	Unique
chicken:500K	1	meleagris_gallopavo	103,734,890	103,740,002	Unique
chicken:500K	1	pygoscelis_adeliae	103,786,999	103,788,821	Unique
chicken:500K	1	falco_peregrinus	104,195,564	104,199,261	Unique
chicken:500K	1	anolis_carolinensis	104,525,322	104,716,505	Unique
chicken:500K	1	meleagris_gallopavo	106,138,711	106,150,991	Unique
chicken:500K	1	chrysemys_picta	106,506,944	106,549,393	Unique
chicken:500K	1	anas_platyrhynchos:columba_livia	107,975,084	107,977,673	Reuse
chicken:500K	1	columba_livia:anas_platyrhynchos	107,975,084	107,977,673	Reuse
chicken:500K	1	chrysemys_picta:opossum	109,641,070	109,716,768	Reuse

chicken:500K	1	opossum:chrysemys_picta	109,641,070	109,716,768	Reuse
chicken:500K	1	corvus_brachyrhynchos	109,808,314	109,809,271	Unique
chicken:500K	1	meleagris_gallopavo:anas_platyrhynchos	111,885,484	111,887,345	Reuse
chicken:500K	1	anas_platyrhynchos:meleagris_gallopavo	111,885,484	111,887,345	Reuse
chicken:500K	1	melopsittacus_undulatus	111,939,623	111,942,334	Unique
chicken:500K	1	anas_platyrhynchos	112,706,301	112,733,100	Unique
chicken:500K	1	columba_livia	112,920,855	112,922,899	Unique
chicken:500K	1	anolis_carolinensis	113,288,049	113,743,498	Unique
chicken:500K	1	corvus_brachyrhynchos	115,159,850	115,165,845	Unique
chicken:500K	1	melopsittacus_undulatus	115,217,491	115,220,744	Unique
chicken:500K	1	anolis_carolinensis	115,802,911	116,878,343	Unique
chicken:500K	1	chrysemys_picta:nipponia_nippon	117,163,254	117,164,167	Reuse
chicken:500K	1	nipponia_nippon:chrysemys_picta	117,163,254	117,164,167	Reuse
chicken:500K	1	anas_platyrhynchos	117,818,423	117,821,824	Unique
chicken:500K	1	anas_platyrhynchos	119,240,498	119,256,036	Unique
chicken:500K	1	chrysemys_picta	120,147,406	121,097,355	Unique
chicken:500K	1	picoides_pubescens	121,246,007	121,496,985	Unique
chicken:500K	1	picoides_pubescens:meleagris_gallopavo	122,091,699	122,243,299	Reuse
chicken:500K	1	meleagris_gallopavo:picoides_pubescens	122,091,699	122,243,299	Reuse
chicken:500K	1	chrysemys_picta	124,379,007	124,402,723	Unique
chicken:500K	1	chrysemys_picta	125,451,718	125,536,504	Unique
chicken:500K	1	meleagris_gallopavo	125,783,117	126,288,559	Unique
chicken:500K	1	chrysemys_picta	127,485,646	127,578,313	Unique
chicken:500K	1	passeriformes	127,801,451	127,804,749	Unique
chicken:500K	1	falco_peregrinus	127,818,501	128,001,910	Unique
chicken:500K	1	chrysemys_picta	128,084,524	128,113,700	Unique
chicken:500K	1	chinese_alligator	129,564,984	129,870,220	Unique
chicken:500K	1	chrysemys_picta	131,024,742	131,039,327	Unique
chicken:500K	1	picoides_pubescens	131,338,446	131,820,701	Unique
chicken:500K	1	columba_livia:chaetura_pelagica	131,823,156	131,829,869	Reuse
chicken:500K	1	chaetura_pelagica:columba_livia	131,823,156	131,829,869	Reuse
chicken:500K	1	meleagris_gallopavo	131,908,115	131,911,152	Unique
chicken:500K	1	meleagris_gallopavo	132,501,353	132,504,105	Unique
chicken:500K	1	chrysemys_picta	132,770,391	133,194,678	Unique
chicken:500K	1	anolis_carolinensis	134,421,246	134,451,426	Unique
chicken:500K	1	galloanserae	137,313,100	137,315,743	Unique
chicken:500K	1	meleagris_gallopavo	137,507,947	137,513,954	Unique
chicken:500K	1	cuculus_canorus	138,068,842	138,434,054	Unique
chicken:500K	1	melopsittacus_undulatus	138,566,658	138,572,517	Unique
chicken:500K	1	meleagris_gallopavo	139,032,177	139,066,771	Unique
chicken:500K	1	galloanserae	139,331,778	139,333,713	Unique
chicken:500K	1	anolis_carolinensis	139,485,298	139,588,983	Unique
chicken:500K	1	chrysemys_picta	141,157,806	141,164,111	Unique
chicken:500K	1	chrysemys_picta	144,075,331	144,099,792	Unique
chicken:500K	1	anolis_carolinensis	144,392,892	144,525,842	Unique
chicken:500K	1	anolis_carolinensis	146,602,292	147,002,228	Unique
chicken:500K	1	chinese_alligator	147,068,497	147,077,576	Unique
chicken:500K	1	pygoscelis_adeliae	147,639,784	147,643,788	Unique
chicken:500K	1	taeniopygia_guttata	149,441,310	149,468,635	Unique
chicken:500K	1	manacus_vitellinus	149,782,988	149,810,958	Unique
chicken:500K	1	ophisthocomus_hoazin:anolis_carolinensis:anas_platyrhynchos	150,689,849	150,805,651	Reuse
chicken:500K	1	anolis_carolinensis:anas_platyrhynchos:ophisthocomus_hoazin	150,689,849	150,805,651	Reuse
chicken:500K	1	anas_platyrhynchos:ophisthocomus_hoazin:anolis_carolinensis	150,689,849	150,805,651	Reuse
chicken:500K	1	pygoscelis_adeliae	151,079,499	151,083,119	Unique
chicken:500K	1	ophisthocomus_hoazin	153,015,186	153,019,601	Unique
chicken:500K	1	cuculus_canorus:anolis_carolinensis	153,137,336	153,198,748	Reuse
chicken:500K	1	anolis_carolinensis:cuculus_canorus	153,137,336	153,198,748	Reuse
chicken:500K	1	ophisthocomus_hoazin	154,604,994	154,610,388	Unique
chicken:500K	1	anolis_carolinensis	155,532,367	155,603,409	Unique
chicken:500K	1	manacus_vitellinus	157,083,652	157,172,969	Unique
chicken:500K	1	aptenodytes_forsteri	157,928,941	157,936,327	Unique
chicken:500K	1	chrysemys_picta	158,034,167	158,057,106	Unique
chicken:500K	1	melopsittacus_undulatus	159,620,982	159,680,092	Unique
chicken:500K	1	chrysemys_picta	160,933,521	161,484,070	Unique
chicken:500K	1	meleagris_gallopavo	163,249,257	163,870,382	Unique
chicken:500K	1	aptenodytes_forsteri	167,067,659	167,069,432	Unique

chicken:500K	1	aptenodytes_forsteri	168,105,793	168,109,658	Unique
chicken:500K	1	Ciconiiformes + Sphenisciformes	169,048,476	169,052,939	Unique
chicken:500K	1	meleagris_gallopavo	169,133,510	169,135,697	Unique
chicken:500K	1	meleagris_gallopavo	170,726,415	170,727,584	Unique
chicken:500K	1	manacus_vitellinus	171,611,382	171,612,419	Unique
chicken:500K	1	anolis_carolinensis	175,314,049	175,317,750	Unique
chicken:500K	1	anolis_carolinensis	176,381,785	176,592,647	Unique
chicken:500K	1	cuculus_canorus	177,114,830	177,144,749	Unique
chicken:500K	1	chrysemys_picta	178,094,496	178,308,958	Unique
chicken:500K	1	pygoscelis_adeliae	178,357,941	178,360,818	Unique
chicken:500K	1	struthio_camelus	178,401,152	178,407,963	Unique
chicken:500K	1	taeniopygia_guttata	178,954,904	178,957,416	Unique
chicken:500K	1	anolis_carolinensis	179,164,984	179,793,950	Unique
chicken:500K	1	struthio_camelus	184,894,333	184,914,526	Unique
chicken:500K	1	falco_peregrinus	186,379,419	186,478,734	Unique
chicken:500K	1	struthio_camelus	187,544,423	187,546,090	Unique
chicken:500K	1	cuculus_canorus	187,950,216	187,955,312	Unique
chicken:500K	1	chrysemys_picta	188,188,795	188,199,033	Unique
chicken:500K	1	cuculus_canorus:picoides_pubescens	189,469,227	189,477,787	Reuse
chicken:500K	1	picoides_pubescens:cuculus_canorus	189,469,227	189,477,787	Reuse
chicken:500K	1	melopsittacus_undulatus	189,928,555	189,931,148	Unique
chicken:500K	1	corvus_brachyrhynchos	192,771,002	192,776,115	Unique
chicken:500K	1	passeriformes	193,329,934	193,952,186	Unique
chicken:500K	2	Passeriformes + Psittaciformes + Falconiformes + Piciformes	928,914	971,312	Unique
chicken:500K	2	galliformes	4,047,084	4,051,112	Unique
chicken:500K	2	anolis_carolinensis	5,578,470	5,838,444	Unique
chicken:500K	2	melopsittacus_undulatus	7,833,126	7,839,039	Unique
chicken:500K	2	picoides_pubescens	9,565,330	9,612,065	Unique
chicken:500K	2	picoides_pubescens	10,233,846	10,245,986	Unique
chicken:500K	2	struthio_camelus:aptenodytes_forsteri	14,004,515	14,021,020	Reuse
chicken:500K	2	aptenodytes_forsteri:struthio_camelus	14,004,515	14,021,020	Reuse
chicken:500K	2	picoides_pubescens	14,896,790	14,917,682	Unique
chicken:500K	2	pygoscelis_adeliae	15,097,979	15,100,449	Unique
chicken:500K	2	picoides_pubescens	16,337,599	16,816,583	Unique
chicken:500K	2	falco_peregrinus	18,278,136	18,282,859	Unique
chicken:500K	2	chrysemys_picta	19,573,392	19,974,224	Unique
chicken:500K	2	chrysemys_picta	20,484,525	20,717,484	Unique
chicken:500K	2	chrysemys_picta	21,420,367	21,455,567	Unique
chicken:500K	2	chrysemys_picta:picoides_pubescens	22,503,208	22,763,386	Reuse
chicken:500K	2	picoides_pubescens:chrysemys_picta	22,503,208	22,763,386	Reuse
chicken:500K	2	taeniopygia_guttata	26,116,750	26,118,526	Unique
chicken:500K	2	aptenodytes_forsteri	26,451,737	26,455,906	Unique
chicken:500K	2	chrysemys_picta	27,442,276	27,450,273	Unique
chicken:500K	2	chrysemys_picta	29,197,699	29,249,212	Unique
chicken:500K	2	melopsittacus_undulatus	31,014,934	31,023,417	Unique
chicken:500K	2	chinese_alligator	32,269,016	32,316,488	Unique
chicken:500K	2	anolis_carolinensis	32,508,397	32,513,114	Unique
chicken:500K	2	ophisthocomus_hoazin	33,487,563	33,492,713	Unique
chicken:500K	2	anolis_carolinensis	34,287,241	34,369,134	Unique
chicken:500K	2	chinese_alligator	34,956,592	36,065,605	Unique
chicken:500K	2	anolis_carolinensis	36,924,075	37,359,222	Unique
chicken:500K	2	columba_livia	37,400,122	37,403,989	Unique
chicken:500K	2	melopsittacus_undulatus	38,686,460	38,686,486	Unique
chicken:500K	2	meleagris_gallopavo	38,793,783	38,802,415	Unique
chicken:500K	2	anolis_carolinensis	41,649,672	41,724,825	Unique
chicken:500K	2	picoides_pubescens:opossum	41,856,660	42,041,569	Reuse
chicken:500K	2	opossum:picoides_pubescens	41,856,660	42,041,569	Reuse
chicken:500K	2	charadrius_vociferus	42,749,690	42,759,851	Unique
chicken:500K	2	python_molurus	43,658,775	43,798,884	Unique
chicken:500K	2	ophisthocomus_hoazin:pygoscelis_adeliae	44,016,868	44,019,713	Reuse
chicken:500K	2	pygoscelis_adeliae:ophisthocomus_hoazin	44,016,868	44,019,713	Reuse
chicken:500K	2	anolis_carolinensis	45,513,934	45,689,972	Unique
chicken:500K	2	taeniopygia_guttata	46,454,805	46,458,276	Unique
chicken:500K	2	anas_platyrhynchos	46,592,363	46,604,008	Unique
chicken:500K	2	neoavians	46,618,768	46,626,138	Unique
chicken:500K	2	chrysemys_picta	47,564,452	47,577,214	Unique

chicken:500K	2	cuculus_canorus	48,654,167	48,697,674	Unique
chicken:500K	2	cuculus_canorus	49,539,261	49,539,440	Unique
chicken:500K	2	neoavians	49,657,416	49,732,044	Unique
chicken:500K	2	columba_livia	50,464,370	50,473,358	Unique
chicken:500K	2	chrysemys_picta	51,643,783	51,658,799	Unique
chicken:500K	2	picoides_pubescens	51,752,914	51,764,177	Unique
chicken:500K	2	chaetura_pelagica	52,387,606	52,390,295	Unique
chicken:500K	2	meleagris_gallopavo	52,718,147	52,750,067	Unique
chicken:500K	2	nipponia_nippon:chrysemys_picta	52,808,248	52,811,678	Reuse
chicken:500K	2	chrysemys_picta:nipponia_nippon	52,808,248	52,811,678	Reuse
chicken:500K	2	nipponia_nippon	53,328,504	53,359,492	Unique
chicken:500K	2	picoides_pubescens	54,271,250	54,315,000	Unique
chicken:500K	2	taeniopygia_guttata	55,061,661	55,098,773	Unique
chicken:500K	2	columba_livia	55,420,152	55,420,379	Unique
chicken:500K	2	Passeriformes + Psittaciformes + Falconiformes + Piciformes + Ciconiiformes + Sphenisciformes + Charadriiformes + Opisthocomiformes	55,625,186	55,637,364	Unique
chicken:500K	2	columba_livia:struthio_camelus	56,143,114	56,146,951	Reuse
chicken:500K	2	struthio_camelus:columba_livia	56,143,114	56,146,951	Reuse
chicken:500K	2	neoavians	57,834,983	57,839,617	Unique
chicken:500K	2	meleagris_gallopavo	59,207,282	59,209,076	Unique
chicken:500K	2	meleagris_gallopavo:chrysemys_picta	59,850,771	59,852,075	Reuse
chicken:500K	2	chrysemys_picta:meleagris_gallopavo	59,850,771	59,852,075	Reuse
chicken:500K	2	ophisthocomus_hoazin	60,296,742	60,300,829	Unique
chicken:500K	2	chrysemys_picta	60,691,648	60,712,604	Unique
chicken:500K	2	anolis_carolinensis	62,619,229	62,677,603	Unique
chicken:500K	2	anas_platyrhynchos	63,025,671	63,079,431	Unique
chicken:500K	2	taeniopygia_guttata	64,083,924	64,085,056	Unique
chicken:500K	2	picoides_pubescens	64,401,902	64,472,781	Unique
chicken:500K	2	anas_platyrhynchos	66,011,079	66,012,678	Unique
chicken:500K	2	picoides_pubescens	67,726,132	67,838,667	Unique
chicken:500K	2	geospiza_fortis	67,926,125	67,927,167	Unique
chicken:500K	2	columba_livia:chrysemys_picta	69,060,390	69,237,940	Reuse
chicken:500K	2	chrysemys_picta:columba_livia	69,060,390	69,237,940	Reuse
chicken:500K	2	ophisthocomus_hoazin	69,470,177	69,474,153	Unique
chicken:500K	2	chrysemys_picta	70,387,946	70,406,245	Unique
chicken:500K	2	falco_peregrinus	70,532,135	70,564,371	Unique
chicken:500K	2	chrysemys_picta	71,356,398	71,902,474	Unique
chicken:500K	2	columba_livia	72,385,540	72,386,007	Unique
chicken:500K	2	meleagris_gallopavo	72,565,588	72,660,123	Unique
chicken:500K	2	taeniopygia_guttata	73,462,415	73,494,799	Unique
chicken:500K	2	chrysemys_picta	73,496,510	73,557,281	Unique
chicken:500K	2	anolis_carolinensis	74,438,667	75,239,376	Unique
chicken:500K	2	anas_platyrhynchos	76,265,023	76,266,817	Unique
chicken:500K	2	falco_peregrinus	76,709,182	77,153,832	Unique
chicken:500K	2	birds	78,382,275	78,396,083	Unique
chicken:500K	2	neoavians	79,878,460	79,888,334	Unique
chicken:500K	2	struthio_camelus	81,209,875	81,215,770	Unique
chicken:500K	2	chrysemys_picta	81,296,190	81,314,003	Unique
chicken:500K	2	chrysemys_picta	83,279,801	83,290,012	Unique
chicken:500K	2	birds	85,698,636	85,709,753	Unique
chicken:500K	2	anolis_carolinensis	86,425,531	86,455,551	Unique
chicken:500K	2	birds_crocs	88,378,699	88,434,628	Unique
chicken:500K	2	chaetura_pelagica	88,883,527	88,884,062	Unique
chicken:500K	2	chrysemys_picta	89,119,372	89,123,243	Unique
chicken:500K	2	falco_peregrinus	89,660,923	89,668,960	Unique
chicken:500K	2	picoides_pubescens	91,979,356	91,988,403	Unique
chicken:500K	2	falco_peregrinus	93,814,414	93,816,927	Unique
chicken:500K	2	taeniopygia_guttata	94,197,054	94,203,449	Unique
chicken:500K	2	taeniopygia_guttata	94,827,570	94,834,761	Unique
chicken:500K	2	aptenodytes_forsteri	95,114,680	95,117,372	Unique
chicken:500K	2	pygoscelis_adeliae	96,014,959	96,016,688	Unique
chicken:500K	2	anas_platyrhynchos	96,880,614	96,886,939	Unique
chicken:500K	2	anolis_carolinensis	96,955,191	97,677,649	Unique
chicken:500K	2	struthio_camelus	97,945,564	97,949,845	Unique
chicken:500K	2	pygoscelis_adeliae	98,868,998	98,870,932	Unique
chicken:500K	2	anolis_carolinensis	99,533,379	99,851,865	Unique

chicken:500K	2	pygoscelis_adeliae	100,337,346	100,380,203	Unique
chicken:500K	2	chrysemys_picta	100,616,775	100,626,901	Unique
chicken:500K	2	anolis_carolinensis	100,762,686	100,835,482	Unique
chicken:500K	2	anas_platyrhynchos	102,943,406	102,945,373	Unique
chicken:500K	2	falco_peregrinus	105,357,995	105,413,652	Unique
chicken:500K	2	anolis_carolinensis	105,489,595	105,536,739	Unique
chicken:500K	2	struthio_camelus	105,901,491	105,905,922	Unique
chicken:500K	2	melopsittacus_undulatus	107,559,592	107,562,725	Unique
chicken:500K	2	pygoscelis_adeliae	108,425,862	108,874,623	Unique
chicken:500K	2	struthio_camelus	109,201,569	109,208,436	Unique
chicken:500K	2	pygoscelis_adeliae	109,557,779	109,559,063	Unique
chicken:500K	2	anas_platyrhynchos	109,781,749	109,784,465	Unique
chicken:500K	2	anolis_carolinensis	111,008,260	111,387,305	Unique
chicken:500K	2	geospiza_fortis	112,225,596	112,226,472	Unique
chicken:500K	2	melopsittacus_undulatus	114,277,052	114,278,756	Unique
chicken:500K	2	struthio_camelus	116,406,396	116,407,730	Unique
chicken:500K	2	anolis_carolinensis	118,487,810	118,498,553	Unique
chicken:500K	2	picoides_pubescens	119,947,980	119,976,619	Unique
chicken:500K	2	chrysemys_picta:struthio_camelus	122,444,754	122,449,354	Reuse
chicken:500K	2	struthio_camelus:chrysemys_picta	122,444,754	122,449,354	Reuse
chicken:500K	2	nipponia_nippon	123,291,867	123,293,778	Unique
chicken:500K	2	chrysemys_picta	124,264,540	124,282,656	Unique
chicken:500K	2	anolis_carolinensis	124,530,222	124,554,787	Unique
chicken:500K	2	picoides_pubescens	127,244,401	127,255,289	Unique
chicken:500K	2	anolis_carolinensis	127,543,498	127,591,374	Unique
chicken:500K	2	pygoscelis_adeliae	127,901,162	127,901,403	Unique
chicken:500K	2	chrysemys_picta	128,279,856	128,287,485	Unique
chicken:500K	2	aptenodytes_forsteri	128,340,629	128,343,689	Unique
chicken:500K	2	anolis_carolinensis	128,382,165	128,416,026	Unique
chicken:500K	2	struthio_camelus	128,418,469	128,422,204	Unique
chicken:500K	2	picoides_pubescens	128,701,548	128,858,113	Unique
chicken:500K	2	aptenodytes_forsteri	129,424,372	129,428,230	Unique
chicken:500K	2	chrysemys_picta	130,016,627	130,032,147	Unique
chicken:500K	2	struthio_camelus	131,246,844	131,249,972	Unique
chicken:500K	2	chrysemys_picta	131,305,924	131,315,078	Unique
chicken:500K	2	picoides_pubescens	131,634,467	131,653,656	Unique
chicken:500K	2	manacus_vitellinus	132,109,270	132,114,257	Unique
chicken:500K	2	chrysemys_picta	132,538,681	132,757,194	Unique
chicken:500K	2	chrysemys_picta:anolis_carolinensis	134,886,970	135,010,512	Reuse
chicken:500K	2	anolis_carolinensis:chrysemys_picta	134,886,970	135,010,512	Reuse
chicken:500K	2	chrysemys_picta	135,864,383	135,878,776	Unique
chicken:500K	2	picoides_pubescens	135,972,434	136,021,056	Unique
chicken:500K	2	birds_crocs_turtles:anas_platyrhynchos	137,356,785	137,389,720	Reuse
chicken:500K	2	anas_platyrhynchos:birds_crocs_turtles	137,356,785	137,389,720	Reuse
chicken:500K	2	picoides_pubescens	137,870,814	137,878,624	Unique
chicken:500K	2	struthio_camelus	140,138,239	140,146,851	Unique
chicken:500K	2	anolis_carolinensis	140,575,554	140,851,304	Unique
chicken:500K	2	anolis_carolinensis	143,321,917	143,679,266	Unique
chicken:500K	2	struthio_camelus	144,835,742	144,841,026	Unique
chicken:500K	2	falco_peregrinus	145,287,794	145,290,476	Unique
chicken:500K	2	birds_crocs_turtles	146,182,101	146,192,494	Unique
chicken:500K	2	struthio_camelus	146,842,177	146,845,526	Unique
chicken:500K	2	pygoscelis_adeliae	147,466,569	147,467,486	Unique
chicken:500K	2	falco_peregrinus	147,541,362	147,846,389	Unique
chicken:500K	3	chicken	2,394,598	2,405,742	Unique
chicken:500K	3	calypte_anna	3,034,655	3,038,742	Unique
chicken:500K	3	picoides_pubescens	3,210,895	3,312,595	Unique
chicken:500K	3	chicken	5,597,364	5,606,412	Unique
chicken:500K	3	chrysemys_picta	6,783,447	6,804,463	Unique
chicken:500K	3	galliformes	7,564,535	7,578,446	Unique
chicken:500K	3	anas_platyrhynchos	8,243,627	8,254,311	Unique
chicken:500K	3	picoides_pubescens:anolis_carolinensis	9,561,036	9,575,521	Reuse
chicken:500K	3	anolis_carolinensis:picoides_pubescens	9,561,036	9,575,521	Reuse
chicken:500K	3	meleagris_gallopavo	11,579,794	11,585,862	Unique
chicken:500K	3	anolis_carolinensis	16,952,136	16,964,605	Unique
chicken:500K	3	corvus_brachyrhynchos	17,188,768	17,189,038	Unique

chicken:500K	3	struthio_camelus	17,470,948	17,525,202	Unique
chicken:500K	3	columba_livia	17,758,951	17,766,813	Unique
chicken:500K	3	egretta_garzetta	18,083,048	18,085,541	Unique
chicken:500K	3	chrysemys_picta:melopsittacus_undulatus	20,095,193	20,134,530	Reuse
chicken:500K	3	melopsittacus_undulatus:chrysemys_picta	20,095,193	20,134,530	Reuse
chicken:500K	3	picoides_pubescens	20,356,657	20,392,546	Unique
chicken:500K	3	chrysemys_picta:corvus_brachyrhynchos	21,477,513	21,479,402	Reuse
chicken:500K	3	corvus_brachyrhynchos:chrysemys_picta	21,477,513	21,479,402	Reuse
chicken:500K	3	anolis_carolinensis	22,895,372	23,190,113	Unique
chicken:500K	3	chrysemys_picta	23,378,425	23,464,347	Unique
chicken:500K	3	anolis_carolinensis	25,985,032	26,040,209	Unique
chicken:500K	3	taeniopygia_guttata	26,122,615	26,127,210	Unique
chicken:500K	3	birds	26,543,594	26,565,518	Unique
chicken:500K	3	birds_crocs_turtles	27,300,432	27,636,343	Unique
chicken:500K	3	taeniopygia_guttata	28,438,993	28,441,126	Unique
chicken:500K	3	anolis_carolinensis	28,776,057	28,776,349	Unique
chicken:500K	3	anolis_carolinensis	30,131,681	30,172,432	Unique
chicken:500K	3	passeroidea + corvoidea	30,203,130	30,204,703	Unique
chicken:500K	3	melopsittacus_undulatus	32,134,455	32,139,488	Unique
chicken:500K	3	cuculus_canorus:egretta_garzetta	33,172,086	33,191,956	Reuse
chicken:500K	3	egretta_garzetta:cuculus_canorus	33,172,086	33,191,956	Reuse
chicken:500K	3	anolis_carolinensis	33,265,404	33,525,193	Unique
chicken:500K	3	anolis_carolinensis	34,654,797	34,911,638	Unique
chicken:500K	3	corvus_brachyrhynchos	35,000,702	35,006,708	Unique
chicken:500K	3	struthio_camelus	37,224,096	37,226,700	Unique
chicken:500K	3	anas_platyrhynchos	38,115,541	38,123,348	Unique
chicken:500K	3	struthio_camelus	41,747,124	41,750,271	Unique
chicken:500K	3	melopsittacus_undulatus	41,860,916	41,870,781	Unique
chicken:500K	3	anolis_carolinensis	42,163,634	42,165,311	Unique
chicken:500K	3	chinese_alligator	42,800,697	43,188,933	Unique
chicken:500K	3	chrysemys_picta	44,186,247	44,199,568	Unique
chicken:500K	3	ophisthocomus_hoazin	44,428,797	44,430,222	Unique
chicken:500K	3	birds_crocs	45,109,646	45,152,243	Unique
chicken:500K	3	anolis_carolinensis	47,049,758	47,352,534	Unique
chicken:500K	3	passeroidea + corvoidea	47,422,091	47,423,361	Unique
chicken:500K	3	anolis_carolinensis	48,670,177	48,758,800	Unique
chicken:500K	3	calypte_anna	51,453,134	51,493,822	Unique
chicken:500K	3	melopsittacus_undulatus	52,415,801	52,432,139	Unique
chicken:500K	3	anolis_carolinensis	52,784,101	53,007,620	Unique
chicken:500K	3	chaetura_pelagica	54,136,314	54,477,699	Unique
chicken:500K	3	corvus_brachyrhynchos	54,847,632	54,849,749	Unique
chicken:500K	3	passeroidea	54,850,519	54,851,694	Unique
chicken:500K	3	struthio_camelus	58,590,235	58,592,262	Unique
chicken:500K	3	anolis_carolinensis	58,604,857	58,916,847	Unique
chicken:500K	3	falco_peregrinus	61,923,831	62,055,966	Unique
chicken:500K	3	chaetura_pelagica	64,225,811	64,231,525	Unique
chicken:500K	3	anolis_carolinensis	65,191,583	65,203,410	Unique
chicken:500K	3	struthio_camelus	65,645,379	65,651,342	Unique
chicken:500K	3	geospiza_fortis	67,404,827	67,409,769	Unique
chicken:500K	3	anolis_carolinensis	68,001,732	68,128,932	Unique
chicken:500K	3	anolis_carolinensis	68,629,451	69,430,563	Unique
chicken:500K	3	struthio_camelus	69,637,970	69,639,587	Unique
chicken:500K	3	falco_peregrinus	70,640,657	70,678,115	Unique
chicken:500K	3	anolis_carolinensis:melopsittacus_undulatus	72,670,426	72,673,346	Reuse
chicken:500K	3	melopsittacus_undulatus:anolis_carolinensis	72,670,426	72,673,346	Reuse
chicken:500K	3	geospiza_fortis	72,951,449	72,960,997	Unique
chicken:500K	3	anolis_carolinensis:melopsittacus_undulatus	74,145,584	74,152,981	Reuse
chicken:500K	3	melopsittacus_undulatus:anolis_carolinensis	74,145,584	74,152,981	Reuse
chicken:500K	3	chrysemys_picta	74,404,804	74,419,563	Unique
chicken:500K	3	struthio_camelus	74,816,295	74,819,040	Unique
chicken:500K	3	geospiza_fortis	75,149,623	75,159,429	Unique
chicken:500K	3	anolis_carolinensis	76,384,821	76,407,626	Unique
chicken:500K	3	struthio_camelus	79,181,347	79,695,187	Unique
chicken:500K	3	anolis_carolinensis	80,710,111	80,832,266	Unique
chicken:500K	3	anolis_carolinensis	81,501,222	81,550,132	Unique
chicken:500K	3	picoides_pubescens	81,819,702	81,827,118	Unique

chicken:500K	3	geospiza_fortis	86,132,809	86,303,133	Unique
chicken:500K	3	anolis_carolinensis	86,539,833	86,633,549	Unique
chicken:500K	3	aptenodytes_forsteri	87,175,111	87,177,148	Unique
chicken:500K	3	anolis_carolinensis	87,502,591	87,541,506	Unique
chicken:500K	3	anolis_carolinensis	88,994,470	89,077,886	Unique
chicken:500K	3	struthio_camelus	89,250,610	89,251,944	Unique
chicken:500K	3	ophisthocomus_hoazin	89,596,487	90,009,436	Unique
chicken:500K	3	corvus_brachyrhynchos	90,967,476	90,968,359	Unique
chicken:500K	3	ophisthocomus_hoazin	91,708,720	91,762,166	Unique
chicken:500K	3	chinese_alligator	91,896,786	92,499,651	Unique
chicken:500K	3	anolis_carolinensis:picoides_pubescens	93,356,963	93,364,309	Reuse
chicken:500K	3	picoides_pubescens:anolis_carolinensis	93,356,963	93,364,309	Reuse
chicken:500K	3	anolis_carolinensis	94,181,274	94,227,755	Unique
chicken:500K	3	cuculus_canorus	95,639,534	96,059,765	Unique
chicken:500K	3	melopsittacus_undulatus:chaetura_pelagica	96,520,444	96,689,726	Reuse
chicken:500K	3	chaetura_pelagica:melopsittacus_undulatus	96,520,444	96,689,726	Reuse
chicken:500K	3	anolis_carolinensis	99,992,424	100,123,044	Unique
chicken:500K	3	anolis_carolinensis	102,130,372	102,184,683	Unique
chicken:500K	3	picoides_pubescens	108,411,758	108,420,098	Unique
chicken:500K	3	geospiza_fortis	109,263,547	109,486,853	Unique
chicken:500K	3	struthio_camelus	109,498,583	109,502,406	Unique
chicken:500K	4	meleagris_gallopavo	1,811,290	2,377,626	Unique
chicken:500K	4	passeriformes	2,895,581	3,392,334	Unique
chicken:500K	4	melopsittacus_undulatus	4,091,249	4,093,342	Unique
chicken:500K	4	passeroidea + corvoidea	4,260,909	4,263,947	Unique
chicken:500K	4	chaetura_pelagica	4,337,257	4,340,935	Unique
chicken:500K	4	python_molurus:opossum	5,091,918	5,123,220	Reuse
chicken:500K	4	opossum:python_molurus	5,091,918	5,123,220	Reuse
chicken:500K	4	struthio_camelus	6,760,632	6,762,877	Unique
chicken:500K	4	chrysemys_picta	7,363,984	7,570,512	Unique
chicken:500K	4	birds_crocs	8,096,981	8,122,315	Unique
chicken:500K	4	aptenodytes_forsteri	8,415,160	8,419,605	Unique
chicken:500K	4	melopsittacus_undulatus	8,675,439	8,684,130	Unique
chicken:500K	4	struthio_camelus	8,950,221	8,953,754	Unique
chicken:500K	4	galliformes	9,481,895	9,530,195	Unique
chicken:500K	4	galliformes	10,936,371	10,959,832	Unique
chicken:500K	4	chaetura_pelagica	12,500,825	12,509,445	Unique
chicken:500K	4	passeriformes	13,339,174	13,345,292	Unique
chicken:500K	4	anas_platyrhynchos:melopsittacus_undulatus	14,061,412	14,063,254	Reuse
chicken:500K	4	melopsittacus_undulatus:anas_platyrhynchos	14,061,412	14,063,254	Reuse
chicken:500K	4	pygoscelis_adeliae	14,751,165	14,752,285	Unique
chicken:500K	4	columba_livia	15,343,102	15,345,468	Unique
chicken:500K	4	falco_peregrinus	15,690,999	15,705,380	Unique
chicken:500K	4	columba_livia:anas_platyrhynchos	16,436,980	16,445,038	Reuse
chicken:500K	4	anas_platyrhynchos:columba_livia	16,436,980	16,445,038	Reuse
chicken:500K	4	taeniopygia_guttata	17,284,492	17,286,345	Unique
chicken:500K	4	geospiza_fortis	17,286,387	17,286,591	Unique
chicken:500K	4	calypte_anna	17,476,955	17,530,858	Unique
chicken:500K	4	melopsittacus_undulatus	17,972,089	17,974,013	Unique
chicken:500K	4	chicken	19,197,800	19,204,786	Unique
chicken:500K	4	pygoscelis_adeliae	20,473,226	20,476,609	Unique
chicken:500K	4	egretta_garzetta:corvus_brachyrhynchos	22,618,556	22,670,686	Reuse
chicken:500K	4	corvus_brachyrhynchos:egretta_garzetta	22,618,556	22,670,686	Reuse
chicken:500K	4	anolis_carolinensis:melopsittacus_undulatus	23,169,805	23,185,692	Reuse
chicken:500K	4	melopsittacus_undulatus:anolis_carolinensis	23,169,805	23,185,692	Reuse
chicken:500K	4	anolis_carolinensis	25,685,215	25,803,724	Unique
chicken:500K	4	pygoscelis_adeliae	27,504,866	27,507,960	Unique
chicken:500K	4	chrysemys_picta	27,640,522	27,664,849	Unique
chicken:500K	4	melopsittacus_undulatus	30,420,803	30,426,405	Unique
chicken:500K	4	anolis_carolinensis	30,757,909	30,767,868	Unique
chicken:500K	4	picoides_pubescens	31,426,037	31,426,428	Unique
chicken:500K	4	chicken	34,200,808	34,294,556	Unique
chicken:500K	4	chinese_alligator	37,744,162	37,806,859	Unique
chicken:500K	4	non_galloanserae	38,688,770	38,696,308	Unique
chicken:500K	4	melopsittacus_undulatus	40,497,561	40,504,359	Unique
chicken:500K	4	galloanserae	41,740,401	41,746,757	Unique

chicken:500K	4	galliformes	48,619,510	49,044,177	Unique
chicken:500K	4	meleagris_gallopavo	49,141,083	49,142,075	Unique
chicken:500K	4	melopsittacus_undulatus	49,553,668	49,889,986	Unique
chicken:500K	4	galloanserae	50,404,603	50,409,644	Unique
chicken:500K	4	galliformes	51,215,264	51,216,208	Unique
chicken:500K	4	charadrius_vociferus	51,794,332	51,959,633	Unique
chicken:500K	4	melopsittacus_undulatus	52,379,462	52,406,329	Unique
chicken:500K	4	falco_peregrinus	53,138,363	53,144,877	Unique
chicken:500K	4	chinese_alligator:melopsittacus_undulatus	53,412,841	53,488,469	Reuse
chicken:500K	4	melopsittacus_undulatus:chinese_alligator	53,412,841	53,488,469	Reuse
chicken:500K	4	calypte_anna	53,862,071	53,943,025	Unique
chicken:500K	4	aptenodytes_forsteri	54,323,333	54,325,858	Unique
chicken:500K	4	anas_platyrhynchos	54,333,412	54,385,406	Unique
chicken:500K	4	melopsittacus_undulatus	54,952,620	54,954,385	Unique
chicken:500K	4	anas_platyrhynchos	55,401,395	55,403,856	Unique
chicken:500K	4	non_galloanserae + non columbiformes	56,811,245	56,814,510	Unique
chicken:500K	4	galliformes	57,799,990	57,801,833	Unique
chicken:500K	4	anolis_carolinensis	58,463,159	58,603,740	Unique
chicken:500K	4	struthio_camelus	59,491,056	59,495,659	Unique
chicken:500K	4	geospiza_fortis	59,566,292	59,590,610	Unique
chicken:500K	4	calypte_anna	59,600,516	59,632,524	Unique
chicken:500K	4	galliformes	60,642,480	60,644,959	Unique
chicken:500K	4	anas_platyrhynchos	62,815,625	62,817,700	Unique
chicken:500K	4	picoides_pubescens	62,931,516	62,990,410	Unique
chicken:500K	4	anas_platyrhynchos	63,850,150	63,927,173	Unique
chicken:500K	4	chrysemys_picta	64,055,029	64,077,495	Unique
chicken:500K	4	picoides_pubescens	64,616,993	64,631,141	Unique
chicken:500K	4	anolis_carolinensis	65,443,231	65,502,021	Unique
chicken:500K	4	anolis_carolinensis	66,474,562	66,662,165	Unique
chicken:500K	4	pygoscelis_adeliae	66,957,086	66,959,875	Unique
chicken:500K	4	ophisthocomus_hoazin	67,182,750	67,188,828	Unique
chicken:500K	4	chrysemys_picta	68,383,438	68,405,576	Unique
chicken:500K	4	falco_peregrinus	68,582,526	68,583,205	Unique
chicken:500K	4	anolis_carolinensis	68,900,830	69,975,771	Unique
chicken:500K	4	melopsittacus_undulatus:chrysemys_picta	70,703,860	70,957,701	Reuse
chicken:500K	4	chrysemys_picta:melopsittacus_undulatus	70,703,860	70,957,701	Reuse
chicken:500K	4	picoides_pubescens	72,985,398	73,031,031	Unique
chicken:500K	4	anolis_carolinensis	73,136,251	73,253,034	Unique
chicken:500K	4	pygoscelis_adeliae	73,555,682	73,572,126	Unique
chicken:500K	4	falco_peregrinus:nipponia_nippon	75,877,708	75,879,110	Reuse
chicken:500K	4	nipponia_nippon:falco_peregrinus	75,877,708	75,879,110	Reuse
chicken:500K	4	picoides_pubescens	76,308,041	76,321,489	Unique
chicken:500K	4	chrysemys_picta	79,805,612	79,827,283	Unique
chicken:500K	4	meleagris_gallopavo	79,978,630	79,979,564	Unique
chicken:500K	4	meleagris_gallopavo	80,965,077	80,969,237	Unique
chicken:500K	4	python_molurus	81,889,928	82,569,298	Unique
chicken:500K	4	pygoscelis_adeliae	84,316,611	84,321,659	Unique
chicken:500K	4	picoides_pubescens	84,825,333	84,858,437	Unique
chicken:500K	4	birds_crocs	85,954,474	85,968,096	Unique
chicken:500K	4	picoides_pubescens:melopsittacus_undulatus:opossum	88,048,820	88,143,200	Reuse
chicken:500K	4	melopsittacus_undulatus:opossum:picoides_pubescens	88,048,820	88,143,200	Reuse
chicken:500K	4	opossum:picoides_pubescens:melopsittacus_undulatus	88,048,820	88,143,200	Reuse
chicken:500K	5	Ciconiiformes + Sphenisciformes:opossum:taeniopygia_guttata:charadrius_vociferus:columba_livia	1,655,553	1,655,666	Reuse
chicken:500K	5	opossum:taeniopygia_guttata:charadrius_vociferus:columba_livia:Ciconiiformes + Sphenisciformes	1,655,553	1,655,666	Reuse
chicken:500K	5	taeniopygia_guttata:charadrius_vociferus:columba_livia:Ciconiiformes + Sphenisciformes:opossum	1,655,553	1,655,666	Reuse
chicken:500K	5	charadrius_vociferus:columba_livia:Ciconiiformes + Sphenisciformes:opossum:taeniopygia_guttata	1,655,553	1,655,666	Reuse
chicken:500K	5	columba_livia:Ciconiiformes + Sphenisciformes:opossum:taeniopygia_guttata:charadrius_vociferus	1,655,553	1,655,666	Reuse
chicken:500K	5	chrysemys_picta	2,304,870	2,309,984	Unique
chicken:500K	5	chrysemys_picta	3,564,577	4,065,269	Unique
chicken:500K	5	anolis_carolinensis	4,316,712	4,503,798	Unique
chicken:500K	5	chicken	5,678,440	5,833,354	Unique

chicken:500K	5	chicken	6,518,476	6,524,426	Unique
chicken:500K	5	melopsittacus_undulatus	7,667,880	7,674,323	Unique
chicken:500K	5	anolis_carolinensis	7,894,600	7,911,483	Unique
chicken:500K	5	melopsittacus_undulatus	8,632,625	8,635,023	Unique
chicken:500K	5	chinese_alligator:ophisthocomus_hoazin	8,790,208	8,805,358	Reuse
chicken:500K	5	ophisthocomus_hoazin:chinese_alligator	8,790,208	8,805,358	Reuse
chicken:500K	5	anolis_carolinensis	9,169,792	9,242,270	Unique
chicken:500K	5	anolis_carolinensis:chrysemys_picta	9,926,444	9,938,611	Reuse
chicken:500K	5	chrysemys_picta:anolis_carolinensis	9,926,444	9,938,611	Reuse
chicken:500K	5	anas_platyrhynchos	11,834,286	11,835,095	Unique
chicken:500K	5	falco_peregrinus	11,919,510	12,159,142	Unique
chicken:500K	5	chrysemys_picta	12,473,289	12,495,570	Unique
chicken:500K	5	calypte_anna	14,131,378	14,139,637	Unique
chicken:500K	5	anas_platyrhynchos	14,388,271	14,389,976	Unique
chicken:500K	5	calypte_anna	15,289,619	15,298,392	Unique
chicken:500K	5	chicken	16,150,527	16,322,850	Unique
chicken:500K	5	anolis_carolinensis	17,071,363	17,082,930	Unique
chicken:500K	5	melopsittacus_undulatus	17,115,509	17,117,046	Unique
chicken:500K	5	anolis_carolinensis	18,161,823	18,266,609	Unique
chicken:500K	5	meleagris_gallopavo	18,876,774	18,878,461	Unique
chicken:500K	5	anolis_carolinensis	19,720,786	19,741,068	Unique
chicken:500K	5	melopsittacus_undulatus	20,700,162	20,707,153	Unique
chicken:500K	5	anolis_carolinensis	20,786,555	20,994,475	Unique
chicken:500K	5	falco_peregrinus	22,196,828	22,198,798	Unique
chicken:500K	5	anas_platyrhynchos	22,450,240	22,452,053	Unique
chicken:500K	5	melopsittacus_undulatus	23,198,288	23,217,253	Unique
chicken:500K	5	chrysemys_picta:cuculus_canorus	24,157,428	24,158,554	Reuse
chicken:500K	5	cuculus_canorus:chrysemys_picta	24,157,428	24,158,554	Reuse
chicken:500K	5	falco_peregrinus	24,751,245	24,843,688	Unique
chicken:500K	5	python_molurus	25,434,270	25,459,863	Unique
chicken:500K	5	melopsittacus_undulatus	25,463,064	25,760,117	Unique
chicken:500K	5	chrysemys_picta	26,032,836	26,067,282	Unique
chicken:500K	5	anolis_carolinensis	26,641,570	26,732,789	Unique
chicken:500K	5	melopsittacus_undulatus	26,818,758	26,820,709	Unique
chicken:500K	5	calypte_anna	27,252,898	27,255,611	Unique
chicken:500K	5	picoides_pubescens	28,318,936	28,326,476	Unique
chicken:500K	5	chrysemys_picta:opossum	29,900,423	29,947,001	Reuse
chicken:500K	5	opossum:chrysemys_picta	29,900,423	29,947,001	Reuse
chicken:500K	5	chrysemys_picta	32,357,176	32,366,436	Unique
chicken:500K	5	anolis_carolinensis	33,431,580	33,444,998	Unique
chicken:500K	5	struthio_camelus	35,340,069	35,343,565	Unique
chicken:500K	5	anolis_carolinensis	35,512,215	35,565,936	Unique
chicken:500K	5	melopsittacus_undulatus	35,619,779	35,622,065	Unique
chicken:500K	5	anas_platyrhynchos	35,997,809	36,000,902	Unique
chicken:500K	5	chrysemys_picta	36,428,161	36,433,460	Unique
chicken:500K	5	anolis_carolinensis	36,776,202	36,873,114	Unique
chicken:500K	5	chrysemys_picta	39,036,097	39,040,675	Unique
chicken:500K	5	struthio_camelus	39,478,845	39,483,743	Unique
chicken:500K	5	cuculus_canorus	39,877,213	39,879,963	Unique
chicken:500K	5	melopsittacus_undulatus	40,606,713	40,607,711	Unique
chicken:500K	5	falco_peregrinus	42,299,220	42,302,334	Unique
chicken:500K	5	charadrius_vociferus:opossum	43,650,462	43,696,529	Reuse
chicken:500K	5	opossum:charadrius_vociferus	43,650,462	43,696,529	Reuse
chicken:500K	5	birds_crocs_turtles	44,859,392	44,944,181	Unique
chicken:500K	5	charadrius_vociferus	45,872,562	45,879,177	Unique
chicken:500K	5	anolis_carolinensis	47,854,136	47,878,922	Unique
chicken:500K	5	chrysemys_picta	47,950,958	47,996,300	Unique
chicken:500K	5	chrysemys_picta	48,582,743	48,605,251	Unique
chicken:500K	5	chrysemys_picta	49,911,179	49,927,169	Unique
chicken:500K	5	anolis_carolinensis	50,250,870	50,289,289	Unique
chicken:500K	5	falco_peregrinus	50,527,346	51,151,614	Unique
chicken:500K	5	chrysemys_picta	51,711,350	51,736,144	Unique
chicken:500K	5	corvus_brachyrhynchos	52,026,935	52,030,607	Unique
chicken:500K	5	chrysemys_picta	52,773,660	52,786,177	Unique
chicken:500K	5	chinese_alligator	53,672,846	53,750,240	Unique
chicken:500K	5	anolis_carolinensis	54,010,068	54,076,230	Unique

chicken:500K	5	python_molurus:charadrius_vociferus	55,173,216	55,177,208	Reuse
chicken:500K	5	charadrius_vociferus:python_molurus	55,173,216	55,177,208	Reuse
chicken:500K	5	pygoscelis_adeliae	55,487,902	55,489,159	Unique
chicken:500K	5	galliformes	56,369,450	56,371,811	Unique
chicken:500K	5	melopsittacus_undulatus	57,288,091	57,289,380	Unique
chicken:500K	5	melopsittacus_undulatus	57,848,365	57,915,389	Unique
chicken:500K	5	Passeriformes + Psittaciformes + Falconiformes + Piciformes + Ciconiiformes + Sphenisciformes + Charadriiformes + Opisthocomiformes	58,599,683	58,603,094	Unique
chicken:500K	6	columba_livia:opossum	2,108,879	2,126,676	Reuse
chicken:500K	6	opossum:columba_livia	2,108,879	2,126,676	Reuse
chicken:500K	6	melopsittacus_undulatus	4,003,881	4,073,220	Unique
chicken:500K	6	chicken	6,070,296	6,073,929	Unique
chicken:500K	6	anolis_carolinensis	6,480,390	6,887,453	Unique
chicken:500K	6	passeroidea	7,198,198	7,210,644	Unique
chicken:500K	6	chicken	10,016,053	10,021,987	Unique
chicken:500K	6	chicken	10,703,766	10,727,907	Unique
chicken:500K	6	meleagris_gallopavo	11,568,201	11,578,014	Unique
chicken:500K	6	chrysemys_picta	12,325,044	12,337,513	Unique
chicken:500K	6	columba_livia	12,714,330	12,716,660	Unique
chicken:500K	6	chrysemys_picta	13,192,400	13,207,895	Unique
chicken:500K	6	ophisthocomus_hoazin	13,358,851	13,361,102	Unique
chicken:500K	6	chrysemys_picta	13,923,466	13,937,220	Unique
chicken:500K	6	pygoscelis_adeliae	14,310,596	14,315,071	Unique
chicken:500K	6	falco_peregrinus	14,954,274	14,959,018	Unique
chicken:500K	6	chrysemys_picta	15,080,377	15,129,014	Unique
chicken:500K	6	melopsittacus_undulatus	15,571,507	15,572,968	Unique
chicken:500K	6	calypte_anna	17,069,464	17,076,358	Unique
chicken:500K	6	melopsittacus_undulatus	18,378,878	18,382,360	Unique
chicken:500K	6	chrysemys_picta	18,648,270	18,697,513	Unique
chicken:500K	6	chrysemys_picta	19,929,389	19,942,492	Unique
chicken:500K	6	melopsittacus_undulatus	22,070,821	22,074,658	Unique
chicken:500K	6	anolis_carolinensis	22,620,021	22,623,448	Unique
chicken:500K	6	charadrius_vociferus	22,717,531	22,721,730	Unique
chicken:500K	6	cuculus_canorus	25,352,314	25,354,524	Unique
chicken:500K	6	anolis_carolinensis	25,956,351	26,158,183	Unique
chicken:500K	6	anolis_carolinensis	29,267,945	29,766,626	Unique
chicken:500K	6	falco_peregrinus	29,922,315	29,936,724	Unique
chicken:500K	6	anolis_carolinensis	30,891,725	30,924,648	Unique
chicken:500K	6	picoides_pubescens:opossum	31,548,944	31,552,698	Reuse
chicken:500K	6	opossum:picoides_pubescens	31,548,944	31,552,698	Reuse
chicken:500K	6	anolis_carolinensis	32,298,609	32,358,786	Unique
chicken:500K	6	chrysemys_picta	34,056,707	34,087,323	Unique
chicken:500K	7	cuculus_canorus	824,926	1,046,242	Unique
chicken:500K	7	columba_livia	1,368,266	1,410,428	Unique
chicken:500K	7	calypte_anna:ophisthocomus_hoazin	2,177,457	2,190,166	Reuse
chicken:500K	7	ophisthocomus_hoazin:calypte_anna	2,177,457	2,190,166	Reuse
chicken:500K	7	neoavians	2,311,590	2,315,242	Unique
chicken:500K	7	picoides_pubescens:taeniopygia_guttata	3,524,947	3,533,623	Reuse
chicken:500K	7	taeniopygia_guttata:picoides_pubescens	3,524,947	3,533,623	Reuse
chicken:500K	7	taeniopygia_guttata	4,500,197	4,500,975	Unique
chicken:500K	7	melopsittacus_undulatus	4,504,977	4,610,656	Unique
chicken:500K	7	pygoscelis_adeliae	4,885,804	4,889,908	Unique
chicken:500K	7	columba_livia:opossum	5,560,953	5,565,900	Reuse
chicken:500K	7	opossum:columba_livia	5,560,953	5,565,900	Reuse
chicken:500K	7	ophisthocomus_hoazin	6,640,074	6,645,292	Unique
chicken:500K	7	chicken	7,336,101	7,339,690	Unique
chicken:500K	7	aptenodytes_forsteri	8,788,746	8,793,058	Unique
chicken:500K	7	anolis_carolinensis	8,811,258	8,858,082	Unique
chicken:500K	7	columba_livia	9,275,107	9,281,206	Unique
chicken:500K	7	picoides_pubescens	9,513,620	9,526,053	Unique
chicken:500K	7	chinese_alligator:calypte_anna:opossum	11,000,835	11,003,302	Reuse
chicken:500K	7	calypte_anna:opossum:chinese_alligator	11,000,835	11,003,302	Reuse
chicken:500K	7	opossum:chinese_alligator:calypte_anna	11,000,835	11,003,302	Reuse
chicken:500K	7	birds	11,508,923	11,525,238	Unique
chicken:500K	7	melopsittacus_undulatus	13,272,439	13,273,667	Unique
chicken:500K	7	columba_livia	13,394,943	13,626,182	Unique

chicken:500K	7	aptenodytes_forsteri	13,832,970	13,838,513	Unique
chicken:500K	7	anas_platyrhynchos	14,086,550	14,087,974	Unique
chicken:500K	7	python_molurus	15,382,491	15,399,827	Unique
chicken:500K	7	chinese_alligator	16,675,493	17,083,971	Unique
chicken:500K	7	melopsittacus_undulatus	21,369,589	21,372,225	Unique
chicken:500K	7	nipponia_nippon:anolis_carolinensis	22,753,402	22,756,738	Reuse
chicken:500K	7	anolis_carolinensis:nipponia_nippon	22,753,402	22,756,738	Reuse
chicken:500K	7	anolis_carolinensis	23,534,976	23,629,770	Unique
chicken:500K	7	anolis_carolinensis	26,139,183	26,187,705	Unique
chicken:500K	7	taeniopygia_guttata	27,786,737	27,787,078	Unique
chicken:500K	7	chrysemys_picta	27,914,687	27,927,763	Unique
chicken:500K	7	chrysemys_picta	28,528,179	28,737,932	Unique
chicken:500K	7	struthio_camelus	29,155,877	29,161,171	Unique
chicken:500K	7	anolis_carolinensis	29,205,132	29,769,898	Unique
chicken:500K	7	chrysemys_picta	30,054,713	30,592,770	Unique
chicken:500K	7	picoides_pubescens	30,722,936	30,726,898	Unique
chicken:500K	7	anolis_carolinensis	33,294,489	33,304,904	Unique
chicken:500K	7	chrysemys_picta	33,391,485	33,397,087	Unique
chicken:500K	7	meleagris_gallopavo	33,538,585	33,554,365	Unique
chicken:500K	7	chinese_alligator	33,611,512	34,511,981	Unique
chicken:500K	7	meleagris_gallopavo	34,739,968	34,750,782	Unique
chicken:500K	7	chrysemys_picta	35,066,129	35,309,128	Unique
chicken:500K	7	meleagris_gallopavo	35,507,172	35,544,927	Unique
chicken:500K	8	anolis_carolinensis	1,107,900	1,172,406	Unique
chicken:500K	8	ophisthocomus_hoazin	1,251,591	1,253,849	Unique
chicken:500K	8	meleagris_gallopavo	1,280,561	1,281,898	Unique
chicken:500K	8	egretta_garzetta	1,337,573	1,339,293	Unique
chicken:500K	8	melopsittacus_undulatus	1,704,944	1,706,600	Unique
chicken:500K	8	falco_peregrinus:pygoscelis_adeliae:melopsittacus_undulatus	2,795,069	2,851,984	Reuse
chicken:500K	8	pygoscelis_adeliae:melopsittacus_undulatus:falco_peregrinus	2,795,069	2,851,984	Reuse
chicken:500K	8	melopsittacus_undulatus:falco_peregrinus:pygoscelis_adeliae	2,795,069	2,851,984	Reuse
chicken:500K	8	calypte_anna	3,561,344	3,597,538	Unique
chicken:500K	8	neoavians	3,968,737	3,995,355	Unique
chicken:500K	8	picoides_pubescens	4,797,639	4,817,837	Unique
chicken:500K	8	melopsittacus_undulatus:passeroidea	6,050,534	6,058,098	Reuse
chicken:500K	8	passeroidea:melopsittacus_undulatus	6,050,534	6,058,098	Reuse
chicken:500K	8	calypte_anna	6,062,393	6,067,710	Unique
chicken:500K	8	falco_peregrinus	6,979,710	7,023,495	Unique
chicken:500K	8	chaetura_pelagica	7,055,817	7,082,127	Unique
chicken:500K	8	galliformes	8,030,291	8,063,725	Unique
chicken:500K	8	falco_peregrinus	8,647,185	8,741,245	Unique
chicken:500K	8	chicken	9,981,443	9,998,301	Unique
chicken:500K	8	ophisthocomus_hoazin	10,593,359	10,597,301	Unique
chicken:500K	8	birds_crocs_turtles	10,985,404	11,017,165	Unique
chicken:500K	8	melopsittacus_undulatus	11,136,138	11,138,691	Unique
chicken:500K	8	meleagris_gallopavo	11,885,274	11,887,182	Unique
chicken:500K	8	melopsittacus_undulatus	12,729,848	12,737,159	Unique
chicken:500K	8	struthio_camelus	12,809,192	12,811,568	Unique
chicken:500K	8	melopsittacus_undulatus:anas_platyrhynchos	13,898,955	13,900,642	Reuse
chicken:500K	8	anas_platyrhynchos:melopsittacus_undulatus	13,898,955	13,900,642	Reuse
chicken:500K	8	struthio_camelus	14,545,573	14,549,643	Unique
chicken:500K	8	manacus_vitellinus	15,376,281	15,386,615	Unique
chicken:500K	8	corvus_brachyrhynchos	16,578,881	16,580,645	Unique
chicken:500K	8	struthio_camelus	17,503,033	17,505,016	Unique
chicken:500K	8	picoides_pubescens	18,548,061	18,554,640	Unique
chicken:500K	8	anolis_carolinensis	19,661,296	20,090,925	Unique
chicken:500K	8	chrysemys_picta	20,496,234	20,541,208	Unique
chicken:500K	8	columba_livia	20,586,001	20,588,474	Unique
chicken:500K	8	anolis_carolinensis	20,715,723	20,746,966	Unique
chicken:500K	8	anolis_carolinensis	21,742,874	22,238,521	Unique
chicken:500K	8	columba_livia	23,002,549	23,005,948	Unique
chicken:500K	8	chrysemys_picta	23,113,815	23,114,945	Unique
chicken:500K	8	corvus_brachyrhynchos	23,290,564	23,292,328	Unique
chicken:500K	8	chrysemys_picta	24,288,232	24,296,277	Unique
chicken:500K	8	chrysemys_picta	25,564,078	25,598,820	Unique
chicken:500K	8	chrysemys_picta	26,810,563	26,825,266	Unique

chicken:500K	8	anolis_carolinensis:chrysemys_picta	27,715,794	27,741,107	Reuse
chicken:500K	8	chrysemys_picta:anolis_carolinensis	27,715,794	27,741,107	Reuse
chicken:500K	9	melopsittacus_undulatus	803,621	809,515	Unique
chicken:500K	9	taeniopygia_guttata	905,234	911,862	Unique
chicken:500K	9	anas_platyrhynchos	1,707,342	1,731,126	Unique
chicken:500K	9	picoides_pubescens	2,002,526	2,011,901	Unique
chicken:500K	9	anolis_carolinensis	2,191,424	2,748,338	Unique
chicken:500K	9	chicken	2,892,593	2,958,453	Unique
chicken:500K	9	galliformes	4,713,046	4,717,342	Unique
chicken:500K	9	chaetura_pelagica	5,588,899	5,599,095	Unique
chicken:500K	9	passeroidea	6,021,423	6,050,816	Unique
chicken:500K	9	anolis_carolinensis	7,037,589	7,060,700	Unique
chicken:500K	9	nipponia_nippon	8,372,133	8,374,940	Unique
chicken:500K	9	melopsittacus_undulatus	8,751,134	8,760,278	Unique
chicken:500K	9	calypte_anna	9,248,220	9,259,023	Unique
chicken:500K	9	struthio_camelus	9,514,536	9,659,195	Unique
chicken:500K	9	picoides_pubescens	10,035,640	10,040,961	Unique
chicken:500K	9	nipponia_nippon	10,207,876	10,211,038	Unique
chicken:500K	9	birds_crocs	11,939,732	11,941,765	Unique
chicken:500K	9	melopsittacus_undulatus	13,441,937	13,445,979	Unique
chicken:500K	9	columba_livia	15,160,944	15,165,067	Unique
chicken:500K	9	egretta_garzetta:opossum	15,661,278	15,666,219	Reuse
chicken:500K	9	opossum:egretta_garzetta	15,661,278	15,666,219	Reuse
chicken:500K	9	aptenodytes_forsteri	18,887,314	18,890,092	Unique
chicken:500K	9	columba_livia	19,340,917	19,343,880	Unique
chicken:500K	9	picoides_pubescens	20,143,957	20,239,721	Unique
chicken:500K	9	melopsittacus_undulatus	20,310,029	20,542,557	Unique
chicken:500K	10	python_molurus	1,092,919	1,100,881	Unique
chicken:500K	10	anas_platyrhynchos	2,035,057	2,050,944	Unique
chicken:500K	10	python_molurus	3,177,480	3,335,685	Unique
chicken:500K	10	columba_livia	3,528,267	3,535,515	Unique
chicken:500K	10	passeroidea	3,606,688	3,614,022	Unique
chicken:500K	10	columba_livia	4,539,435	4,542,050	Unique
chicken:500K	10	picoides_pubescens	4,719,159	4,723,126	Unique
chicken:500K	10	geospiza_fortis	5,130,398	5,131,698	Unique
chicken:500K	10	birds_crocs_turtles	6,084,476	6,216,543	Unique
chicken:500K	10	melopsittacus_undulatus	6,299,680	6,302,508	Unique
chicken:500K	10	corvus_brachyrhynchos:columba_livia	7,420,438	7,420,666	Reuse
chicken:500K	10	columba_livia:corvus_brachyrhynchos	7,420,438	7,420,666	Reuse
chicken:500K	10	cuculus_canorus:opossum	7,523,168	7,526,342	Reuse
chicken:500K	10	opossum:cuculus_canorus	7,523,168	7,526,342	Reuse
chicken:500K	10	chrysemys_picta	10,110,317	10,126,178	Unique
chicken:500K	10	anas_platyrhynchos	10,224,248	10,225,564	Unique
chicken:500K	10	picoides_pubescens	11,157,298	11,165,493	Unique
chicken:500K	10	python_molurus	13,640,138	13,702,296	Unique
chicken:500K	10	python_molurus	14,416,785	14,476,395	Unique
chicken:500K	10	pygoscelis_adeliae	15,569,181	15,571,720	Unique
chicken:500K	10	python_molurus	16,376,918	16,403,220	Unique
chicken:500K	10	picoides_pubescens	16,657,158	17,157,759	Unique
chicken:500K	10	chrysemys_picta	18,068,255	18,076,445	Unique
chicken:500K	11	melopsittacus_undulatus	1,456,402	1,461,750	Unique
chicken:500K	11	nipponia_nippon	1,915,188	1,918,551	Unique
chicken:500K	11	columba_livia:ophisthocomus_hoazin:meleagris_gallopavo	2,655,301	2,704,647	Reuse
chicken:500K	11	ophisthocomus_hoazin:meleagris_gallopavo:columba_livia	2,655,301	2,704,647	Reuse
chicken:500K	11	meleagris_gallopavo:columba_livia:ophisthocomus_hoazin	2,655,301	2,704,647	Reuse
chicken:500K	11	pygoscelis_adeliae	5,711,060	5,714,402	Unique
chicken:500K	11	taeniopygia_guttata	5,982,285	5,993,811	Unique
chicken:500K	11	anolis_carolinensis	6,376,611	6,859,319	Unique
chicken:500K	11	cuculus_canorus	6,902,270	6,959,872	Unique
chicken:500K	11	manacus_vitellinus:anolis_carolinensis:columba_livia	7,364,048	7,381,880	Reuse
chicken:500K	11	anolis_carolinensis:columba_livia:manacus_vitellinus	7,364,048	7,381,880	Reuse
chicken:500K	11	columba_livia:manacus_vitellinus:anolis_carolinensis	7,364,048	7,381,880	Reuse
chicken:500K	11	charadrius_vociferus:ophisthocomus_hoazin	9,333,607	9,339,798	Reuse
chicken:500K	11	ophisthocomus_hoazin:charadrius_vociferus	9,333,607	9,339,798	Reuse
chicken:500K	11	anas_platyrhynchos	9,422,811	9,426,047	Unique
chicken:500K	11	egretta_garzetta	9,470,672	9,473,804	Unique

chicken:500K	11	python_molurus	9,583,258	9,696,069	Unique
chicken:500K	11	picoides_pubescens:birds_crocs_turtles	10,632,770	10,656,405	Reuse
chicken:500K	11	birds_crocs_turtles:picoides_pubescens	10,632,770	10,656,405	Reuse
chicken:500K	11	anas_platyrhynchos:taeniopygia_guttata	11,966,948	11,967,127	Reuse
chicken:500K	11	taeniopygia_guttata:anas_platyrhynchos	11,966,948	11,967,127	Reuse
chicken:500K	11	chrysemys_picta	12,156,113	12,180,667	Unique
chicken:500K	11	anolis_carolinensis	12,404,420	12,429,749	Unique
chicken:500K	11	passeriformes	13,422,171	13,521,153	Unique
chicken:500K	11	pygoscelis_adeliae	13,579,131	13,581,776	Unique
chicken:500K	11	anolis_carolinensis	13,986,639	14,000,710	Unique
chicken:500K	11	columba_livia	14,586,082	14,586,950	Unique
chicken:500K	11	picoides_pubescens	14,854,680	14,860,828	Unique
chicken:500K	11	ophisthocomus_hoazin	16,239,001	16,239,773	Unique
chicken:500K	11	taeniopygia_guttata	17,088,803	17,104,352	Unique
chicken:500K	11	melopsittacus_undulatus	17,903,320	18,372,530	Unique
chicken:500K	12	struthio_camelus	664,445	668,816	Unique
chicken:500K	12	meleagris_gallopavo	955,085	1,122,939	Unique
chicken:500K	12	taeniopygia_guttata	1,197,274	1,200,040	Unique
chicken:500K	12	chrysemys_picta:struthio_camelus	1,705,129	1,707,655	Reuse
chicken:500K	12	struthio_camelus:chrysemys_picta	1,705,129	1,707,655	Reuse
chicken:500K	12	falco_peregrinus:columba_livia	4,375,410	4,379,409	Reuse
chicken:500K	12	columba_livia:falco_peregrinus	4,375,410	4,379,409	Reuse
chicken:500K	12	anolis_carolinensis	5,449,787	5,722,957	Unique
chicken:500K	12	columba_livia:calypte_anna	5,877,180	5,893,702	Reuse
chicken:500K	12	calypte_anna:columba_livia	5,877,180	5,893,702	Reuse
chicken:500K	12	anas_platyrhynchos:opossum	8,582,113	8,597,309	Reuse
chicken:500K	12	opossum:anas_platyrhynchos	8,582,113	8,597,309	Reuse
chicken:500K	12	melopsittacus_undulatus:opossum:struthio_camelus	9,086,320	9,092,642	Reuse
chicken:500K	12	opossum:struthio_camelus:melopsittacus_undulatus	9,086,320	9,092,642	Reuse
chicken:500K	12	struthio_camelus:melopsittacus_undulatus:opossum	9,086,320	9,092,642	Reuse
chicken:500K	12	picoides_pubescens	11,227,412	11,231,272	Unique
chicken:500K	12	melopsittacus_undulatus	11,551,029	11,683,198	Unique
chicken:500K	12	cuculus_canorus:chrysemys_picta	11,979,190	11,987,050	Reuse
chicken:500K	12	chrysemys_picta:cuculus_canorus	11,979,190	11,987,050	Reuse
chicken:500K	12	anolis_carolinensis:struthio_camelus	12,933,015	12,936,217	Reuse
chicken:500K	12	struthio_camelus:anolis_carolinensis	12,933,015	12,936,217	Reuse
chicken:500K	12	melopsittacus_undulatus	13,605,943	13,607,116	Unique
chicken:500K	12	pygoscelis_adeliae	14,172,195	14,175,795	Unique
chicken:500K	12	chaetura_pelagica:anolis_carolinensis	14,330,677	14,336,569	Reuse
chicken:500K	12	anolis_carolinensis:chaetura_pelagica	14,330,677	14,336,569	Reuse
chicken:500K	12	struthio_camelus	15,305,398	15,310,490	Unique
chicken:500K	12	aptenodytes_forsteri	16,159,856	16,160,615	Unique
chicken:500K	12	picoides_pubescens	16,751,878	16,755,561	Unique
chicken:500K	12	chrysemys_picta	17,432,270	17,449,746	Unique
chicken:500K	13	sphenisciformes:anas_platyrhynchos:anolis_carolinensis:calypte_anna	1,099,253	1,111,239	Reuse
chicken:500K	13	anas_platyrhynchos:anolis_carolinensis:calypte_anna:sphenisciformes	1,099,253	1,111,239	Reuse
chicken:500K	13	anolis_carolinensis:calypte_anna:sphenisciformes:anas_platyrhynchos	1,099,253	1,111,239	Reuse
chicken:500K	13	calypte_anna:sphenisciformes:anas_platyrhynchos:anolis_carolinensis	1,099,253	1,111,239	Reuse
chicken:500K	13	columba_livia:passeroidea	2,655,460	2,658,550	Reuse
chicken:500K	13	passeroidea:columba_livia	2,655,460	2,658,550	Reuse
chicken:500K	13	calypte_anna	3,514,739	3,533,688	Unique
chicken:500K	13	calypte_anna:columba_livia	4,070,726	4,095,041	Reuse
chicken:500K	13	columba_livia:calypte_anna	4,070,726	4,095,041	Reuse
chicken:500K	13	picoides_pubescens:melopsittacus_undulatus	6,036,831	6,046,713	Reuse
chicken:500K	13	melopsittacus_undulatus:picoides_pubescens	6,036,831	6,046,713	Reuse
chicken:500K	13	manacus_vitellinus	6,082,360	6,117,838	Unique
chicken:500K	13	passeriformes	7,174,015	7,179,113	Unique
chicken:500K	13	calypte_anna	7,469,700	7,485,516	Unique
chicken:500K	13	columba_livia	7,515,964	7,720,606	Unique
chicken:500K	13	struthio_camelus	7,767,021	7,768,547	Unique
chicken:500K	13	anas_platyrhynchos	7,919,172	7,922,917	Unique
chicken:500K	13	anolis_carolinensis	8,632,070	8,651,981	Unique
chicken:500K	13	melopsittacus_undulatus	10,816,294	10,817,247	Unique
chicken:500K	13	chrysemys_picta	11,960,636	11,971,043	Unique
chicken:500K	13	birds_crocs_turtles	12,328,743	12,343,054	Unique
chicken:500K	13	struthio_camelus	12,462,337	12,467,832	Unique

chicken:500K	13	anolis_carolinensis	13,847,048	14,316,699	Unique
chicken:500K	13	anolis_carolinensis	15,098,457	15,106,937	Unique
chicken:500K	13	chrysemys_picta	15,330,416	15,337,387	Unique
chicken:500K	13	egretta_garzetta	15,865,413	15,866,065	Unique
chicken:500K	13	cuculus_canorus	15,966,691	15,973,874	Unique
chicken:500K	13	columba_livia	16,162,870	16,166,111	Unique
chicken:500K	13	pygoscelis_adeliae	16,331,160	16,335,126	Unique
chicken:500K	13	passeriformes	16,442,109	16,449,831	Unique
chicken:500K	13	cuculus_canorus:opossum	16,546,835	16,553,197	Reuse
chicken:500K	13	opossum:cuculus_canorus	16,546,835	16,553,197	Reuse
chicken:500K	14	galliformes	937,476	938,425	Unique
chicken:500K	14	ophisthocomus_hoazin:python_molurus	2,257,536	2,262,242	Reuse
chicken:500K	14	python_molurus:ophisthocomus_hoazin	2,257,536	2,262,242	Reuse
chicken:500K	14	python_molurus	4,648,820	4,665,036	Unique
chicken:500K	14	python_molurus:egretta_garzetta	5,544,062	5,545,003	Reuse
chicken:500K	14	egretta_garzetta:python_molurus	5,544,062	5,545,003	Reuse
chicken:500K	14	corvus_brachyrhynchos	7,260,399	7,262,151	Unique
chicken:500K	14	galliformes	7,458,779	7,459,817	Unique
chicken:500K	14	taeniopygia_guttata	8,319,574	8,323,624	Unique
chicken:500K	14	galloanserae	8,900,214	8,905,370	Unique
chicken:500K	14	columba_livia	9,806,105	9,822,187	Unique
chicken:500K	14	anas_platyrhynchos	10,013,943	10,015,219	Unique
chicken:500K	14	calypte_anna	10,111,015	10,137,040	Unique
chicken:500K	14	melopsittacus_undulatus	11,443,743	11,445,309	Unique
chicken:500K	14	anas_platyrhynchos	11,771,133	11,783,564	Unique
chicken:500K	14	struthio_camelus	11,784,001	11,785,184	Unique
chicken:500K	14	nipponia_nippon	12,301,965	12,306,775	Unique
chicken:500K	14	melopsittacus_undulatus	12,685,505	12,732,885	Unique
chicken:500K	14	cuculus_canorus:opossum	14,448,014	14,460,580	Reuse
chicken:500K	14	opossum:cuculus_canorus	14,448,014	14,460,580	Reuse
chicken:500K	15	chrysemys_picta:columba_livia:opossum	1,413,015	1,415,700	Reuse
chicken:500K	15	columba_livia:opossum:chrysemys_picta	1,413,015	1,415,700	Reuse
chicken:500K	15	opossum:chrysemys_picta:columba_livia	1,413,015	1,415,700	Reuse
chicken:500K	15	passeroidea + corvoidea	2,705,003	2,710,900	Unique
chicken:500K	15	melopsittacus_undulatus	3,343,726	3,353,807	Unique
chicken:500K	15	calypte_anna	3,465,155	3,479,161	Unique
chicken:500K	15	anas_platyrhynchos	4,411,747	4,506,261	Unique
chicken:500K	15	chinese_alligator	5,252,656	5,264,122	Unique
chicken:500K	15	egretta_garzetta	5,592,766	5,596,355	Unique
chicken:500K	15	chinese_alligator:falco_peregrinus	6,829,488	6,924,047	Reuse
chicken:500K	15	falco_peregrinus:chinese_alligator	6,829,488	6,924,047	Reuse
chicken:500K	15	galliformes	7,846,314	7,848,556	Unique
chicken:500K	15	cuculus_canorus:picoides_pubescens	9,254,385	9,257,275	Reuse
chicken:500K	15	picoides_pubescens:cuculus_canorus	9,254,385	9,257,275	Reuse
chicken:500K	15	galliformes	9,909,430	9,910,262	Unique
chicken:500K	15	struthio_camelus	10,487,534	10,591,127	Unique
chicken:500K	15	anolis_carolinensis	11,161,808	11,201,515	Unique
chicken:500K	17	calypte_anna	2,871,262	2,877,534	Unique
chicken:500K	17	anas_platyrhynchos	4,993,187	4,994,737	Unique
chicken:500K	17	calypte_anna	6,169,421	6,644,566	Unique
chicken:500K	17	melopsittacus_undulatus:anas_platyrhynchos	7,716,805	7,720,537	Reuse
chicken:500K	17	anas_platyrhynchos:melopsittacus_undulatus	7,716,805	7,720,537	Reuse
chicken:500K	17	chrysemys_picta	9,200,662	9,208,405	Unique
chicken:500K	18	birds_crocs_turtles	1,000,267	1,015,247	Unique
chicken:500K	18	melopsittacus_undulatus	2,197,952	2,212,185	Unique
chicken:500K	18	picoides_pubescens	2,977,071	2,978,963	Unique
chicken:500K	18	python_molurus:chaetura_pelagica:anolis_carolinensis	3,331,503	3,345,661	Reuse
chicken:500K	18	chaetura_pelagica:anolis_carolinensis:python_molurus	3,331,503	3,345,661	Reuse
chicken:500K	18	anolis_carolinensis:python_molurus:chaetura_pelagica	3,331,503	3,345,661	Reuse
chicken:500K	18	chicken	5,035,833	5,038,911	Unique
chicken:500K	18	chrysemys_picta	5,678,684	5,683,509	Unique
chicken:500K	18	columba_livia	6,003,591	6,012,147	Unique
chicken:500K	18	calypte_anna	6,249,462	6,255,483	Unique
chicken:500K	18	taeniopygia_guttata	6,541,531	6,543,304	Unique
chicken:500K	18	anas_platyrhynchos	6,961,049	6,965,328	Unique
chicken:500K	18	taeniopygia_guttata	8,250,097	8,460,549	Unique

chicken:500K	18	anolis_carolinensis	9,189,826	9,244,180	Unique
chicken:500K	18	chicken	10,250,901	10,252,269	Unique
chicken:500K	19	chrysemys_picta	2,451,348	2,463,993	Unique
chicken:500K	19	nipponia_nippon	2,615,152	2,617,239	Unique
chicken:500K	19	cuculus_canorus	3,131,482	3,134,678	Unique
chicken:500K	19	python_molurus	3,208,604	3,209,047	Unique
chicken:500K	19	melopsittacus_undulatus	3,410,958	3,416,346	Unique
chicken:500K	19	python_molurus	4,171,937	4,183,215	Unique
chicken:500K	19	nipponia_nippon	4,793,009	4,798,436	Unique
chicken:500K	19	melopsittacus_undulatus	5,017,036	5,021,729	Unique
chicken:500K	19	python_molurus	5,048,828	5,128,350	Unique
chicken:500K	19	calypte_anna	5,337,152	5,338,106	Unique
chicken:500K	19	melopsittacus_undulatus	5,745,795	5,950,769	Unique
chicken:500K	19	picoides_pubescens	6,306,946	6,313,159	Unique
chicken:500K	19	melopsittacus_undulatus	6,616,426	6,625,487	Unique
chicken:500K	19	chrysemys_picta	7,072,415	7,074,641	Unique
chicken:500K	19	melopsittacus_undulatus	7,183,359	7,406,182	Unique
chicken:500K	19	picoides_pubescens:opossum	8,082,390	8,086,089	Reuse
chicken:500K	19	opossum:picoides_pubescens	8,082,390	8,086,089	Reuse
chicken:500K	19	melopsittacus_undulatus	8,164,070	8,168,121	Unique
chicken:500K	19	pygoscelis_adeliae	8,246,490	8,248,115	Unique
chicken:500K	20	python_molurus	584,606	596,162	Unique
chicken:500K	20	Passeriformes + Psittaciformes + Falconiformes	1,333,670	1,408,720	Unique
chicken:500K	20	falco_peregrinus	2,912,249	2,917,570	Unique
chicken:500K	20	anolis_carolinensis	4,827,323	4,853,153	Unique
chicken:500K	20	melopsittacus_undulatus	5,360,290	5,365,107	Unique
chicken:500K	20	anolis_carolinensis	5,489,432	5,490,512	Unique
chicken:500K	20	calypte_anna	5,599,490	5,730,428	Unique
chicken:500K	20	passeriformes	6,166,901	6,184,998	Unique
chicken:500K	20	galliformes	7,001,727	7,075,274	Unique
chicken:500K	20	melopsittacus_undulatus	8,006,032	8,020,568	Unique
chicken:500K	20	falco_peregrinus	8,748,268	9,173,608	Unique
chicken:500K	20	galloanserae	11,104,293	11,105,591	Unique
chicken:500K	20	charadrius_vociferus	11,924,949	11,925,766	Unique
chicken:500K	20	chrysemys_picta	12,080,537	12,086,867	Unique
chicken:500K	20	chrysemys_picta	13,545,923	13,725,161	Unique
chicken:500K	21	calypte_anna	1,410,962	1,411,557	Unique
chicken:500K	21	picoides_pubescens	1,742,633	1,746,443	Unique
chicken:500K	21	manacus_vitellinus	1,973,004	1,975,004	Unique
chicken:500K	21	anas_platyrhynchos	2,040,160	2,069,200	Unique
chicken:500K	21	chinese_alligator	2,069,546	2,083,439	Unique
chicken:500K	21	taeniopygia_guttata	2,446,661	2,451,283	Unique
chicken:500K	21	calypte_anna	2,510,908	2,526,321	Unique
chicken:500K	21	falco_peregrinus	2,544,212	3,035,731	Unique
chicken:500K	21	aptenodytes_forsteri	3,112,908	3,114,261	Unique
chicken:500K	21	anas_platyrhynchos:calypte_anna	3,446,651	3,456,223	Reuse
chicken:500K	21	calypte_anna:anas_platyrhynchos	3,446,651	3,456,223	Reuse
chicken:500K	21	non_galloanserae	4,201,222	4,202,505	Unique
chicken:500K	21	struthio_camelus:passeriformes	4,923,548	4,925,686	Reuse
chicken:500K	21	passeriformes:struthio_camelus	4,923,548	4,925,686	Reuse
chicken:500K	21	galloanserae	5,833,290	5,834,593	Unique
chicken:500K	22	chrysemys_picta:passeroidea	1,690,414	1,842,704	Reuse
chicken:500K	22	passeroidea:chrysemys_picta	1,690,414	1,842,704	Reuse
chicken:500K	22	anas_platyrhynchos:chrysemys_picta:cuculus_canorus	2,750,346	3,021,382	Reuse
chicken:500K	22	chrysemys_picta:cuculus_canorus:anas_platyrhynchos	2,750,346	3,021,382	Reuse
chicken:500K	22	cuculus_canorus:anas_platyrhynchos:chrysemys_picta	2,750,346	3,021,382	Reuse
chicken:500K	24	taeniopygia_guttata	655,637	661,276	Unique
chicken:500K	24	anolis_carolinensis:egretta_garzetta	1,409,081	1,418,698	Reuse
chicken:500K	24	egretta_garzetta:anolis_carolinensis	1,409,081	1,418,698	Reuse
chicken:500K	24	passeroidea + corvoidea	1,587,957	1,594,703	Unique
chicken:500K	24	manacus_vitellinus	1,791,493	1,794,698	Unique
chicken:500K	24	struthio_camelus	1,799,944	1,803,933	Unique
chicken:500K	24	chrysemys_picta	2,110,625	2,572,205	Unique
chicken:500K	24	melopsittacus_undulatus:opossum	2,826,835	2,832,754	Reuse
chicken:500K	24	opossum:melopsittacus_undulatus	2,826,835	2,832,754	Reuse
chicken:500K	24	taeniopygia_guttata	2,899,692	2,907,440	Unique

chicken:500K	24	egretta_garzetta	3,073,782	3,286,913	Unique
chicken:500K	24	columba_livia	3,782,241	3,789,548	Unique
chicken:500K	24	galloanserae	4,335,317	4,338,440	Unique
chicken:500K	24	python_molurus:manacus_vitellinus:opossum	5,700,925	5,708,862	Reuse
chicken:500K	24	manacus_vitellinus:opossum:python_molurus	5,700,925	5,708,862	Reuse
chicken:500K	24	opossum:python_molurus:manacus_vitellinus	5,700,925	5,708,862	Reuse
chicken:500K	25	meleagris_gallopavo	1,366,135	1,387,679	Unique
chicken:500K	26	columba_livia	1,056,366	1,062,734	Unique
chicken:500K	26	Ciconiiformes + Sphenisciformes:falco_peregrinus:charadrius_vociferus	1,618,004	1,620,819	Reuse
chicken:500K	26	falco_peregrinus:charadrius_vociferus:Ciconiiformes + Sphenisciformes	1,618,004	1,620,819	Reuse
chicken:500K	26	charadrius_vociferus:Ciconiiformes + Sphenisciformes:falco_peregrinus	1,618,004	1,620,819	Reuse
chicken:500K	26	anolis_carolinensis	2,589,751	2,976,621	Unique
chicken:500K	26	columba_livia	3,183,469	3,418,980	Unique
chicken:500K	26	anolis_carolinensis	4,671,499	4,687,281	Unique
chicken:500K	27	taeniopygia_guttata	837,886	1,048,658	Unique
chicken:500K	27	taeniopygia_guttata	1,549,207	1,590,407	Unique
chicken:500K	27	struthio_camelus	1,662,631	1,667,249	Unique
chicken:500K	27	cuculus_canorus	1,693,185	1,695,062	Unique
chicken:500K	27	anas_platyrhynchos	1,974,080	2,199,495	Unique
chicken:500K	27	cuculus_canorus	2,298,926	2,303,220	Unique
chicken:500K	27	anolis_carolinensis:python_molurus	4,012,023	4,123,959	Reuse
chicken:500K	27	python_molurus:anolis_carolinensis	4,012,023	4,123,959	Reuse
chicken:500K	28	meleagris_gallopavo:taeniopygia_guttata	1,117,235	1,525,445	Reuse
chicken:500K	28	taeniopygia_guttata:meleagris_gallopavo	1,117,235	1,525,445	Reuse
chicken:500K	28	anas_platyrhynchos:corvus_brachyrhynchos	2,406,536	2,571,594	Reuse
chicken:500K	28	corvus_brachyrhynchos:anas_platyrhynchos	2,406,536	2,571,594	Reuse
chicken:500K	28	chrysemys_picta:opossum	4,200,683	4,209,256	Reuse
chicken:500K	28	opossum:chrysemys_picta	4,200,683	4,209,256	Reuse
chicken:500K	Z	aptenodytes_forsteri:opossum	979,285	1,094,355	Reuse
chicken:500K	Z	opossum:aptenodytes_forsteri	979,285	1,094,355	Reuse
chicken:500K	Z	anolis_carolinensis	1,680,442	1,724,562	Unique
chicken:500K	Z	taeniopygia_guttata	1,812,765	1,822,812	Unique
chicken:500K	Z	melopsittacus_undulatus	2,272,653	2,280,566	Unique
chicken:500K	Z	ophisthocomus_hoazin	4,048,204	4,054,715	Unique
chicken:500K	Z	anolis_carolinensis	4,294,217	4,323,596	Unique
chicken:500K	Z	melopsittacus_undulatus	5,340,642	5,344,172	Unique
chicken:500K	Z	anas_platyrhynchos	5,862,360	6,160,056	Unique
chicken:500K	Z	melopsittacus_undulatus	8,914,063	9,028,447	Unique
chicken:500K	Z	cuculus_canorus	9,924,640	9,927,805	Unique
chicken:500K	Z	charadrius_vociferus	9,958,193	9,963,494	Unique
chicken:500K	Z	cuculus_canorus	10,901,016	10,961,395	Unique
chicken:500K	Z	manacus_vitellinus	11,114,924	11,130,427	Unique
chicken:500K	Z	falco_peregrinus:anolis_carolinensis	12,839,311	12,919,881	Reuse
chicken:500K	Z	anolis_carolinensis:falco_peregrinus	12,839,311	12,919,881	Reuse
chicken:500K	Z	manacus_vitellinus	13,143,856	13,169,663	Unique
chicken:500K	Z	chrysemys_picta:chinese_alligator:charadrius_vociferus	16,782,557	16,791,441	Reuse
chicken:500K	Z	chinese_alligator:charadrius_vociferus:chrysemys_picta	16,782,557	16,791,441	Reuse
chicken:500K	Z	charadrius_vociferus:chrysemys_picta:chinese_alligator	16,782,557	16,791,441	Reuse
chicken:500K	Z	chinese_alligator:anolis_carolinensis:egretta_garzetta	17,898,245	17,903,166	Reuse
chicken:500K	Z	anolis_carolinensis:egretta_garzetta:chinese_alligator	17,898,245	17,903,166	Reuse
chicken:500K	Z	egretta_garzetta:chinese_alligator:anolis_carolinensis	17,898,245	17,903,166	Reuse
chicken:500K	Z	falco_peregrinus	18,478,315	18,544,132	Unique
chicken:500K	Z	chrysemys_picta	19,248,033	19,302,284	Unique
chicken:500K	Z	birds_crocs_turtles	19,414,315	19,853,613	Unique
chicken:500K	Z	corvus_brachyrhynchos	20,972,795	20,981,761	Unique
chicken:500K	Z	struthio_camelus	21,487,641	21,492,981	Unique
chicken:500K	Z	passeroidea + corvoidea	21,600,514	21,820,648	Unique
chicken:500K	Z	melopsittacus_undulatus	22,287,753	22,295,551	Unique
chicken:500K	Z	ophisthocomus_hoazin:opossum	23,189,406	23,357,914	Reuse
chicken:500K	Z	opossum:ophisthocomus_hoazin	23,189,406	23,357,914	Reuse
chicken:500K	Z	taeniopygia_guttata	23,642,099	23,650,576	Unique
chicken:500K	Z	neoavians	25,352,141	25,356,301	Unique
chicken:500K	Z	struthio_camelus	26,888,103	26,895,919	Unique
chicken:500K	Z	columba_livia	26,996,220	27,341,257	Unique
chicken:500K	Z	anolis_carolinensis	28,916,020	30,816,623	Unique
chicken:500K	Z	manacus_vitellinus	31,676,732	31,690,177	Unique

chicken:500K	Z	struthio_camelus	32,461,385	32,462,900	Unique
chicken:500K	Z	cuculus_canorus	33,012,869	33,017,315	Unique
chicken:500K	Z	chaetura_pelagica	33,292,535	33,294,102	Unique
chicken:500K	Z	struthio_camelus	34,804,736	35,168,928	Unique
chicken:500K	Z	corvus_brachyrhynchos	35,540,785	35,545,313	Unique
chicken:500K	Z	struthio_camelus	35,704,684	36,600,739	Unique
chicken:500K	Z	nipponia_nippon	37,148,361	37,163,331	Unique
chicken:500K	Z	passeriformes	37,182,429	37,190,619	Unique
chicken:500K	Z	columba_livia:opossum	37,194,831	37,197,546	Reuse
chicken:500K	Z	opossum:columba_livia	37,194,831	37,197,546	Reuse
chicken:500K	Z	charadrius_vociferus	38,863,464	38,872,578	Unique
chicken:500K	Z	chrysemys_picta:egretta_garzetta:cuculus_canorus	39,647,312	39,669,436	Reuse
chicken:500K	Z	egretta_garzetta:cuculus_canorus:chrysemys_picta	39,647,312	39,669,436	Reuse
chicken:500K	Z	cuculus_canorus:chrysemys_picta:egretta_garzetta	39,647,312	39,669,436	Reuse
chicken:500K	Z	chaetura_pelagica:opossum:taeniopygia_guttata	40,681,332	40,681,671	Reuse
chicken:500K	Z	opossum:taeniopygia_guttata:chaetura_pelagica	40,681,332	40,681,671	Reuse
chicken:500K	Z	taeniopygia_guttata:chaetura_pelagica:opossum	40,681,332	40,681,671	Reuse
chicken:500K	Z	anas_platyrhynchos:opossum	42,933,766	42,995,895	Reuse
chicken:500K	Z	opossum:anas_platyrhynchos	42,933,766	42,995,895	Reuse
chicken:500K	Z	birds_crocs_turtles:anas_platyrhynchos	43,745,812	43,886,883	Reuse
chicken:500K	Z	anas_platyrhynchos:birds_crocs_turtles	43,745,812	43,886,883	Reuse
chicken:500K	Z	galliformes	45,523,252	45,523,597	Unique
chicken:500K	Z	chrysemys_picta	49,154,029	49,165,405	Unique
chicken:500K	Z	ophisthocomus_hoazin	49,860,775	49,866,122	Unique
chicken:500K	Z	columba_livia	50,619,010	51,067,128	Unique
chicken:500K	Z	melopsittacus_undulatus	52,396,104	52,434,452	Unique
chicken:500K	Z	charadrius_vociferus:columba_livia	52,766,676	52,770,272	Reuse
chicken:500K	Z	columba_livia:charadrius_vociferus	52,766,676	52,770,272	Reuse
chicken:500K	Z	cuculus_canorus	53,402,670	53,405,606	Unique
chicken:500K	Z	corvus_brachyrhynchos	53,818,831	54,161,501	Unique
chicken:500K	Z	chrysemys_picta	54,473,359	54,530,403	Unique
chicken:500K	Z	anolis_carolinensis	54,596,800	54,761,360	Unique
chicken:500K	Z	passeriformes	54,824,372	54,824,429	Unique
chicken:500K	Z	struthio_camelus	55,990,603	56,006,779	Unique
chicken:500K	Z	galliformes	56,821,813	56,827,515	Unique
chicken:500K	Z	passeriformes	60,954,531	60,985,440	Unique
chicken:500K	Z	galloanserae	64,005,006	64,098,619	Unique
chicken:500K	Z	Passeriformes + Psittaciformes + Falconiformes + Piciformes	65,319,293	65,323,872	Unique
chicken:500K	Z	picoides_pubescens:opossum	66,298,329	66,326,285	Reuse
chicken:500K	Z	opossum:picoides_pubescens	66,298,329	66,326,285	Reuse
chicken:500K	Z	neoavians	66,941,666	66,948,009	Unique
chicken:500K	Z	struthio_camelus	67,454,337	67,504,692	Unique
chicken:500K	Z	struthio_camelus	68,069,168	68,075,403	Unique
chicken:500K	Z	aptenodytes_forsteri	69,580,942	69,584,173	Unique
chicken:500K	Z	melopsittacus_undulatus	69,916,741	69,920,985	Unique
chicken:500K	Z	galloanserae	70,824,374	70,827,839	Unique
chicken:500K	Z	galloanserae	72,985,809	73,023,355	Unique
chicken:500K	Z	charadrius_vociferus	78,573,617	78,575,593	Unique
chicken:500K	Z	passeroidea:egret_ibis	78,740,986	78,748,138	Reuse
chicken:500K	Z	egret_ibis:passeroidea	78,740,986	78,748,138	Reuse
chicken:500K	Z	galloanserae	79,987,451	80,647,861	Unique
chicken:500K	Z	Ciconiiformes + Sphenisciformes	81,350,113	81,364,283	Unique
chicken:500K	Z	geospiza_fortis	81,463,790	81,479,690	Unique
