# Supplementary material for: Novel Insights into Chromosome Evolution in Birds, Archosaurs, and Reptiles
Source: Genome Biol Evol. 2016 Jul 10;8(8):2442–51. doi: 10.1093/gbe/evw166 (PMC5010900; doi:10.1093/gbe/evw166)
Supplement: Supplementary Data [file supp_evw166_Supplementary_file_GBE_afterRevision.pdf]

Novel insights into chromosome evolution in birds, archosaurs, and reptiles.

Marta Farré[1], Jitendra Narayan[2], Gancho T. Slavov[2], Joana Damas[1], Loretta Auvil[3], Cai Li[4,5], Erich D. Jarvis[6], David W. Burt[7], Darren K. Griffin[8§], Denis M. Larkin[1§*]

[1]Department of Comparative Biomedical Sciences, Royal Veterinary College, Royal College Street, NW1 0TU, University of London, London, UK

[2]Institute of Biological, Environmental and Rural Sciences, Aberystwyth University, Penglais Campus, SY23 3FL, Aberystwyth, UK

[3]Illinois Informatics Institute, University of Illinois, IL 61801, Urbana, Illinois, USA

[4]China National GeneBank, BGI-Shenzhen, 518083, Shenzhen, China

[5]Centre for GeoGenetics, Natural History Museum of Denmark, University of Copenhagen, 1350 Copenhagen, Denmark

[6]Department of Neurobiology, Howard Hughes Medical Institute, Duke University Medical Center, MD 20815, Durham, USA

[7]Department of Genomics and Genetics, The Roslin Institute and Royal (Dick) School of Veterinary Studies, University of Edinburgh, EH25 9RG, Midlothian, UK

[8]School of Biosciences, University of Kent, CT2 7NY, Canterbury, UK

Supplementary Data and Methods

1. Identification of syntenic fragments.

Twenty avian, four reptile and one mammalian genome assemblies with scaffold N50 > 2Mbp or assembled at chromosome level (Suppl. Table 1) were aligned against the chicken chromosome sequences using the SatsumaSynteny program (Grabherr, et al. 2010). The pairwise alignments were cleaned from overlapping and duplicated matches and syntenic fragments (SFs) defined using SyntenyTracker (Donthu, et al. 2009). We used sets of parameters that allowed the detection of genome rearrangements that are ≥ 500Kbp, ≥ 300Kbp, ≥ 100Kbp in the chicken chromosome sequences. The SFs found in the genomes assembled to chromosomes represent complete HSBs, while those found in fragmented assemblies may often be partial synteny blocks (Fig. 1).

Supplementary Table 1. Species and assemblies used in this study.

Species	Scientific name	Accession number	Scaffold N50 (Mbp)	Reference
Chicken	*Gallus gallus*	GCA_000002315.2	Chromosomes	(ICGSC 2004)
Common cuckoo	*Cuculus canorus*	GCA_000709325.1	3.0	(Zhang, et al. 2014a)
American crow	*Corvus brachyrhynchos*	GCA_000691975.1	6.9	"
Little egret	*Egretta garzetta*	GCA_000687185.1	3.1	"
Crested ibis	*Nipponia nippon*	GCA_000708225.1	5.4	"
Domestic pigeon	*Columba livia*	GCA_000337935.1	3.2	"
Hoatzin	*Opisthocomus hoazin*	GCA_000692075.1	2.9	"
Golden-collared manakin	*Manacus vitellinus*	GCA_000692015.2	2.5	"
Medium-ground finch	*Geospiza fortis*	GCA_000277835.1	5.2	"
Downy woodpecker	*Picoides pubescens*	GCA_000699005.1	2.0	"
Adelie penguin	*Pygoscelis adeliae*	GCA_000699105.1	5.0	"
Emperor penguin	*Aptenodytes forsteri*	GCA_000699145.1	5.1	"
Anna's hummingbird	*Calypte anna*	GCA_000699085.1	4.0	"
Chimney swift	*Chaetura pelagica*	GCA_000747805.1	3.8	"
Killdeer	*Charadrius vociferous*	GCA_000708025.2	3.6	"
Peregrine falcon	*Falco peregrinus*	GCA_000337955.1	3.9	(Zhan, et al. 2013)
Ostrich	*Struthio camelus*	GCA_000698965.1	3.5	"
Pekin duck	*Anas platyrhyncos*	N/A	Pseudochromosomes	*
Budgerigar	*Melopsittacus undulatus*	N/A	Pseudochromosomes	(Ganapathy, et al. 2014)
Turkey	*Meleagris gallopavo*	GCA_000146605.2	Chromosomes	(Dalloul, et al. 2010)
Zebra finch	*Taenopygia guttata*	GCA_000151805.2	Chromosomes	(Warren, et al. 2010)
Anole lizard	*Anolis carolinensis*	GCA_000090745.2	Chromosomes	(Alföldi, et al. 2011)
Boa constrictor	*Boa constrictor*	5C	3.8	(Bradnam, et al. 2013)
Painted turtle	*Chrysemys picta*	GCA_000241765.1	5.2	(Shaffer, et al. 2013)
Chinese alligator	*Alligator sinensis*	GCA_000455745.1	2.2	(Wan, et al. 2013)
Opossum	*Monodelphis domestica*	GCF_000002295.2	Chromosomes	(Mikkelsen, et al. 2007)

* We obtained the duck agp file based on the radiation hybrid map from T. Faraut and A. Vignal and used this information to order and orient duck scaffolds on chromosomes. ** We used the budgerigar genome assembled at megascaffold level with the aid of an optical map.

2. Identification and classification of EBRs using a newly-developed algorithm

We used a new multistep approach to detect and classify evolutionary breakpoint regions (EBRs). First, we used a *Perl* script to identify all potential breakpoint regions (BRs) as the intervals between two adjacent SFs in the reference genome chromosomes. This was done separately for each SF set at each resolution of SF detection. If a target genome was not assembled to the chromosomal level, only BRs found within the scaffolds of the target species were classified as EBRs at the final step. Second, all BRs selected in the previous step (found in between HSB boundaries or within scaffolds) from all target genomes from the same SF set were cross-compared for reference genome coordinate overlaps. A target genome selected BR that overlapped with more than one non-overlapping selected BR in any other target genome(s) was treated as a *gap* and genomes containing gaps at any reference chromosome position were excluded from classification of EBRs at that position. All intervals in a reference genome chromosome between adjacent scaffolds from a single target genome that overlapped a selected BR in any other target genome were treated as gaps as well.

Breakpoint regions were assigned to phylogenetic lineages using the total evidence nucleotide tree (TENT; (Jarvis, et al. 2014), retaining only the branches leading to species used in the BR analysis (Fig. 2) and the outgroup species. We performed phylogenetic classification of BRs and, where justified, assigned EBR status using an *ad hoc* likelihood ratio approach. Assuming that the detection of BRs was statistically independent across genomes, we calculated the likelihood of any given hypothesis (H_i) regarding the phylogenetic classification of a BR as:

$$L(H_i) = \prod_{j=1}^{n} P_{ij}(D_j|H_i),$$
,

where $P_{ij}(D_j|H_i)$ was the conditional probability of occurrence of the observed data in species j (D_j), assuming that H_i was correct. The probability $P_{ij}(D_j|H_i)$ was assigned one of four possible values, each corresponding to one of four mutually exclusive events:

$$P_{ij}(D_j|H_i) = \begin{cases} \beta_j, \\ 1-\beta_j, \\ R_{jk}, or \\ 1 \end{cases}$$

The first probability, β_j, or the probability of failing to detect a BR, was assigned when the occurrence of a BR in species j was expected under hypothesis H_i, but no BR was detected. The second probability (1 - β_j) corresponded to the opposite event (i.e., when the occurrence of a BR in species j was expected under hypothesis H_i, and a BR was indeed detected). The third probability, R_{jk}, or the probability of random overlap between a BR in species j and interval of interest k, was assigned when no BR was expected in species j under hypothesis H_i, but a BR was detected. Finally, when no BR was expected or detected, a value of one was assigned.

We estimated β_j for each species and resolution of BR detection by cross-referencing BRs detected for that species at higher and lower resolutions. More specifically, the estimate of β_j for any intermediate resolution was calculated as the proportion of BRs that were not detected at that resolution, but were detected at both higher and lower resolutions. For the highest resolution, β_j was calculated as the proportion of BRs that were not detected at that resolution but were detected at two lower resolutions. For the lowest resolution, the value of β_j was extrapolated using a general regression neural network algorithm (Specht 1991) on β_j values from higher resolutions. The probability of random overlap between a BR and a genome region of interest (R_{jk}) was approximated using a non-homogeneous Poisson process (Ross 1996). To parameterize the process, we first grouped all BRs in size classes, then calculated the rate of occurrence of each class in each genome, and finally approximated R_{jk} as:

$$R_{jk} \approx \lambda_{L(j)}(L_j + M_k),$$

where $\lambda_{L(j)}$ was the rate of occurrence of BRs from size class L in species j, L_j was the average size of BRs from class L in species j, and M_k was the size of the genome region of interest k.

After likelihoods were calculated for all possible hypotheses, ratios were calculated between the likelihoods of the first and second most likely hypotheses. These likelihood ratios were used as a quantitative basis for assigning BRs to phylogenetic branches, thereby qualifying them as EBRs, and distinguishing EBRs from BRs that could not be unambiguously assigned to a phylogenetic branch (e.g., *uncertain* BRs for which the two most likely hypotheses had equal likelihoods). In addition, we classified those EBRs that overlapped in one or more species from different phylogenetic nodes as *reuse* EBRs.

3. Testing the algorithmic approach of EBR detection using a published EBR set

To test if our algorithm is classifying EBRs in sequenced animal genomes correctly, we applied our new methodology to the previously published cattle genome dataset that had EBRs identified (Elsik, et al. 2009). To identify EBRs in the cattle genome, we aligned human (hg19), rhesus macaque (rheMac2), dog (canFam4), mouse (mmu9), and pig (susScr3) chromosome assemblies to cattle chromosomes (UMD3.1) using the SatsumaSynteny program (Grabherr, et al. 2010). We defined HSBs at three resolutions (≥ 100Kbp, ≥ 300Kbp, and ≥ 500Kbp) using SyntenyTracker (Donthu, et al. 2009). And finally, we applied our algorithm to detect and classify EBRs in the cattle genome using the following topology: (((human, rhesus), mouse), (dog, (cattle, pig))). We performed two EBR classifications: (i) using EBR intervals exactly as they were defined from the HSBs sets, and (ii) allowing EBR intervals to be extended by 20Kbp. The extended set could potentially allow for the detection of additional EBRs in the lineage leading to cattle in the regions of the reference genome, where exact identification of HSB boundaries was complicated due to duplications or local

misalignments. We then translated published manually defined cattle lineage-specific EBR coordinates from the Btau4.0 assembly (Elsik, et al. 2009) to the UMD3.1 assembly used in our analysis. This was done using the UCSC Genome Browser *LiftOver* tool (Kent, et al. 2002).

Out of the 100 cattle lineage-specific EBRs identified in the Btau4.0 assembly, 98 were successfully translated to the UMD3.1 coordinates. We excluded the EBRs found on cattle chromosome X (BTAX) from the comparison because the Btau4.0 assembly had a very incomplete BTAX assembly (Partipilo, et al. 2011). The translated UMD3.1 coordinates of the remaining 90 cattle lineage-specific autosomal EBRs were compared to the EBRs detected by our algorithm. Out of the 90 EBRs, our algorithm classified 76 (84.44%) as cattle lineage-specific in the non-extended EBR set at the same resolution of HSBs detection (500Kbp). When we allowed the EBR intervals to be extended by 20Kbp, 86 (95.55%) of the 90 cattle lineage-specific EBRs were identified. In the extended set, the remaining four EBRs were reported as *gaps* and were excluded from the EBR classification step (e.g., Suppl. Fig. 1). As expected, in the extended set we observed a decrease in the number of lineage-specific EBRs compared to the non-extended set (up to 25% for the rhesus lineage-specific EBRs) and also a higher fraction of EBRs was classified as reuse (8% in the extended set vs. 7% in the non-extended set) (Suppl. Table 2).

While the extension of EBR intervals may help recovering additional reference-specific EBRs (11% in our set), it also leads to the overestimation of the number of reuse EBRs and to the underestimation of the number of lineage-specific EBRs. Such problematic EBRs would need to be carefully verified using fluorescence *in situ* hybridization, PCR or other techniques. Therefore, in our bioinformatic analysis of the avian chromosomal rearrangements we chose to be conservative and not to extend EBR boundaries.

In our comparison of the Btau4.0 and UMD3.1 assemblies, we identified 35 additional cattle lineage-specific EBRs that were not reported in the Btau4.0 (Elsik, et al. 2009). To trace their origin, we translated the sequence coordinates of Btau4.0 using the *LiftOver* tool. Twenty-nine out of the 35 additional EBRs did not match a synteny break in Btau4.0 compared to other species. The 29 EBRs represent EBRs that result from differences between Btau4.0 and UMD3.1 or differences in the methodology of genome comparisons: the cattle genome comparison (Elsik, et al. 2009) was performed using the alignment of a limited number of cattle BAC-end sequences against other species while in our analysis we used complete whole-genome sequence alignments. Six of the 35 additional EBRs were not reported previously as EBRs in the pig genome, and were therefore classified as artiodactyl- rather than cattle lineage-specific in the cattle genome paper. These six EBRs result from differences between the pig genome assembly (susScr3) used in our analysis and the pig physical map used in the cattle genome paper (Elsik, et al. 2009).

Supplementary Table 2. Comparison of automated approach of EBR detection to previously published, manually-defined cattle lineage-specific EBRs.

Category	Not extended	Extended 20Kbp
No. autosomal cattle lineage-specific EBRs from (Elsik, et al. 2009)	90	90
No. cattle lineage-specific EBRs overlapping with published data	76	86
Overlap (%)	84.44	95.55
Cattle lineage-specific EBRs	119	126
Total EBRs	603	577
Reuse EBRs (%)	42(7%)	47(8%)

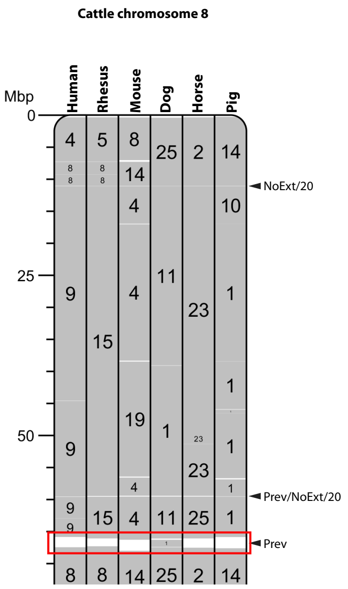

Supplementary Figure 1. Comparison of the algorithmic approach to manually defined cattle lineage-specific EBR set. Cattle chromosome 8 showing the EBRs previously detected and published (Prev), newly detected EBRs not extending the boundaries (NoExt) and extending by 20Kbp (20). The red rectangle demarcates an example of EBR classified as a "gap" by our algorithm.

4. Testing the algorithmic approach to detect EBRs using simulated rearrangements

To further evaluate accuracy of our algorithm in detecting and classifying EBRs we applied our methodology to a set of six genomes with a known history of simulated rearrangements. We designed a tree with four internal nodes and six leaves (Suppl. Fig. 2). Then, we simulated five rearranged genomes using RSVSim (Bartenhagen and Dugas 2013), starting with the DNA sequences of three chicken chromosomes (GGA8, GGA9 and GGA10) with a total length of ~72Mbp. We allowed only inversions and balanced translocations, keeping track of the coordinates of chromosome rearrangement events from the parent to the child branch. The number of introduced rearrangements for each branch was estimated using the avian genome dataset and ranged from 5 to 127 events, corresponding to 10-254 simulated EBRs in branches leading to A4 and L4, respectively (Suppl. Tables 3 and 4). The rearrangements in the internal nodes were hardcoded and non-overlapping, while rearrangements in leaves were placed randomly but avoiding the previously hardcoded regions. Finally, we reconstructed simulated HSBs using the L5 genome as a reference. To define the HSBs we translated the breakpoint coordinates obtained from RSVSim in each target genome to L5, by first detecting the intermediate ancestors between the two species, then translating the coordinates of the

target species to the intermediate ancestors, and finally translating the coordinates of the ancestors to L5. Therefore, we were able to record the genome coordinates and branch for each simulated EBR in L5. Then we defined HSBs at three levels of resolution: 7Kbp, 21Kbp and 35Kbp, corresponding to 100Kbp, 300Kbp and 500Kbp in our avian dataset because our simulated genomes were ~15 times smaller than the chicken genome.

Two sets of simulated genomes were used to test our methodology. One of them mimics six complete chromosome assemblies. Another set contains a combination of two 'scaffold-based' and four 'chromosome-based' assemblies (Suppl. Tables 3 and 4). In the set of combined scaffold- and chromosome-based assemblies, we used the same simulated genomes as in the first set but randomly broke HSBs in target species L2 and L6 to mimic N50 of 300 and 210Kbp equivalent to N50 of 5 and 3.5Mbp, respectively, in our avian genome set (Suppl. Table 4).

Our algorithm was applied to both simulated sets. As expected, our approach was not able to distinguish between the L1-specific and A1-specific EBRs, because L1 is an outgroup for all species from the A1 node. However, our algorithm correctly classifies them as 'L1 or A1-specific'.

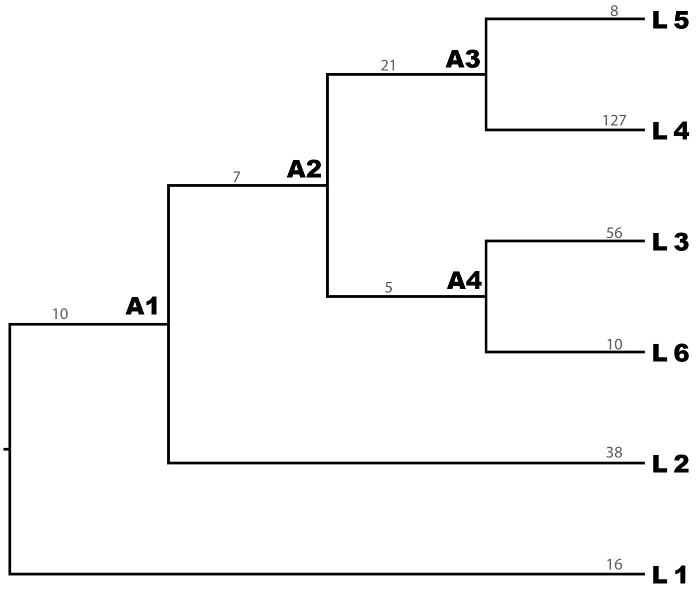

Supplementary Figure 2. Cladogram showing the phylogenetic relationships of the simulated genomes and number of simulated rearrangements on each branch. L5 was used as a reference genome in our analysis.

For the set of chromosome-level simulated genomes, β ranged from 0.0001 at 7Kbp for L6 to 0.000175 at 35Kbp for L3. For the combined set of chromosome- and scaffold-based simulated genomes, β ranged from 0.0303 at 7Kbp for L1 to 0.0533 at 35Kbp for L4.

For the set of simulated rearrangements in chromosome-level genomes, the match between our recorded positions and classifications of EBRs and those provided by the algorithm was 100% at 35Kbp resolution. At 21Kbp resolution, our algorithm detected all EBRs, but misclassified one EBR as A4-specific instead of A3-specific, representing a frequency of agreement in classification of 99.6%.

Although the ratio of two top hypothesis likelihoods for all other EBR ranged from 0.9995 to 0.9999 at 21Kbp resolution, this misclassified EBR had a very low ratio of 0.0001, which, following the filtering criteria we applied to our avian dataset, would be excluded. Finally, at 7Kbp resolution, our algorithm detected 3 more EBRs in L5 (Suppl. Table 3) than expected. These EBRs were defined using only 20% and 40% of the simulated genomes, while the rest were defined using all genomes in the dataset. Using our filtering criteria of the avian dataset, these 3 EBRs would also be excluded. Therefore, the results of this simulation demonstrated that our approach is accurate and robust in defining and classifying EBRs in chromosome-based assemblies.

Supplementary Table 3. Number of simulated rearrangements in each chromosome-based genome at three resolutions of rearrangement detection.

Species	No. simulated events	7Kbp			21Kbp			35Kbp		
		No. detectable EBRs	No. detected EBRs	% agreement	No. detectable EBRs	No. detected EBRs	% agreement	No. detectable EBRs	No. detected EBRs	% agreement
A1/L1	26	52	52	100	28	28	100	5	5	100
A2	7	14	14	100	8	8	100	4	4	100
A3	21	42	42	100	18	17	94.44	12	12	100
A4	5	10	10	100	6	7	85.71	4	4	100
L2	38	76	76	100	36	36	100	0	0	100
L3	56	112	112	100	51	51	100	0	0	100
L4	127	254	254	100	109	109	100	0	0	100
L5	8	16	16*	100	8	8	100	6	6	100
L6	10	20	20	100	11	11	100	0	0	100
Total (%)	298	596	596	100	275	275	100	31	31	100

*We detected 3 more EBRs than simulated, but they were filtered after applying the same criteria as in the avian dataset.

In the set of combined scaffold-based and chromosome-based genomes at 35Kbp resolution, 100% of the simulated EBRs were detected, while 88.73% and 93.70% of EBRs were detected at 21Kbp and 7Kbp resolutions, respectively. The decrease in detection between the simulated rearrangements in the chromosome-based only set (Suppl. Table 3) and the combined scaffold-based and chromosome-based genomes (Suppl. Table 4) is due to our algorithm excluding the putative breakpoints that are present between two simulated scaffolds. This exclusion leads to fewer EBRs detected in L2 and L6 than were originally simulated.

At 7Kbp resolution all but nine EBRs detected were correctly classified (representing 92.28% of all simulated EBRs) and the nine EBRs were labelled as ‘uncertain’, because the algorithm was not able to assign them to a single branch (Suppl. Table 4). At 21Kbp, 85.82% of all simulated EBRs were detected and correctly classified while eight EBRs were ‘uncertain’. One EBR was misclassified at 21Kbp resolution, with a low top hypothesis likelihoods ratio of 0.0001. Finally, at 35Kbp, 83.87% of the simulated EBRs were correctly classified, with 5 being ‘uncertain’ EBRs.

Overall, we demonstrated that our algorithm is capable of detecting and classifying EBRs in chromosome-based and scaffold-based assemblies. As expected, in the scaffold-based assemblies the fraction of EBRs detected is affected by assembly fragmentation. However, the accuracy of EBR classification does not suffer for those EBRs that are assigned to a single branch with a high hypothesis likelihoods ratio and high fraction of genomes being used in the classification.

Supplementary Table 4. Number of simulated rearrangements in each scaffold or chromosome-based genome at three resolutions of rearrangement detection.

Species	Type	No. simulated events	7Kbp			21Kbp			35Kbp		
			No. detectable EBRs	No. detected EBRs	% agreement	No. detectable EBRs	No. detected EBRs	% agreement	No. detectable EBRs	No. detected EBRs	% agreement
A1/L1	chr	26	52	52	100	28	28	100	5	5	100
A2	chr	7	14	11	78.57	8	5	62.50	4	1	25
A3	chr	21	42	42	100	18	17	94.44	12	12	100
A4	chr	5	10	4	40	6	2	33.33	4	2	50
L2	scaff	38	76	47	61.84	36	15	41.67	0	0	100
L3	chr	56	112	112	100	51	51	100	0	0	100
L4	chr	127	254	254	100	109	109	100	0	0	100
L5	chr	8	16	16*	100	8	8	100	6	6	100
L6	scaff	10	20	12	60	11	1	9.09	0	0	100
Total classified (%)	_	_	596	550	92.28	275	236	85.82	31	26	83.87
L3 or A4	_	_	_	6	_	_	5	_	_	2	_
L1 or A2	_	_	_	3	_	_	3	_	_	3	_
Total detected (%)	_	298	596	559	93.79	275	244	88.73	31	31	100

*We detected one more EBR than simulated but with a very low top hypothesis likelihoods ratio therefore filtered from the final EBR set.

5. Evolutionary Breakpoint Regions (EBRs) in avian genomes

We applied our new algorithm to identify and classify the EBRs in the 21 avian and five outgroup genomes. Using the SFs described previously, we set 100 Kbp as the basal resolution and counted as reuse EBRs those that had a likelihood ratio of 1 to 20 between the first and the second most likely hypothesis. At 100Kbp we identified 1,796 EBRs and 211 were classified as reuse (Suppl. Table 5).

Supplementary Table 5. Number of EBRs in each avian lineage with at least one EBR at 100Kbp resolution.

Classification	Detected no. EBRs	No. EBRs after filtering
**Lineage-specific**		
Pekin duck	113	NA
Emperor penguin	38	1
Anna's hummingbird	92	60
Chimney swift	45	21
Killdeer	25	4
Chicken	16	NA
Domestic pigeon	102	77
American crow	37	13
Common cuckoo	106	64
Little egret	40	18
Peregrine falcon	86	38
Medium ground finch	35	6
Golden collared manakin	35	23
Turkey	255	NA
Budgerigar	181	NA
Crested ibis	39	8
Hoatzin	39	19
Downy woodpecker	147	56
Adelie penguin	47	5
Ostrich	124	30
Zebra finch	47	47
**Clade-specific**		
Galliformes	42	NA
Galloanserae	15	NA
Trochiliformes + Apodiformes	2	NA
Ciconiiformes	3	NA
Passeroidea	14	NA
Passeroidea + Corvoidea	19	NA
Passeriformes	16	NA
Sphenisciformes	4	NA
Passeriformes + Psittaciformes + Falconiformes + Piciformes + Ciconiiformes + Sphenisciformes + Charadriiformes + Opisthocomiformes	2	NA
Non-galloanserae	11	NA
Non-galloanserae + non-columbiformes	1	NA
Neognathae	9	NA
Avian	9	NA
**Total/average EBRs**	1,796*	1,021*
**Reuse EBRs**	211 (11.75%)	92 (9.01%)

* Total number of EBRs does not include the reuse EBRs, because these were counted as lineage- or order-specific in corresponding lineages. The chicken genome coordinates of the EBRs could be found in Supplementary Table 6.

We compared the number of EBRs across resolutions and found that the turkey lineage has a large difference in the number of EBRs observed at 100Kbp and 300Kbp resolutions (Suppl. Fig. 3). The most likely explanation is that the turkey genome has a large number of local misassemblies at < 300Kbp resolution. This is consistent with previous findings using the hybrid Illumina+454 turkey genome assembly (Zhang, et al. 2014b). We did not observe similar trends in any other bird genome (Suppl. Fig. 3). Therefore, we decided to exclude turkey from subsequent analyses.

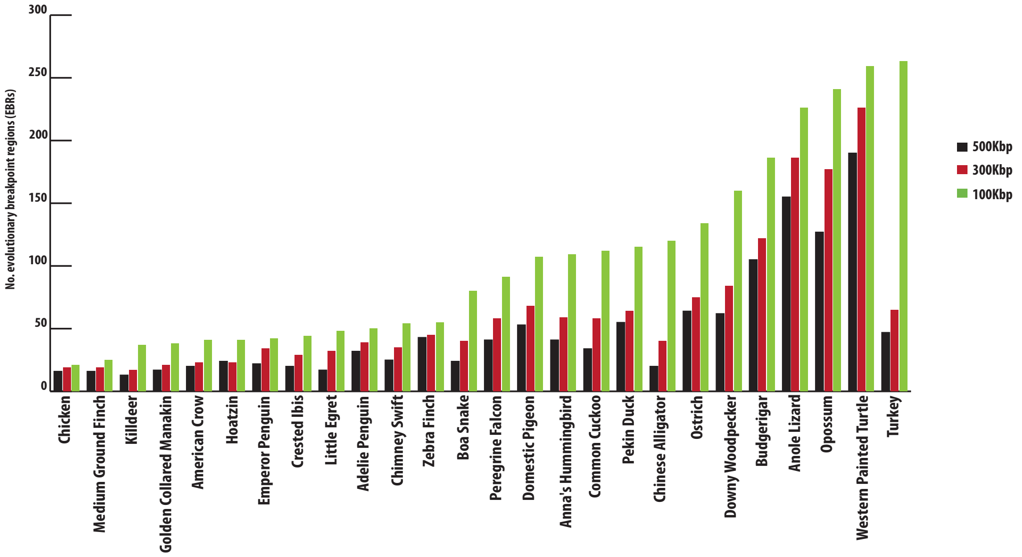

Supplementary Figure 3. Number of evolutionary breakpoint regions (EBRs) detected in each species using three resolutions for HSB detection. Black bars represent 500Kbp resolution; 300Kbp resolution is represented in red and 100Kbp resolution in green bars. Note that while all species have ~1.4x increase in the number of EBRs for adjacent resolutions, turkey has ~4x more EBRs at the 100Kbp resolution than at 300Kbp.

6. Evaluating EBR qualities and removal of chimeric joints from the EBR lists for genomes assembled without a map aid.

In order to remove most chimeric joints within scaffolds that could mimic EBRs in our dataset and obtain estimates of the fraction of false positive EBRs (chimeric joints) in our final filtered set, we followed the steps below.

All EBRs found in a single species assembly only (lineage-specific EBRs) were translated from the reference genome (chicken) coordinates into the corresponding target species scaffold coordinates. For three species assemblies, representative of the range of genome sequencing coverage (pigeon (63x), ostrich (85x) and peregrine falcon (105x)), PCR amplification across a subset of the EBR intervals (91 total) ranging from 245bp to 5.3Kbp was attempted (Suppl. Fig. 4). In parallel, we mapped all the read libraries used to assemble target genomes (Zhang, et al. 2014b) to the species scaffolds using Bowtie2 (Langmead and Salzberg 2012). Then, we computed the minimum number of

pair-end reads *spanning* any base pair position(s) within the EBR intervals mentioned above as well as genome average spanning (Suppl. Fig. 5).

We then compared the presence/absence of a successful PCR amplification (a PCR product of an expected size) with the calculated minimum pair-end read spanning within the corresponding EBR. This allowed us to establish the minimum read spanning threshold for the EBRs, which would result in minimum number of non-amplifiable (likely chimeric) EBRs for each representative assembly (Suppl. Table 7). To confirm that negative PCR results represented chimeric joints and not failed PCRs, where possible, we selected primers spanning putative chimeric boundaries and the adjacent scaffold (PCR product < 6Kbp), according to the pair-wise alignments to chicken as reference and assuming no rearrangement in that region. We tested eight such joints and we were able to confirm seven as chimeric (one in peregrine falcon, two in ostrich and four in pigeon).

For peregrine falcon, we established the minimum base spanning threshold of 582, leading to estimated 0% of chimeric joints being included in our peregrine falcon filtered lineage-specific EBR set. On the other hand, this threshold corresponds to a rate of false negative of 33.33%, indicating that ~33% of excluded EBRs after applying the filtering step could represent real lineage-specific EBRs (Suppl. Table 7 and Suppl. Fig. 6).

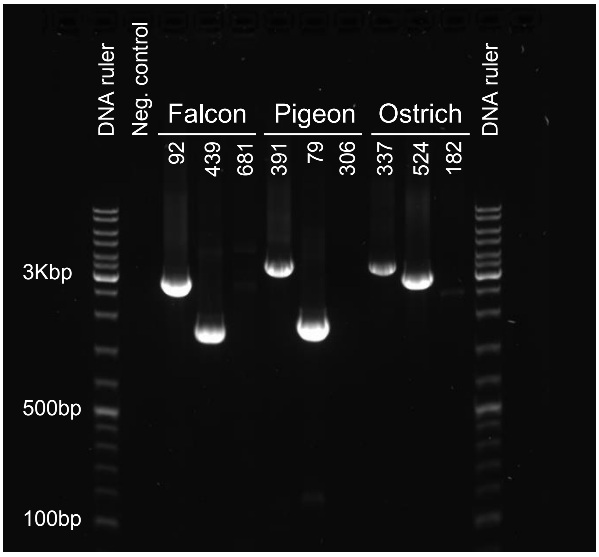

Supplementary Figure 4. EBR verification by PCR analysis. Numbers in each lane represent scaffold IDs. For each species, 3 EBRs in 3 different scaffolds are shown. Negative results (chimeric joints) are shown for scaffolds 681, 306 and 182 in peregrine falcon, pigeon and ostrich, respectively.

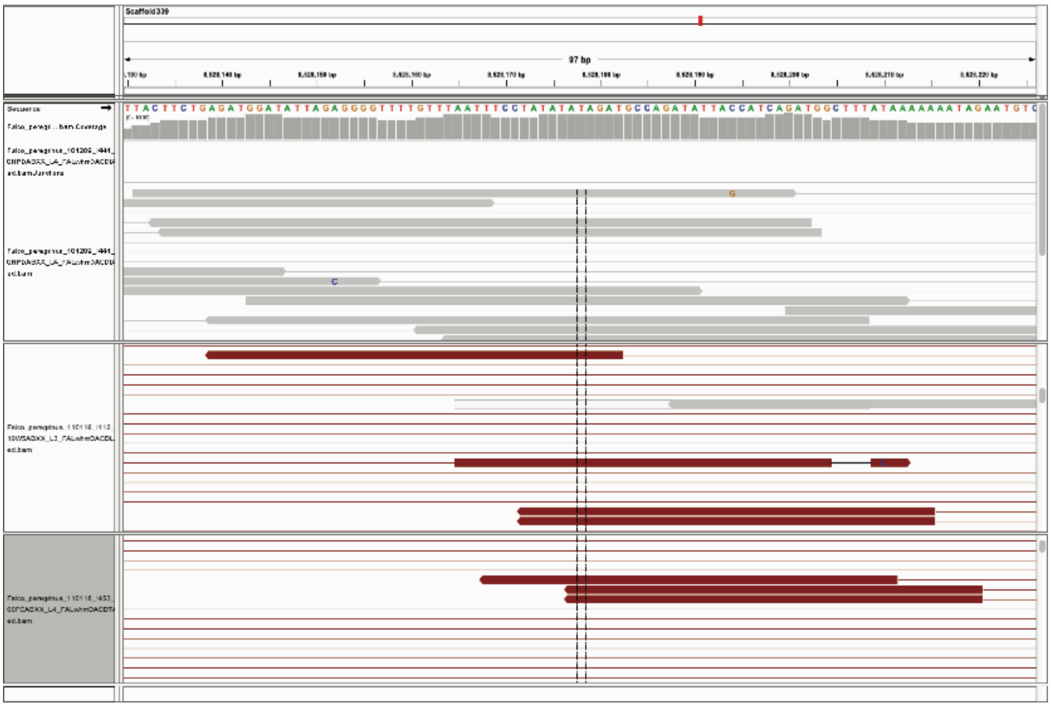

Supplementary Figure 5. Alignments of three pair-end read libraries to peregrine falcon genome. The *spanning* coverage was calculated as the number of pair-end reads mapping to a single-base and the number of read pairs spanning this position. In this example, the position 8,528,178, highlighted with dotted lines, has a spanning coverage of 46 but read coverage of 15.

We then used these spanning thresholds established for falcon, pigeon and ostrich to identify likely chimeric joints in the EBRs in the rest of the genome assemblies that had similar average genome-wide pair read spanning coverage. The EBRs with the minimum pair read spanning higher than the corresponding thresholds were included for further analyses. On average, more than 40% of the lineage-specific EBRs were kept for further analyses ranging from 2.63% to 75.49% in Emperor penguin and pigeon, respectively (Suppl. Table 8).

Supplementary Table 7. PCR results and minimum base spanning threshold for 3 species.

		**Pigeon**	**Ostrich**	**Peregrine falcon**
	Sequencing coverage	63x	85x	105x
	Minimum base spanning threshold	84	216	582
	EBRs in PCR range (< 6Kbp)	69	50	14
	No. EBRs PCR tested	30	47	13
	No. chimeric EBRs detected	4	2	1
**EBRs below minimum base spanning**	In agreement with PCR	4	6	4
	In disagreement with PCR	3	5	2
	Estimated false negative rate	42.86	45.45	33.33
**EBRs above minimum base spanning**	In agreement with PCR	18	32	7
	In disagreement with PCR	5	4	0
	Estimated false positive rate	21.74	11.11	0.00

Supplementary Table 8. Number of detected and maintained EBRs for the genomes assembled to a scaffold level.

Lineage	Genome coverage	Average spanning in scaffolds	Spanning threshold	No. lineage EBRs in reference	No. lineage non chimeric EBRs	% lineage non chimeric EBRs
Ostrich	85x	524.08	216	124	30	24.19
Peregrine falcon	105x	838.44	582	66	38	57.58
Pigeon	63x	331.39	84	102	77	75.49
Chimney swift	103x	611.32	216	45	21	46.67
Killdeer	100x	629.18	216	25	4	16.00
Emperor penguin	61x	446.57	84	37	1	2.63
Medium ground finch	115x	818.19	582	34	6	17.65
American crow	80x	1075.78	582	37	13	35.14
Common cuckoo	100x	892.41	582	103	64	62.14
Anna's hummingbird	110x	1019.18	582	91	60	65.93
Little egret	74x	682.23	216	40	18	45.00
Golden-collared manakin	110x	1108.75	582	35	23	65.71
Downy woodpecker	105x	777.49	582	146	56	38.36
Crested ibis	105x	1307.46	582	38	8	21.05
Adelie penguin	60x	556.49	216	38	5	13.16
Hoatzin	100x	567.23	216	38	19	50.00

For additional verification of EBRs in genomes assembled with an aid of maps, we translated duck lineage-specific EBRs into the duck genome coordinates. Then, by comparing the duck scaffolds and the chromosome assembly assisted by a radiation hybrid (RH) map, we detected chimeric scaffolds. Only 16 lineage-specific EBRs (6.99%) in the duck genome were found in scaffolds detected as chimeric by the RH map. But only one of them (0.44%) overlapped with a scaffold interval detected as a chimeric joint in the RH map suggesting that 99.56% of duck lineage-specific EBRs are located in well-assembled regions of the duck genome. This indicates that map-assisted assemblies (except turkey, see above) are likely to contain a very low number of chimeric joints, and all detected EBRs could be used for further statistical analyses.

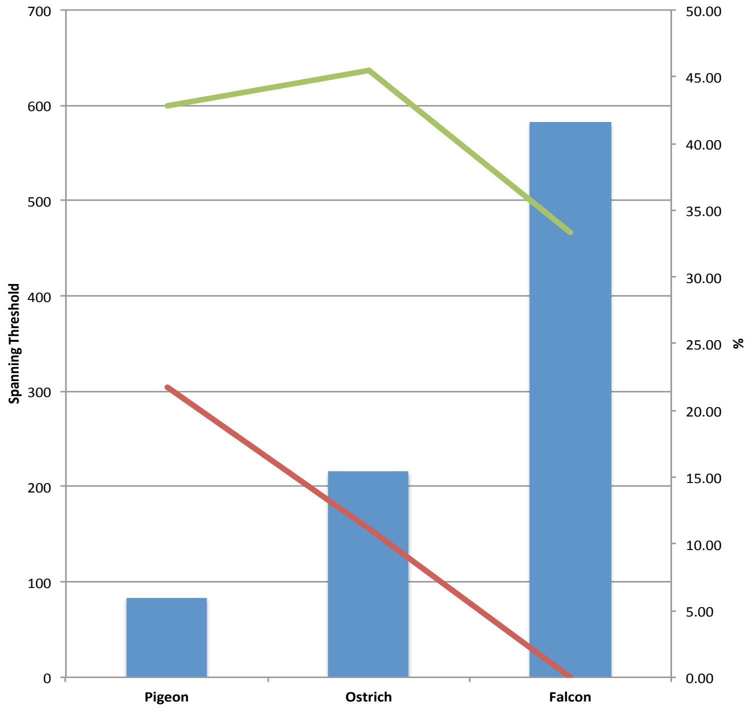

Supplementary Figure 6. Spanning threshold coverage in three representative species. Blue bars show the threshold coverage established using pair-read mapping and PCR results. Red line represents the percentage of chimeric joints counted as EBRs, while the green line represents the percentage of real EBRs discarded in the subset of eliminated EBRs.

7. Comparing densities of transposable elements (TEs) in EBRs and other parts of bird genomes.

We first compared the total number of TEs across all bird genomes, to investigate if NGS assemblies have a lower number of TEs due to assembly issues in repetitive regions. Consistent with an analysis in a companion study (Zhang, et al. 2014b), we found that the overall number of TEs in NGS bird genomes was similar to those genomes assembled using the Sanger method (e.g chicken and zebra finch) (Suppl. Fig. 7). Likewise, the Downy woodpecker was an outlier and had 2.17×10^8, ~2.5 times more TEs. This demonstrates that our TE identification approach works well.

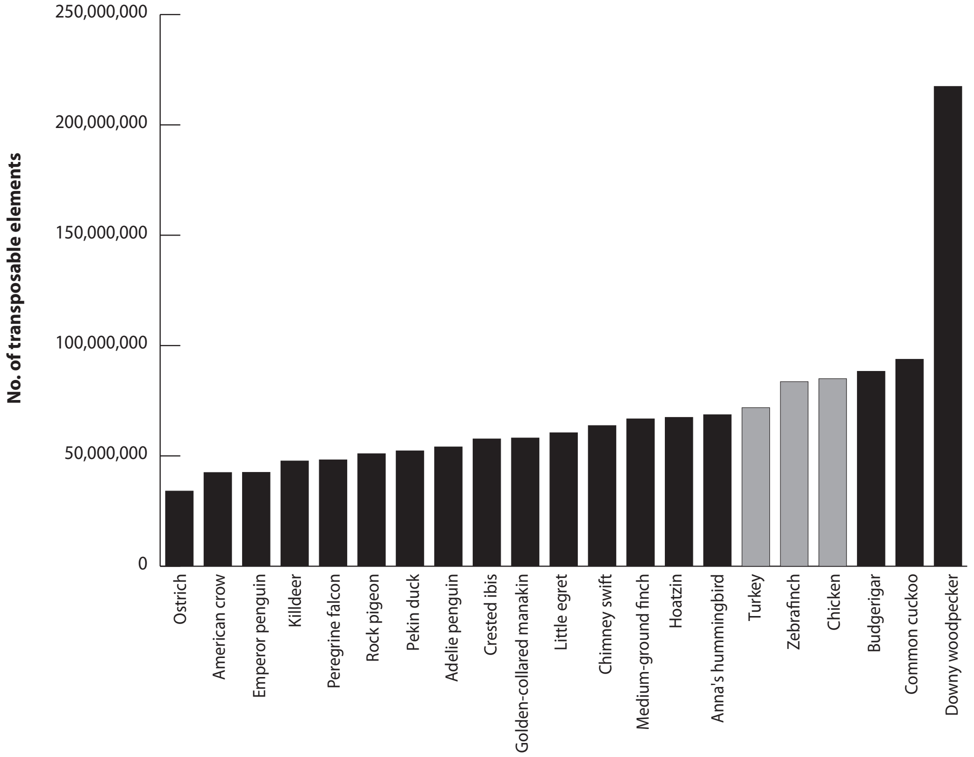

Supplementary Figure 7. Total number of transposable elements (TEs) in bird genomes. Black bars represent genomes assembled using next generation sequencing (NGS), while grey bars represent genomes assembled using Sanger sequencing.

8. Defining msHSBs sets and testing the random breakage model on these sets

Multispecies homologous synteny blocks were defined as the regions of reference chromosomes that had no EBRs or uncertain breakpoint regions detected in a set of species included in the msHSBs detection. Five sets of msHSBs were defined: (i) avian msHSBs, found in all bird species, (ii) archosaurian msHSBs, found in birds and crocodiles, (iii) archosaurian/testudines msHSBs, found in birds, crocodiles, and turtles, (iv) sauropsida msHSBs, found in all reptile species, and (v) amniote msHSBs, identified in all species studied. The distribution of msHSB sizes was tested for goodness-of-fit to an exponential distribution using the Kolmogorov-Smirnov test following Pevzner & Tesler (Pevzner and Tesler 2003) and Larkin et al (Larkin, et al. 2009) (Suppl. Table 9). The random breakage model was tested according to (Churchill, et al. 1990) and (Pevzner and Tesler 2003). The Kolmogorov-Smirnov test of goodness-of-fit to an exponential distribution was used to measure the largest difference between the observed and theoretical distribution (Pevzner and Tesler 2003). The computed Kolmogorov-Smirnov statistic (D) for each msHSBs dataset was compared to the critical value of 0.075 (α=0.01; (Massey 1951)) (Suppl. Table 9). Excluding the largest msHSBs in each set, we found that msHSBs size distribution followed an exponential distribution in all msHSB sets except archosaurian/testudines. However, we detected 21 msHSBs longer than the maximum

lengths expected from a random distribution of EBRs (Suppl. Table 10 and Suppl. Table 11), indicating that large msHSBs could be maintained in evolution of bird and other reptile genomes (Suppl. Table 10), including six large amniote, four reptile, three archosaurian/testudines, three archosaurian, and five avian msHSBs that were longer than the maximum lengths expected from a random distribution of evolutionary breakpoints (Suppl. Table 10). From these, one amniote msHSB on GGA1, two sauropsid msHSBs on GGA6 and GGA3, one archosaurian/testudines msHSB on GGA3, one archosaurian msHSB and one avian msHSB on GGA1 were significantly larger than expected if EBRs were distributed randomly (p-value <0.05; Suppl. Table 10 and Suppl. Table 11).

Supplementary Table 9. Kolmogorov-Smirnov test (D) in each msHSB set.

msHSB set	D with all msHSBs	D excluding largest msHSBs
Amniote	0.079	0.073
Reptile	0.076	0.071
Archosaurian/Testudines	0.082	0.077
Archosaurian	0.081	0.074
Avian	0.078	0.072

The expected maximum msHSB sizes (S) were calculated following an exponential distribution as $L[\gamma + \ln(n+1)]$, where L is the mean msHSB length, n is the number of msHSBs and $\gamma = 0.5772$ is Euler's constant. And the probability to find an msHSB under the Poisson process was calculated as $Pr(x(n)>S) = 1-\exp(-\exp(\ln(n)-S/L))$, where x is the msHSB size, n is the total number of msHSBs and S the expected maximum msHSB size, following previous publications (Churchill, et al. 1990; Larkin, et al. 2009).

Supplementary Table 10. Multispecies Homologous Synteny Blocks (msHSBs) present in different subsets of species.

Statistics	Avian	Archosaurian	Archosaurian & Testudines	Sauropsid	Amniote
No. msHSBs	1,746	1,634	1,606	1,592	1,514
Total length (Mbp)	765.21	665.03	651.74	545.95	534.92
Coverage of chicken genome (%)	76.29	66.3	64.98	54.43	53.33
Max length (Mbp)	4.81	4.46	4.67	4.67	4.17
Expected max length (Mbp)*	3.52	3.25	3.06	2.73	2.79
No.msHBSs > 1.5Mbp	85	67	62	45	44
Coverage of chicken genome of long msHSBs (%)	18.12	14.07	13.17	9.16	8.03
No. genes in long msHSBs	1,315	1,024	959	706	676
Percentage of total genes (10,830)	12.14	9.45	8.85	6.52	6.24

* The expected maximum msHSB lengths were calculated assuming an exponential distribution as $L[\gamma + \ln(n+1)]$, where L is mean msHSB length, n is the number of msHSBs, and $\gamma = 0.5772$ is Euler's constant (Churchill, et al. 1990). All msHSB set chicken genome coordinates are reported in Supplementary Table 11.

References

Alföldi J, et al. 2011. The genome of the green anole lizard and a comparative analysis with birds and mammals. Nature 477: 587-591. doi: 10.1038/nature10390

Bartenhagen C, Dugas M 2013. RSVSim: an R/Bioconductor package for the simulation of structural variations. Bioinformatics 29: 1679-1681. doi: 10.1093/bioinformatics/btt198

Bradnam KR, et al. 2013. Assemblathon 2: evaluating *de novo* methods of genome assembly in three vertebrate species. Gigascience 2: 10. doi: 10.1186/2047-217x-2-10

Churchill GA, Daniels DL, Waterman MS 1990. The distribution of restriction enzyme sites in *Escherichia coli*. Nucleic Acids Research 18: 589-597.

Dalloul RA, et al. 2010. Multi-platform next-generation sequencing of the domestic turkey (*Meleagris gallopavo*): genome assembly and analysis. PLoS Biol 8. doi: 10.1371/journal.pbio.1000475

Donthu R, Lewin HA, Larkin DM 2009. SyntenyTracker: a tool for defining homologous synteny blocks using radiation hybrid maps and whole-genome sequence. BMC Res Notes 2: 148. doi: 10.1186/1756-0500-2-148

Elsik CG, et al. 2009. The genome sequence of taurine cattle: a window to ruminant biology and evolution. Science 324: 522-528. doi: 10.1126/science.1169588

Ganapathy G, et al. 2014. High-coverage sequencing and annotated assemblies of the budgerigar genome. Gigascience 3: 11. doi: 10.1186/2047-217x-3-11

Grabherr MG, et al. 2010. Genome-wide synteny through highly sensitive sequence alignment: Satsuma. Bioinformatics 26: 1145-1151. doi: 10.1093/bioinformatics/btq102

ICGSC 2004. Sequence and comparative analysis of the chicken genome provide unique perspectives on vertebrate evolution. Nature 432: 695-716. doi: 10.1038/nature03154

Jarvis ED, et al. 2014. Whole-genome analyses resolve early branches in the tree of life of modern birds. Science 346: 1320-1331. doi: Doi 10.1126/Science.1253451

Kent WJ, et al. 2002. The human genome browser at UCSC. Genome Research 12: 996-1006. doi: 10.1101/gr.229102. Article published online before print in May 2002

Langmead B, Salzberg SL 2012. Fast gapped-read alignment with Bowtie 2. Nature methods 9: 357-359. doi: 10.1038/nmeth.1923

Larkin DM, et al. 2009. Breakpoint regions and homologous synteny blocks in chromosomes have different evolutionary histories. Genome Research 19: 770-777. doi: 10.1101/gr.086546.108

Massey FJ 1951. The Kolmogorov-Smirnov Test for Goodness of Fit. Journal of the American Statistical Association 46: 68-78. doi: 10.1080/01621459.1951.10500769

Mikkelsen TS, et al. 2007. Genome of the marsupial Monodelphis domestica reveals innovation in non-coding sequences. Nature 447: 167-177. doi: 10.1038/nature05805

Partipilo G, D'Addabbo P, Lacalandra GM, Liu GE, Rocchi M 2011. Refinement of *Bos taurus* sequence assembly based on BAC-FISH experiments. BMC Genomics 12: 639. doi: 10.1186/1471-2164-12-639

Pevzner P, Tesler G 2003. Human and mouse genomic sequences reveal extensive breakpoint reuse in mammalian evolution. Proceedings of the National Academy of Sciences of the United States of America 100: 7672-7677. doi: 10.1073/pnas.1330369100

Ross SM. 1996. Simulation. San Diego: Academic Press.

Shaffer HB, et al. 2013. The western painted turtle genome, a model for the evolution of extreme physiological adaptations in a slowly evolving lineage. Genome Biology 14: R28. doi: 10.1186/gb-2013-14-3-r28

Specht DF 1991. A general regression neural network. IEEE Trans Neural Netw 2: 568-576. doi: 10.1109/72.97934

Wan Q-H, et al. 2013. Genome analysis and signature discovery for diving and sensory properties of the endangered Chinese alligator. Cell Res 23: 1091-1105. doi: 10.1038/cr.2013.104

Warren WC, et al. 2010. The genome of a songbird. Nature 464: 757-762. doi: 10.1038/nature08819

Zhan X, et al. 2013. Peregrine and saker falcon genome sequences provide insights into evolution of a predatory lifestyle. Nat Genet 45: 563-566. doi: 10.1038/ng.2588

Zhang G, et al. 2014a. Comparative genomic data of the Avian Phylogenomics Project. Gigascience 3: 26. doi: 10.1186/2047-217x-3-26

Zhang G, et al. 2014b. Comparative genomics reveals insights into avian genome evolution and adaptation. Science 346: 1311-1320. doi: 10.1126/science.1251385
